# Author Correction: Spatial fibroblast niches define Crohn’s fistulae

**DOI:** 10.1038/s41586-025-09956-2

**Published:** 2025-11-25

**Authors:** Colleen McGregor, Xiao Qin, Marta Jagielowicz, Tarun Gupta, Zinan Yin, Verena Lentsch, David Fawkner-Corbett, Vy Wien Lai, Paula Gomez Castro, Esther Bridges, Chloe Hyun-Jung Lee, Huei-Wen Chuang, Lei Deng, Anna Aulicino, Renuka Teague, Sorayya Moradi, Jun Sung Park, Jeongmin Woo, Kexin Xu, Ruchi Tandon, Nicole Cianci, Jan Bornschein, Ling-Pei Ho, Paulina Siejka-Zielinska, Zoe Christoforidou, Sarah Hill, Johannes Lehmann, Rhea Kujawa, Paola Vargas Gutierrez, Carol Cheng, Maria Greco, Katherine Baker, Mark Bignell, Bruce George, Eve Fryer, Michael Vieth, Agne Antanaviciute, Alison Simmons

**Affiliations:** 1 Medical Research Council Translational Immune Discovery Unit (MRC TIDU), Weatherall Institute of Molecular Medicine (WIMM), University of Oxford, Oxford, UK; 2 Translational Gastroenterology and Liver Unit, John Radcliffe Hospital, Oxford, UK; 3 Academic Paediatric Surgery Unit, Nuffield Department of Surgical Sciences, University of Oxford, Oxford, UK; 4 MRC WIMM Centre for Computational Biology, WIMM, Oxford, UK; 5 Oxford Centre for Histopathological Research, Oxford University Hospitals NHS Trust, Oxford, UK; 6 Department of Cellular Pathology, Oxford University Hospitals NHS Trust, Oxford, UK; 7 Center for Human Genetics, Nuffield Department of Medicine, University of Oxford, Oxford, UK; 8 Kennedy Institute of Rheumatology, University of Oxford, Oxford, UK; 9 MRC WIMM Advanced Single Cell OMICS Facility, WIMM, Oxford, UK; 10 Department of Colorectal Surgery, Oxford University Hospitals NHS Trust, Oxford, UK; 11 Institute of Pathology, Friedrich-Alexander University Erlangen-Nuremberg, Klinikum Bayreuth, Bayreuth, Germany

**Keywords:** Mucosal immunology, Crohn's disease, Immunopathogenesis, Crohn's disease, Experimental models of disease

Correction to: *Nature* 10.1038/s41586-025-09744-y Published online 12 November 2025

In the version of the article initially published, the “*TWIST1*” and “*OSR2*” labels in Fig. 2c were switched and have now been amended so that the columns labels (from left to right) read “Control, *ORS2*, *TWIST1*” in the HTML and PDF versions of the article.

