# Supplementary material for: Spatial fibroblast niches define Crohn’s fistulae
Source: Nature. 2025 Nov 12;649(8097):703–12. doi: 10.1038/s41586-025-09744-y (PMC12804086; doi:10.1038/s41586-025-09744-y)
Supplement: Supplementary file 1 — This file contains Supplementary Tables 2 and 3, and Supplementary Figures. Supplementary Table 2: Meta-analysis and literature review table summarizing fibroblast subsets associated with IBD identified across single-cell transcriptomic studies. Supplementary Table 3: Summary of cell numbers obtained per sample across all assays. Supplementary Figures: Supplementary Figures summarizing additional immunohistochemistry data, FACS gating strategy, scRNA-seq and ST dataset quality control metrics, gene markers and additional analyses. [file 41586_2025_9744_MOESM1_ESM.pdf]

nature portfolio

https://doi.org/10.1038/s41586-025-09744-y

Supplementary information

Spatial fibroblast niches define Crohn's fistulae

In the format provided by the authors and unedited

Supplementary Table 2: Meta analysis and literature review table summarising fibroblast subsets associated with inflammatory bowel disease identified across single-cell transcriptomic studies.

Novel Class	Markers	Gene Expression	Proposed Function	Tissue Location (CO: Colon, IL: Ileum, PA: Perianal)	Full Thickness vs. Biopsy	Disease: CD/UC Phenotype	Reference	Shares Features With Clusters In Integrated Data
**Inflammatory fibroblasts CHI3L1hi**	CHI3L1, MMP1	MMP1, MMP3, MMP13, MT1E, MT1M, MT1X, FOSB, EGR1, GLUL, FOS, CHI3L2, CHI3L1, CXCL13, CLU, CA12, NFKBIZ, SOD2	Pro-fibrotic, cellular movement, invasion and migration, leucocyte migration	PA	Fistula tract biopsy	CD<br><br>Perianal fistula	Levantovsky et al. 2024	S1-CHI3L1+<br>S2-CHI3L1+<br>S3-CHI3L2+<br>S3-FAP+<br>S3-FOSL1+
**Inflammation-Associated Fibroblasts (IAFs) – IL13RA2/IL11^{+}**	FAP, TWIST1, IL13RA2, IL11	IL11, IL24, IL13RA2, WNT2B, WNT5A, WNT5B, FAP, TWIST1, WNT2, PLAU, CHI3L1, MMP3, TNFSF11, MMP10, IL1R1, OSMR, STRA6, IL1B, GOS2, IL8, IL10, CCL11, TNFRSF11B, NOTCH2NL, DKK3, CXCL12, BMP4, BMP5	Mediate anti-TNF drug resistance, cell-to-cell communication, pro-fibrotic	CO	Biopsies	UC	Smillie et al. 2019	S2-CHI3L1+
**Activated fibroblasts**	CD90, PDPN,CTHRC1, CHI3L1, PDGFRA, MMP2	CXCL13, CXCL6, CXCL16, CXCL1, CXCL2, CXCL5, CXCL8, CCL2, CCL7, IL6, IL11	Pro-inflammatory - neutrophil and monocyte recruitment,<br>Pro-fibrotic	IL	Biopsies	CD	Martin et. al 2019	S1-CHI3L1+<br>S2-CHI3L1+
**S4**	CCL19, CCL21, TNFSF14, CD74	CCL21, CXCL13, TNFSF11, CD24, OLFM3, PTGDS, JAK3, PSMD2, IL32, IL33, CXCL2, CXCL3, TNFSF14, FDCSP, PDPN, C3	Pro-inflammatory, immunomodulatory	CO	Biopsies	UC	Kinchen et al. 2018	S4
**IAF**	IL1B, IL6	TWIST1, PRRX1, TNC, COL1A2, F3, APOE, CXCL1, ADAMDEC1, MMP3, IL11, CXCL5, WNT5A, BMP1, IL1B, IL24, IL6	Inflammatory signalling, stress adaptation, extracellular matrix organisation	CO	Biopsies	UC (Paediatric)	Huang et al. 2019*	S1-CHI3L1+<br>S2-CHI3L1+
**Inflammatory fibroblasts IL11^{+}CHI3L1^{+}**	CHI3L1, IL11, MMP3, MMP1, TNC	SOD2, TIMP1, COL6A3, TGFBI, CHI3L1, PHLDA1, COL1A1, IGFBP5A, SPON2, TNC, PCOLCE, IER3, MMP3	Tissue remodelling, pro-fibrotic and immunomodulatory signalling	CO	Biopsies	CD<br><br>Stricturing	Kong et al. 2023	S1-CHI3L1+<br>S2-CHI3L1+<br>S3-CHI3L2+<br>S3-FAP+<br>S3-FOSL1+

**LUM⁺ fibroblasts (Fibroblast cluster 9 (C9) and 12 (C12))**	LUM<br>C9: DCN<br>C12: JUN	COL4A1, COL4A2, COL15A1, COL6A3, COL18A1, ADAMDEC1, LAMB1, GREM1	C9 & C12: Pro-fibrotic, ECM and tissue structure organisation, C9: WNT signalling regulation	IL	Full thickness	CD<br><br>Stricturing	Humphreys et al. 2024*	All S1, S3 subsets
**MMP⁺/WNT5A⁺ fibroblasts**	MMP1, MMP3, WNT5A	SOD2, IER3, COL1A2, COL3A1, COL5A1, MMP1, MMP3, FTH1, MT-CO2, MT-CO1, CD11, PHLDA1, CCN2, IL1R1, CD11, PLAU, CHI3L1, WNT2, IL24	Intercellular interactions – Principal signal senders in fibrosis	IL	Full thickness	CD<br><br>Stricturing	Mukherjee et al. 2023*	S1-CHI3L1+<br>S2-CHI3L1+<br>S3-CHI3L2+<br>S3-FAP+<br>S3-FOSL1+
**CXCL14/ADAMDEC1⁺**	ADAMDEC1, CXCL14, CCL11	CXCL14, APOE, FABP5, IGFBP7, COL3A1, COL18A1, IL1R1, MT-CO2, MT-CO1, CDH11	Intercellular interactions – Central signal senders in fibrosis<br>Tissue remodelling – increased ECM production	IL	Full thickness	CD<br><br>Stricturing	Mukherjee et al. 2023*	All S1 subsets
**CXCL14/F3/PDGFRA⁺**	F3, CXCL14, PDGFRA, POSTN	MT-CO2, MT-CO1, IGFBP3, PHGR1, CDH11, PLAU, ILIR1	Intercellular interactions – Central signal senders in fibrosis<br>Tissue remodelling – increased ECM production	IL	Full thickness	CD<br><br>Stricturing	Mukherjee et al. 2023*	All S2 subsets
**S4 Fibroblast (FLC)**	CCL19, FDCSP	COL1A1, COL6A1, FOXL1, ADAMDEC1, ZEB2, TAGLN, APOE	Pro-inflammatory signalling, epithelial-stromal crosstalk, WNT-mediated TA cell signalling, TNF-related epithelial apoptosis, altered epithelial cell composition	IL	Biopsies	CD (Paediatric)	Elmentaite et al. 2020	S4<br>S1 CXCL10+
**CXCL10⁺CCL19⁺**	CCL19, CCL2, CCL13, CD74, HLA-DRA, VCAM1, CCL5	CCL19, CXCL19, CD74, HLA-DRA, RBP5, CCL21, CXCL10, IRF8M, OASL, CXCL11, IL32, TNFSF13B, HLA-DRB1	Immune cell interaction, antigen presentation, T cell activation, strong interferon response, pro-inflammatory signalling.	CO	Biopsies	UC	Korsunsky et al. 2022*	S1 CXCL10+<br>S4
**SPARC⁺COL3A1⁺**	COL11A1, SPARC, LRRC15, MMP13, MMP11, COL3A1 COL1A1, TGFB1	CTHRC1, COL1A1, KIF28B, POSTN, ADAM12, COL3A1, MMP13, SPARC, CTHRC1, KIF26B, COL11A1, CRABP2, LRRC15, MMP11, FAP, COL5A1, TGFB1	ECM remodelling, tissue development, matrix disassembly, structural support.	CO	Biopsies	UC	Korsunsky et al. 2022*	S3-FAP+<br>S3-CHI3L2+<br>S2-CHI3L1+
**Activated fibroblasts**	CD10, TIMP1, IL1R1, CD44, CXCL14, MMP1, MMP3	TIMP1, MMP1, MMP3, AREG, TMEM158, TNFRSF11B, PDGFRA	Increased signature in vedolizumab non-responders	CO	Biopsies	UC	Mennillo et al. 2024*	S1-CHI3L1+<br>S2-CHI3L1+<br>S3-CHI3L2+<br>S3-FAP+

								S3-FOSL1+
**TWIST1⁺FAP⁺ fibroblasts**	TWIST1, FAP, CHI3L1, CTHRC1, IGFBP4, GREM1, CTSK, THBS2, C3, FBLN2	COL1A1, COL3A1, FAP, TWIST1, CHI3L1, NT5E, GREM1, FNDC1	Tissue remodelling – ECM and structural organisation	IL	Full thickness	CD<br><br>Stricturing	Zhang et al. 2024*	S3-FOSL1+<br>S3-FAP+<br>S3-CHI3L2+
**FAP⁺ fibroblasts**	FAP, CD82, TWIST1, POSTN	TWIST1, SIX2, PRRX2, MSX2, HIF1A, CD90, IL6, IL11, TGFB1, CXCL1, CXCL5, CXCL6, CCL2, CCL7, IL32, CXCL16, IL32, CXCL10	Fibrosis, immune modulation and inflammatory cell recruitment	IL	Full thickness	CD<br><br>Stricturing	Ke et al. 2024*	S1-CXCL10+<br>S1-CHI3L1+<br>S2-CHI3L1+<br>S3-CHI3L2+<br>S3-FAP+<br>S3-FOSL1+
**THY1⁺FAP⁺PDPN⁺ fibroblast**	THY1, PDPN, FAP, WNT2B, POSTN, CD74,	FTH1, SOD2, PDPN, NNMT, LUM, TIMP1, RARRES2, CXCL1, PHLDA1, COL3A1, PLAU, COL6A3, CHI3L1, COL1A1, TNFAIP6, DCN, PDGFRA, PDGFRB, ABCA8, WNT2B, FOS, SOX6, POSTN, CD74, C3, TNC, NRG1, LOCL2	Anti-TNF non-remission	IL, CO	Biopsies	CD, UC	Thomas et al. 2024	S2-CHI3L1+<br>S3-CHI3L2+<br>S3-FAP+
**PDPN⁺ Inflammatory/Activated Fibroblasts**	PDPN, MMP1, SOD2	CHI3L1, MMP1, PDPN, SOD2, COL7A1, MT2A, MMP3, SERPINE1, CEMIP, SCHLAP1, IGHG1, IL11, IGKC, CXCL5, IGLC3, CXCL1	Central regulator of immune-stromal interactions	IL	Full thickness	CD	Li et al. 2024	S1-CHI3L1+<br>S2-CHI3L1+<br>S3-CHI3L2+<br>S3-FAP+<br>S3-FOSL1+
**Inflammatory fibroblasts**	PDPN, THY1, FAP	MMP13, MMP3, MMP1, CRABP2, CXCL5, DPT, CXCL6, TNFAIP6, IL11, PTGFR, FDCSP, CA12, OGN, CHI3L1, ADAM12, PDPN	IL-1R dependent neutrophil-chemoattraction	IL, CO	Biopsies<br><br>Full thickness	UC, CD	Friedrich et al. 2021*	S1-CHI3L1+<br>S2-CHI3L1+<br>S3-CHI3L2+<br>S3-FAP+<br>S3-FOSL1+
Adult samples unless stated otherwise. *Studies not included in meta-analysis.<br>Key: CD, Crohn's disease; ECM, Extracellular matrix; UC, Ulcerative Colitis								

Sample Group	Mean Cells	SD Cells	N Samples	Assay
EXTERNAL: ENTERO/COLOCUTANEOUS	155255	133381	6	Xenium 480-plex
EXTERNAL: PERI-ANAL	100494	64007	6	Xenium 480-plex
INTERNAL/SMALL/LARGE INTESTINE	161682	90525	29	Xenium 480-plex
NOT FISTULISING	87119	70793	12	Xenium 480-plex
FISTULA	77810	46768	5	Xenium 5100-plex
HC	65678	47880	7	Xenium 5100-plex
ULCER	144510	46827	7	Xenium 5100-plex
DIVERTICULAR	3509	962	4	Visium
EXTERNAL: ENTERO/COLOCUTANEOUS	3437	588	3	Visium
EXTERNAL: PERI-ANAL	2706	621	8	Visium
INTERNAL/SMALL/LARGE INTESTINE	3496	1016	11	Visium
NOT FISTULISING	1942	683	7	Visium
Fist.Inv	6448	3579	7	scRNA-Seq
Fist.N.Inv	8611	3283	3	scRNA-Seq
Inf.Inv	5053	2975	6	scRNA-Seq
Inf.N.Inv	6953	3030	3	scRNA-Seq
Stric.Inv	5675	1626	6	scRNA-Seq
Stric.N.Inv	6405	2045	3	scRNA-Seq
Healthy	3284	1457	6	scRNA-Seq

Supplementary Table 3. Summary of cell/spot counts per sample obtained via spatial and scRNAseq assays.

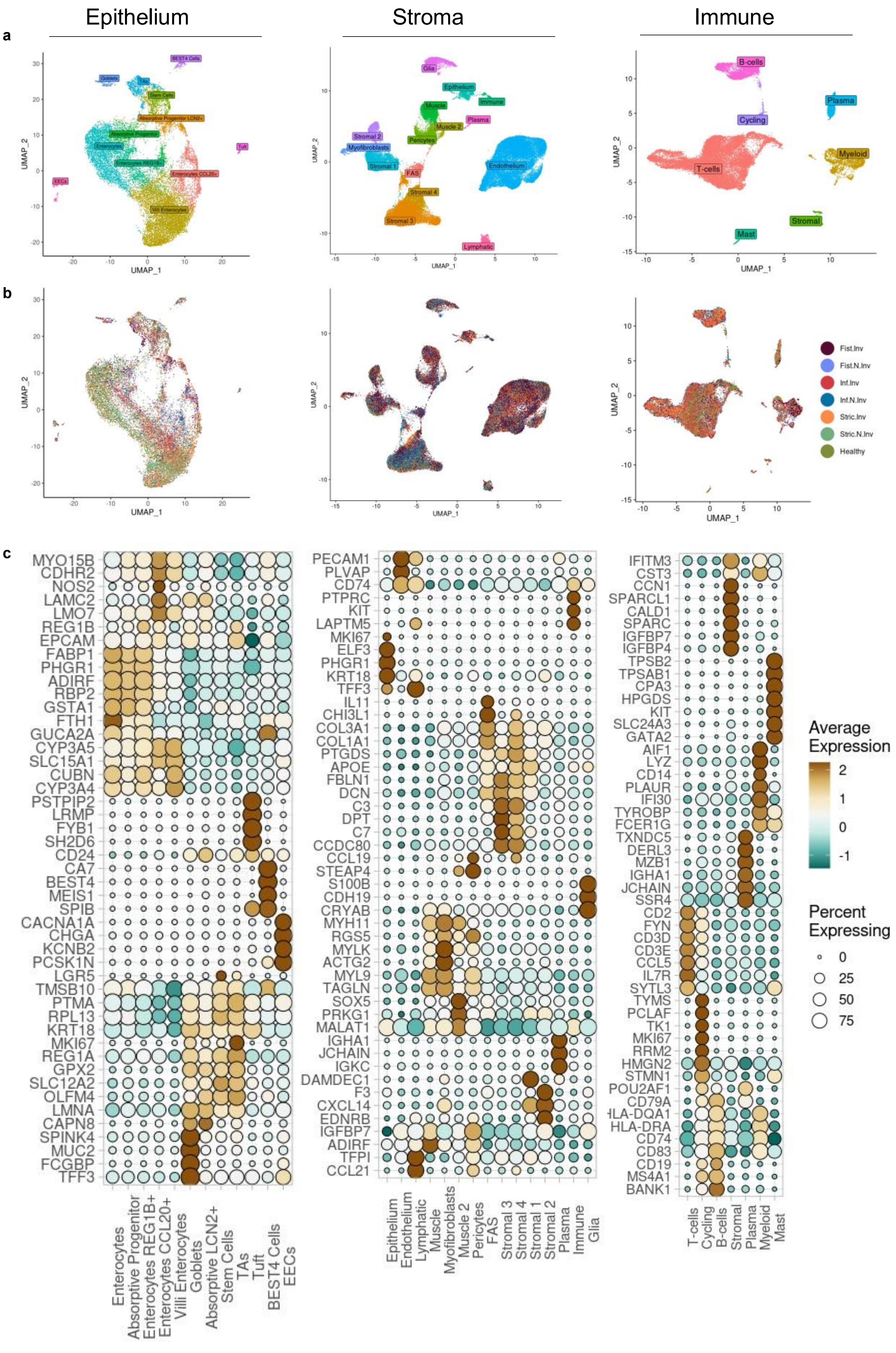

Supplementary Figure 1.a. UMAP embedding visualising all epithelial, stromal and immune cell clusters detected in scRNAseq cohort. **b.** As in a, except sample type overlay is shown. **c.** Dotplot visualising top cluster markers for epithelial, stromal and immune cells identified in scRNAseq cohort.

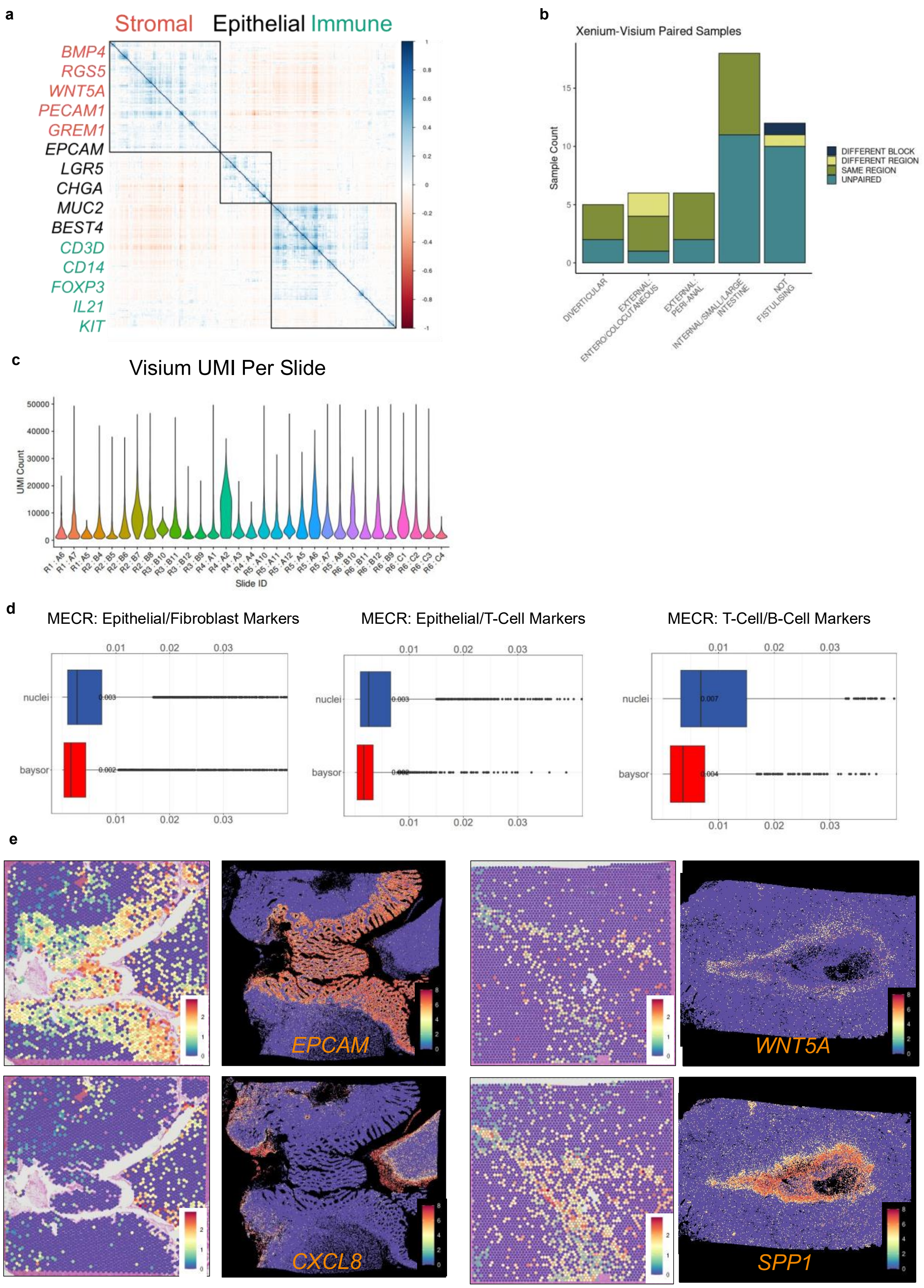

Supplementary Figure 2.a. Gene-gene correlation plot visualising the correlation structure of custom pan-gut 480 gene target panel designed for Xenium ST. Selected stromal, immune and epithelial gene markers are highlighted. **b.** Barplot visualising overlap between paired Xenium and Visium ST samples per sample type group. **c.** Per spot UMI count distribution in Visium ST cohort across all tissue sections profiled. **d.** Mutually exclusive marker co-expression ratio score comparing nuclei-based segmentation of Xenium *in situ* data with transcript-density based segmentation with baysor. **e.** Selected gene expression spatial overlays comparing measures from paired Visium and Xenium ST sections, with a representative diverticular disease fistula (left) and non-epithelialised CD fistula section (right) shown. Xenium ST section of the latter is rotated at ~45 angle compared to the Visium ST section.

Xenium ST 480-plex Cohort Cell Type Clusters

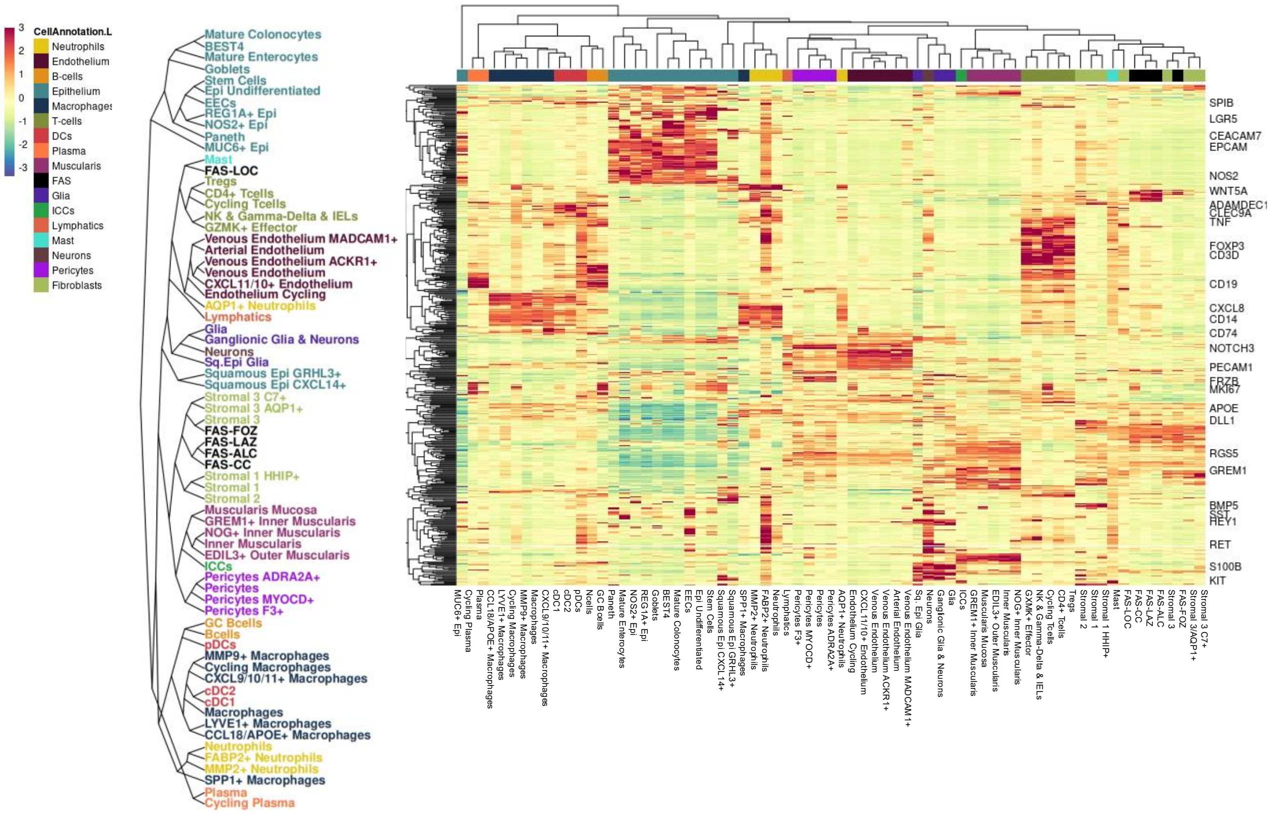

Xenium ST 5100-plex Cohort Cell Type Clusters

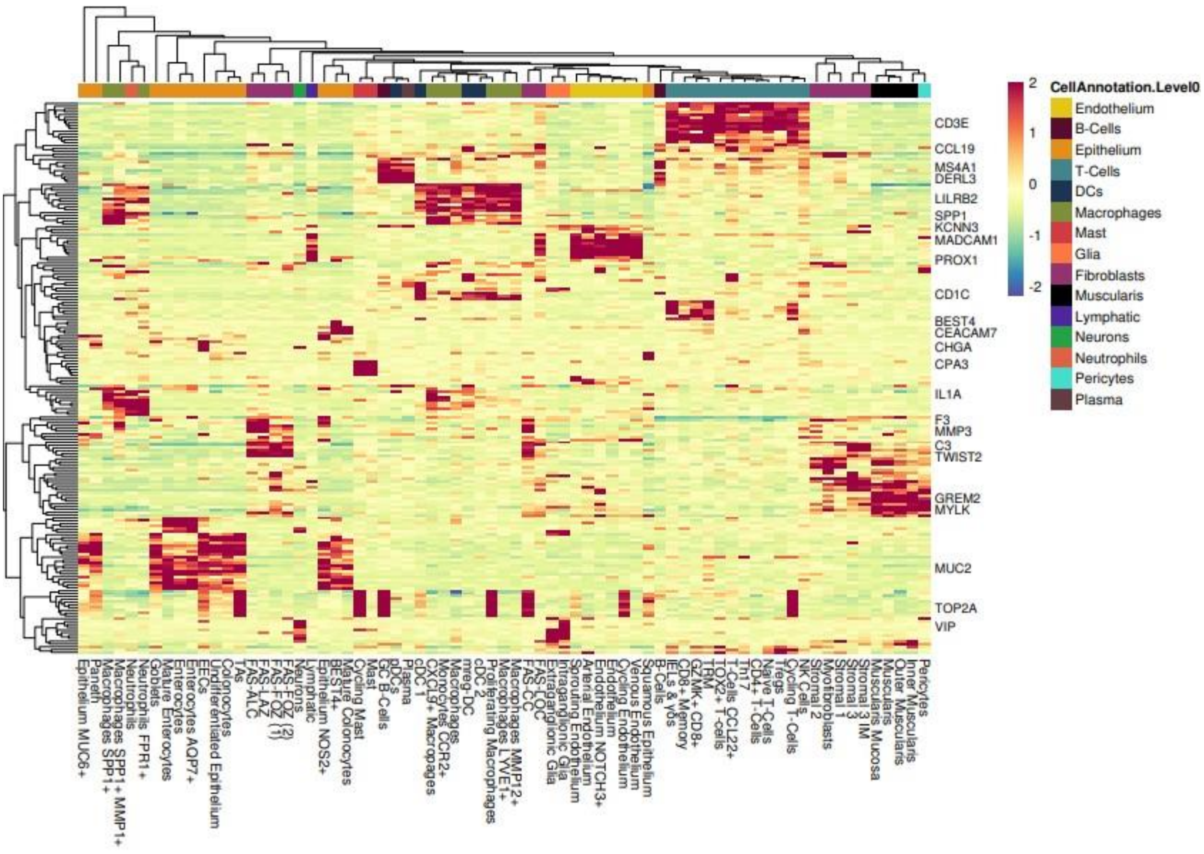

Supplementary Figure 3. Heatmaps visualising cell type cluster specific gene expression in Xenium ST 480-plex (top) and 5100-plex (bottom) cohorts.

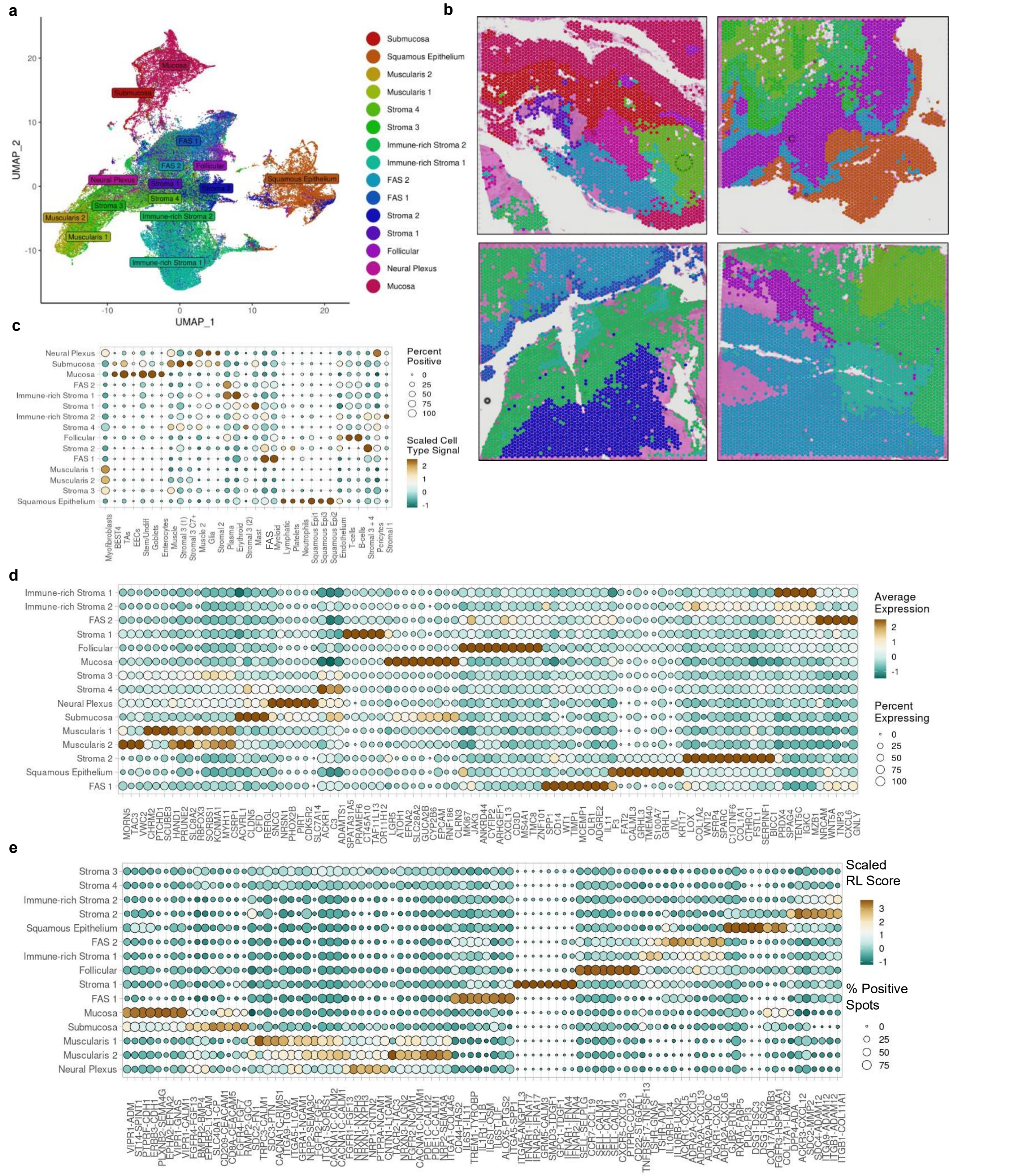

Supplementary Figure 4.a. UMAP embedding visualising fine Visium ST clusters detected in the full cohort. **b.** Spatial overlay of Visium ST integrated regions over selected epithelialised (top row) and non-epithelialised (bottom row) fistula slides. **c.** Dotplot visualising scRNA-Seq cell type deconvolution signal across each spatial region/cluster identified in Visium ST cohort. **d.** Dotplot visualisating top cluster marker genes for each spatial region/cluster identified in Visium ST cohort. **e.** Dotplot visualising top spot cluster specific receptor-ligand nteractions detected in Visium ST cohort.

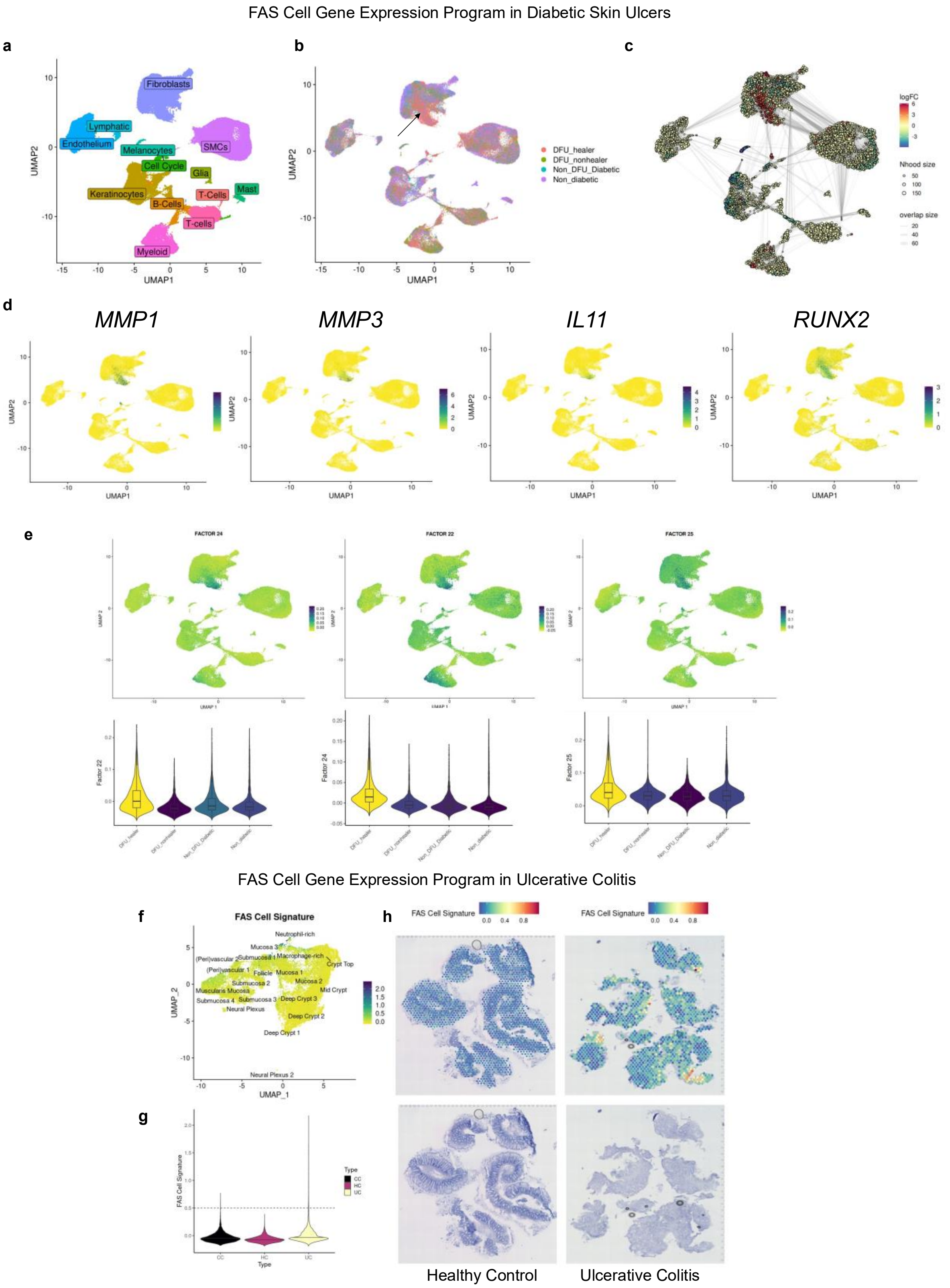

Supplementary Figure 5. a. UMAP visualisation of cell clusters from scRNA-Seq data from Theocharidis *et al*, 2022. **b.** UMAP overlay visualising sample/disease state for each cell. Arrow indicates an area of fibroblast embedding enriched in healing ulcers. **c**. Differential abundance analysis using miloR visualising local embedding enrichment of fibroblasts between diabetic foot ulcer (DFU) healers and non-healers. **d**. Selected FAS cell markers showing specific expression in DFU-enriched fibroblast cells. **e.** UMAP overlays visualising key selected FAS associated cNMF factors identified in intestinal fibroblast meta analysis scored in cells from Theocharidis *et al,* 2022 study. Corresponding violin plots comparing experimental groups in fibroblast cells only from Theocharidis *et al,* 2022 study are shown beneath each UMAP. **f.** UMAP visualisation of Visium ST data from a colitis study from Gupta *et al*, 2024 showing FAS cell type signature score in all spots in the study. Labels indicate broad annotated spatial regions. **g.** Violin plot comparing FAS cell signature score between healthy controls (HC), ulcerative colitis (UC) and checkpoint-inhibitor induced colitis (CC) patients. **h.** FAS cell signature score overlay over a representative HC and UC tissue section from the Gupta *et al* 2024 study.

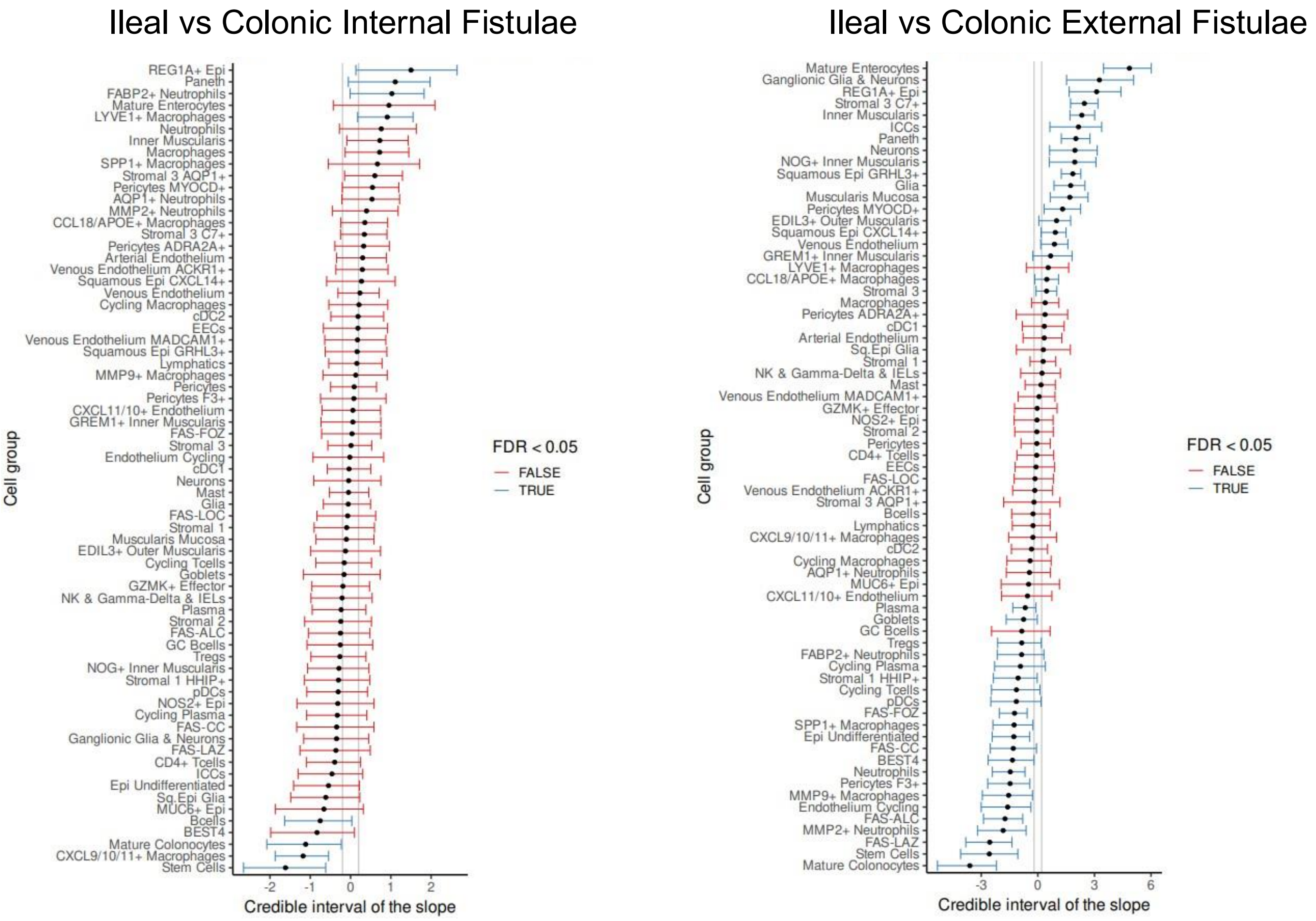

Supplementary Figure 6. Differential abundance plot comparing location differences between ileal and colonic internal (left) and external (right)fistulae in Xenium ST 480-plex cohort (n=53 total samples). Bands represent the 95% Bayesian credible interval of the slope (logit fold change in cluster proportion per unit change in the covariate), indicating the range of effect sizes compatible with the data, given the model.

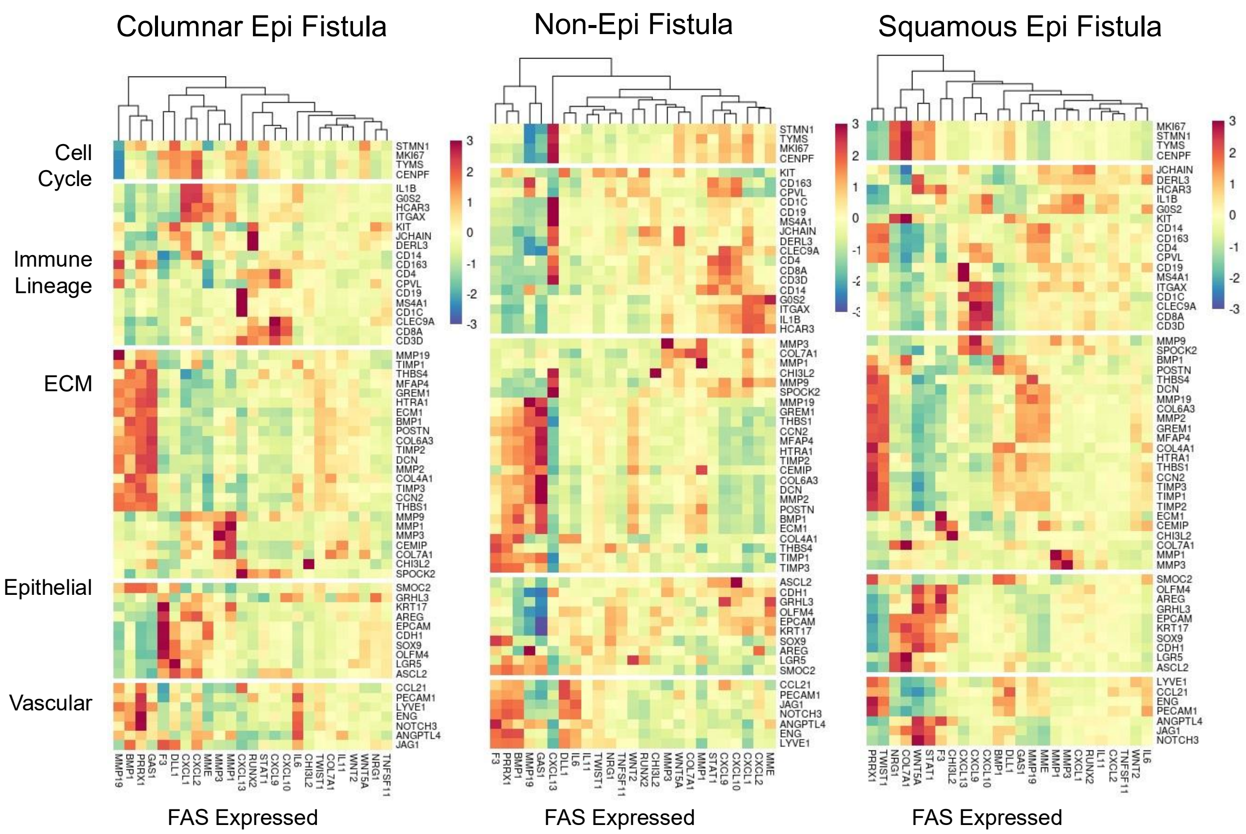

Supplementary Figure 7. Heatmaps comparing gene wise bivariate spatial correlation (scaled, row-wise) of FAS cell marker genes and key selected marker genes, visualising spatial transcript co-localisation irrespective of cell segmentation and assignment. For example, *CXCL13* shows strong spatial co-localisation with B-cell and T-cell lineage markers; *WNT5A* expression has high spatial correlation with cell cycle marker genes e.g. *MKI67*.

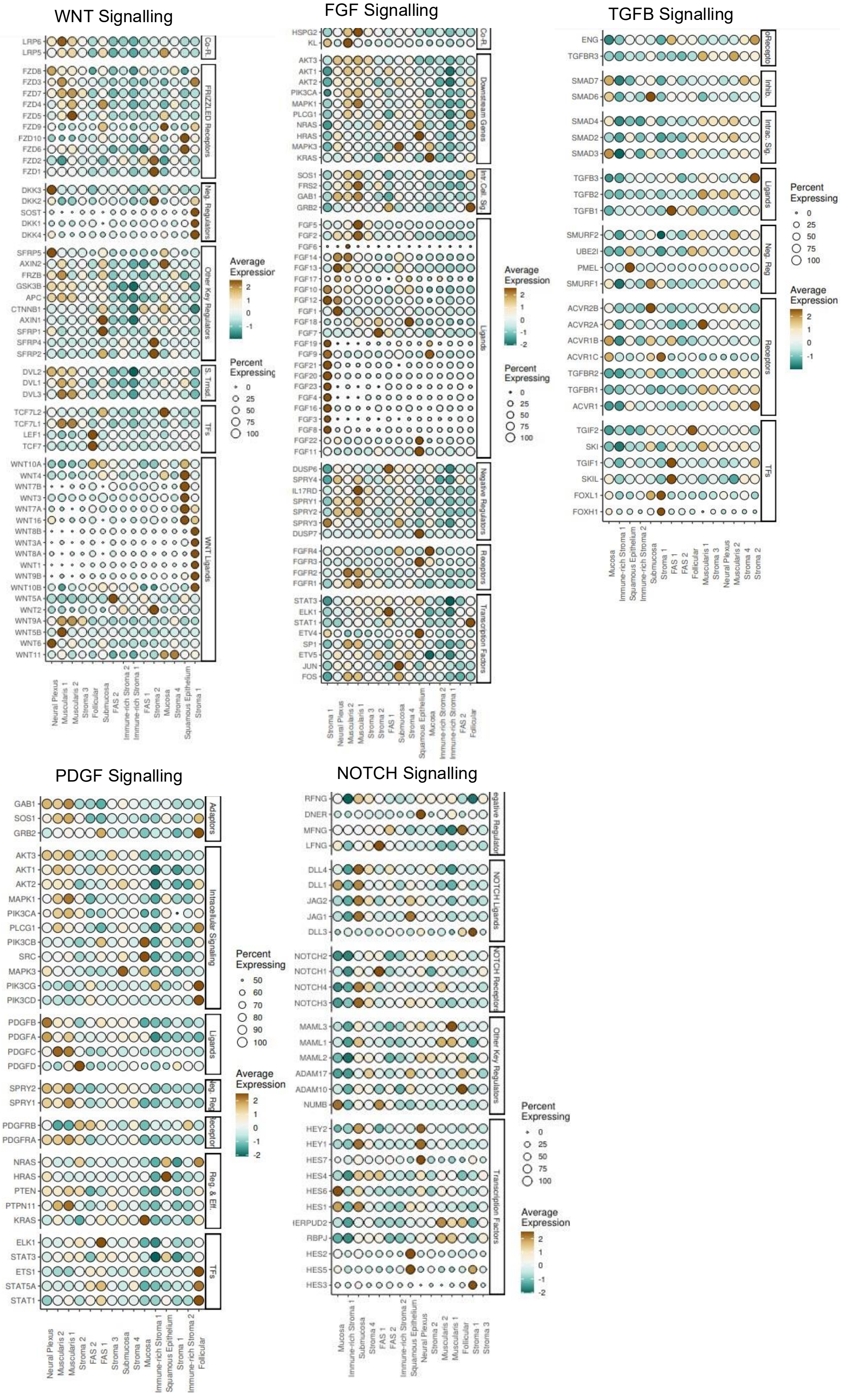

Supplementary Figure 8. Dotplots visualising expression of key WNT, FGF, TGFB, PDGF and NOTCH signalling pathway genes in Visium ST data per integrated spatial region.

a b

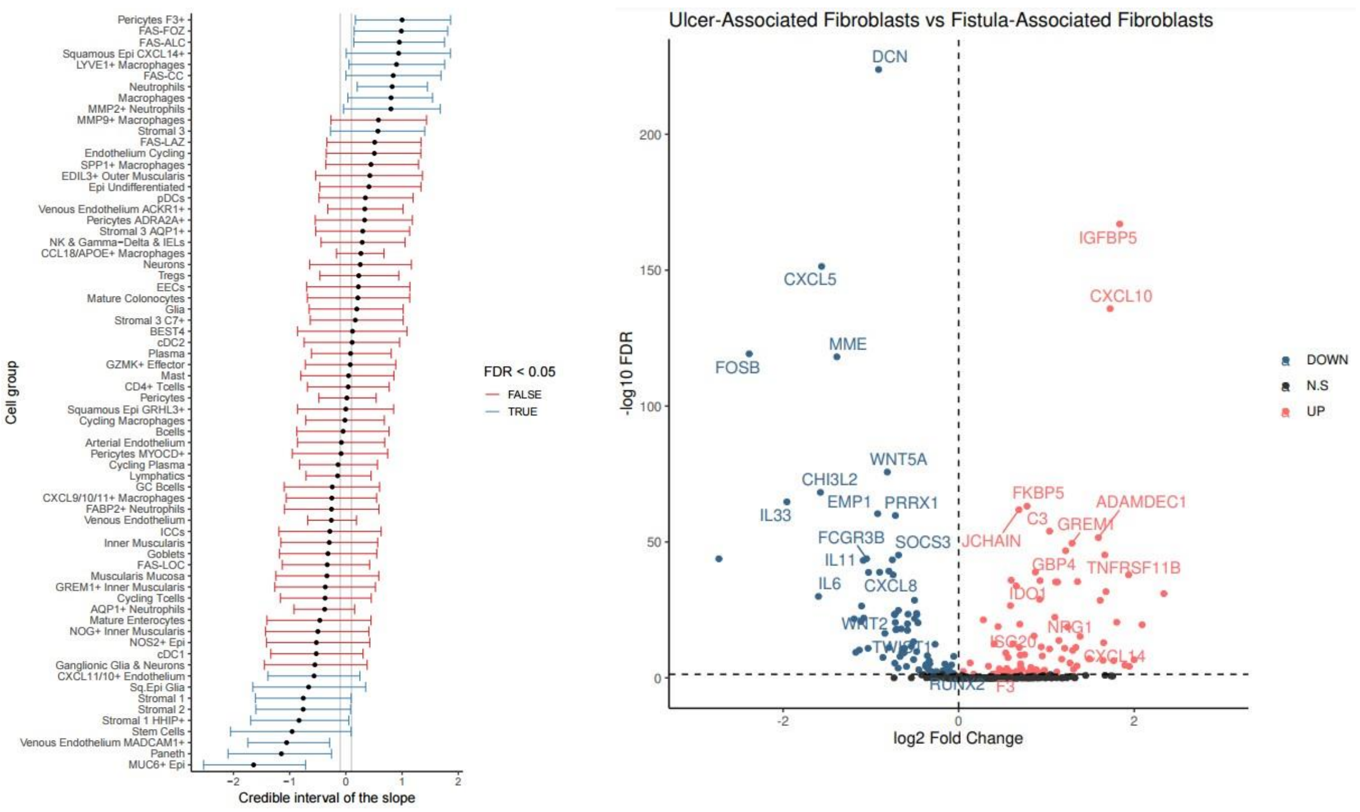

Supplementary Figure 9. a. Differential cell type abundance analysis comparing CD fistulae vs CD ulcer regions in Xenium ST 480-plex cohort (n=53 total samples). **b.** Volcano plot comparing differentially expressed genes between FAS cell subtypes identified in CD ulcers vs fistulae profiled using 480-plex Xenium ST cohort (n=53 total samples).

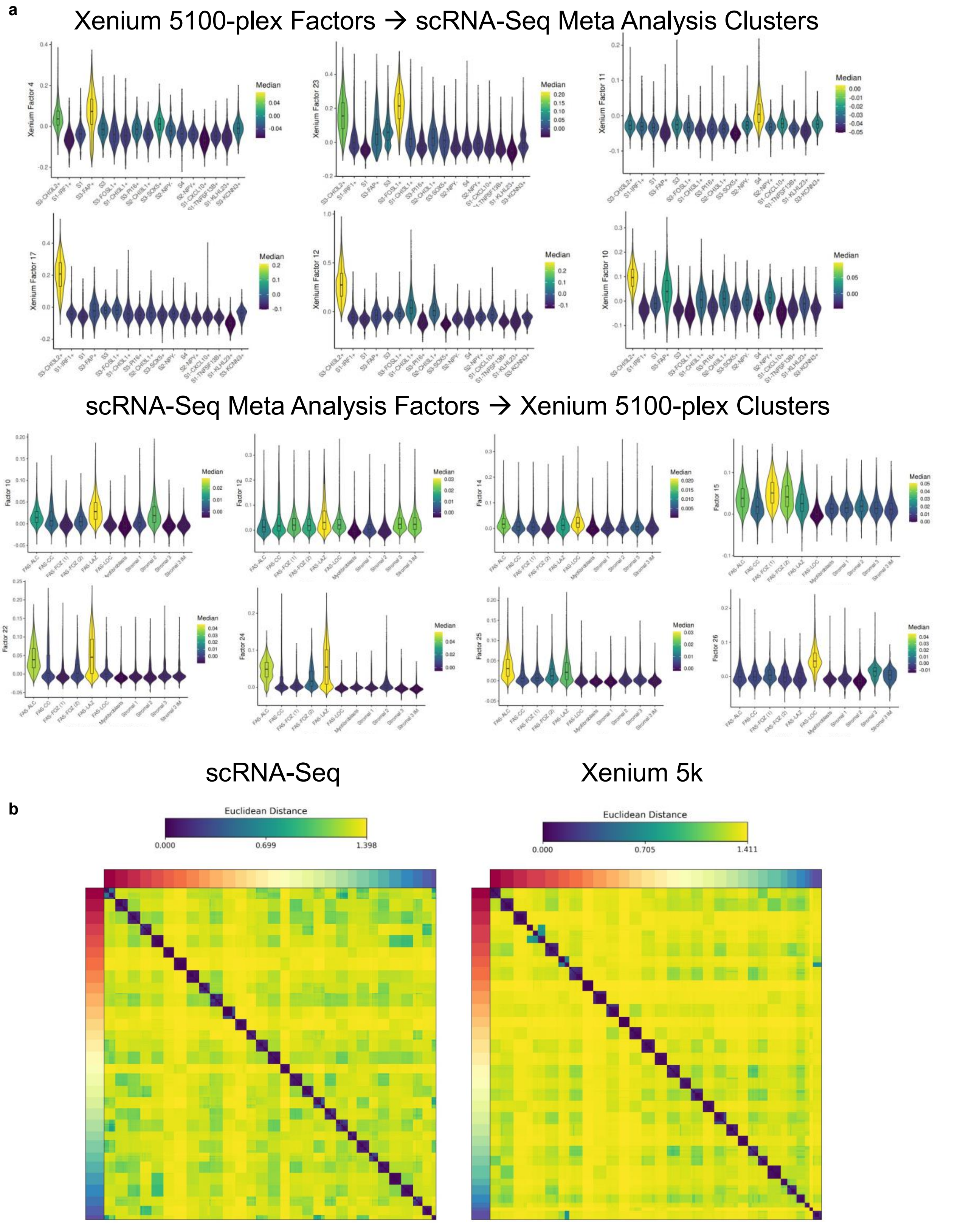

Supplementary Figure 10. **a.** Violin plots visualising selected disease-enriched cNMF factors detected in fibroblast cells from 5100-plex Xenium ST dataset and scored/applied to cells from scRNA-Seq fibroblast meta analysis, visualising factor correspondence to meta analysis clusters. The bottom panels show the reverse analysis, with selected cNMF factors detected from fibroblast scRNA-Seq meta analysis applied to 5100-plex Xenium ST dataset.**b**. cNMF factor pairwise distance plots from scRNA-Seq meta analysis and Xenium 5100-plex dataset.

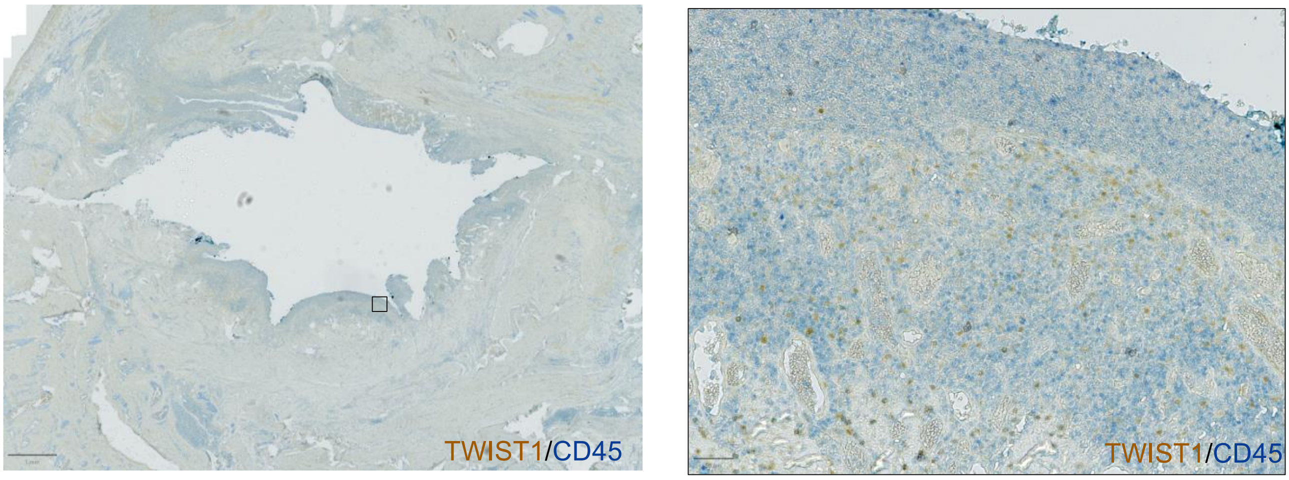

Supplementary Figure 11. A representative IHC image of a non-epithelialised CD fistula section co-stained for TWIST1 and CD45. The black box in section of the left represents a field of view corresponding to fistula edge shown on the right. Scale bars: 1mm and (ii) 50 μm respectively.

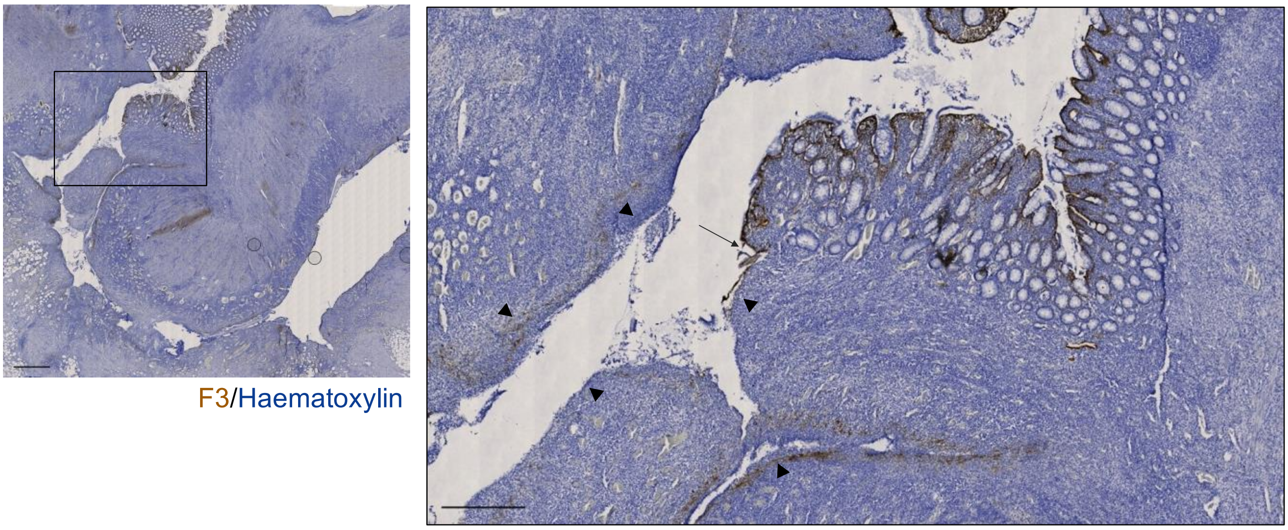

Supplementary Figure 12. Representative IHC image of a colonic CD fistula stained for the stromal marker F3. The black line highlights the selected field of view demonstrating epithelial loss (black arrow) along the fistula tract (arrowheads). Scale bars: (i) 1 mm, (ii) 500 μm respectively.

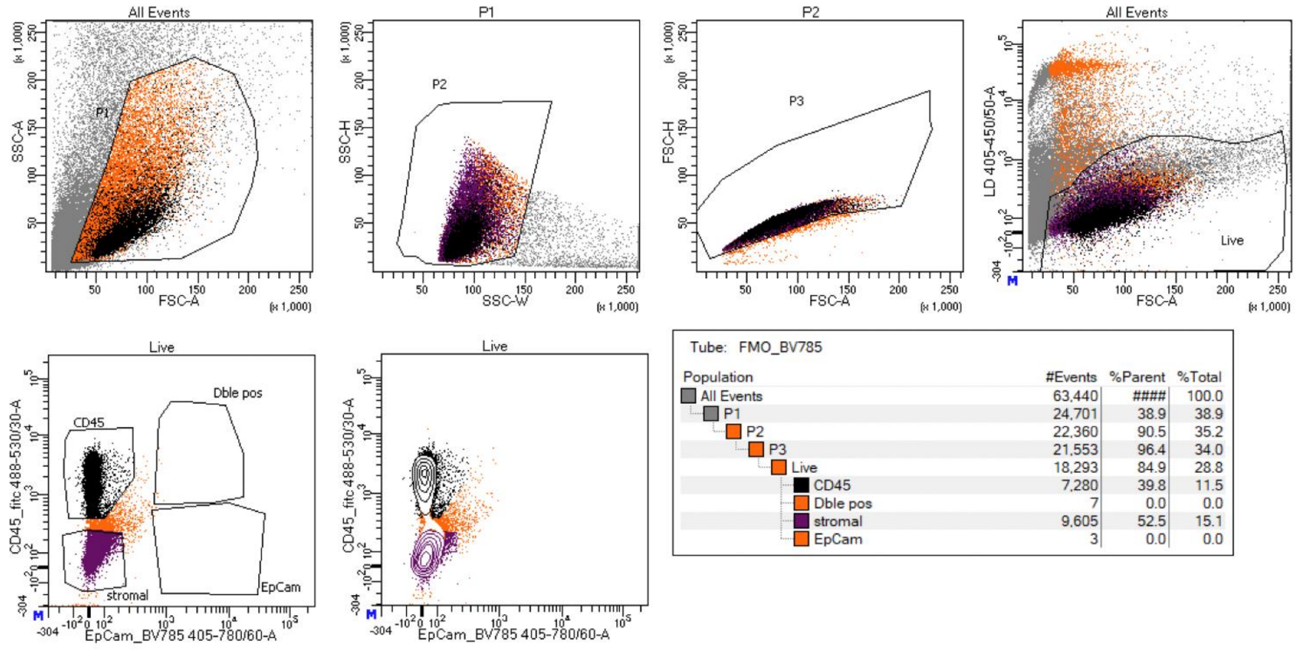

Tube: FMO_BV785

Population	#Events	%Parent	%Total
All Events	63,440	####	100.0
P1	24,701	38.9	38.9
P2	22,360	90.5	35.2
P3	21,553	96.4	34.0
Live	18,293	84.9	28.8
CD45	7,280	39.8	11.5
Dble pos	7	0.0	0.0
stromal	9,605	52.5	15.1
EpCam	3	0.0	0.0

Supplementary Figure 13. FACS gating strategy for scRNAseq analysis cell sorting.
