# Supplementary material for: Spatial fibroblast niches define Crohn’s fistulae
Source: Nature. 2025 Nov 12;649(8097):703–12. doi: 10.1038/s41586-025-09744-y (PMC12804086; doi:10.1038/s41586-025-09744-y)
Supplement: Supplementary file 2 — Reporting Summary [file 41586_2025_9744_MOESM2_ESM.pdf]

nature portfolio

Corresponding author(s): Agne Antanaviciute & Alison Simmons

Last updated by author(s): Sep 28, 2025

Reporting Summary

Nature Portfolio wishes to improve the reproducibility of the work that we publish. This form provides structure for consistency and transparency in reporting. For further information on Nature Portfolio policies, see our Editorial Policies and the Editorial Policy Checklist.

Statistics

For all statistical analyses, confirm that the following items are present in the figure legend, table legend, main text, or Methods section.

n/a	Confirmed	
☐	☒	The exact sample size (*n*) for each experimental group/condition, given as a discrete number and unit of measurement
☐	☒	A statement on whether measurements were taken from distinct samples or whether the same sample was measured repeatedly
☐	☒	The statistical test(s) used AND whether they are one- or two-sided<br>*Only common tests should be described solely by name; describe more complex techniques in the Methods section.*
☐	☒	A description of all covariates tested
☐	☒	A description of any assumptions or corrections, such as tests of normality and adjustment for multiple comparisons
☐	☒	A full description of the statistical parameters including central tendency (e.g. means) or other basic estimates (e.g. regression coefficient) AND variation (e.g. standard deviation) or associated estimates of uncertainty (e.g. confidence intervals)
☐	☒	For null hypothesis testing, the test statistic (e.g. *F*, *t*, *r*) with confidence intervals, effect sizes, degrees of freedom and *P* value noted<br>*Give P values as exact values whenever suitable.*
☒	☐	For Bayesian analysis, information on the choice of priors and Markov chain Monte Carlo settings
☒	☐	For hierarchical and complex designs, identification of the appropriate level for tests and full reporting of outcomes
☐	☒	Estimates of effect sizes (e.g. Cohen's *d*, Pearson's *r*), indicating how they were calculated

Our web collection on statistics for biologists contains articles on many of the points above.

Software and code

Policy information about availability of computer code

Data collection	BD FACS Diva Software v9.0 BD Biosciences<br>Zeiss Axioscanner (Software: Zen Blue Edition Version 3.3.89.0000)
Data analysis	FACS: FlowJo v10.8.1 FlowJo.com<br>PCR, Image data: Graphpad Prism v9.5.1 (528) www.graphpad.com<br>Qupath software (version 0.5.0)<br><br>Illumina bcl2fastq (version 2.20.0.422)<br>FastQC software (version 0.11.9)<br>10x Genomics Cellranger software (version 7.1.0)<br>10X Genomics Spaceranger software (version 2.1.0)<br>cNMF pipeline (version 1.7.0)<br>Cutadapt software (version 4.4)<br>STAR aligner software (version 2.7.11a)<br>subread featureCounts software (version 2.0.6)<br>multiQC software (version 1.14)<br>Picard tools (version 3.2.0)<br>pySCENIC pipeline (version 0.12.2b0)<br>Baysor algorithm (0.6.2)

R package DropletUtils (version 1.24)
R package scCustomize (version 2.1.2)
R package Seurat (version 5.1.0)
R package Harmony (version 1.2.1)
R package clustree (version 0.5.1)
R package DESeq2 (version 1.44.0)
R package miloR (version 2.0.0)
R package sccomp (version 1.9.5)
R package ggpraph (version 2.2.1)
R package clusterProfiler (version 4.12.6)
R package DBI (version1.66.0)
R package CARD (version 1.1)
R package scCustomize (version 2.1.2)
R package MAST (version 1.33.0)
R package AUCell(version 1.30.1)

Python (version 3.9)
Python package skimage (version 0.20.0)
Python package numpy (version 1.26.4)
Python package pandas (version 2.1.1)
Python package scikit-learn (version 1.5.2)
Python package matplotlib (version 3.7.1)
Python package seaborn (version 0.13.2)

Code used in the data analysis has been deposited on github https://github.com/agneantanaviciute/cdfistulaspatialtranscriptomics and https://github.com/ChloeHJ/spamsc.

For manuscripts utilizing custom algorithms or software that are central to the research but not yet described in published literature, software must be made available to editors and reviewers. We strongly encourage code deposition in a community repository (e.g. GitHub). See the Nature Portfolio guidelines for submitting code & software for further information.

Data

Policy information about availability of data

All manuscripts must include a data availability statement. This statement should provide the following information, where applicable:

- Accession codes, unique identifiers, or web links for publicly available datasets
- A description of any restrictions on data availability
- For clinical datasets or third party data, please ensure that the statement adheres to our policy

All raw sequencing data has been deposited on GEO (series GSE284232), accessions GSE283945(Visium ST), GSE305631(bulk RNA-Seq) and GSE284230 (scRNA-Seq). All processed scRNA-Seq, Visium ST and Xenium ST Seurat data RDS object files, image data and scRNAseq and spatial transcriptomics data tables have been deposited as Mendeley Data: doi: 10.17632/tn972brm9s.1 (https://data.mendeley.com/datasets/tn972brm9s/1), doi: 10.17632/mxy6p6wfmy.1 (https://data.mendeley.com/preview/mxy6p6wfmy) and doi: 10.17632/64fkdfcpzb.1 (https://data.mendeley.com/datasets/64fkdfcpzb/1).

Data for scRNA-Seq meta analysis was obtained from GEO: GSE189185, GSE114374, GSE134809, GSE260842, GSE260833, GSE266546; ArrayExpress: E-MTAB-8901; Broad Single Cell Portal SCP259, SCP1884; and Zenodo https://doi.org/10.5281/zenodo.13768607.

Additional scRNA-Seq reference data for cell type deconvolution was downloaded from GEO: GSE201153 and GSE163668.

A browsable spatial data portal web app is available at https://simmonslab.shinyapps.io/cd-fistula-data-portal/

Research involving human participants, their data, or biological material

Policy information about studies with human participants or human data. See also policy information about sex, gender (identity/presentation), and sexual orientation and race, ethnicity and racism.

Reporting on sex and gender	Samples were balanced across biological sex (as reported clinically) as part of the study design, where possible - scRNAseq (14 F, 19M), Visium ST (15 F, 19M), Xenium ST (28 F, 25M), biomark (36F, 48M).
Reporting on race, ethnicity, or other socially relevant groupings	Not applicable, no data on race and ethnicity was collected.
Population characteristics	Human ileal and colonic tissue was collected from consenting adult. A detailed sample overview with sex, age and other clinical characteristics is provided in Supplementary Table 1.
Recruitment	Patients were recruited to studies in a randomized fashion depending on their presentation to hospital as part of their routine clinical care. Recruitment and collection was by multiple investigators, at multiple sites, over a period of many years, with no obvious biases that could be identified.
Ethics oversight	All samples were collected with informed consent as per the principles of Helsinki under the aegis of studies and procedures

Ethics oversight	approved by different NHS Research Ethics Committees (RECs). The relevant registered approvals are as follows:<br>REC reference(s):<br>TIP Study: 18/WM/0237<br>GI Biobank: 16/YH/0247<br>IBD Biobank: 09/H1204/30<br>FFPE Tissue samples (OCHRe): 19/SC/0173<br>FFPE Tissue Samples (Bayreuth): Friedrich Alexander University, Bayreuth, Germany, Ethics number: 23-131 bp

Note that full information on the approval of the study protocol must also be provided in the manuscript.

Field-specific reporting

Please select the one below that is the best fit for your research. If you are not sure, read the appropriate sections before making your selection.

☒ Life sciences ☐ Behavioural & social sciences ☐ Ecological, evolutionary & environmental sciences

For a reference copy of the document with all sections, see nature.com/documents/nr-reporting-summary-flat.pdf

Life sciences study design

All studies must disclose on these points even when the disclosure is negative.

Sample size	No formal sample size calculations were performed for spatial and single cell analyses. Instead, we analyzed all available patient samples meeting inclusion criteria resulting in a large cohort. This sample size exceeds many prior studies of intestinal tissue using these methods and provides sufficient statistical power to detect robust cell state differences and spatial patterns. For bulkRNA-Seq, we used n=5 biological replicates per group, which is in line with accepted practice for bulk RNA-Seq discovery studies, providing sufficient power to detect consistent DEGs while balancing feasibility and resources.
Data exclusions	No data were excluded from analysis.
Replication	All experiments were performed in triplicates or more in order to ensure reliability and reproducibility of results. Exact quantification for each replicate is described in methods and figure legends.
Randomization	Patients attending hospital for their planned care were consented in a random fashion to collect research samples, and classified into groups - healthy or disease - based on histopathological analysis blinded to the research objective. For TWIST1 and OSR2 overexpression experiments, bulk RNA seq, qPCR and imaging, a paired design was used rather than randomised, where samples from the same donor were used across all experimental groups. All statistical analyses of these data were performed in a paired fashion, accounting for donor variability as a covariate in the statistical testing.
Blinding	Blinding was not necessary as experimental read-outs were automated (FACS, qPCR, scRNA-Seq, spatial transcriptomics, bulkRNAseq). Histopathology annotations were blinded.

Reporting for specific materials, systems and methods

We require information from authors about some types of materials, experimental systems and methods used in many studies. Here, indicate whether each material, system or method listed is relevant to your study. If you are not sure if a list item applies to your research, read the appropriate section before selecting a response.

Materials & experimental systems

n/a	Involved in the study	
☐	☒	Antibodies
☐	☒	Eukaryotic cell lines
☒	☐	Palaeontology and archaeology
☒	☐	Animals and other organisms
☐	☒	Clinical data
☒	☐	Dual use research of concern
☒	☐	Plants

Methods

n/a	Involved in the study	
☒	☐	ChIP-seq
☐	☒	Flow cytometry
☒	☐	MRI-based neuroimaging

Antibodies

Antibodies used	Immunohistochemistry:<br>TWIST1 E5G9Y Rabbit IgG RRID:AB_3064916 (pH 6, 1:800)<br>Anti-F3 AMAb91235 Mouse IgG RRID:AB_2665858 (pH 6, 1:250)<br>CD45 30-F11 Rat IgG2b, kappa RRID:AB_467251 (pH 6, 1:50)<br><br>Immunoflourescence:<br>Neutrophil Elastase NIMP-R14 Rat IgG2b 1:100 RRID:AB_303154

F3 (C0142) CL3905 Mouse IgG1 1:100 RRID:AB_2665858
WNT5A MAB645 Rat IgG2A 1:200 RRID:AB_10571221
Cleaved Caspase 3, 5A1E, Rabbit IgG 1:100 RRID:AB_2928048
CD45, RM1007, Rabbit, IgG, 1:100 RRID:AB_3678516
Periostin, EPR20806, Rabbit, IgG, 10ug/ml RRID:AB_2924310
NaKATPase, Rabbit, IgG, 10ug/ml RRID:AB_3451994
Collagen 7, Abcam: ab198899, Rabbit, IgG, 10ug/ml
Vimentin, EPR3776, Rabbit, IgG, 5 ug/ml RRID:AB_2909595
PRRX2, HPA026808, Rabbit, IgG, 1:100 RRID:AB_10603228
OSR2,HPA052425, Rabbit, IgG, 1:100 RRID:AB_2681826
TWIST1, E5G9Y, Rabbit, IgG, 1:100 RRID:AB_3064916
RUNX2, EPR14334, Rabbit, IgG, 1:100 RRID:AB_2889254
KI67, SP6 Rabbit IgG 1:100 RRID: AB_302459
Pan-Cytokeratin PCK-26 Mouse IgG1 1:100 RRID: AB_305450
Pan-Cadherin EPR1792Y IgG 10 ug/ml RRID: AB_3106921

FACS:
Brilliant Violet 785'" anti-human CD326 (EpCAM) Antibody, 9C4 clone BioLegend Cat# 324237 RRID:AB_2632936 (1:50)
FITC anti-human CD45 Antibody, H130 clone BioLegend Cat# 304005 RRID:AB_314393 (1:100)
Human Trustain FcX (Fc Block) Biolegend Cat# 422302, RRID:AB_2818986 (TruStain FcX was added at 5 µl per 1×10^6 cells in 100 µl staining volume)
DAPI (4'6-diamidino-2- phenylindole) Solution, BD Pharminogen, Cat#564907 (1:1000)

CITE-seq:
TotalSeqC anti-human CD4 Antibody BioLegend Cat# 344651 RRID:AB_2800921 (1:100)
TotalSeqC anti-human CD8 Antibody BioLegend Cat# 344753 RRID:AB_2800922(1:100)
TotalSeqC anti-human CD56 (NCAM) Antibody BioLegend Cat# 362559 RRID:AB_2801002 (1:100)
TotalSeqC anti-human CD3 Antibody BioLegend Cat# 344849 RRID:AB_2814272 (1:100)
TotalSeqC anti-human CD45RA Antibody BioLegend Cat# 304163 RRID:AB_2800764 (1:100)
TotalSeqC anti-human CD45RO Antibody BioLegend Cat# 304259 RRID:AB_2800766 (1:100)
TotalSeqC anti-human CD279 (PD-1) Antibody BioLegend Cat# 329963 RRID:AB_2800862 (1:100)
TotalSeqC anti-human CD103 (Integrin aE) Antibody BioLegend Cat# 350233 RRID:AB_2800933 (1:100)
TotalSeqC anti-human Hashtag 1 Antibody BioLegend Cat# 394661 RRID:AB_2801031 (0.75µl per $1x10^6$ cells per 100µl staining volume)
TotalSeqC anti-human Hashtag 2 Antibody BioLegend Cat# 394663 RRID:AB_2801032 (0.75µl per $1x10^6$ cells per 100µl staining volume)
TotalSeqC anti-human Hashtag 3 Antibody BioLegend Cat# 394665 RRID:AB_2801033 (0.75µl per $1x10^6$ cells per 100µl staining volume)
TotalSeqC anti-human Hashtag 4 Antibody BioLegend Cat# 394667 RRID:AB_2801034 (0.75µl per $1x10^6$ cells per 100µl staining volume)
TotalSeqC anti-human Hashtag 5 Antibody BioLegend Cat# 394669 RRID:AB_2801035 (0.75µl per $1x10^6$ cells per 100µl staining volume)
TotalSeqC anti-human Hashtag 6 Antibody BioLegend Cat# 394671 RRID:AB_2820042 (0.75µl per $1x10^6$ cells per 100µl staining volume)
TotalSeqC anti-human Hashtag 7 Antibody BioLegend Cat# 394673 RRID:AB_2820043 (0.75µl per $1x10^6$ cells per 100µl staining volume)

Validation

All commercially available antibodies are commonly used clones with extensive validation, the relevant literature being detailed on the supplier website. A validation statement from BioLegend: Antibody validation is a critical step in the journey towards obtaining consistent reproducibility in science. To ensure they are both specific and sensitive, we validate our antibodies through a variety of methods including:

- Testing on multiple cell and tissue types with a variety of known expression levels.
- Validation in multiple applications as a cross-check for specificity and to provide additional clarity for researchers.
- Comparison to existing antibody clones.
- Using cell treatments to modulate target expression, such as phosphatase treatment to ensure phospho-antibody specificity.
(www.biolegend.com/en-gb/bio-bits/highly-specific-validated-antibodies)

Where relevant, we further confirmed specificity by assessing staining in the expected cell types and tissue structures, and by comparing with transcript expression profiles in our datasets. All antibodies were additionally tested on full-thickness intestinal sections, both inflamed and non-inflamed, with staining conditions optimised through serial dilutions and pH adjustments around the supplier’s recommended conditions. Specificity was further supported by validating the distribution of stromal markers, for example by confirming absence of staining in epithelial cells.

Eukaryotic cell lines

Policy information about cell lines and Sex and Gender in Research

Cell line source(s)

HEK293 cells were obtained from ATCC (#CRL-1573).

Authentication

HEK293 cells were authenticated by the supplier (ATCC #CRL-1573).

Mycoplasma contamination | HEK293 cells were tested mycoplasma-free.

Commonly misidentified lines (See ICLAC register) | *Name any commonly misidentified cell lines used in the study and provide a rationale for their use.*

Clinical data

Policy information about clinical studies

All manuscripts should comply with the ICMJE guidelines for publication of clinical research and a completed CONSORT checklist must be included with all submissions.

Clinical trial registration	NA
Study protocol	NA
Data collection	NA
Outcomes	NA

Plants

Seed stocks	NA
Novel plant genotypes	NA
Authentication	NA

Flow Cytometry

Plots

Confirm that:

- [x] The axis labels state the marker and fluorochrome used (e.g. CD4-FITC).
- [x] The axis scales are clearly visible. Include numbers along axes only for bottom left plot of group (a 'group' is an analysis of identical markers).
- [x] All plots are contour plots with outliers or pseudocolor plots.
- [x] A numerical value for number of cells or percentage (with statistics) is provided.

Methodology

Sample preparation	For flow cytometry sorting samples were processed identically to those described in methods section. Following this they were then washed with staining buffer (PBS with 2% BSA and 0.01%Tween) and were then stained with appropriate antibodies (Flow cytometry/CITE-seq) at pre-optimized concentrations for 30 minutes. Samples were then washed and sorted directly as described. Prior to running samples compensations were calculated with an unstained cellular control and compensation beads (BD).
Instrument	BD FACS Aria Illu, BD FACS Fusion (Sorting)
Software	BD FACS Diva Software v9.0, with Quantification performed using FlowJo v10.8.1
Cell population abundance	CD45+, CD45-EPCAM+ or CD45-EPCAM- abundance: 10-90% of parent population, depending on degree of inflammation and tissue source material.<br>As all sorted cells were subject to single cell analysis, we were able to validate transcriptionally that there was no unexpected cellular contamination.
Gating strategy	CD45+Sort: FSC-SSC-> Singlets -> Live Dead -> EPCAM-CD45+<br>CD45-EPCAM- Sort: FSC-SSC-> Singlets -> Live Dead -> EPCAM-CD45-

- [x] Tick this box to confirm that a figure exemplifying the gating strategy is provided in the Supplementary Information.
