# Supplementary material for: Spatial fibroblast niches define Crohn’s fistulae
Source: Nature. 2025 Nov 12;649(8097):703–12. doi: 10.1038/s41586-025-09744-y (PMC12804086; doi:10.1038/s41586-025-09744-y)

Supplementary Data: Visium and Xenium Cohort - Annotations

KEY:

VISIUM FOV

XENIUM FOV

FISTULA

BAY 113385_21

COLO-
CUTANEOUS
CD FISTULA

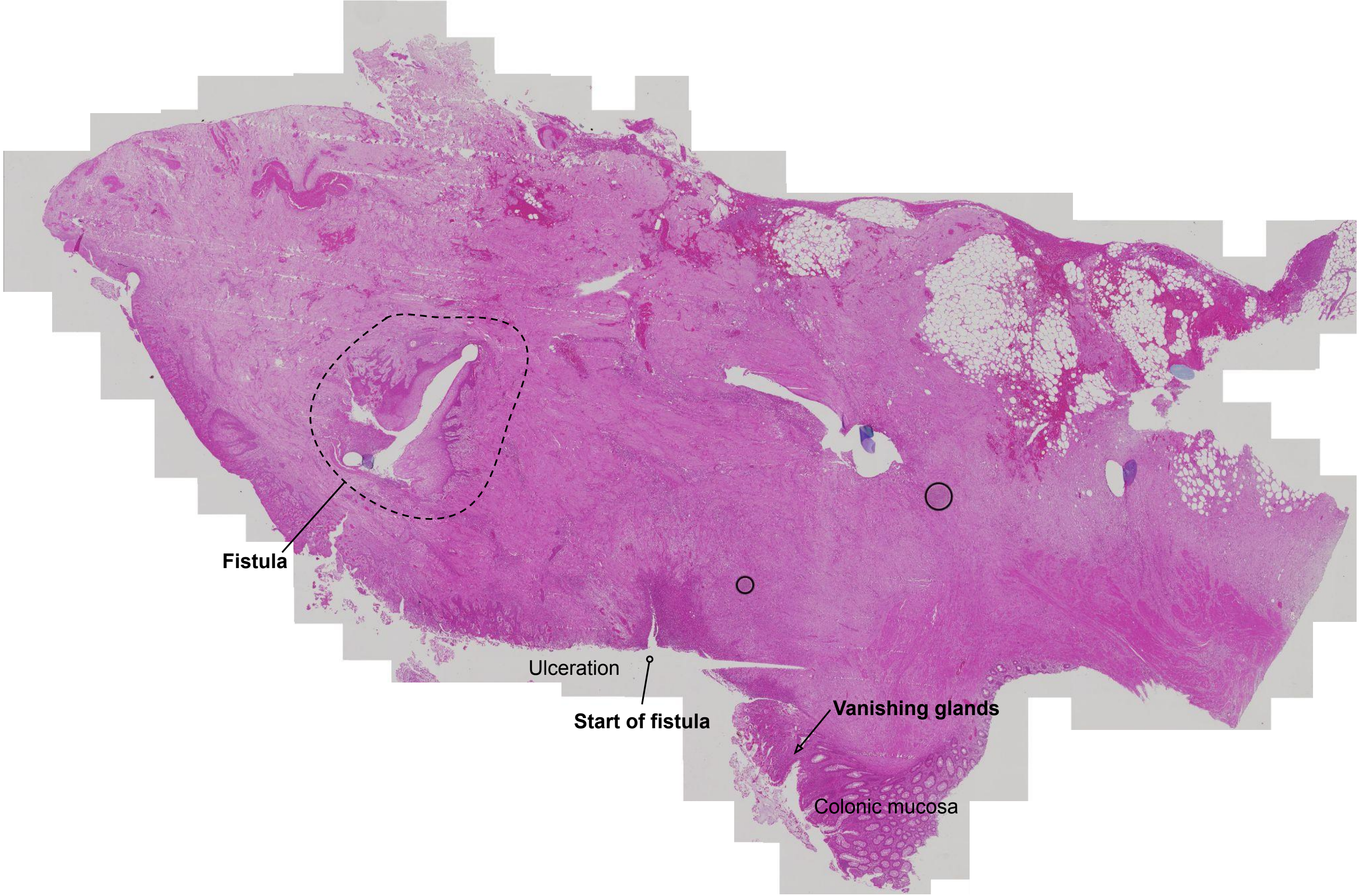

BAY 113385_21

COLO-
CUTANEOUS
CD FISTULA

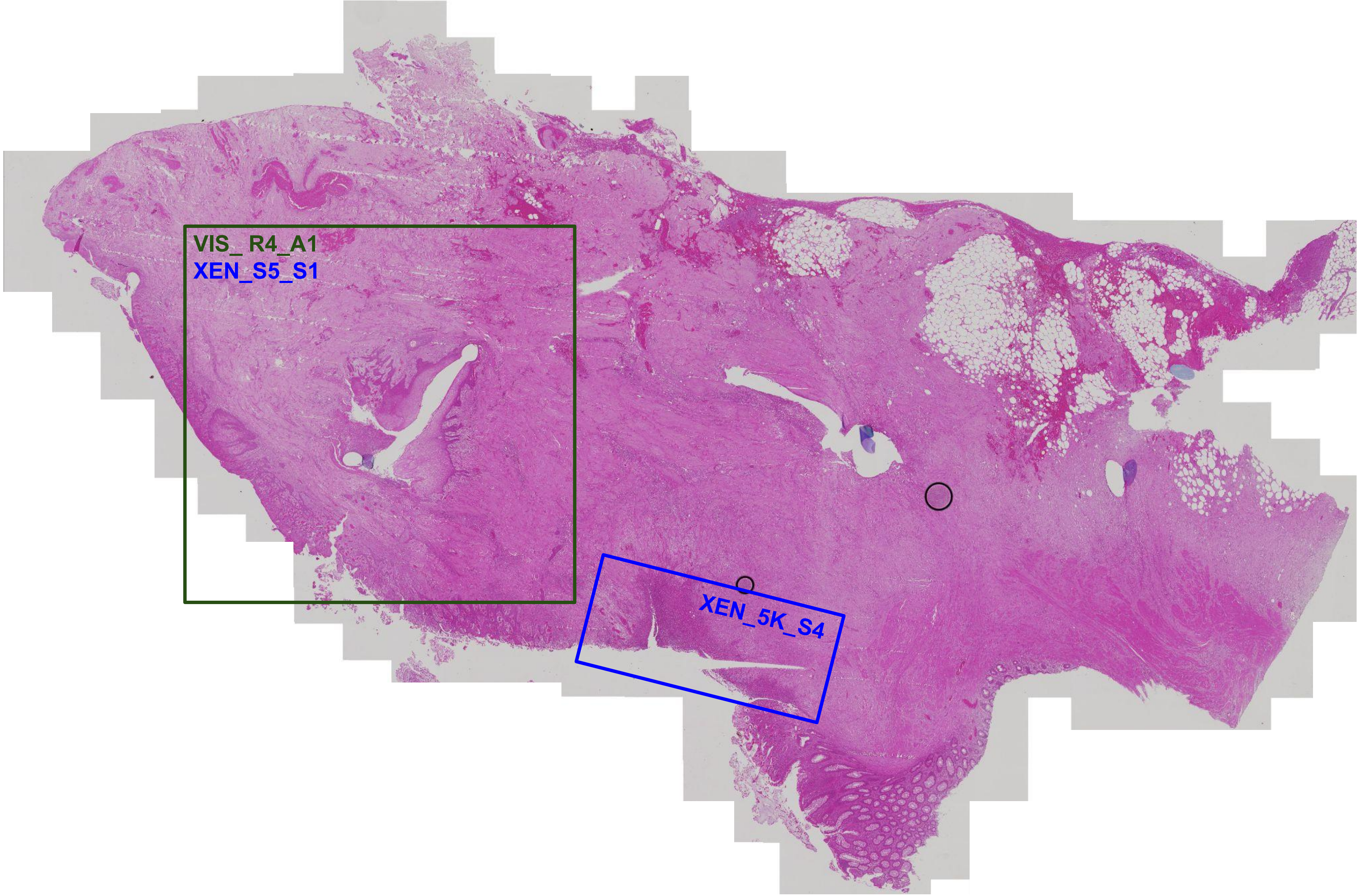

BAY 113385_21

COLOCUTANEOUS CD FISTULA

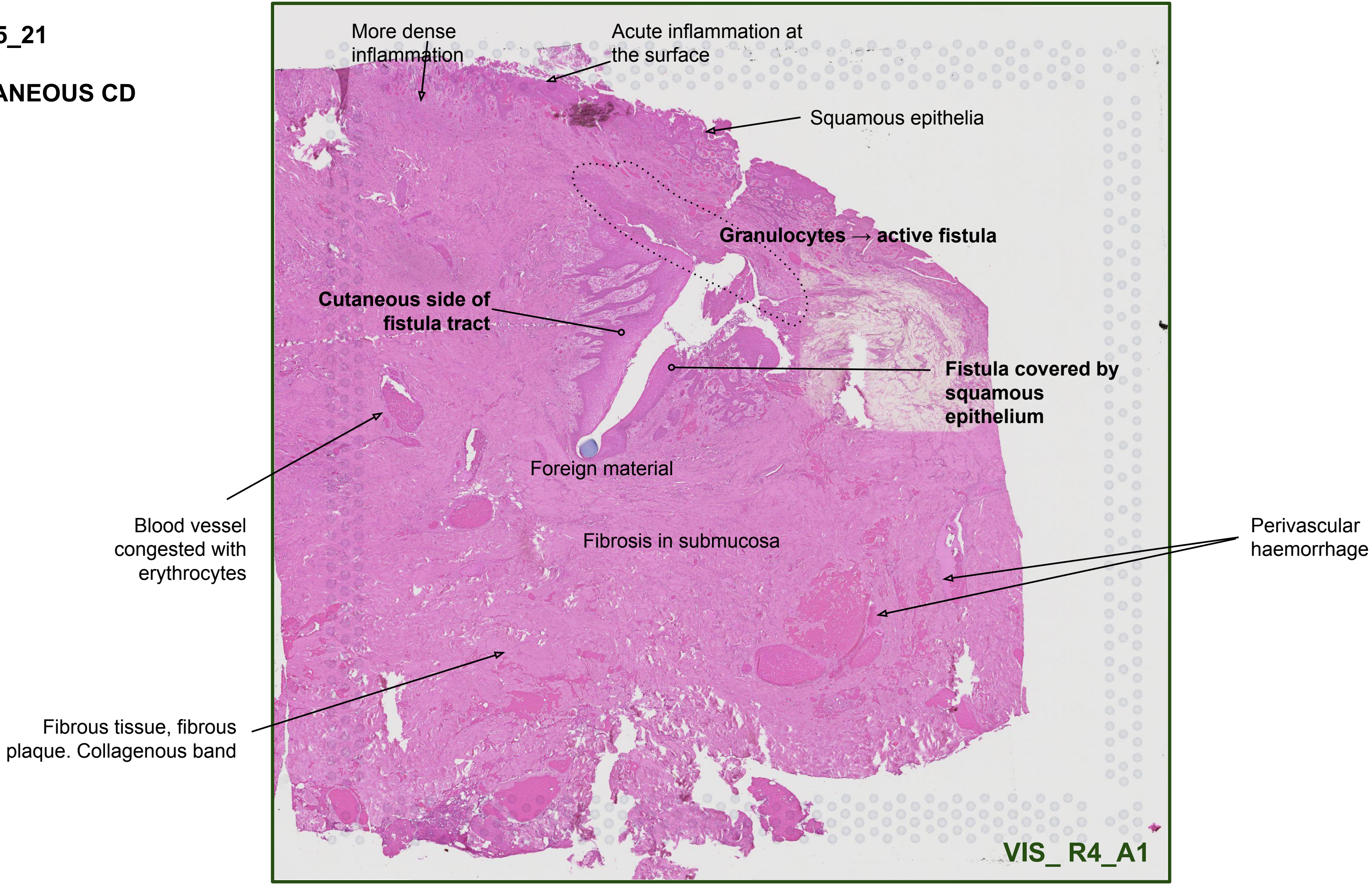

BAY 113385_21

COLOCUTANEOUS CD FISTULA

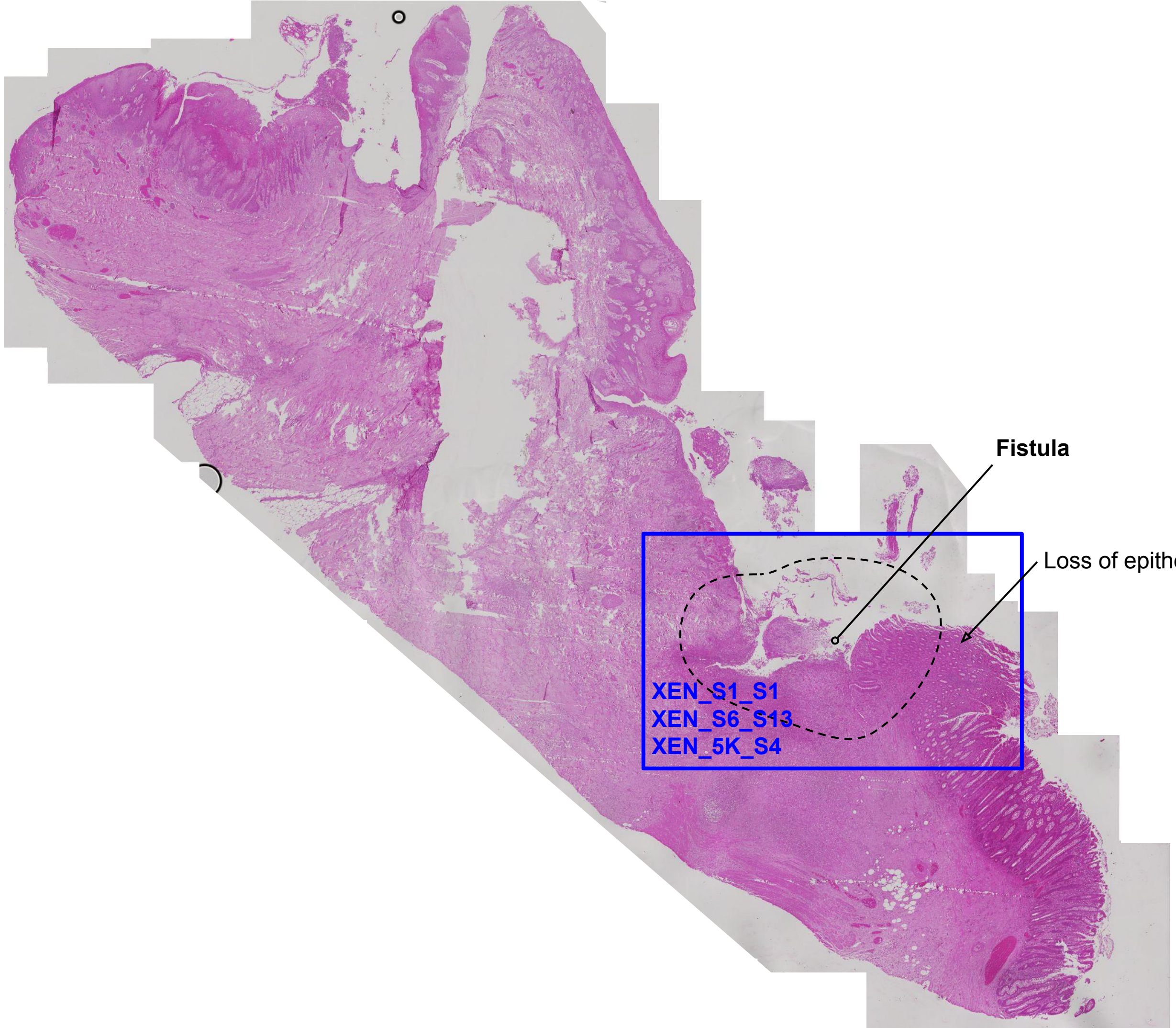

BAY 035829_17

ILEAL CD FISTULA

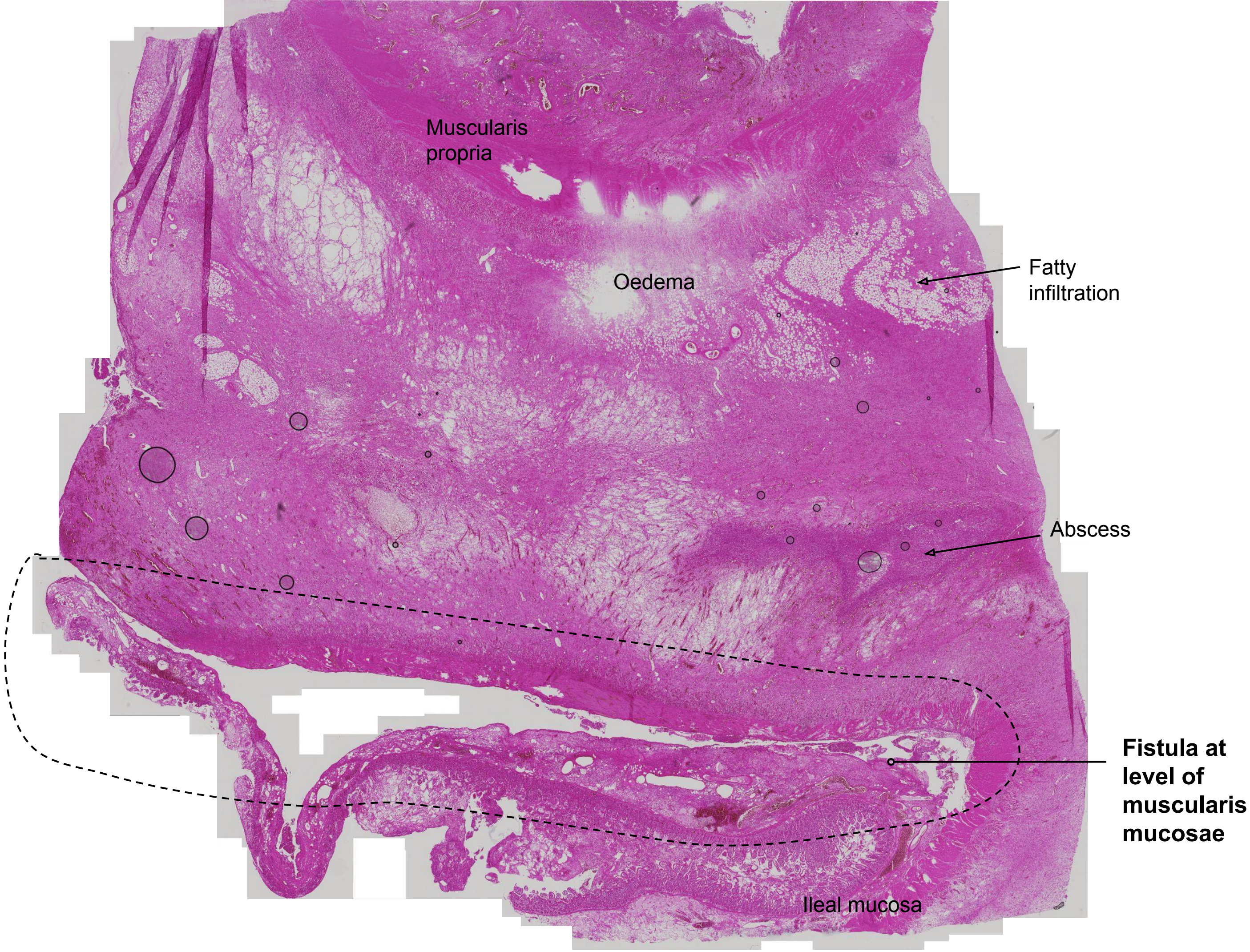

BAY 035829_17

ILEAL CD FISTULA

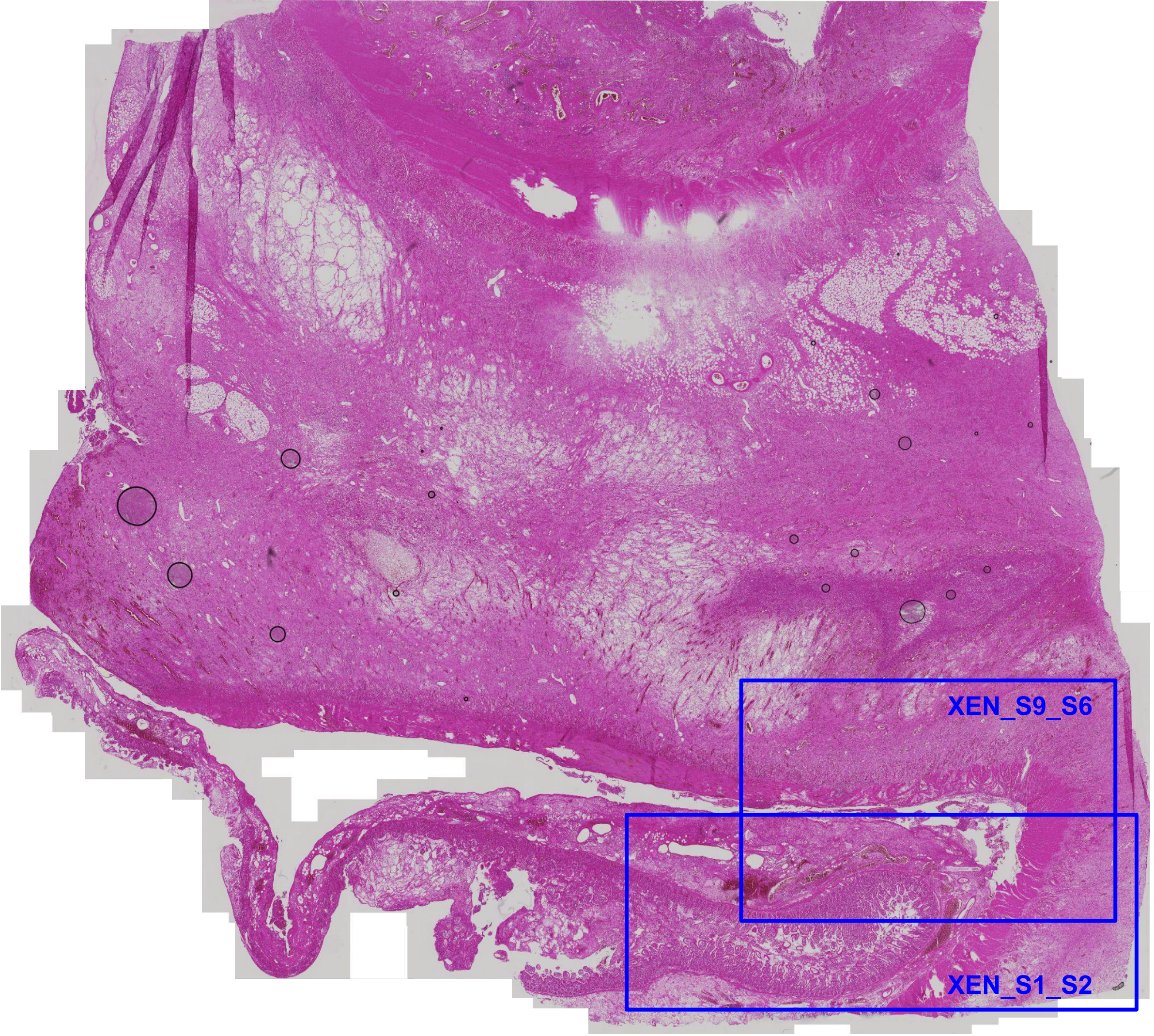

BAY 100435_22

PERIANAL CD FISTULA

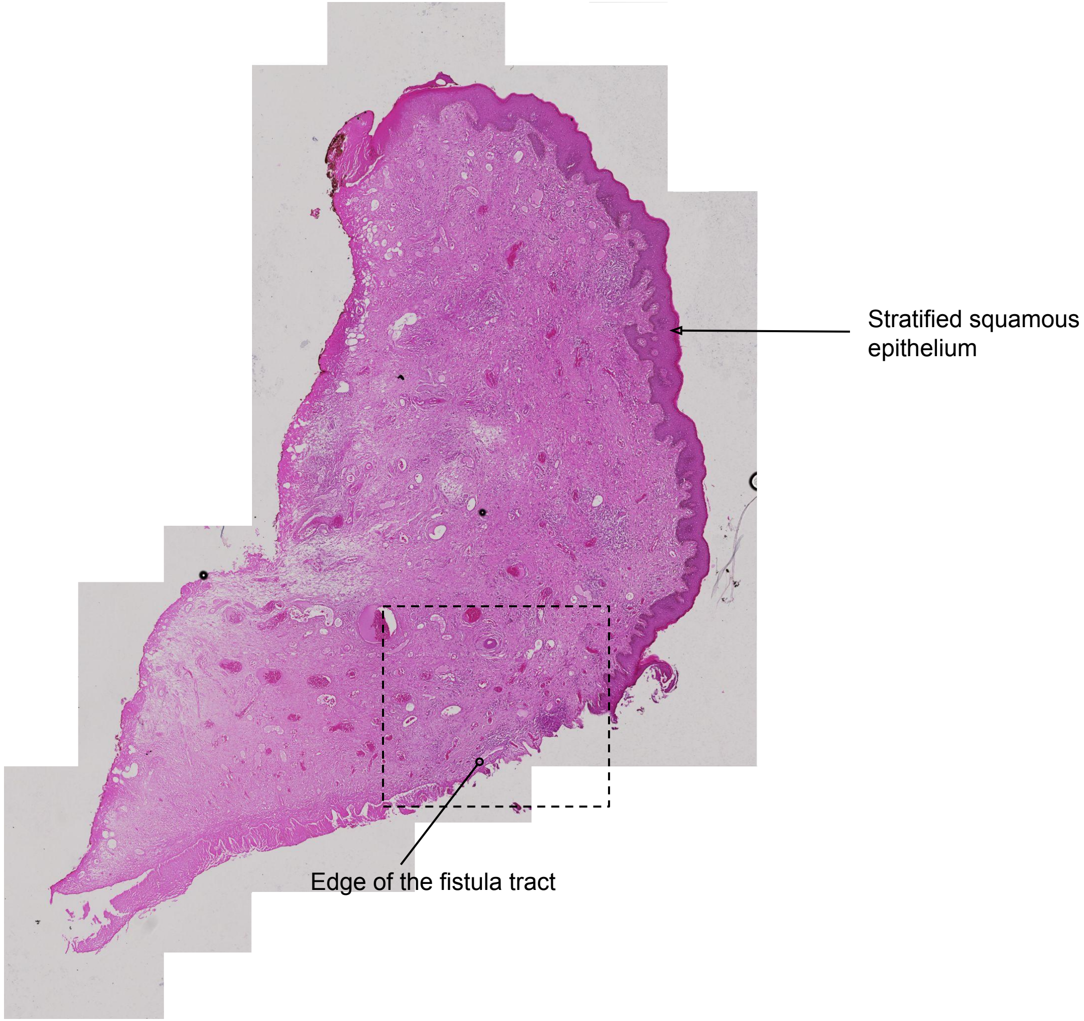

BAY 100435_22

PERIANAL CD FISTULA

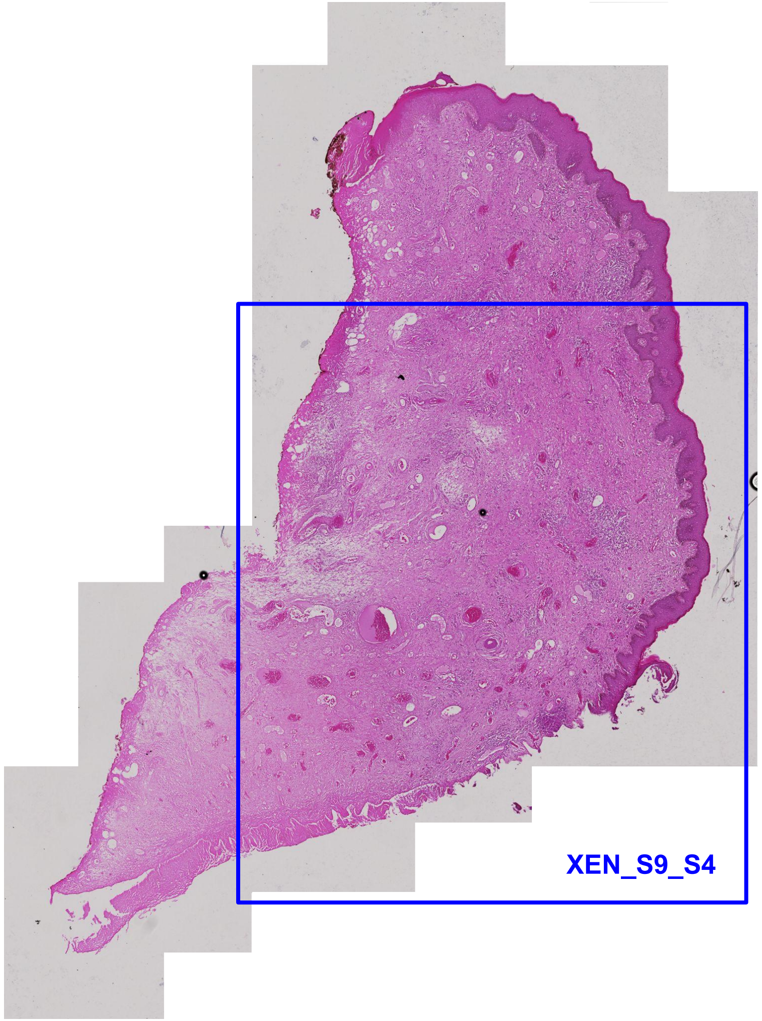

BAY 104603_20

ILEOCAECAL CD FISTULA

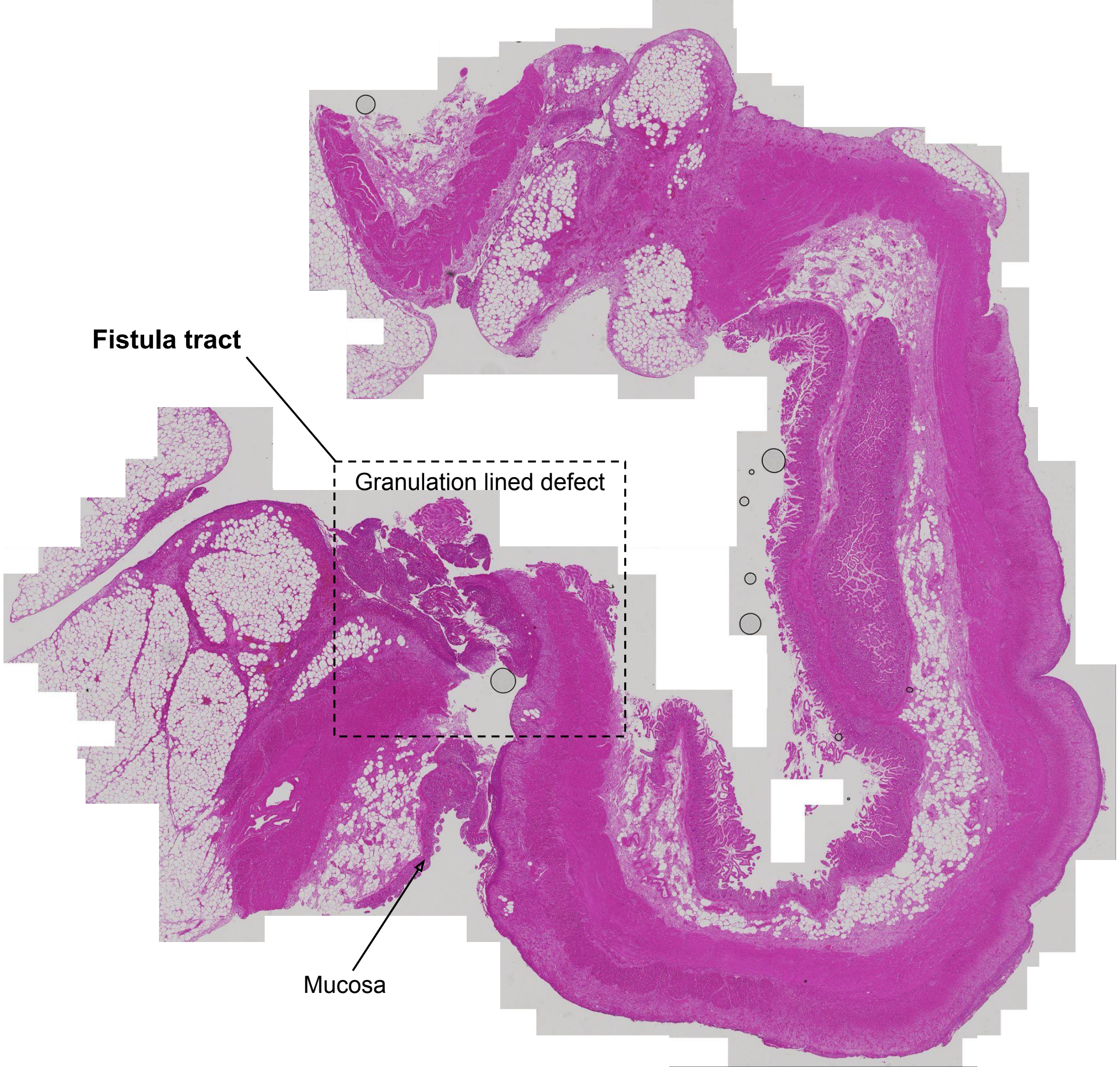

BAY 104603_20

ILEOCAECAL CD FISTULA

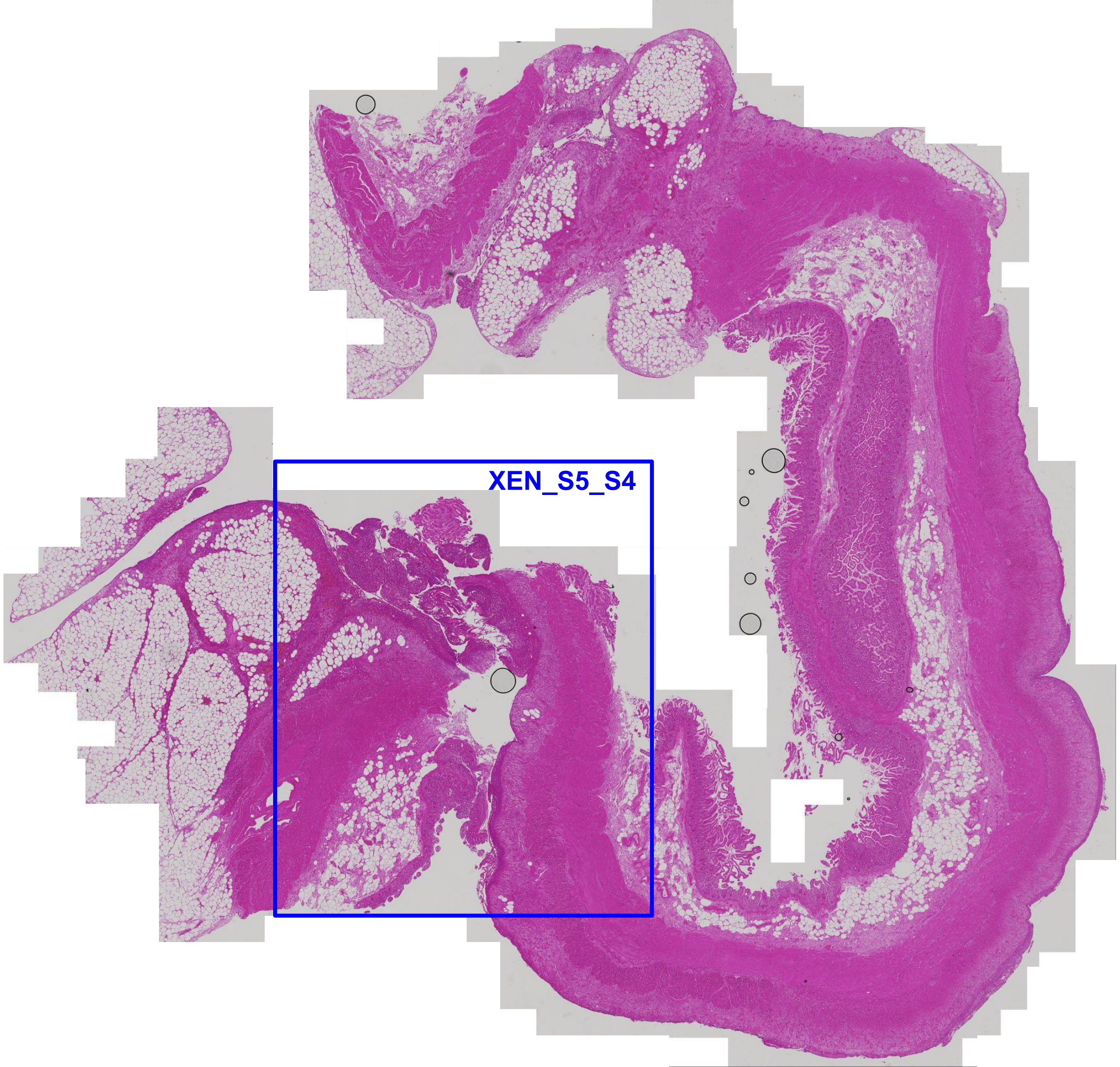

BAY 104603_20

ILEOCAECAL CD FISTULA

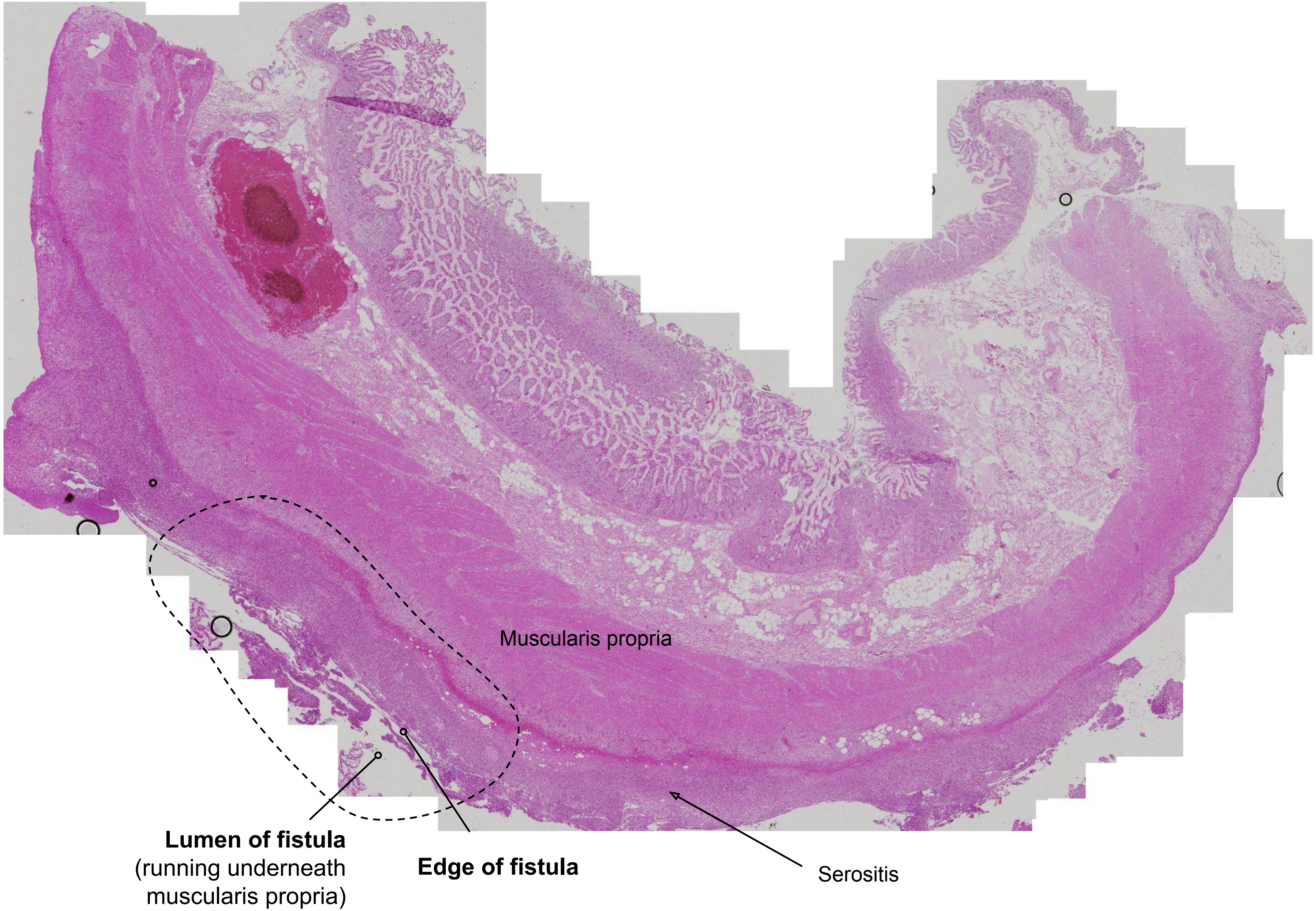

BAY 104603_20

ILEOCAECAL CD FISTULA

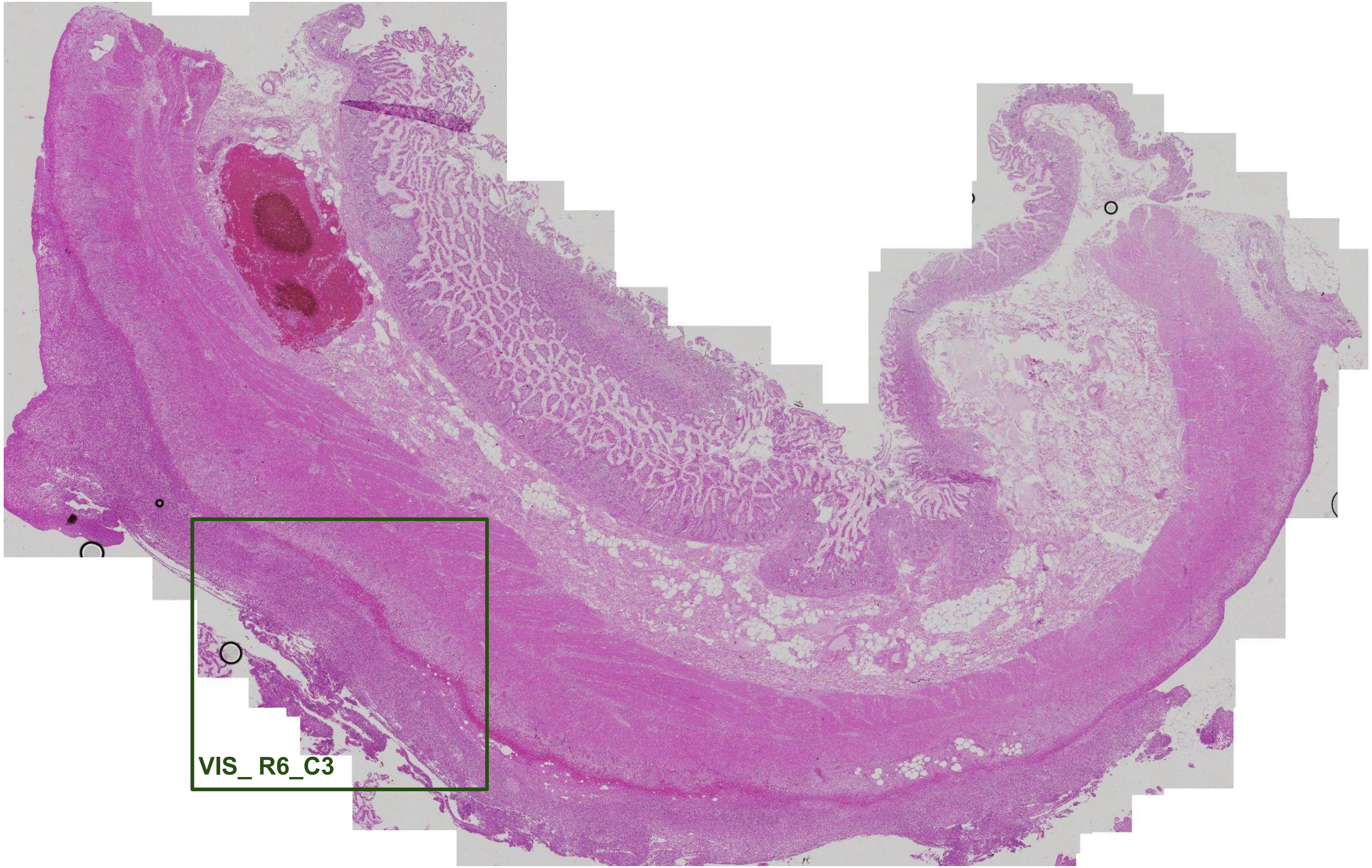

BAY 104603_20

ILEOCAECAL CD FISTULA

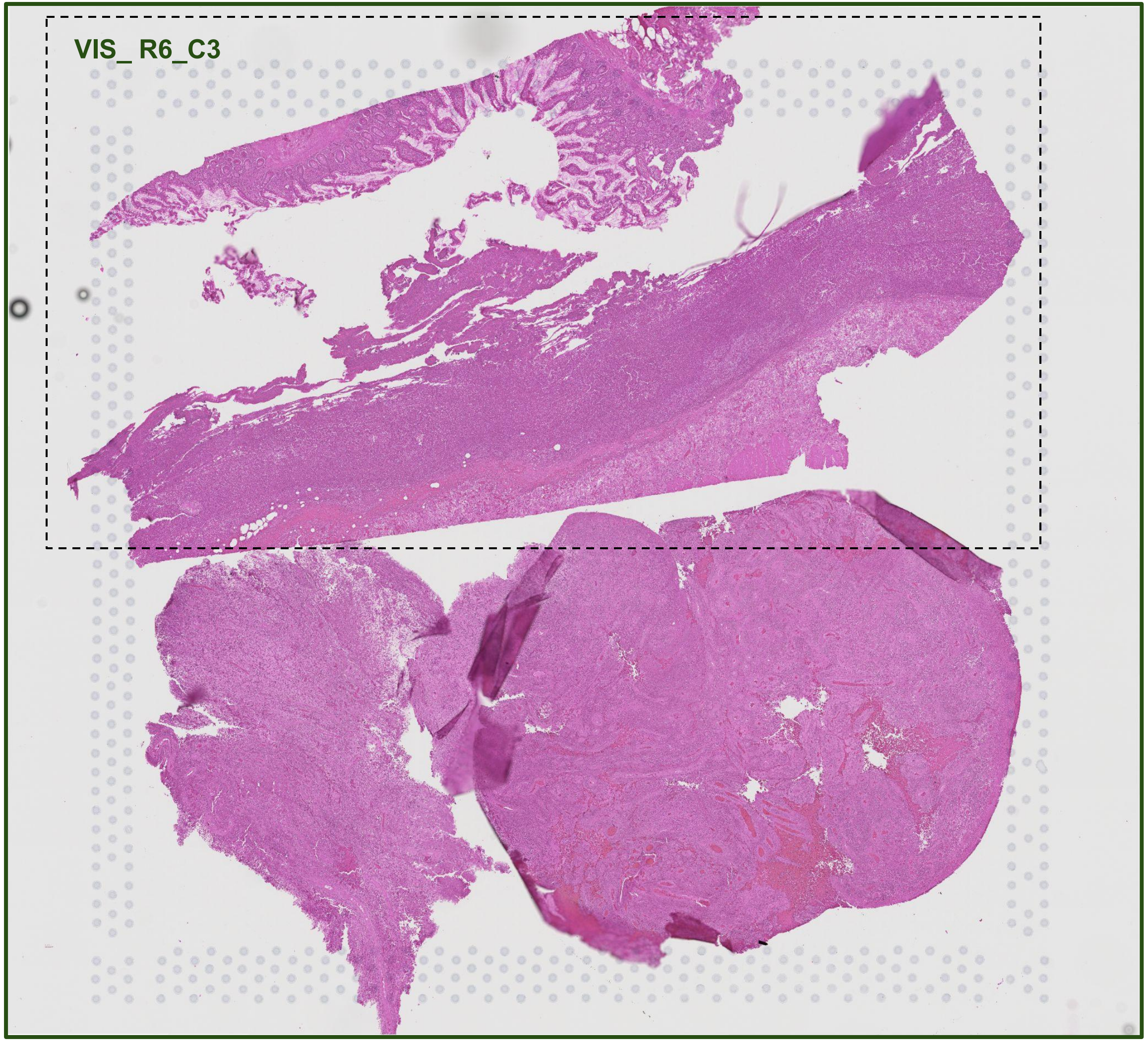

BAY 105338_20

TRANSSPHINCTERIC CD FISTULA

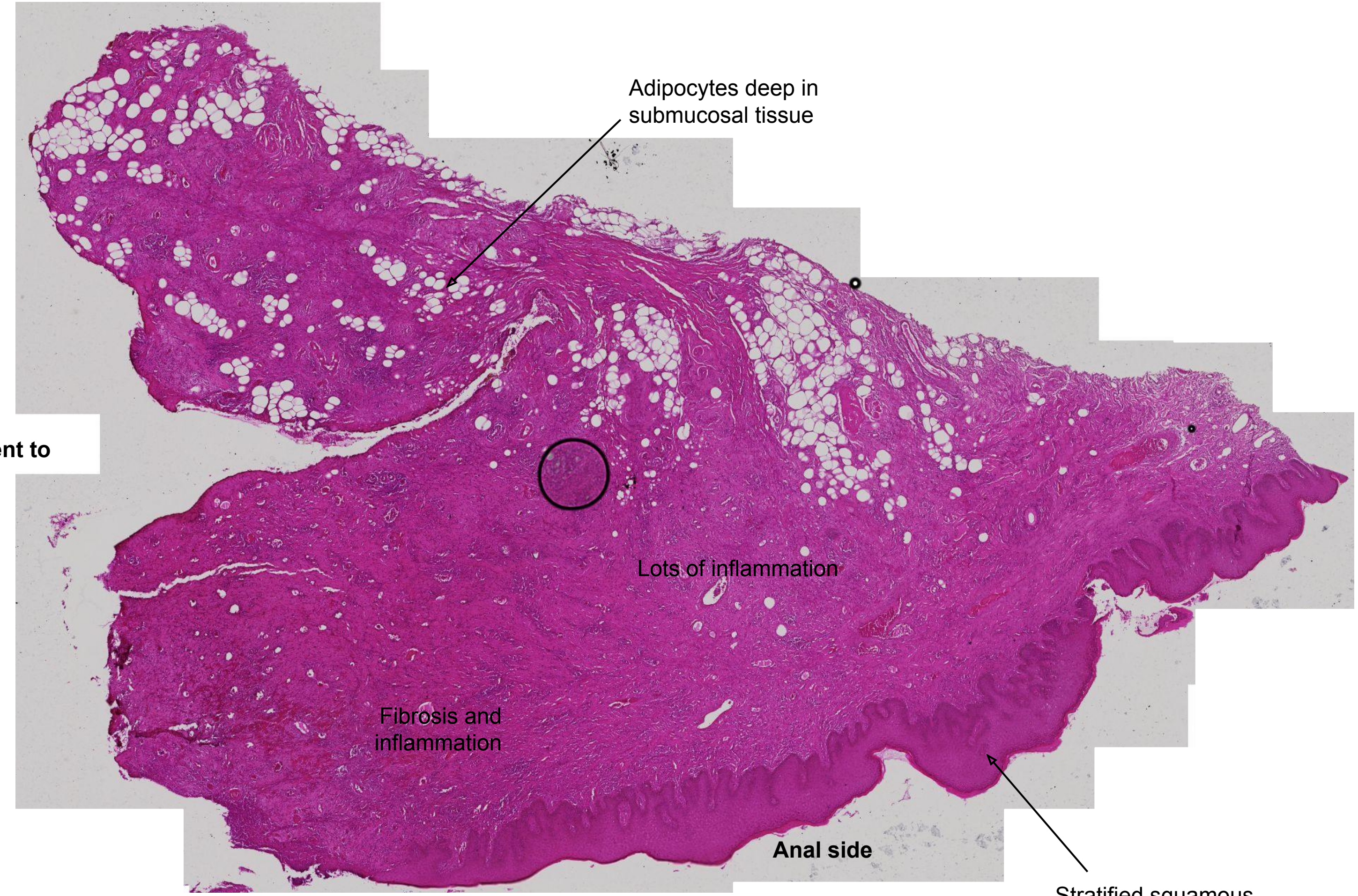

BAY 105338_20

TRANSSPHINCTERIC CD FISTULA

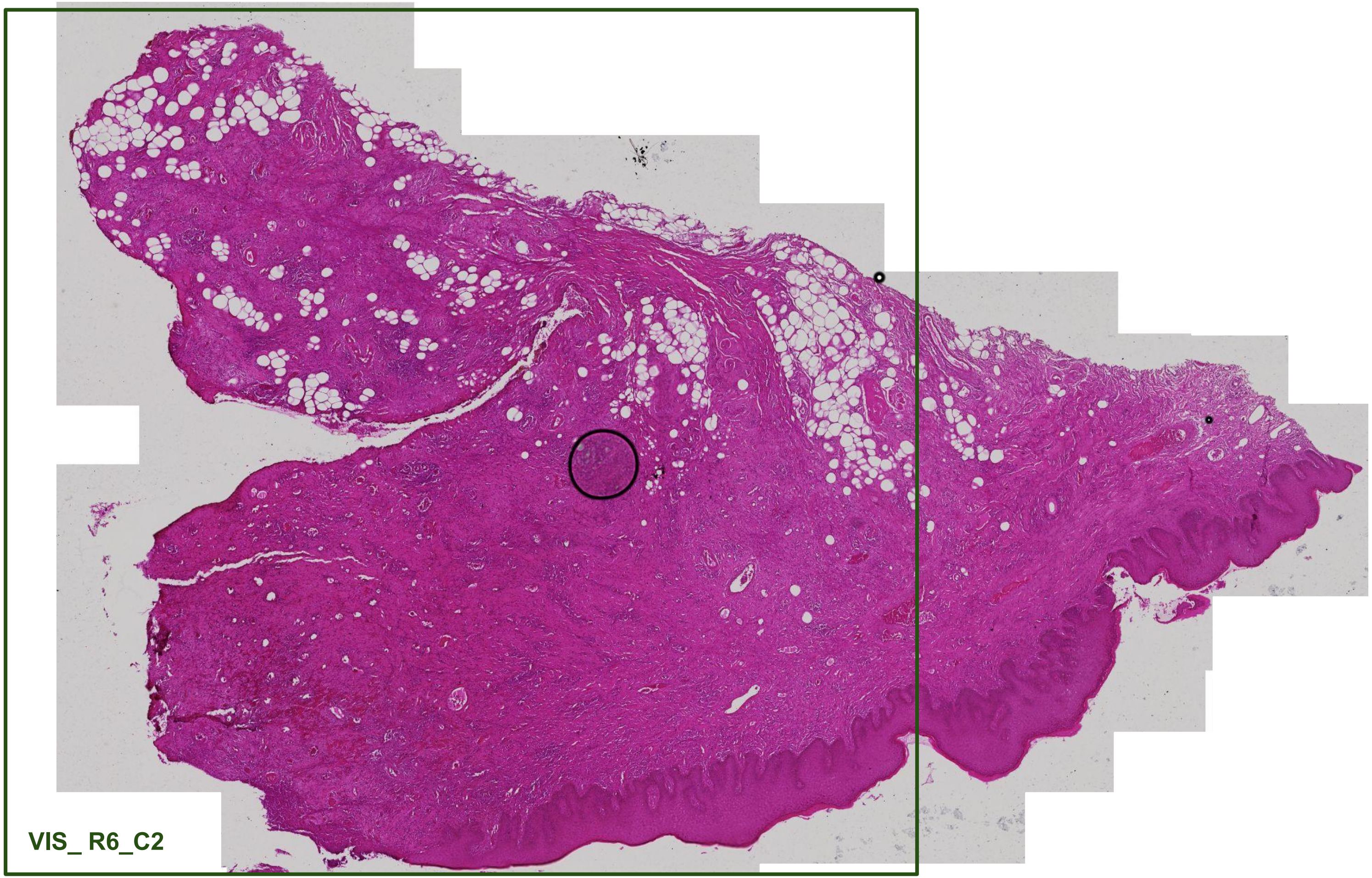

BAY 105338_20

TRANSSPHINCTERIC CD FISTULA

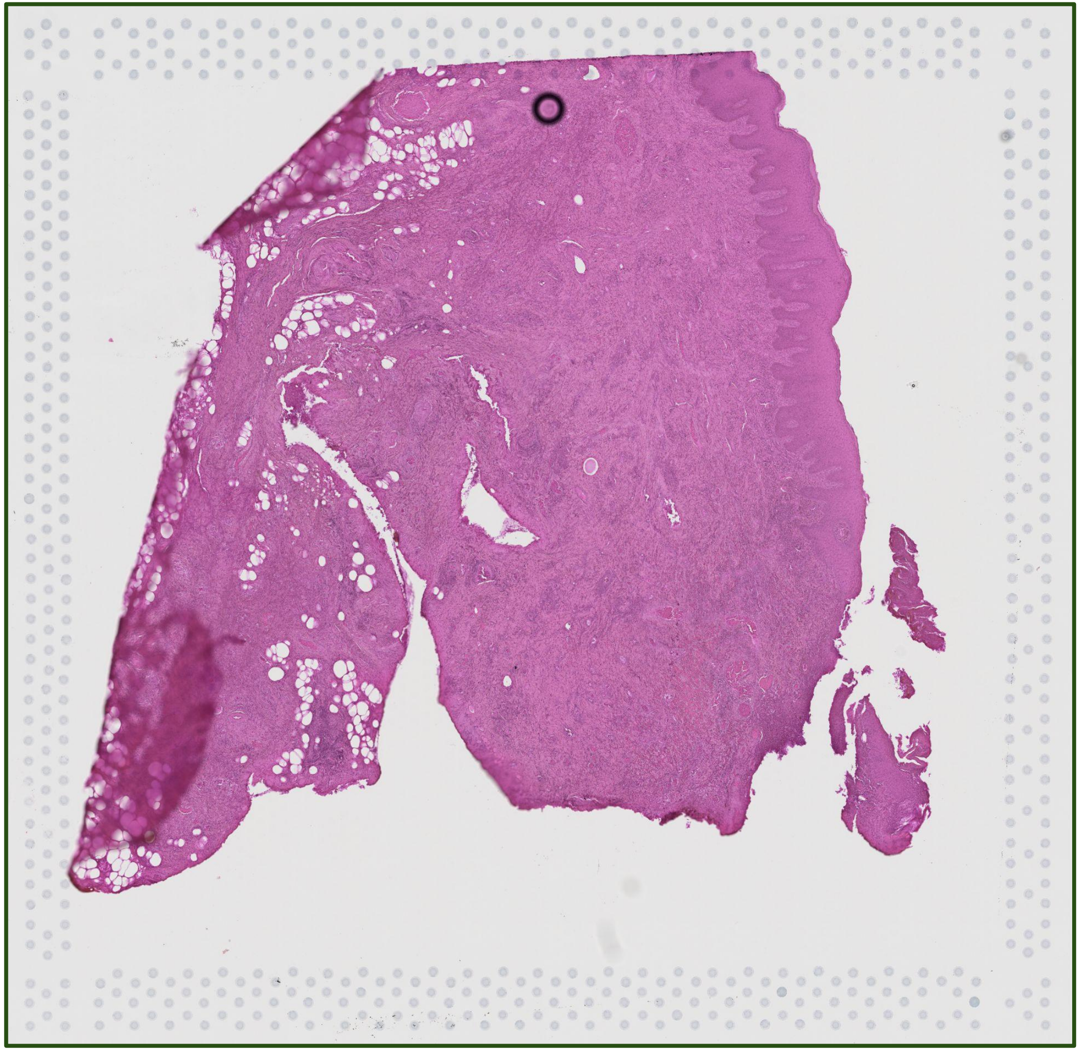

BAY 115964_11

ILEOCOLIC CD FISTULA (BIOPSY)

Mucosa (fistula biopsy)

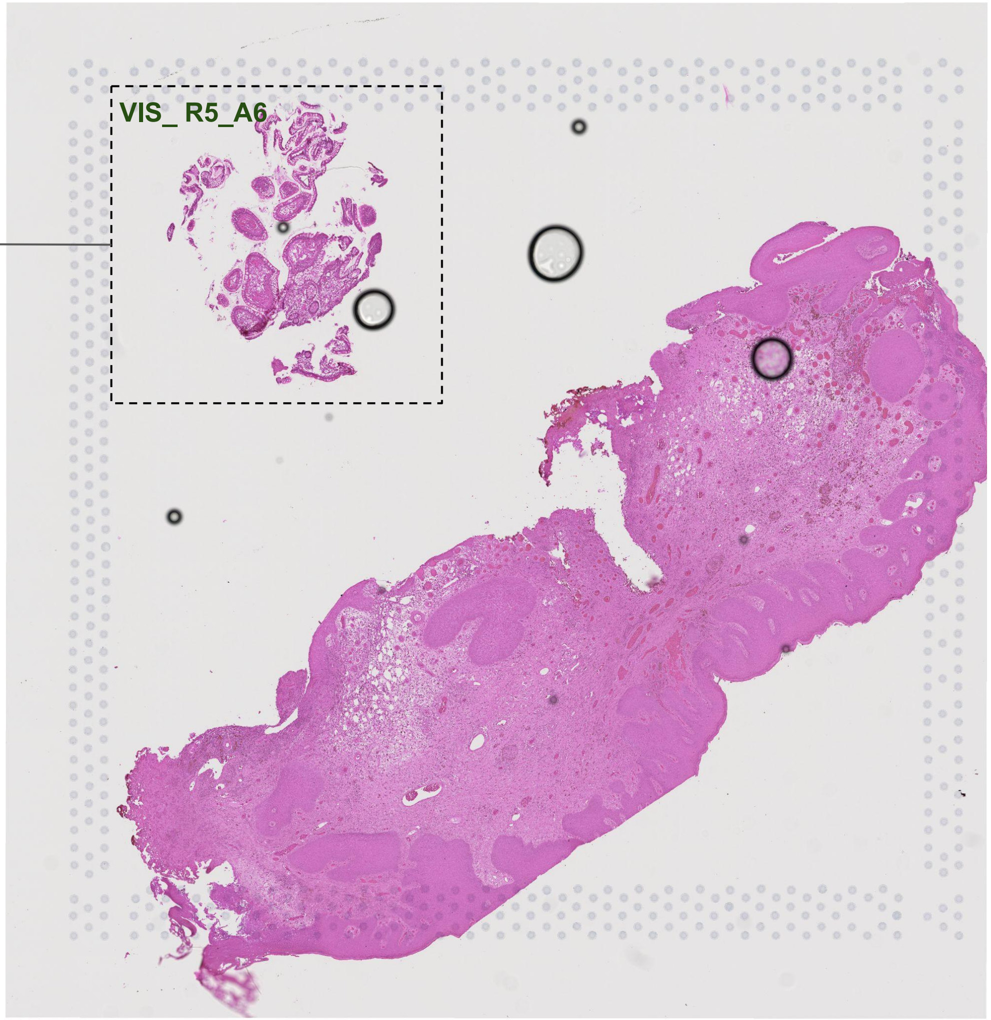

BAY_137968_11

ILEOCOLIC CD FISTULA

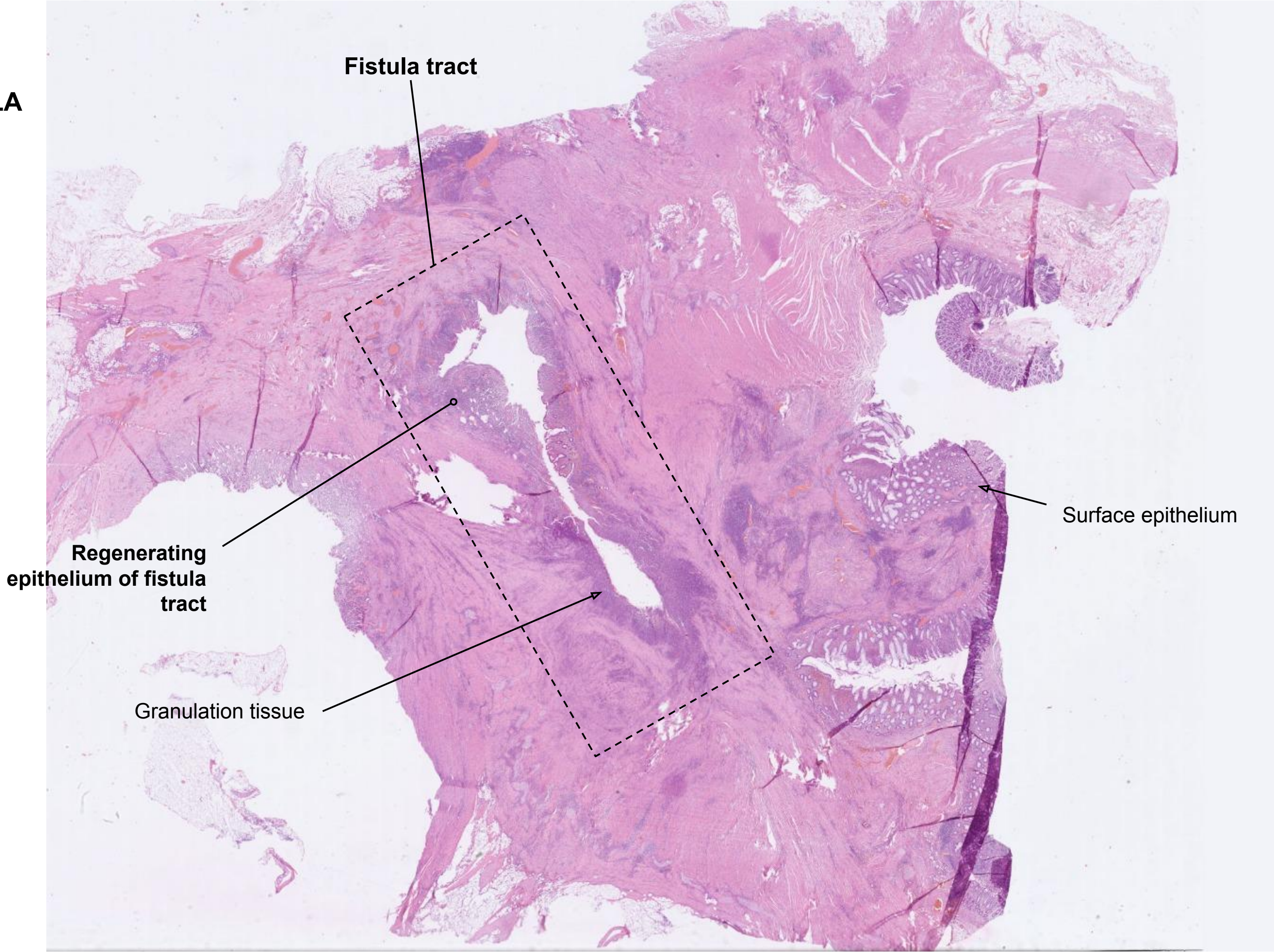

BAY_137968_11

ILEOCOLIC CD FISTULA

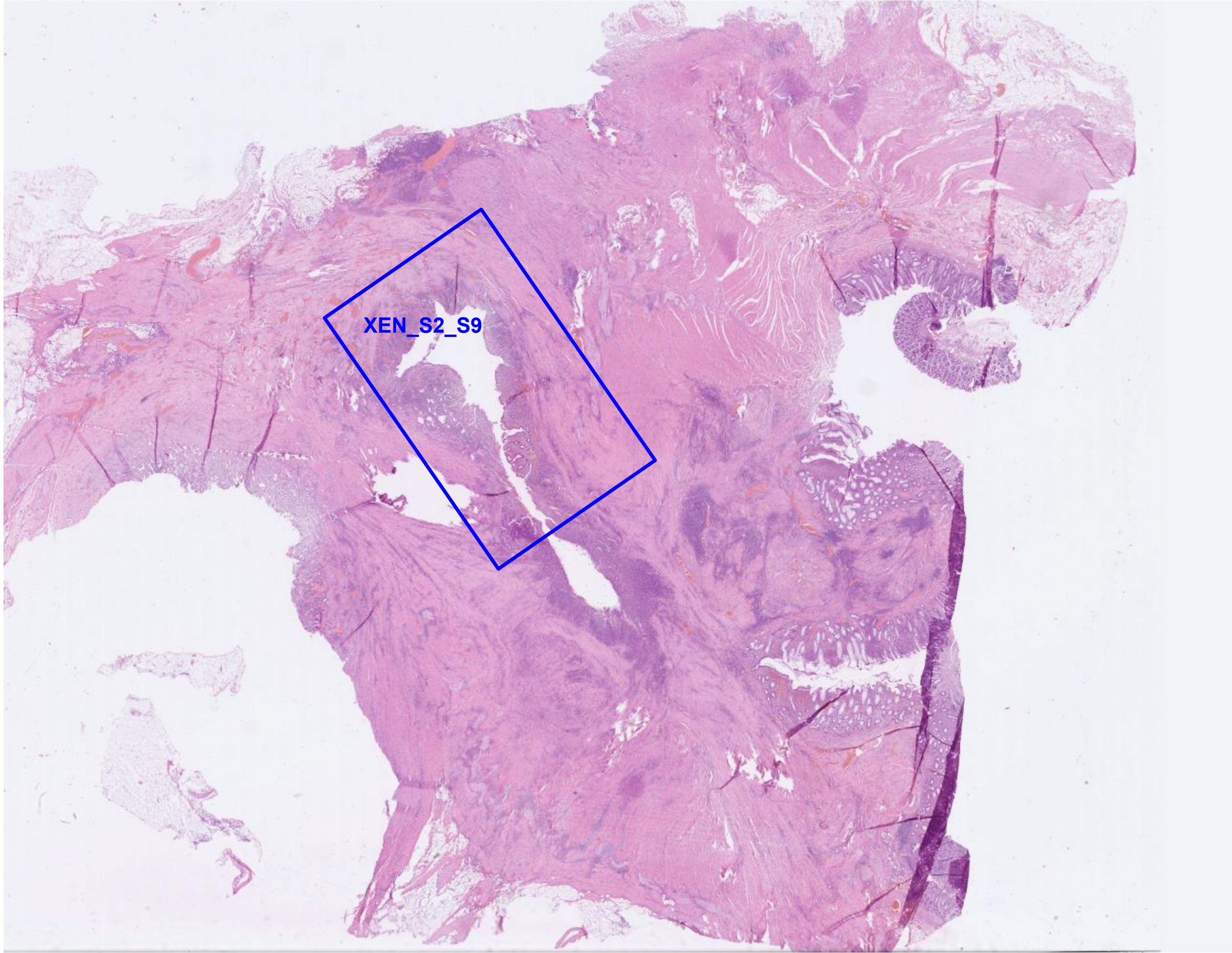

BAY_20986_23

JEJUNAL CD FISTULA

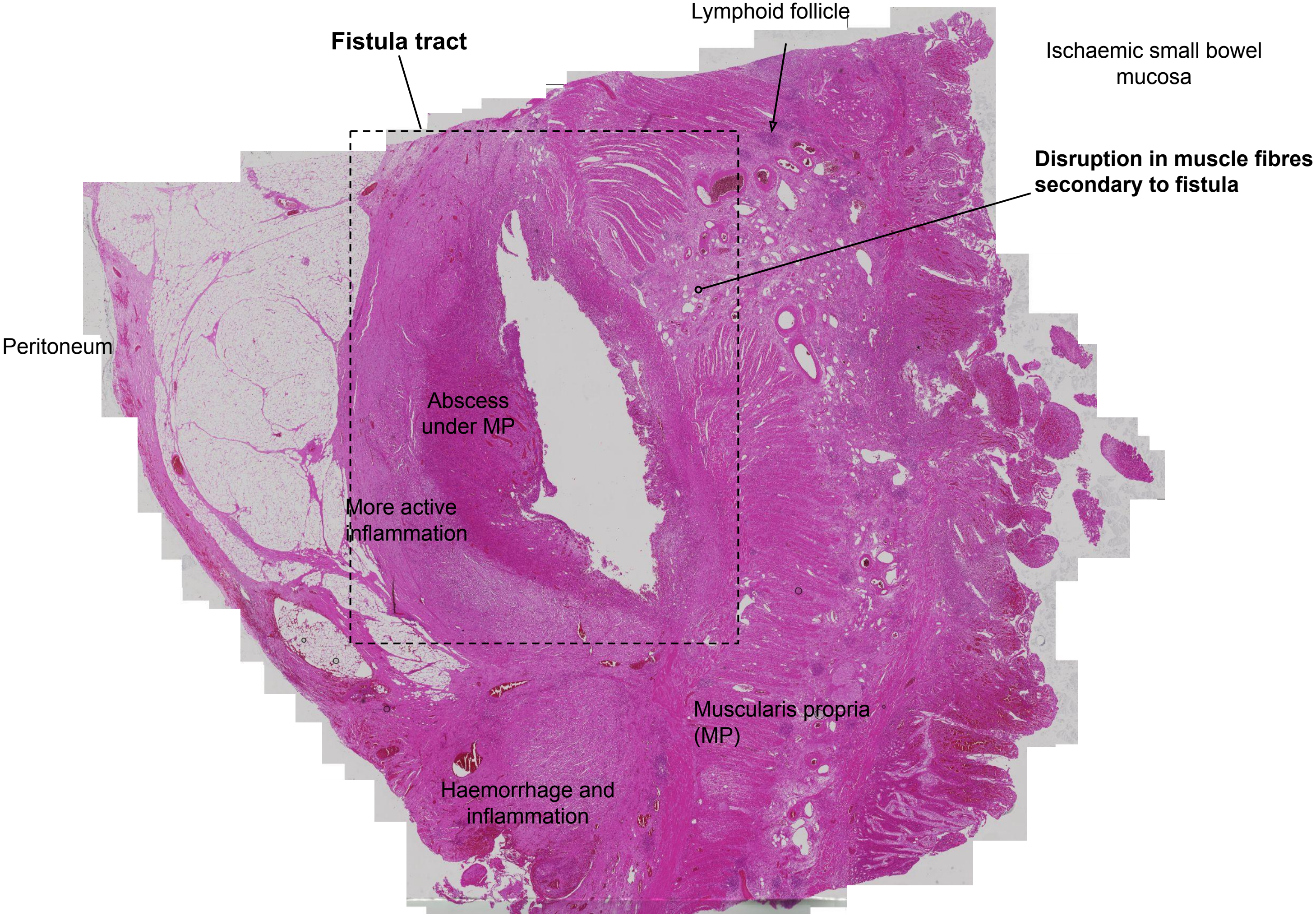

BAY_20986_23

JEJUNAL CD FISTULA

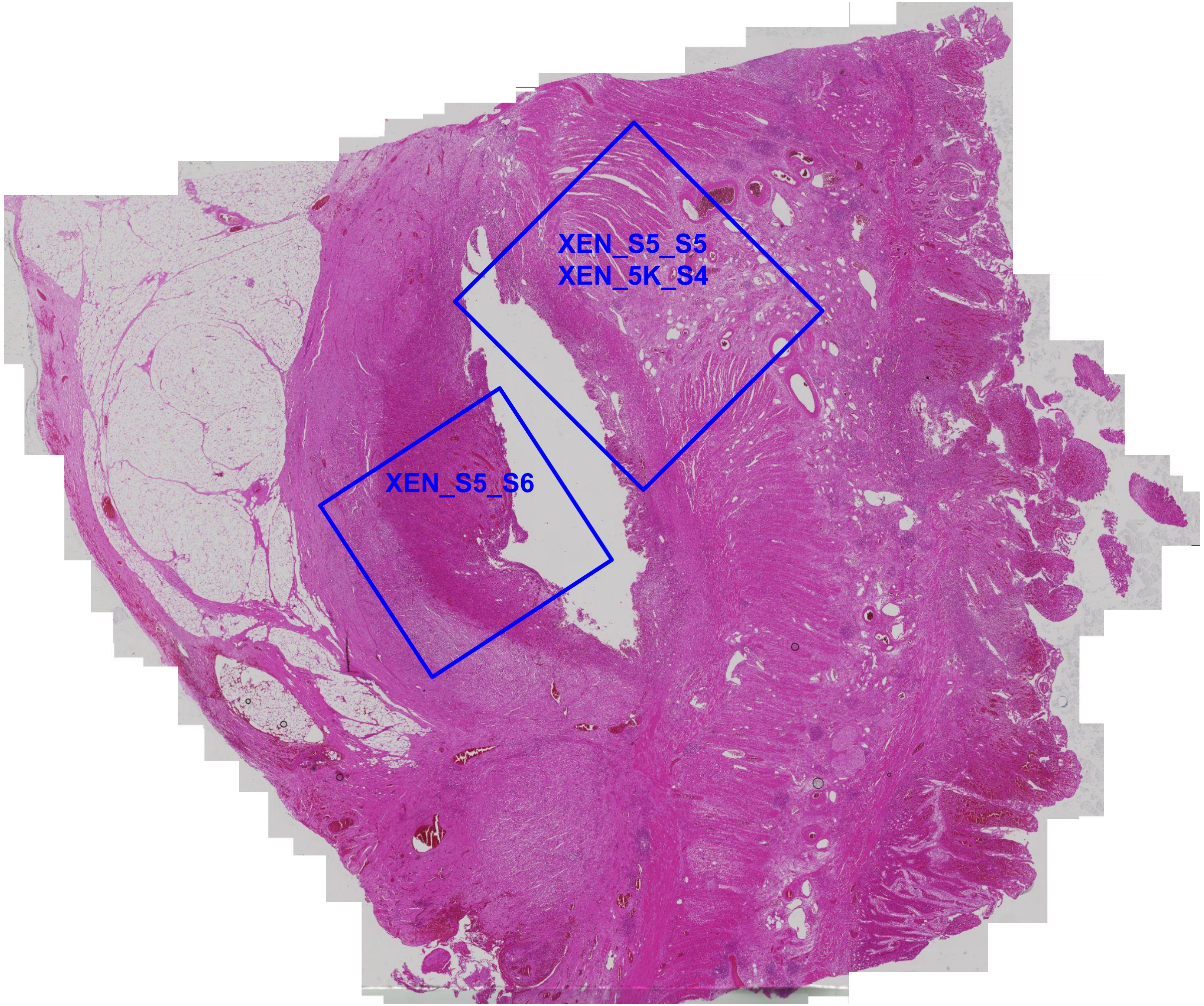

BAY_22635_21

SIGMOID-JEJUNAL CD
FISTULA

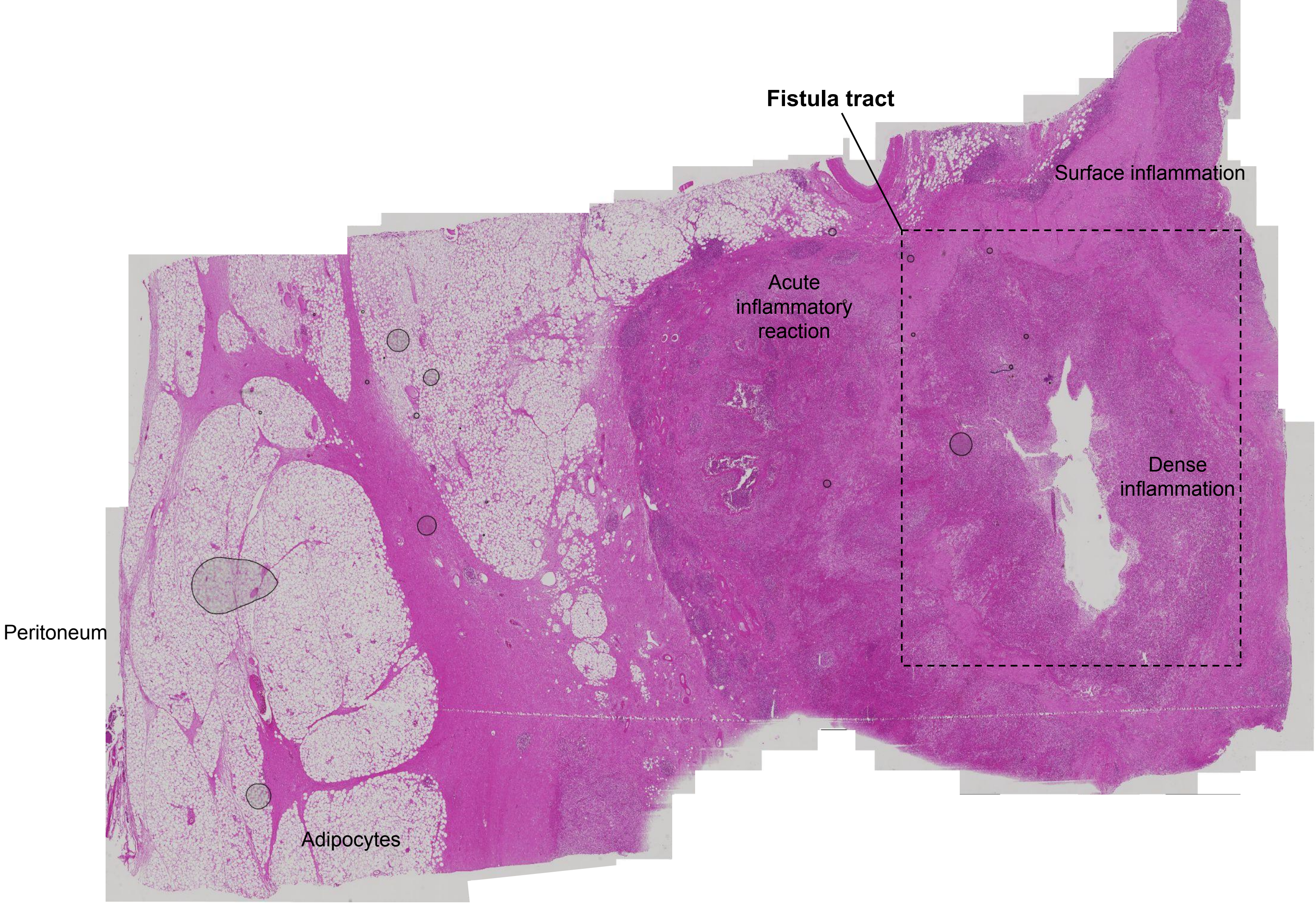

BAY_22635_21

SIGMOID-JEJUNAL CD
FISTULA

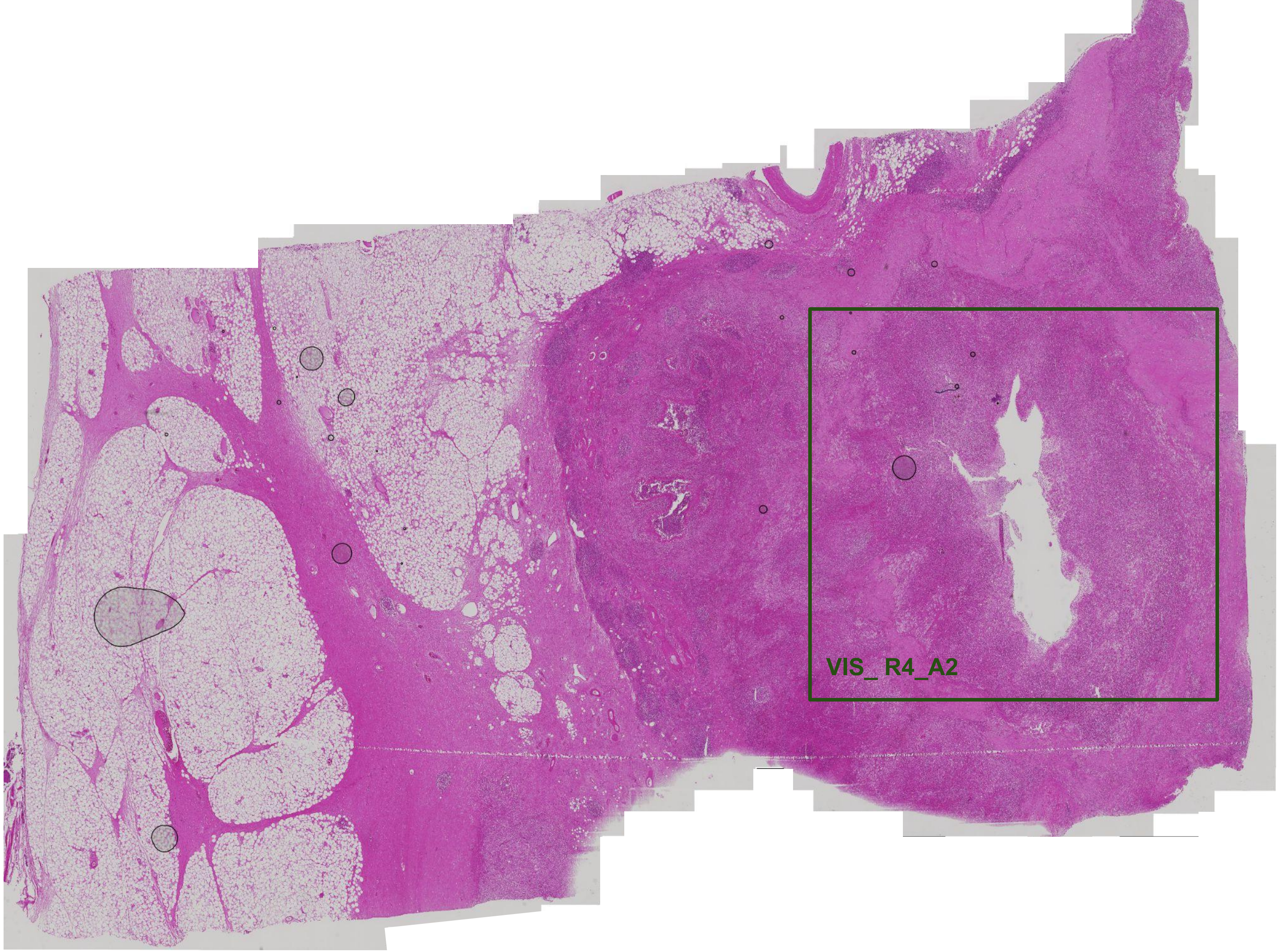

BAY_22635_21

SIGMOID-JEJUNAL CD FISTULA

Abscess in a fistula section

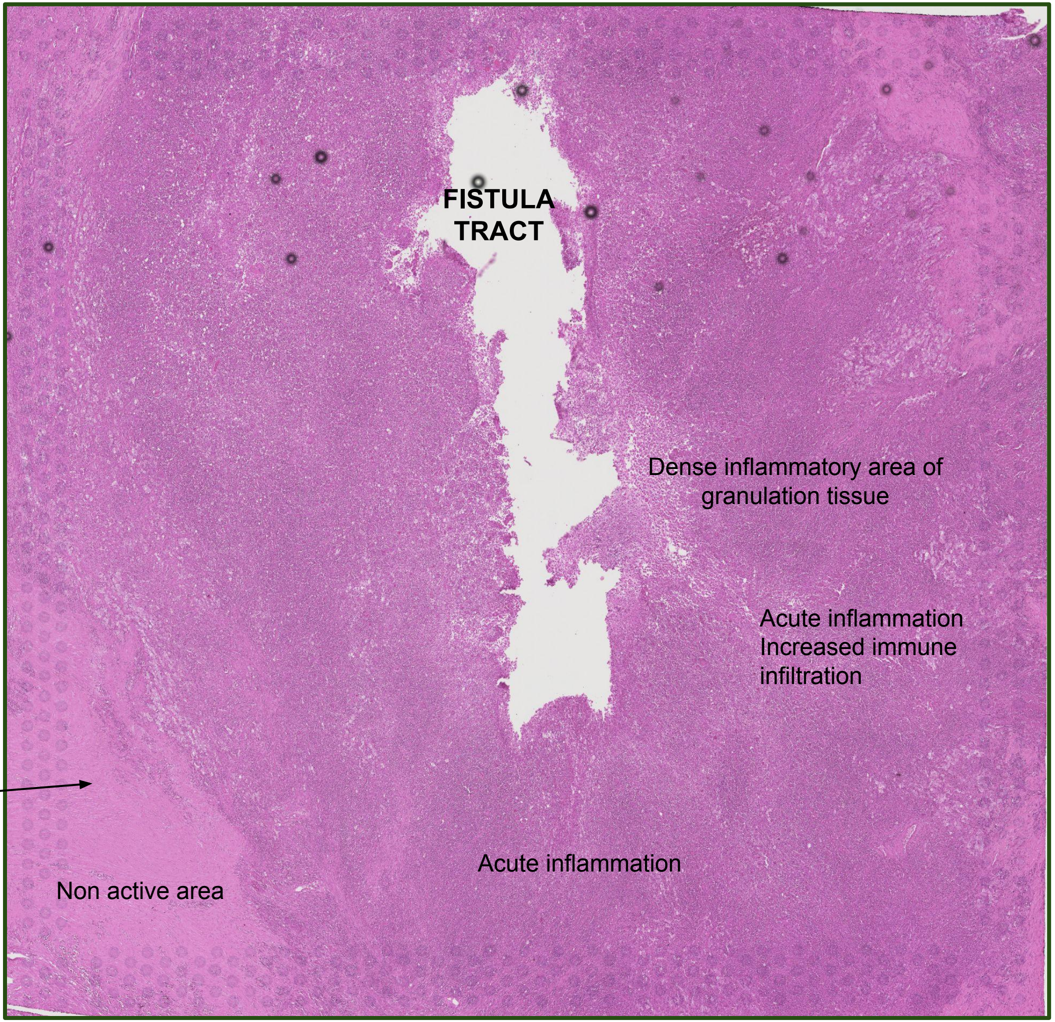

BAY_26534_12

ILEO-PERITONEAL CD FISTULA

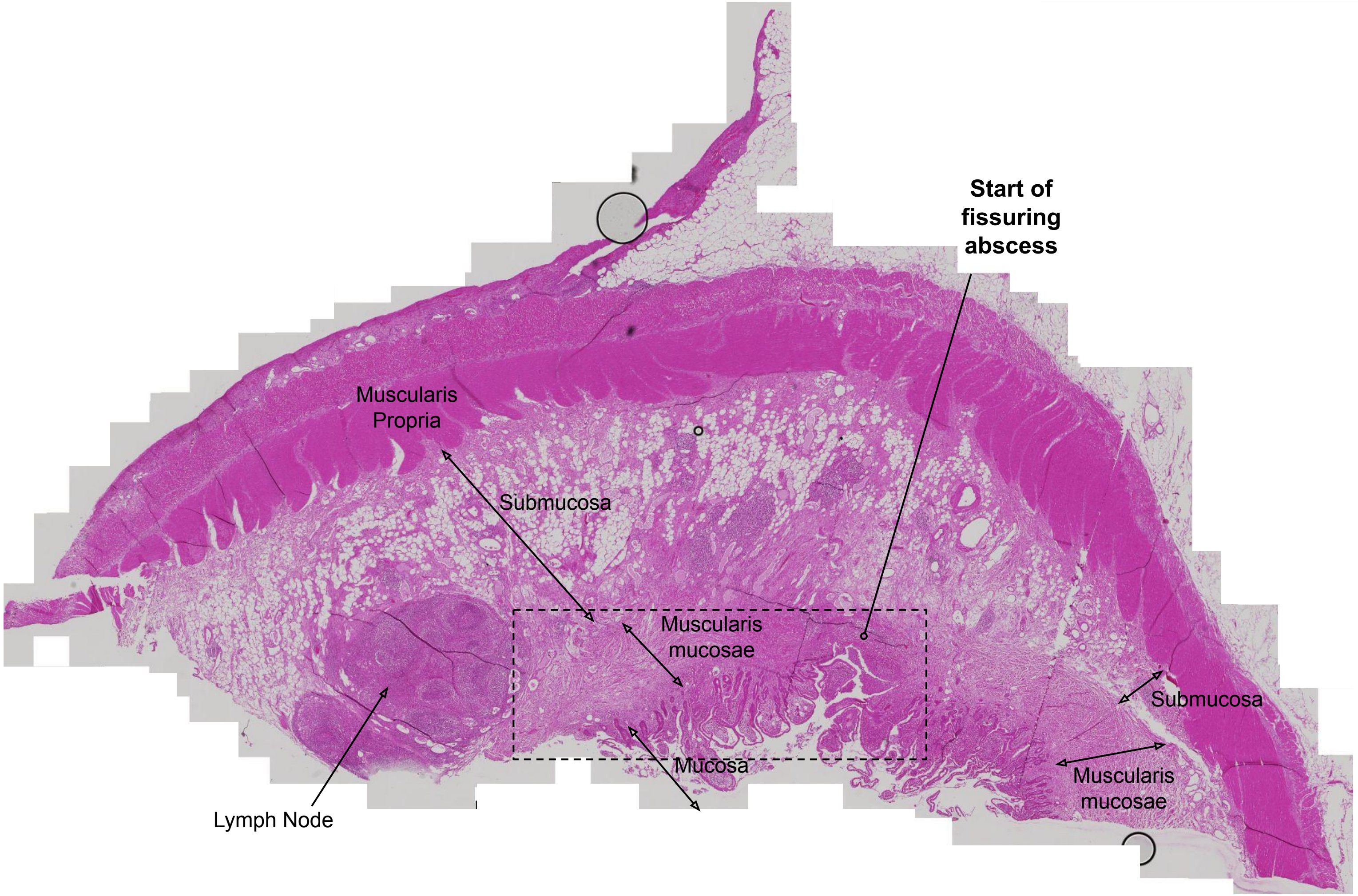

BAY_26534_12

ILEO-PERITONEAL
CD FISTULA

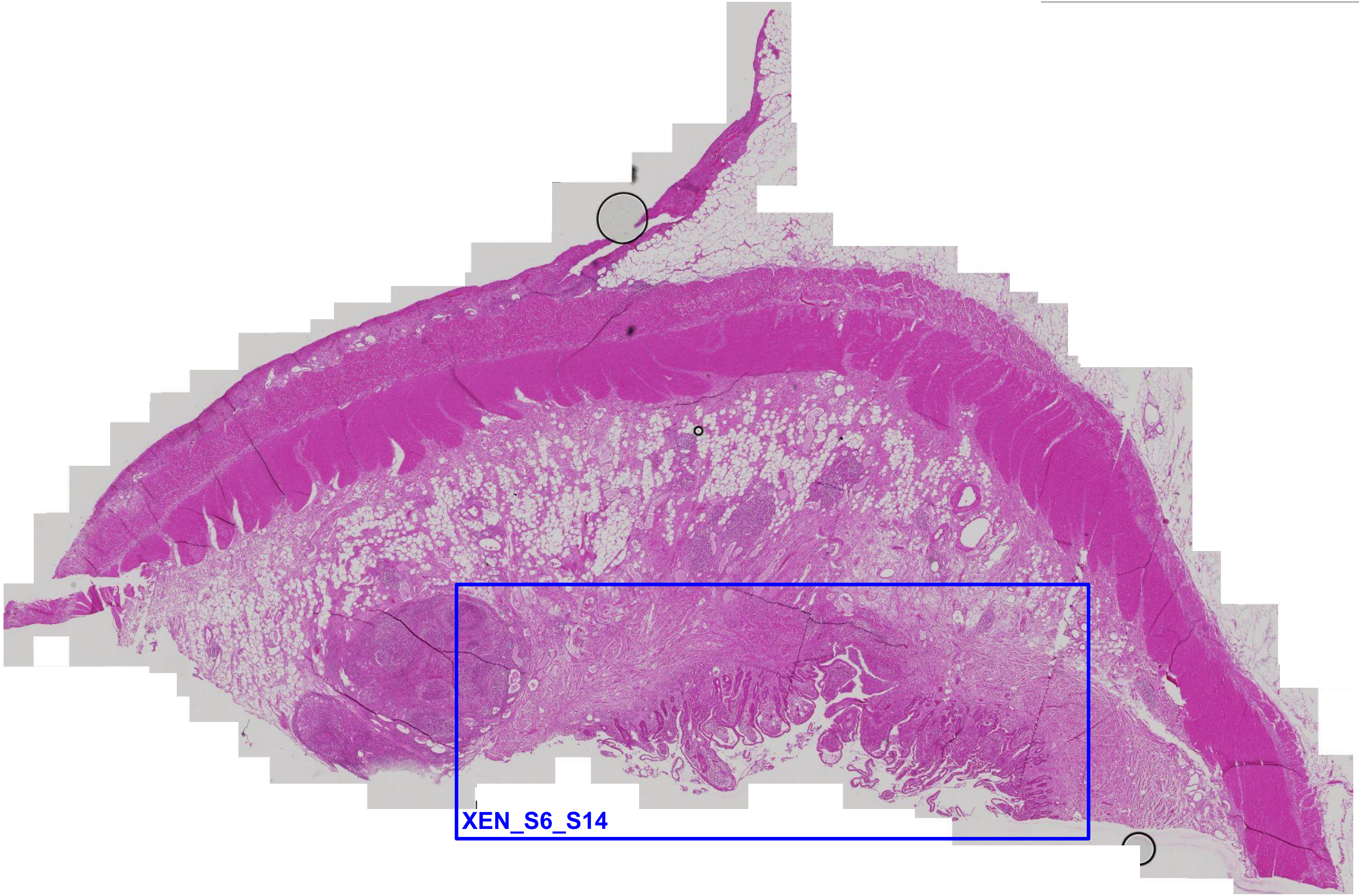

BAY_48715_20

ANAL CD FISTULA

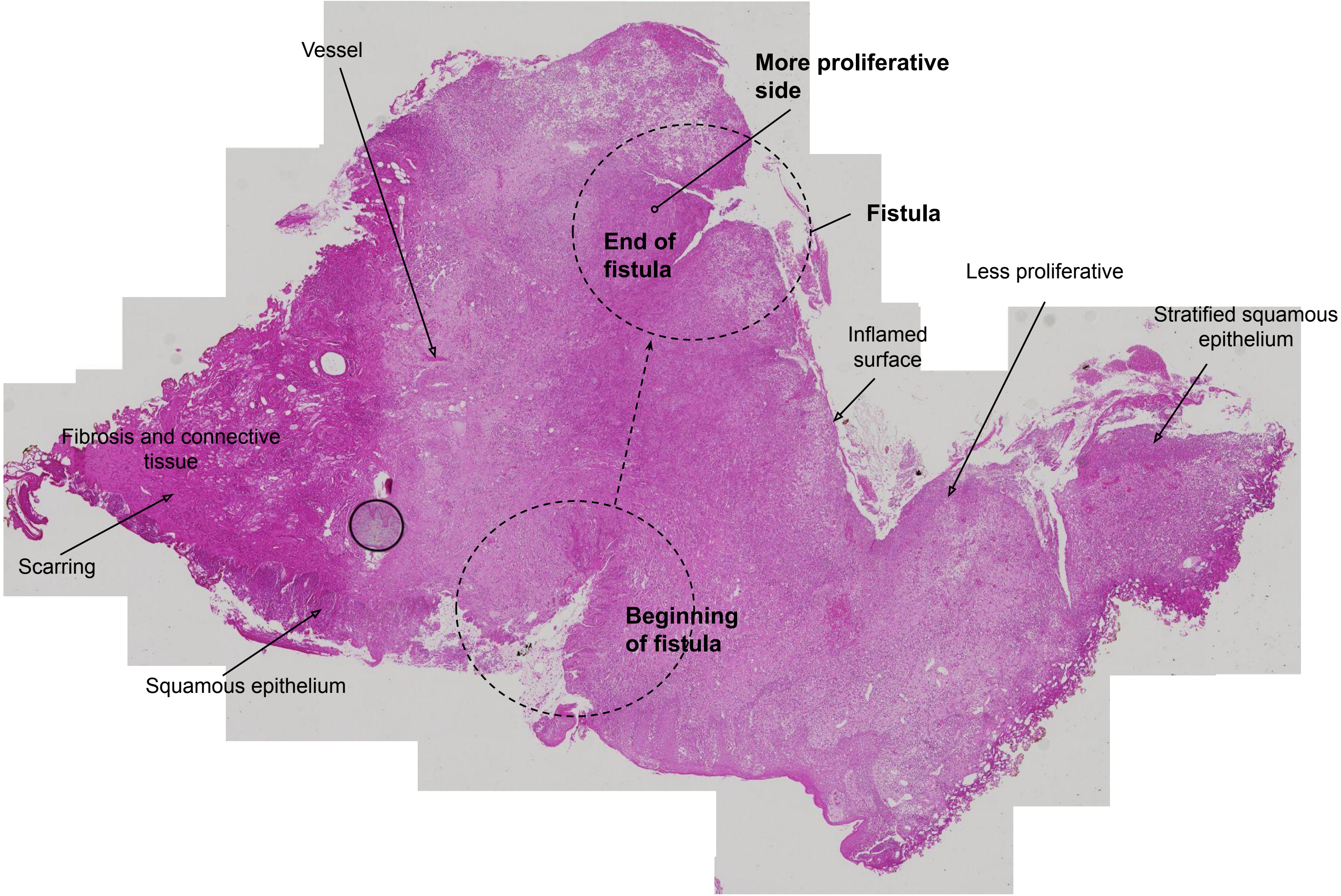

BAY_48715_20

ANAL CD FISTULA

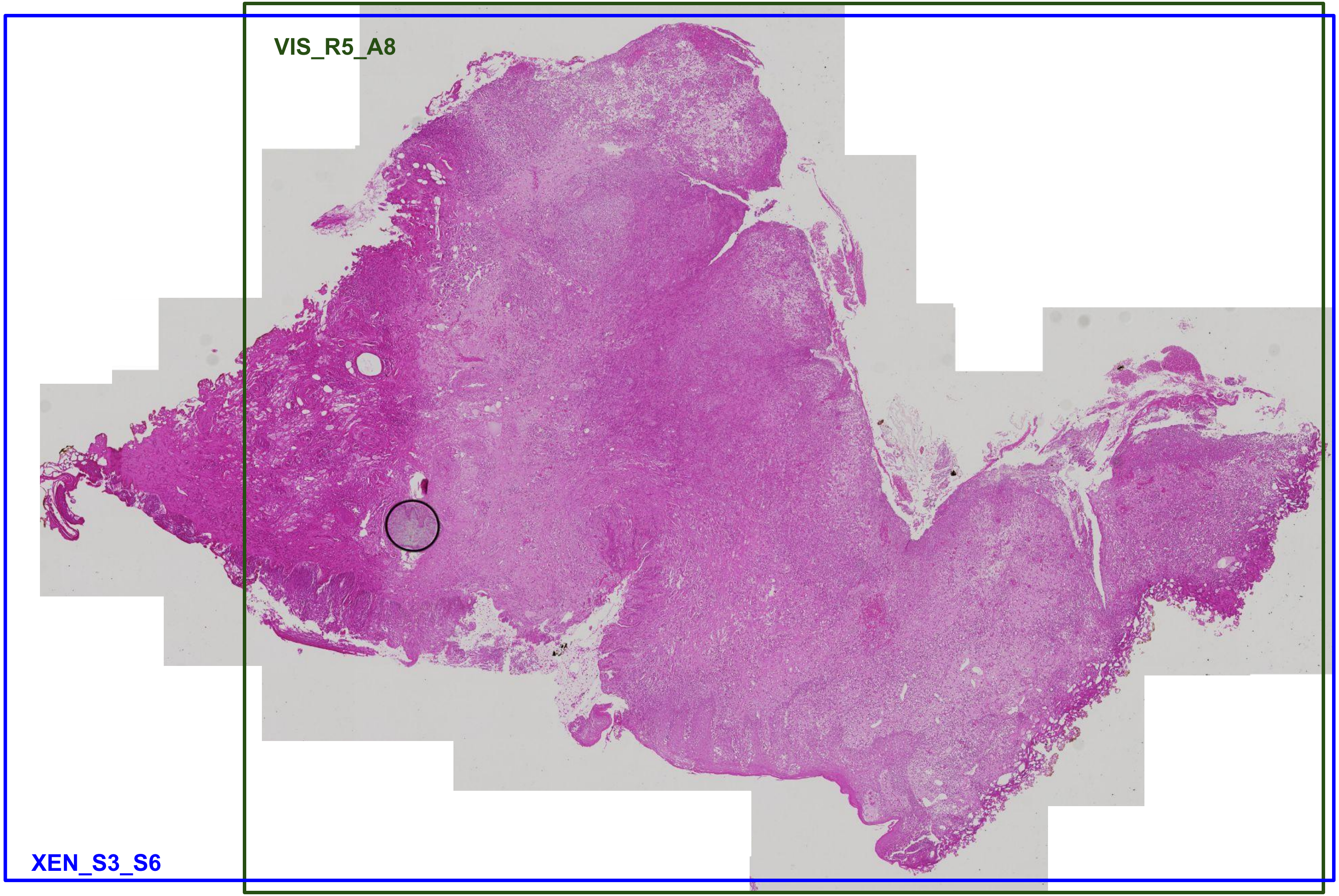

BAY_48715_20

ANAL CD FISTULA

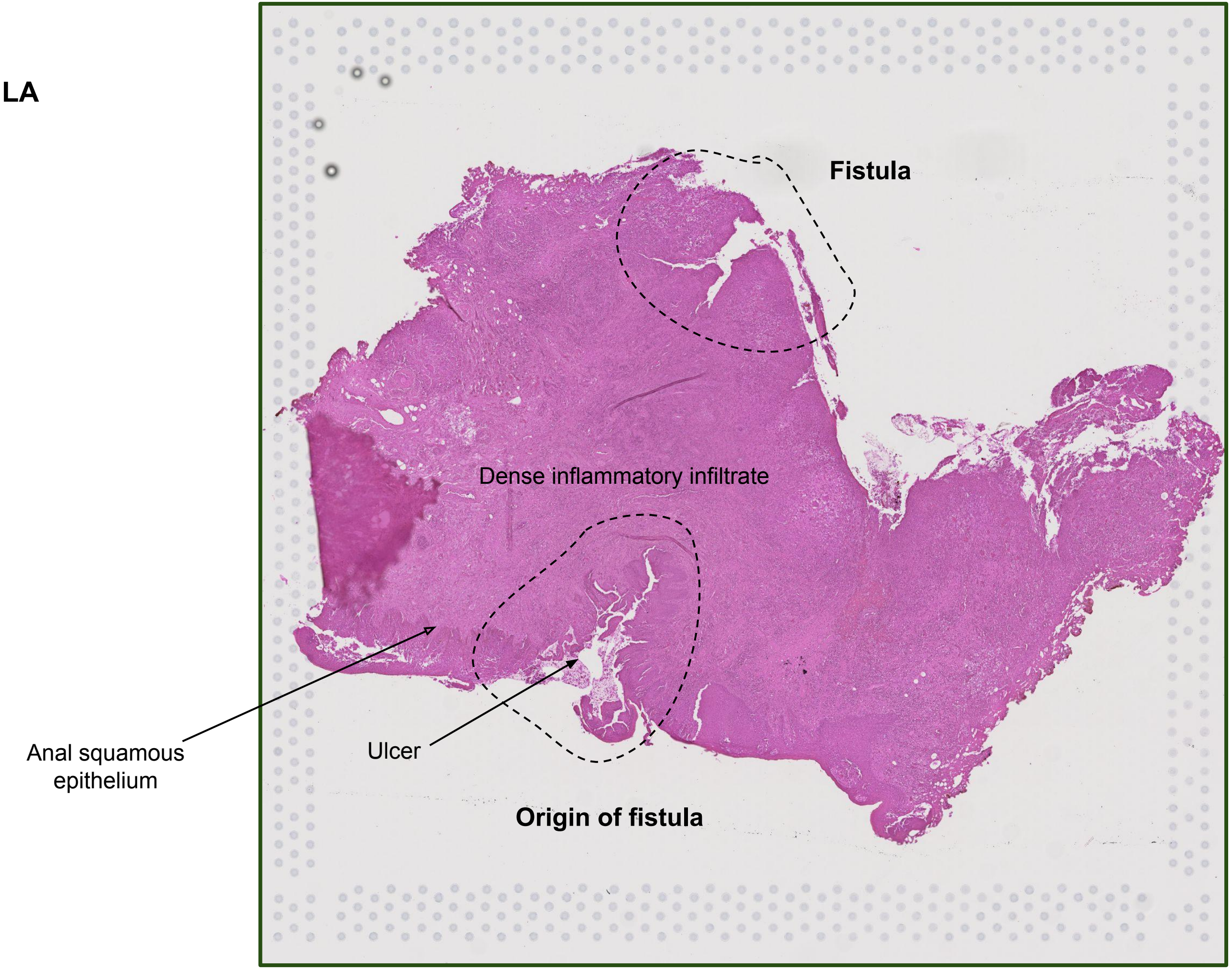

BAY_79501_12

SIGMOID-PERITONEAL CD FISTULA

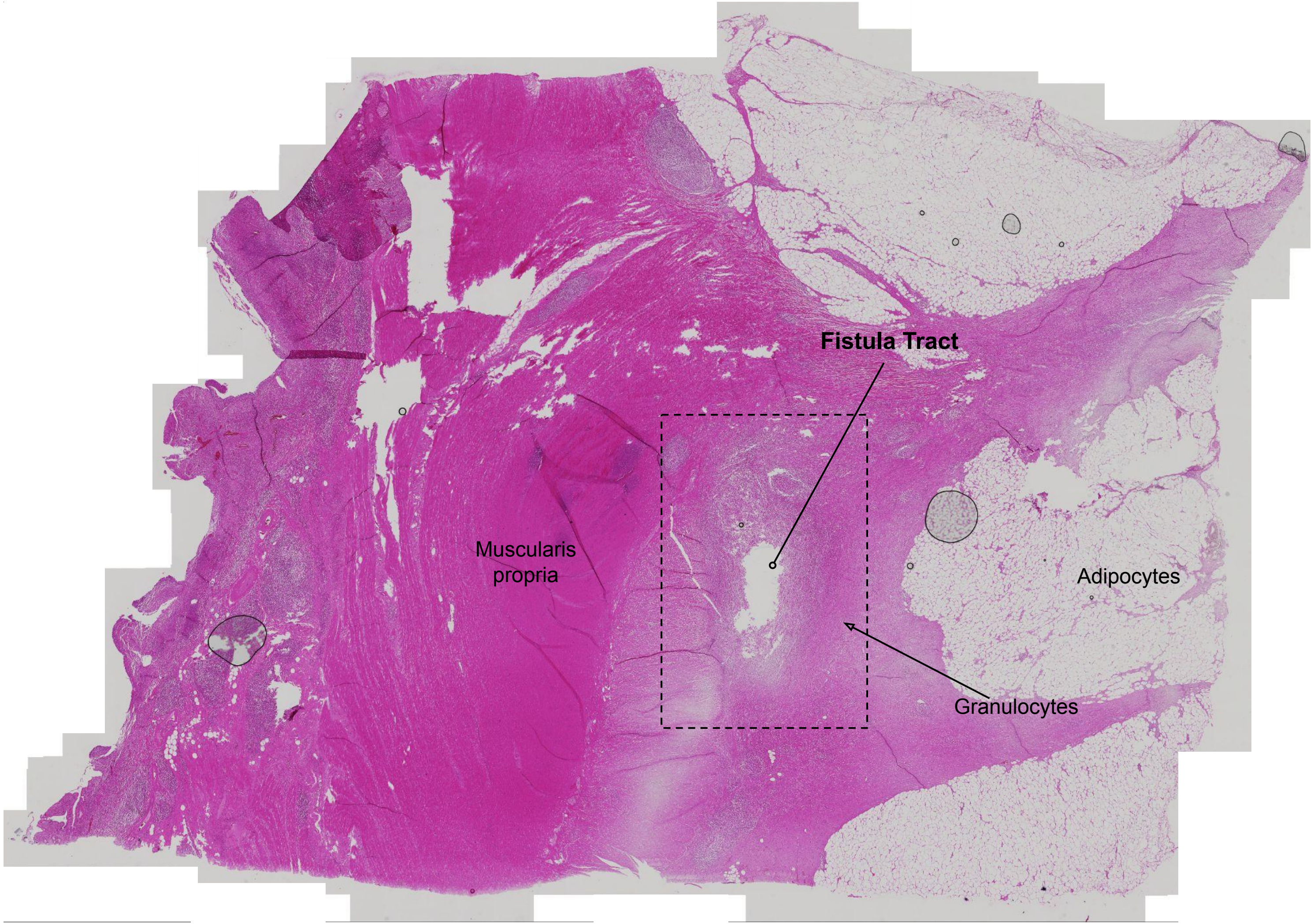

BAY_79501_12

SIGMOID-PERITONEAL
CD FISTULA

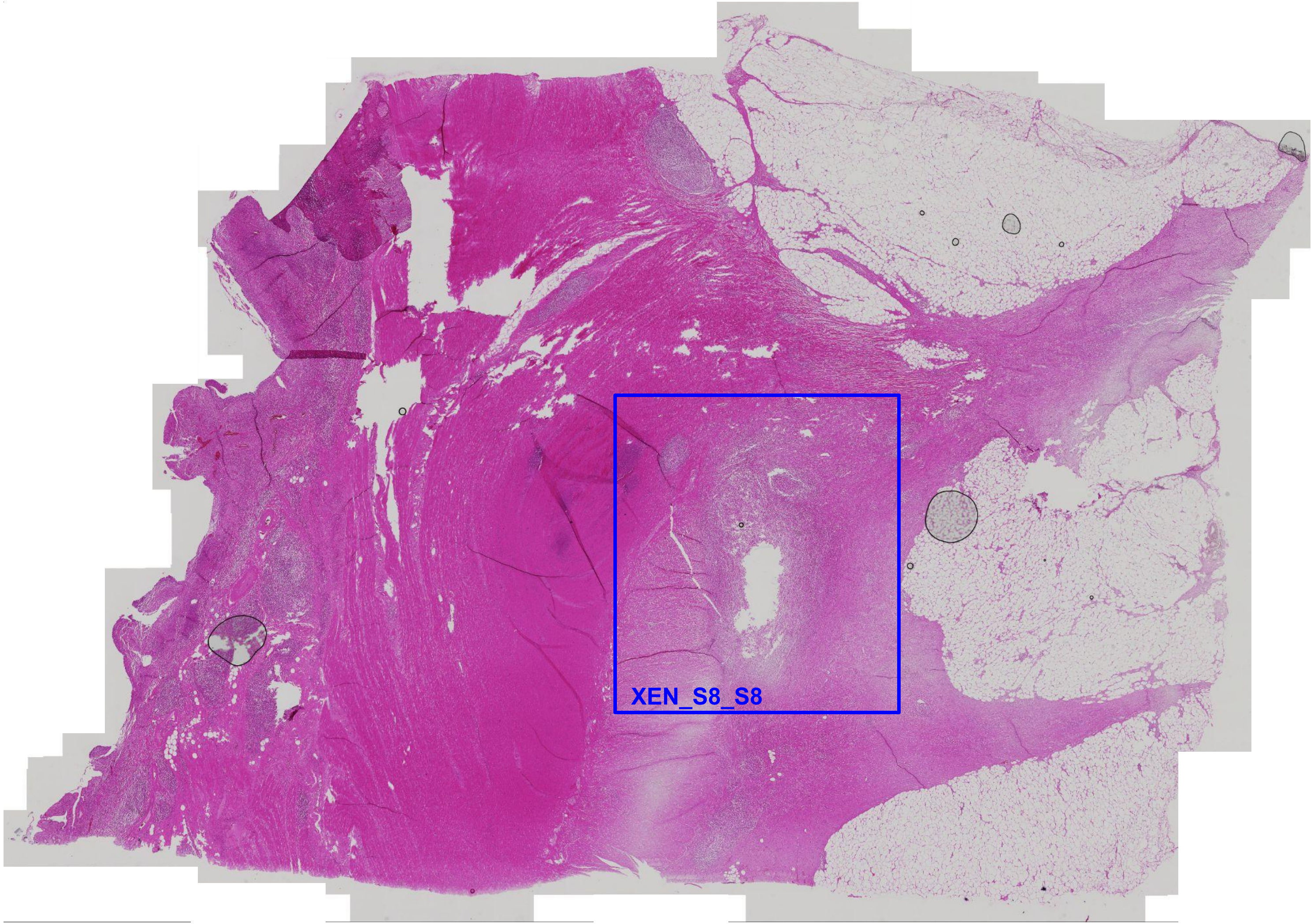

BAY_86068_17

COLONIC CD FISTULA

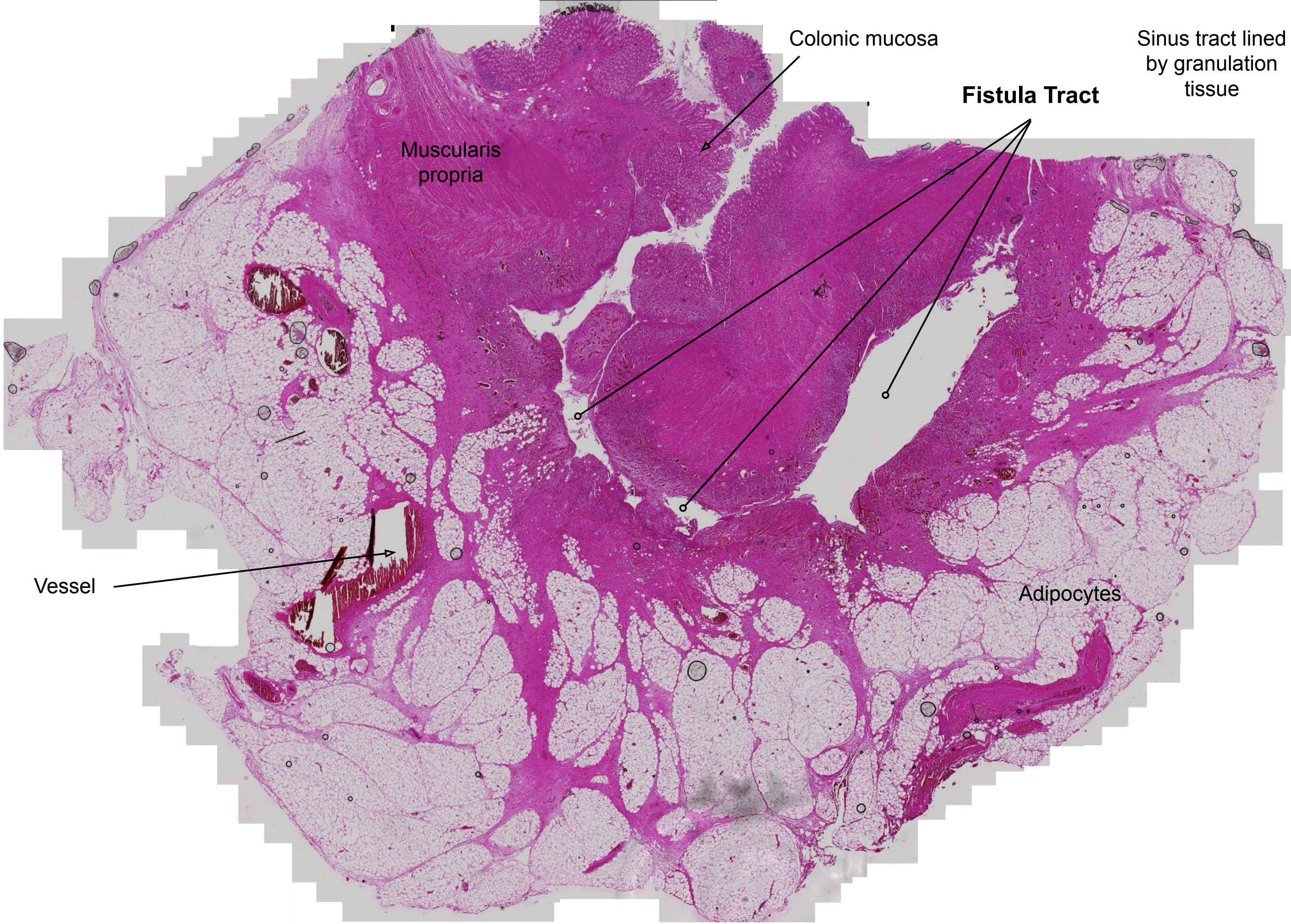

BAY_86068_17

COLONIC CD FISTULA

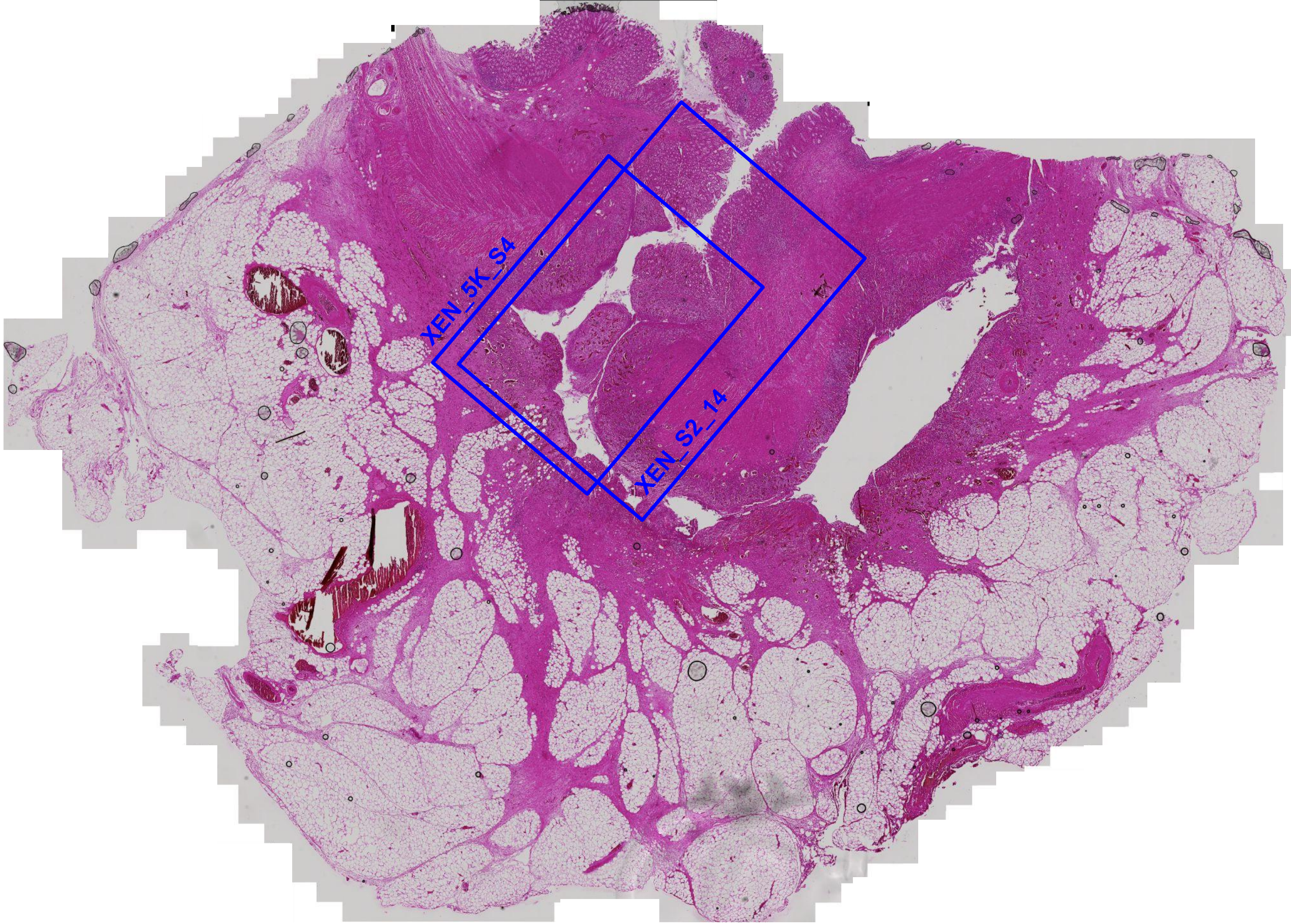

BAY_94350_21

ILEOCAECAL CD FISTULA

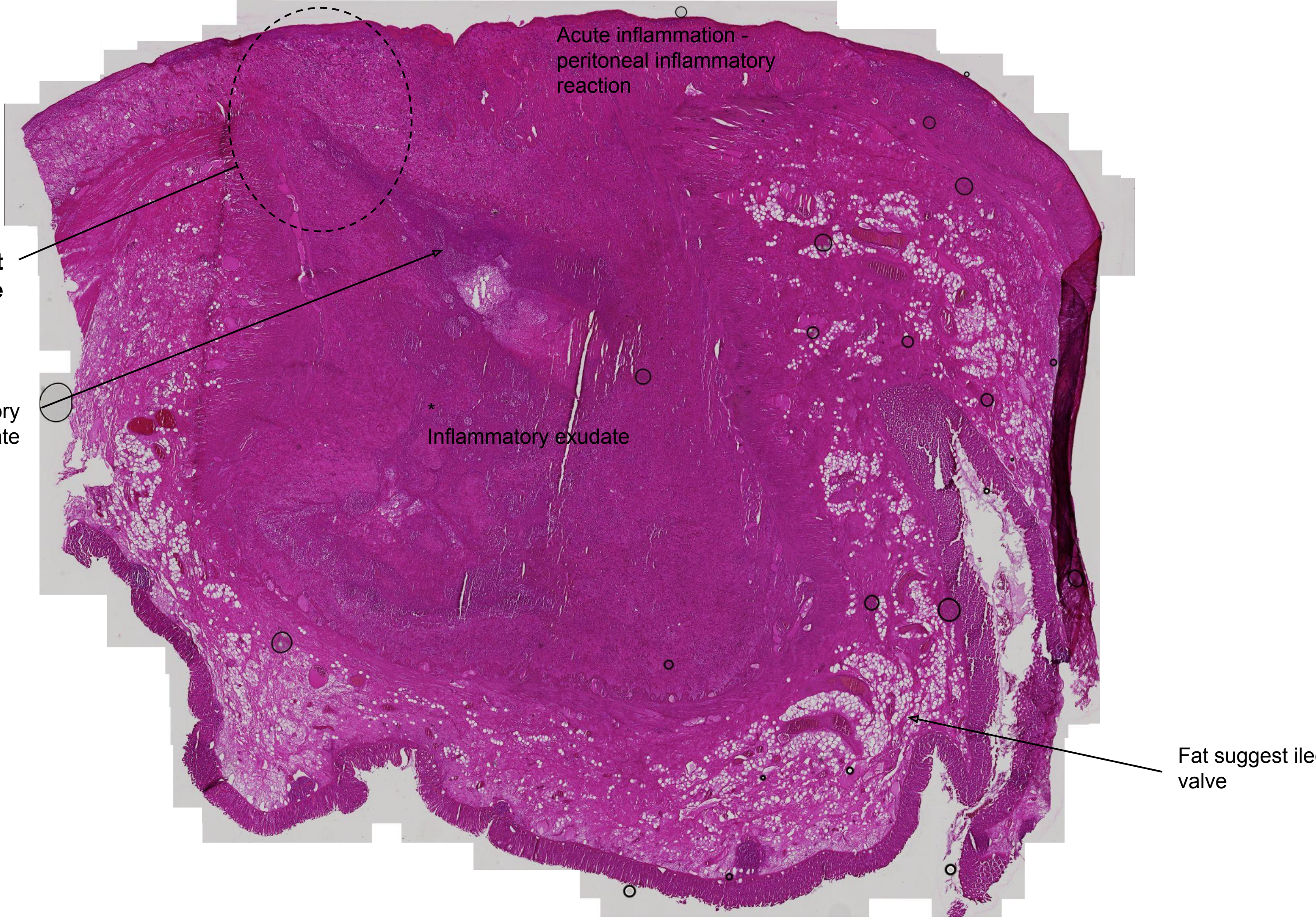

BAY_94350_21

ILEOCAECAL CD
FISTULA

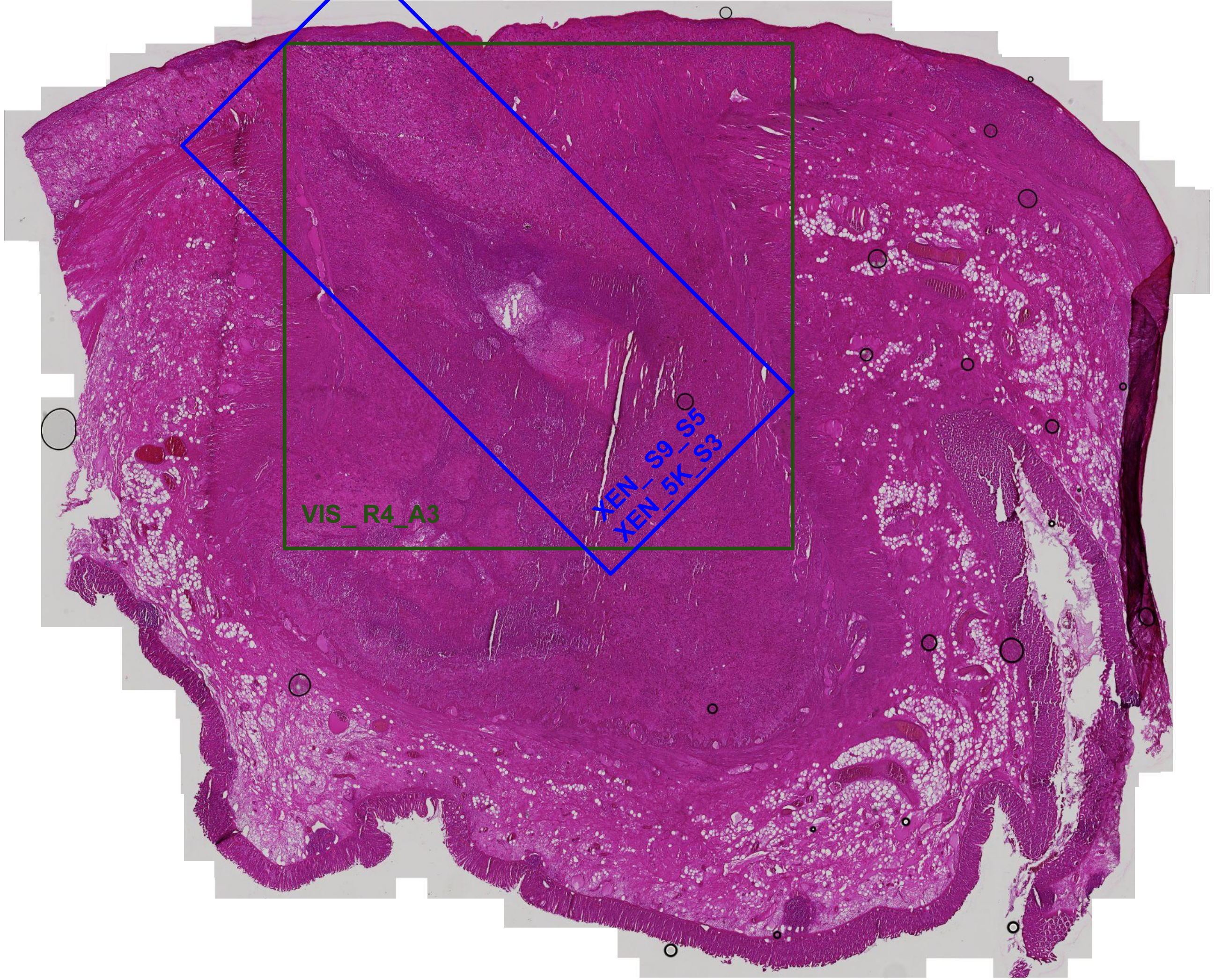

BAY_94350_21

ILEOCAECAL CD FISTULA

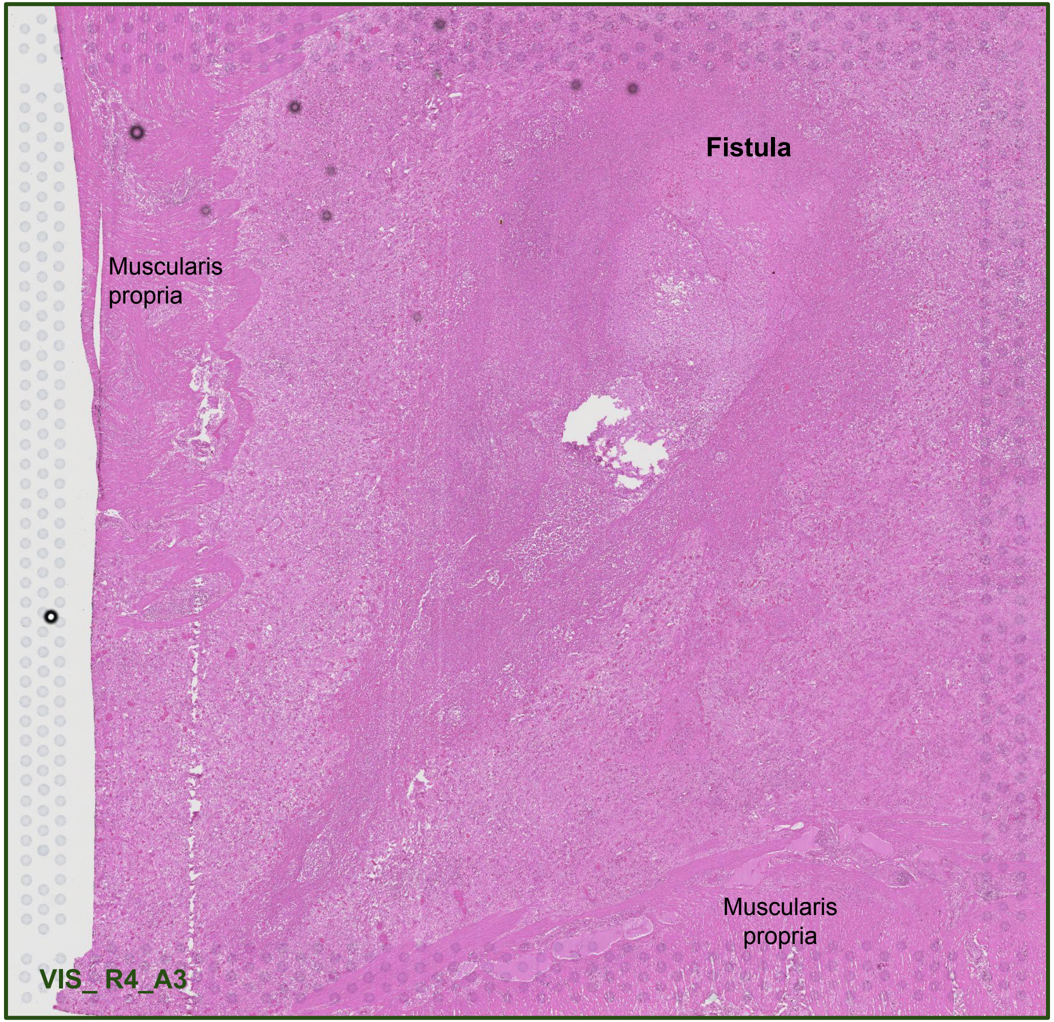

JR 43196_21

PERIANAL
CD FISTULA

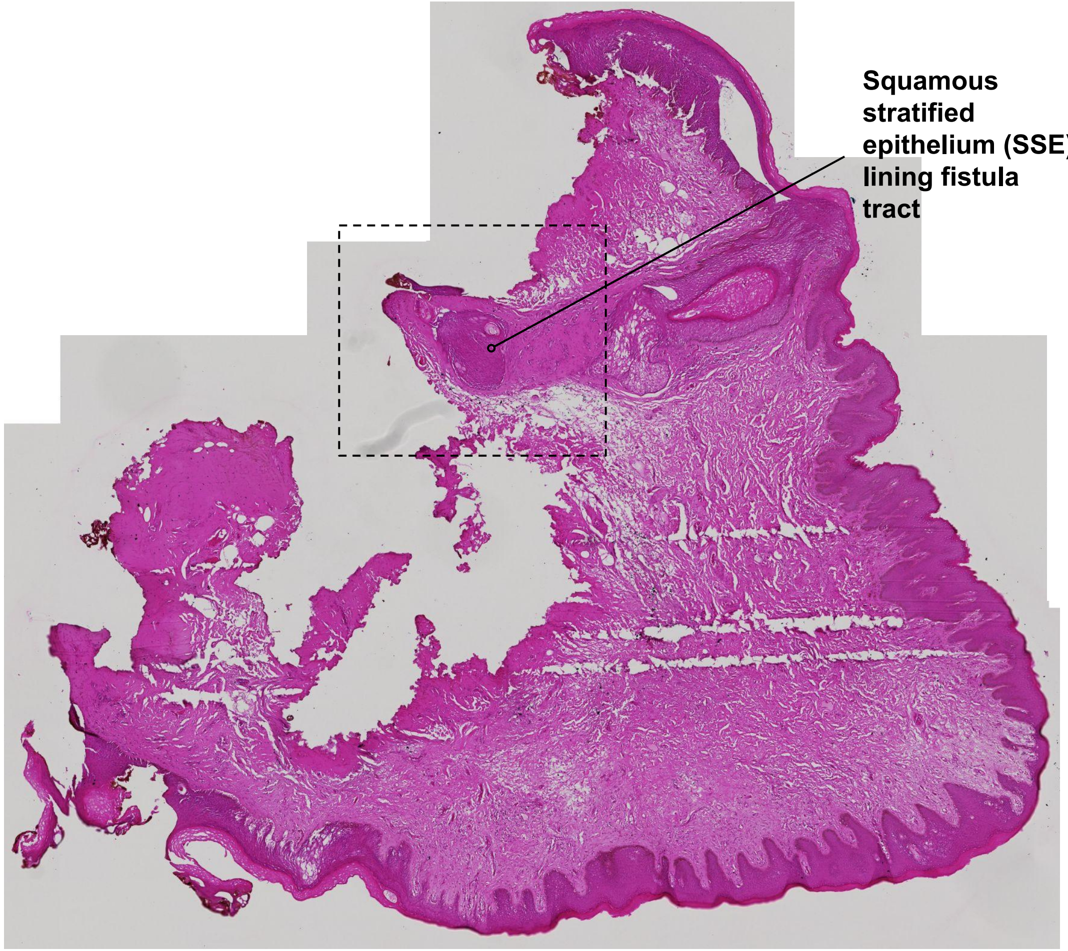

JR 43196_21

PERIANAL
CD FISTULA

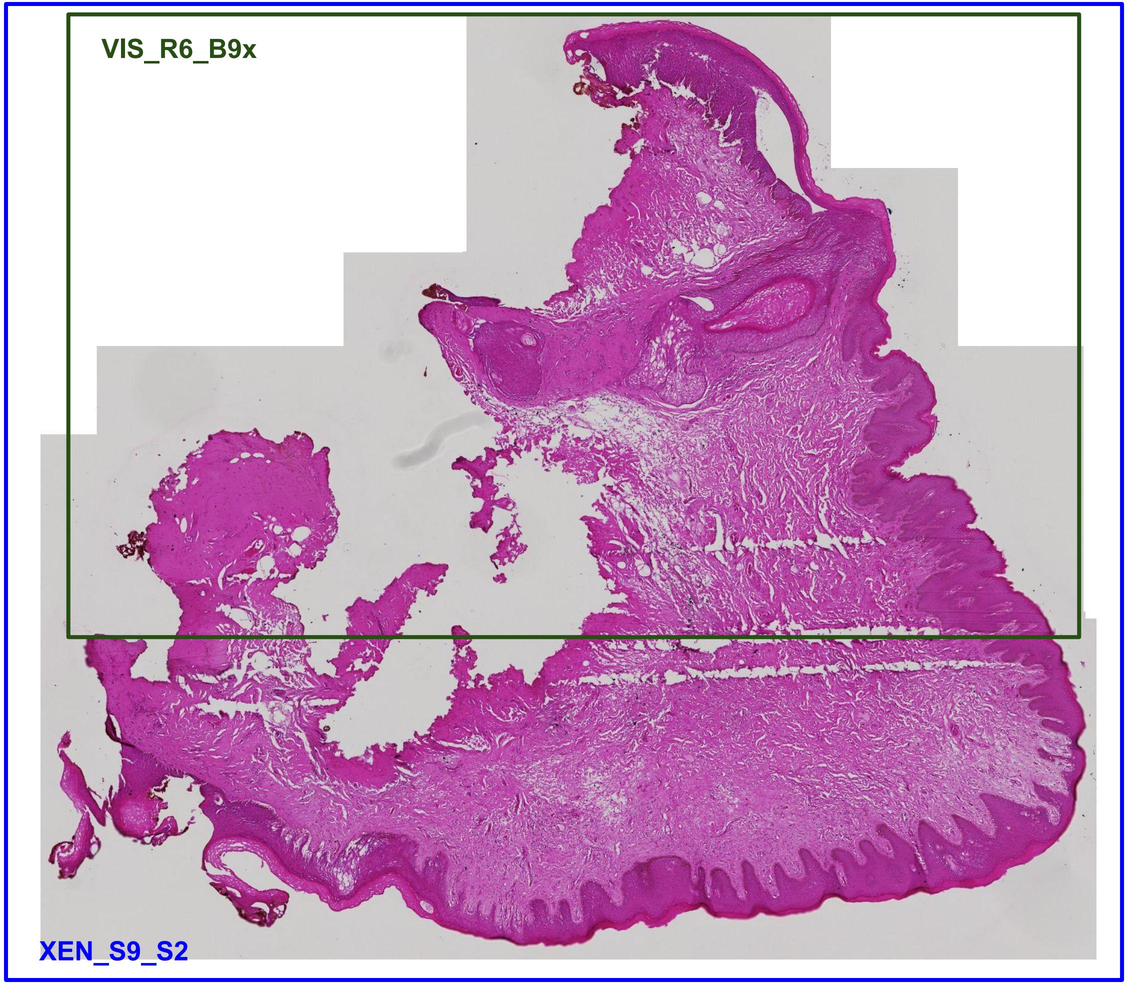

JR 43196_21

PERIANAL
CD FISTULA

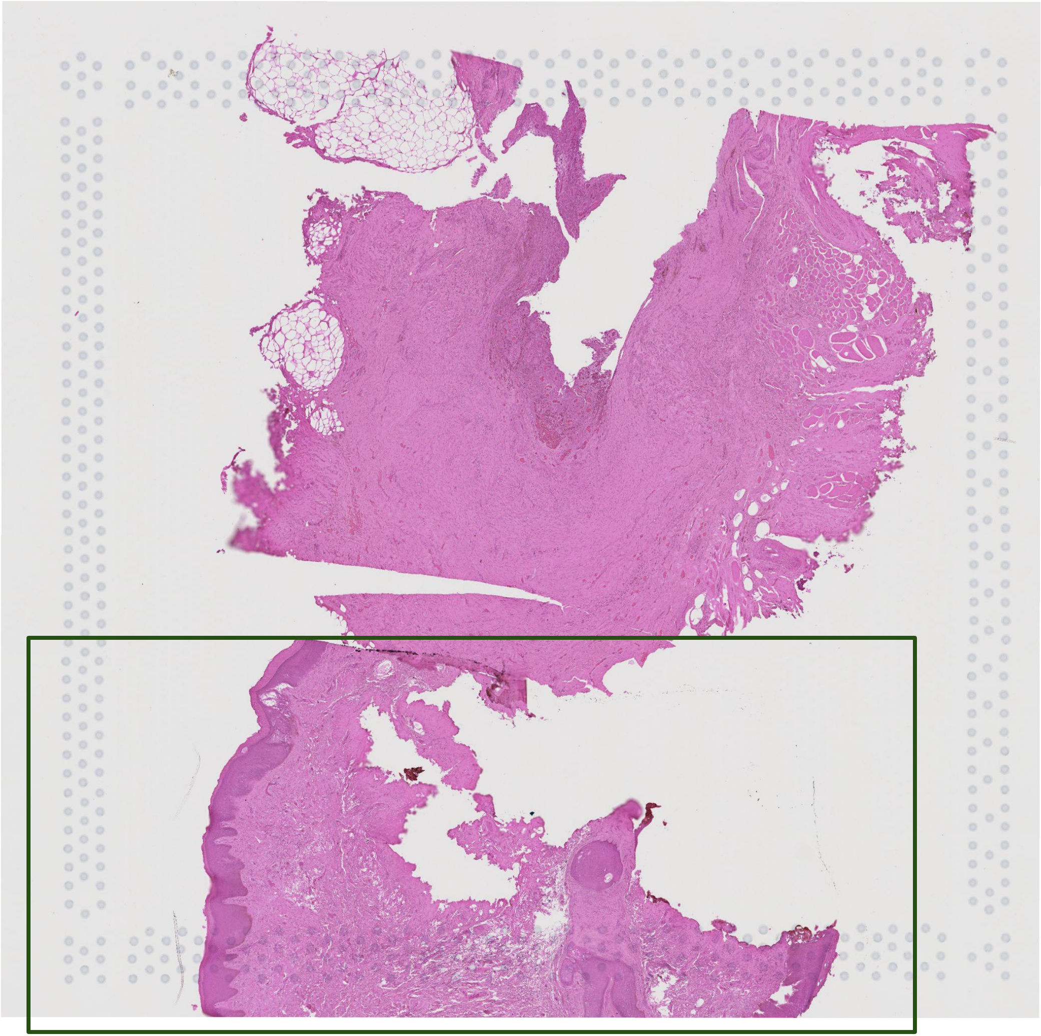

JR_17451_20

ILEOSIGMOID CD FISTULA

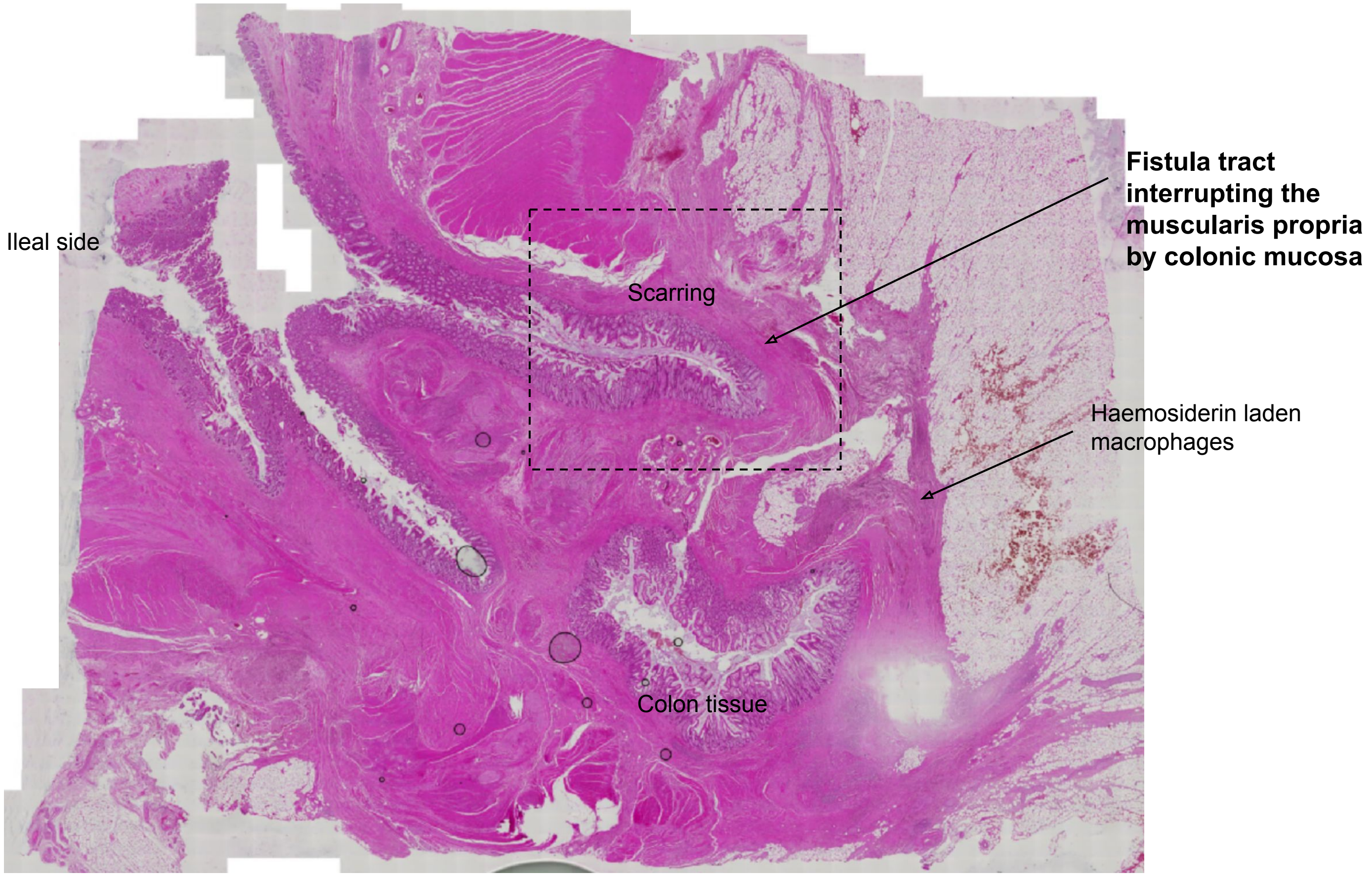

JR_17451_20

ILEOSIGMOID CD
FISTULA

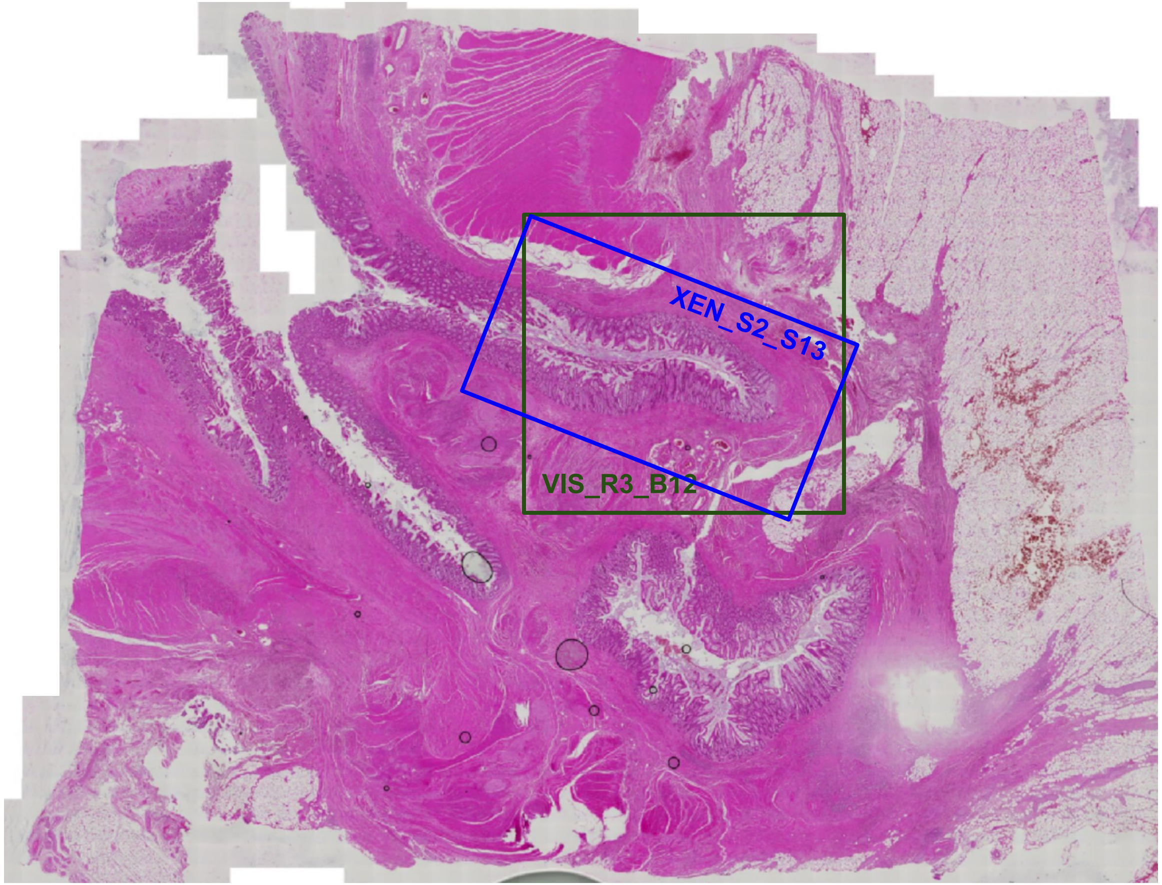

JR_17451_20

ILEOSIGMOID CD
FISTULA

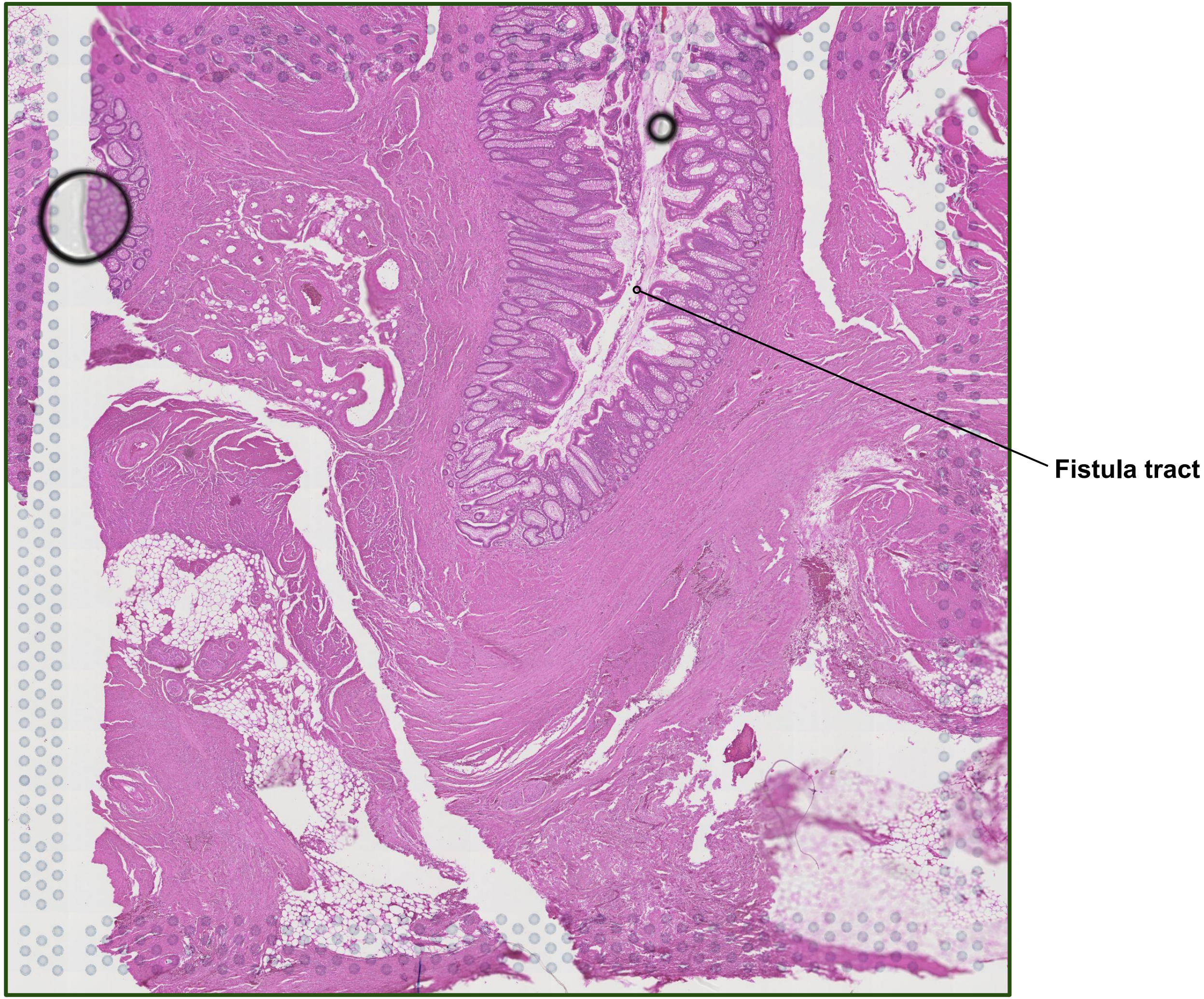

JR_18076_22

ANAL CD
FISTULA

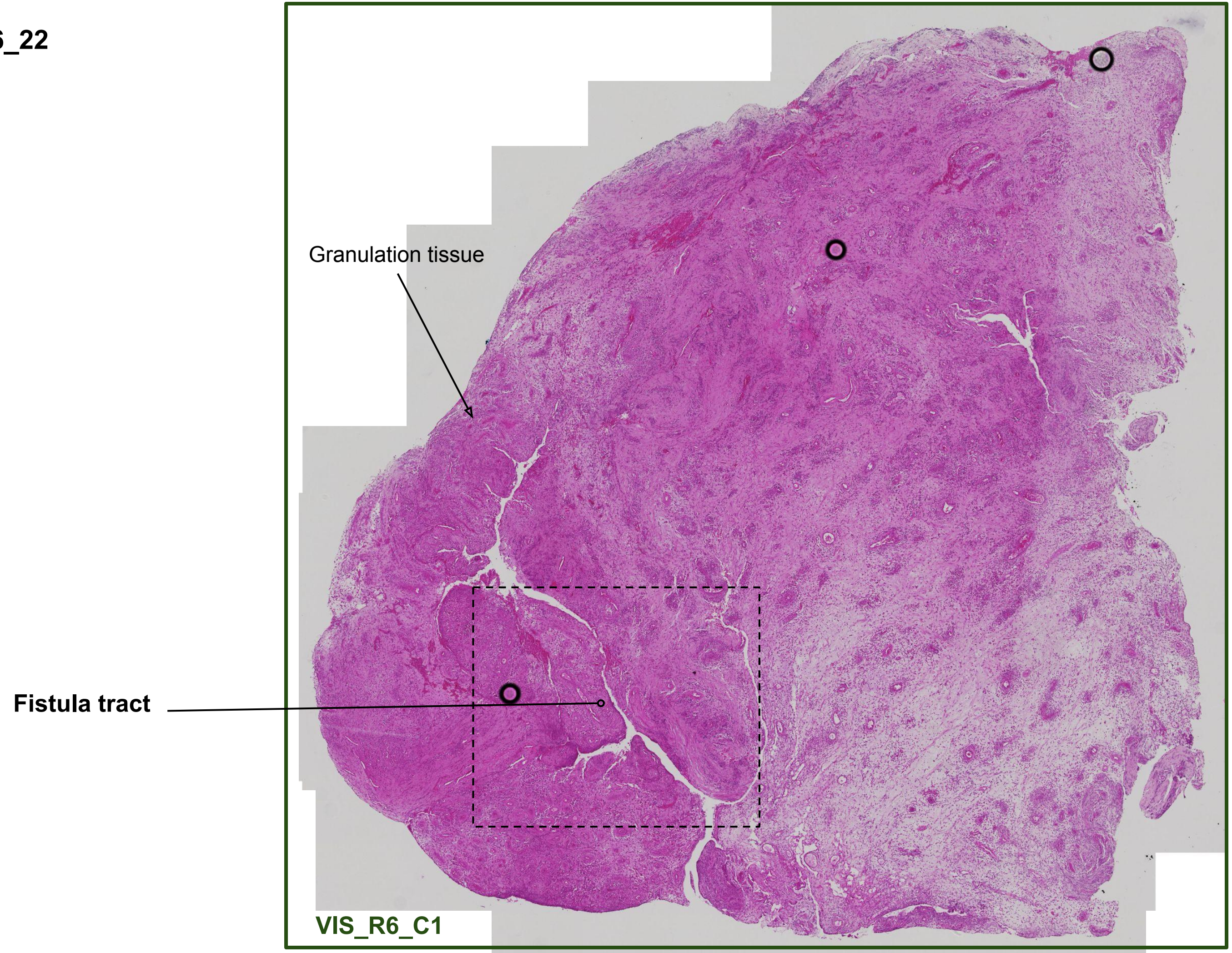

JR_18076_22

ANAL CD
FISTULA

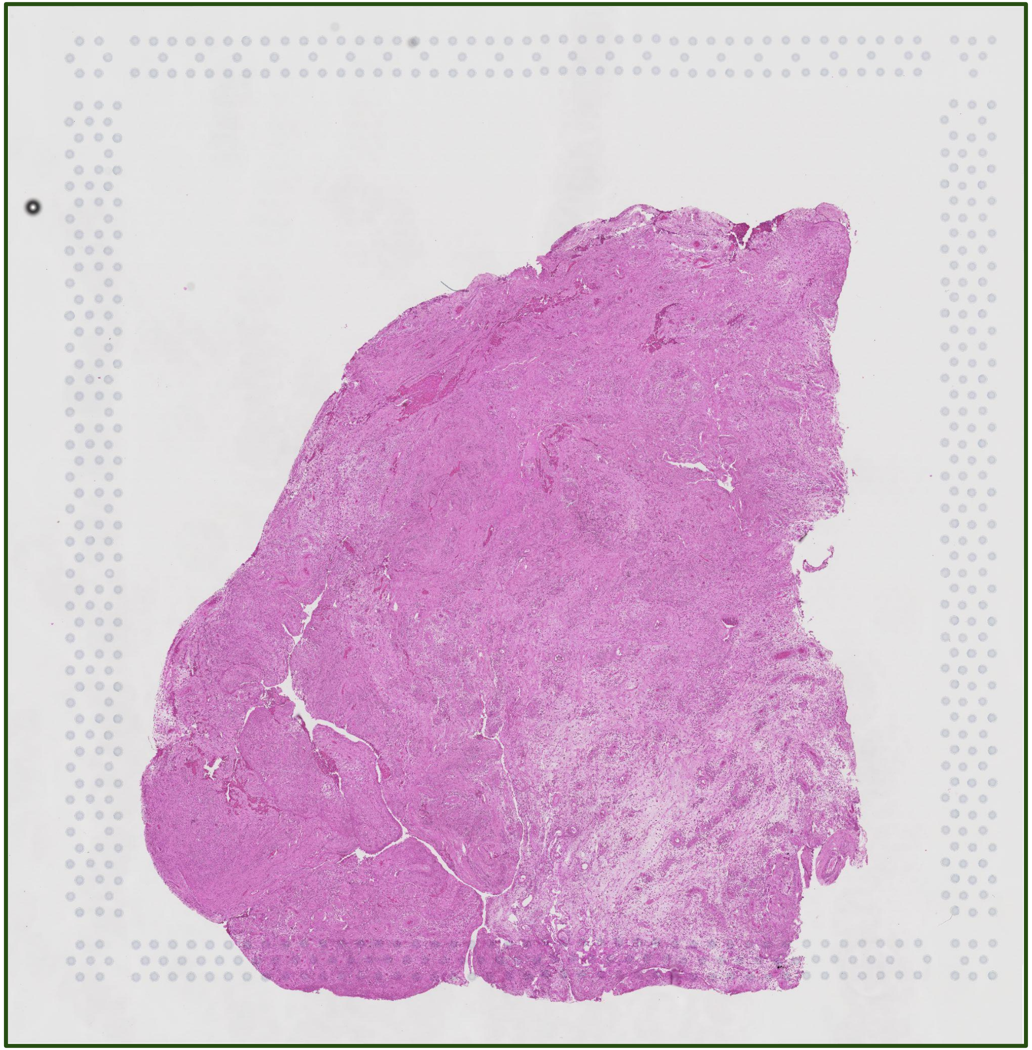

JR_20291_22

PERIANAL CD FISTULA

Granulation tissue with inflammation. Granulation tissue lining fistula tract (running in multiple directions)

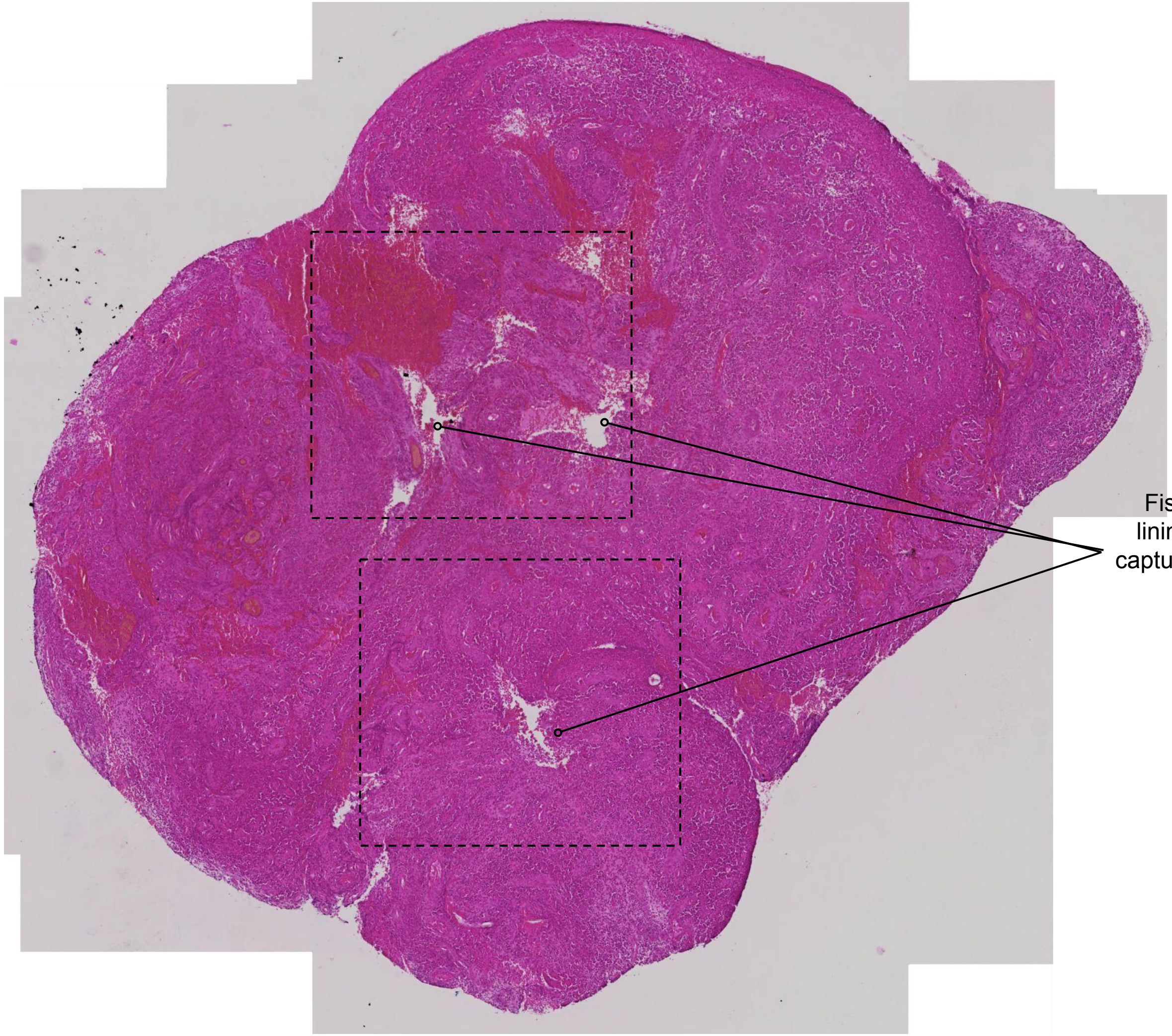

Fistula curetting - lining of the fistula captured but not the architecture

JR_20291_22

PERIANAL CD
FISTULA

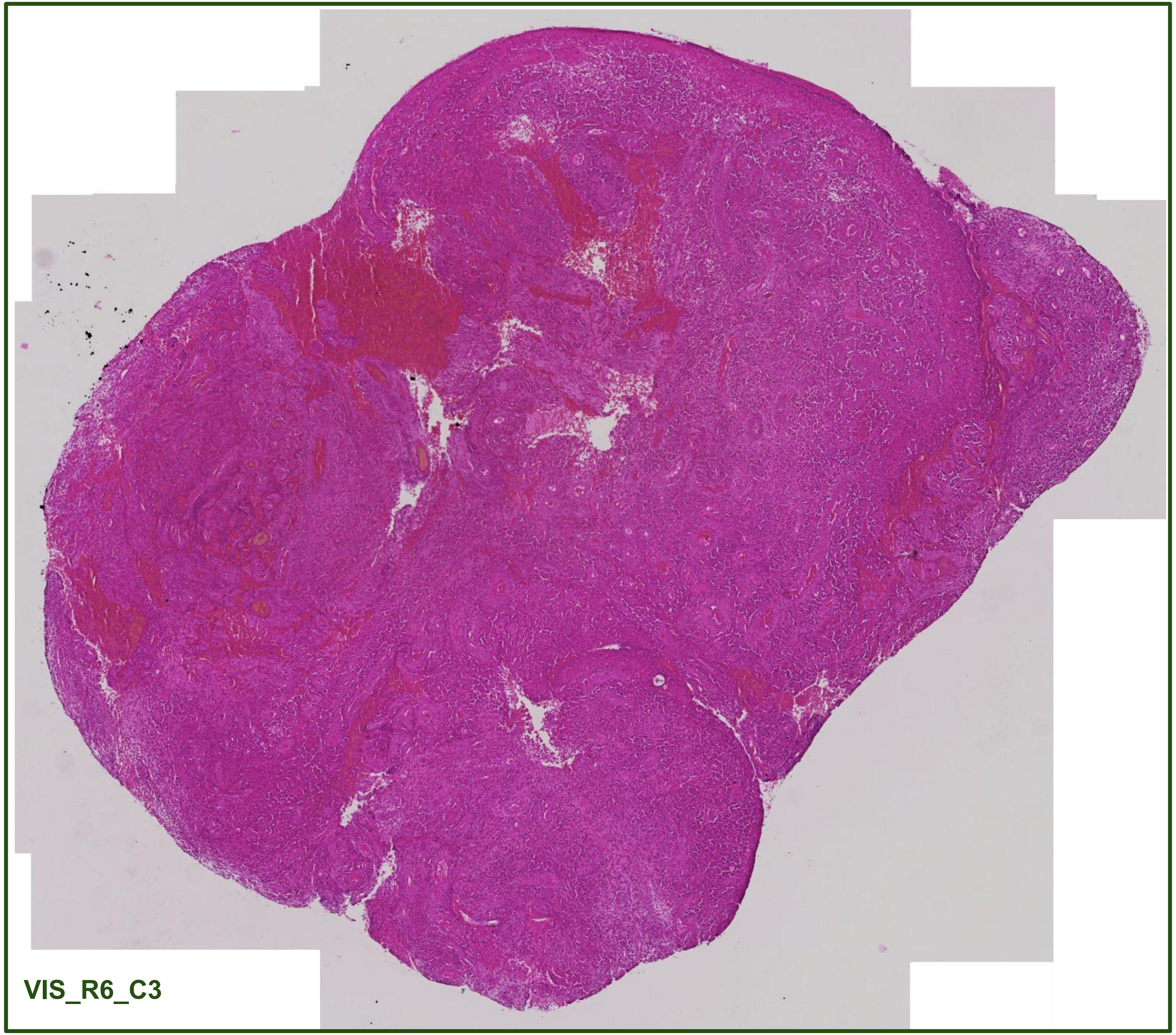

JR_20291_22

PERIANAL CD
FISTULA

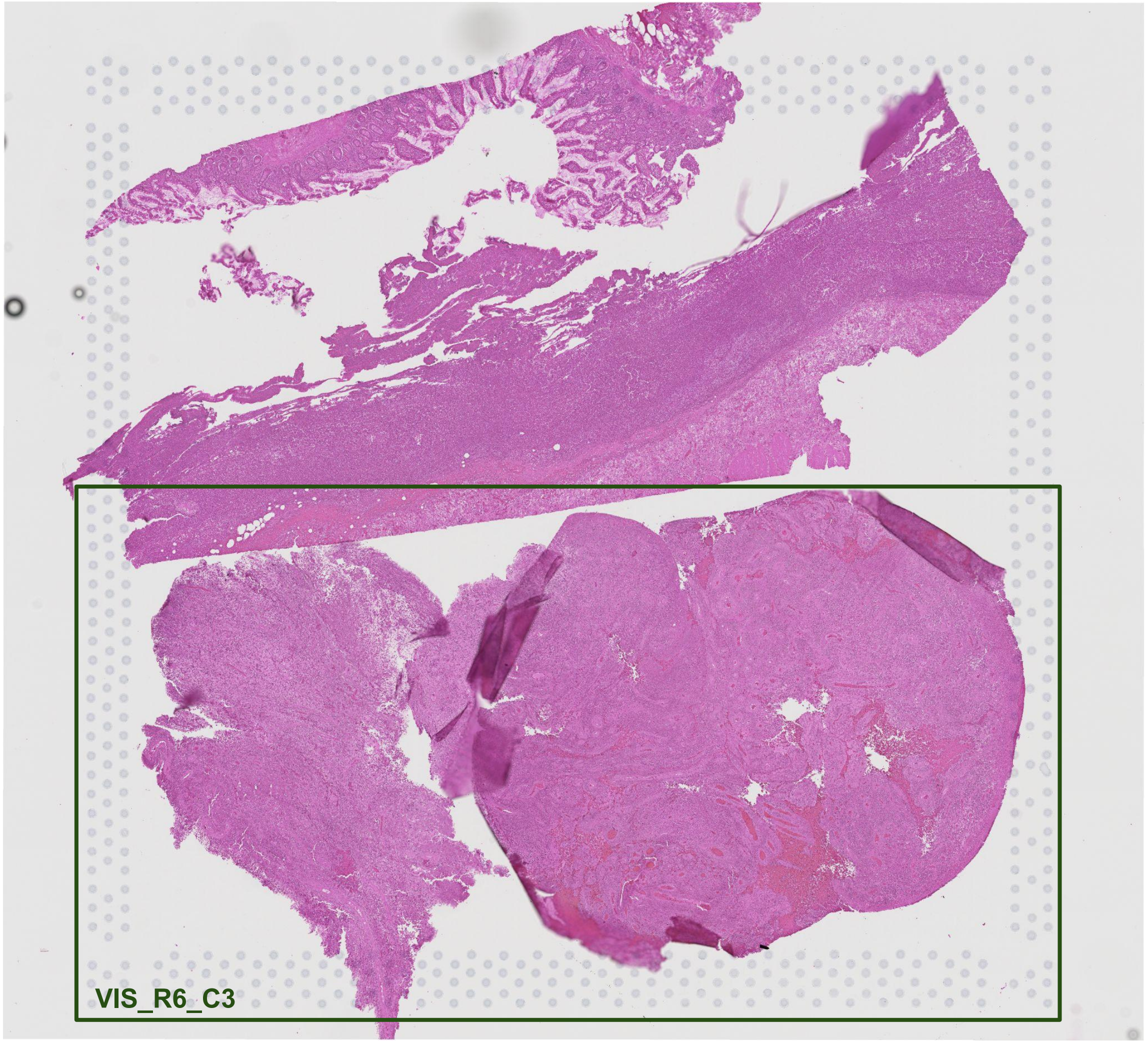

JR_22046_20

ILEOCOLIC CD FISTULA

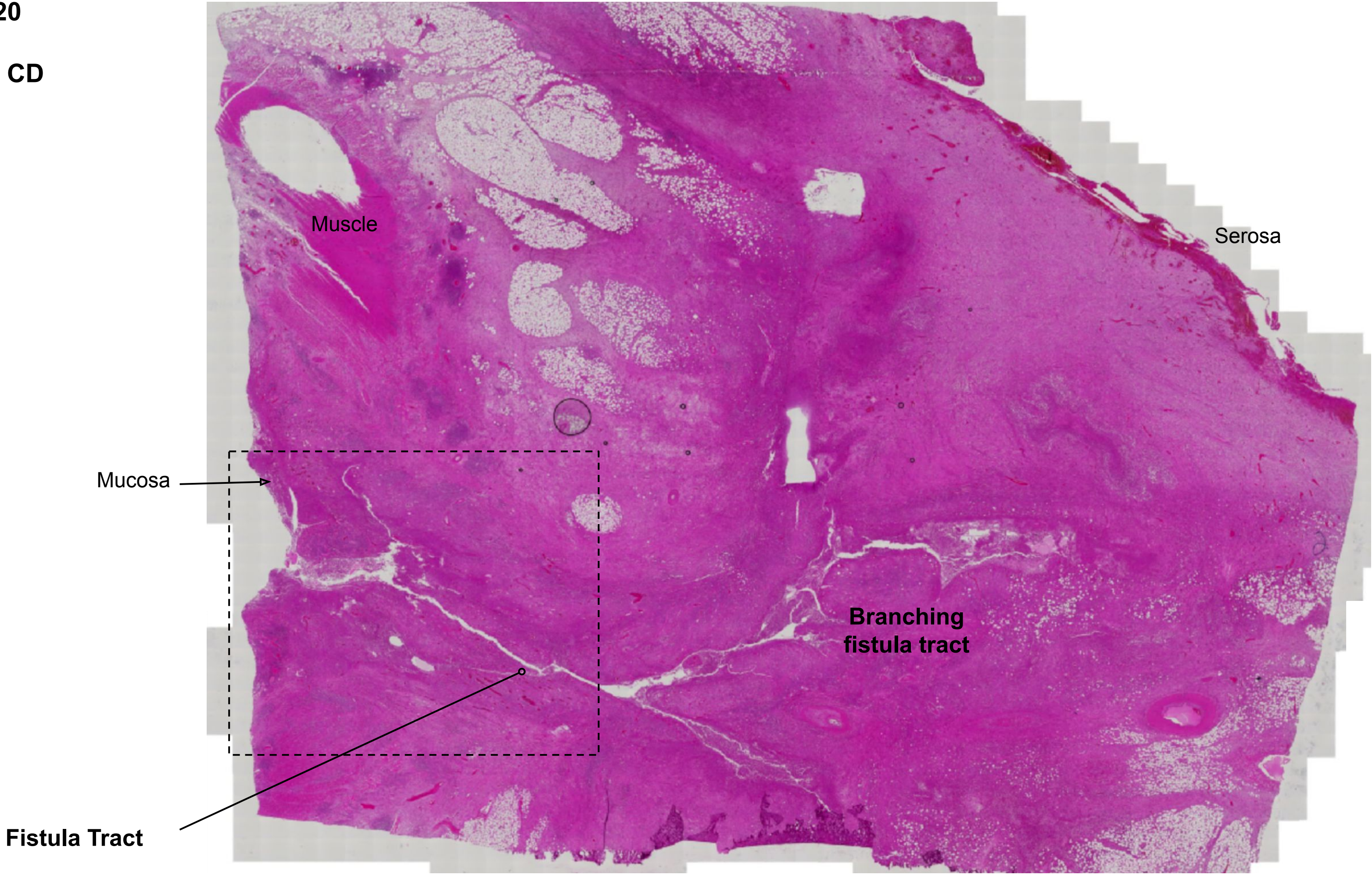

JR_22046_20

ILEOCOLIC CD
FISTULA

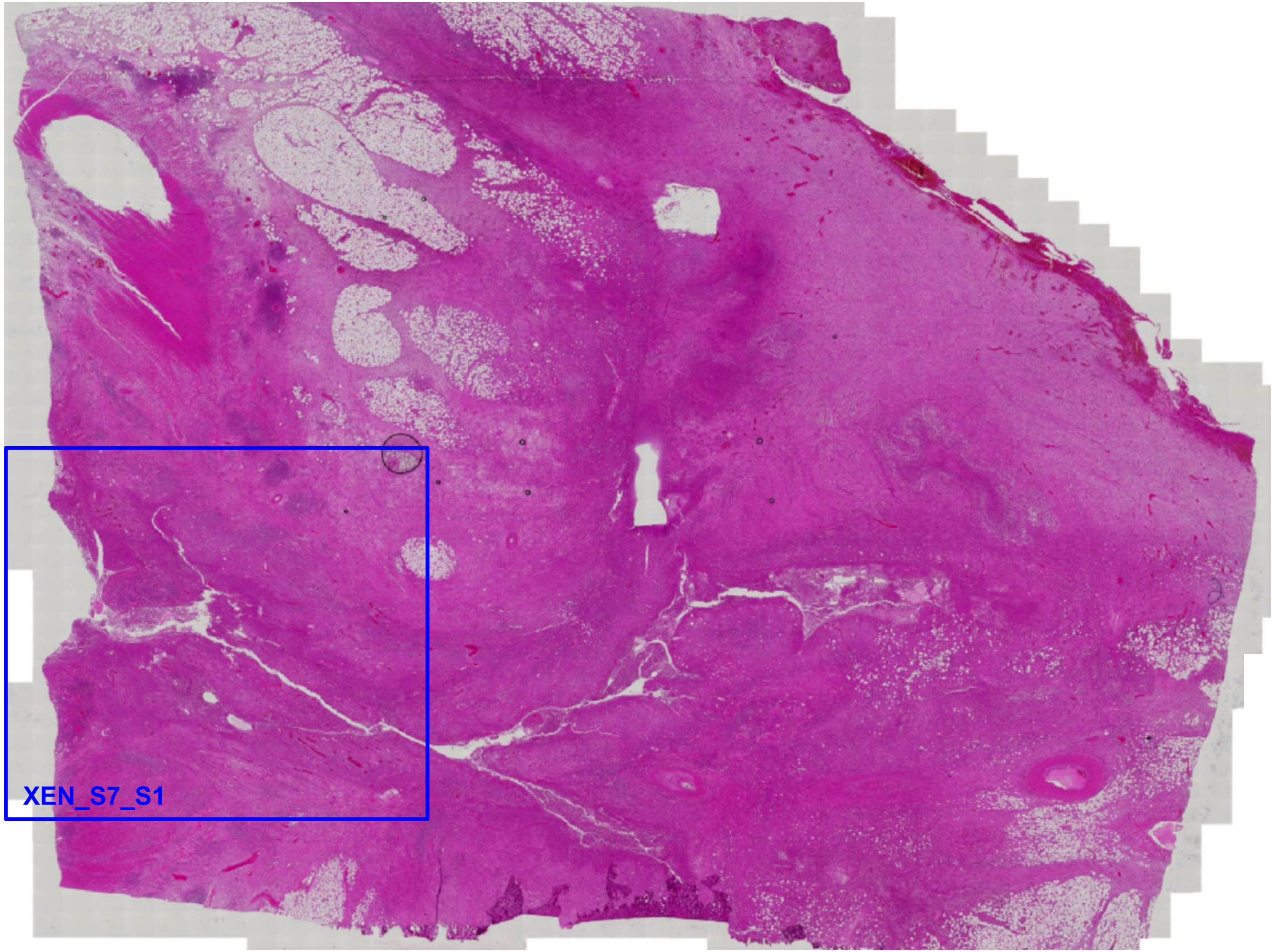

JR_23234_23

ENTEROCUTANEOUS CD FISTULA

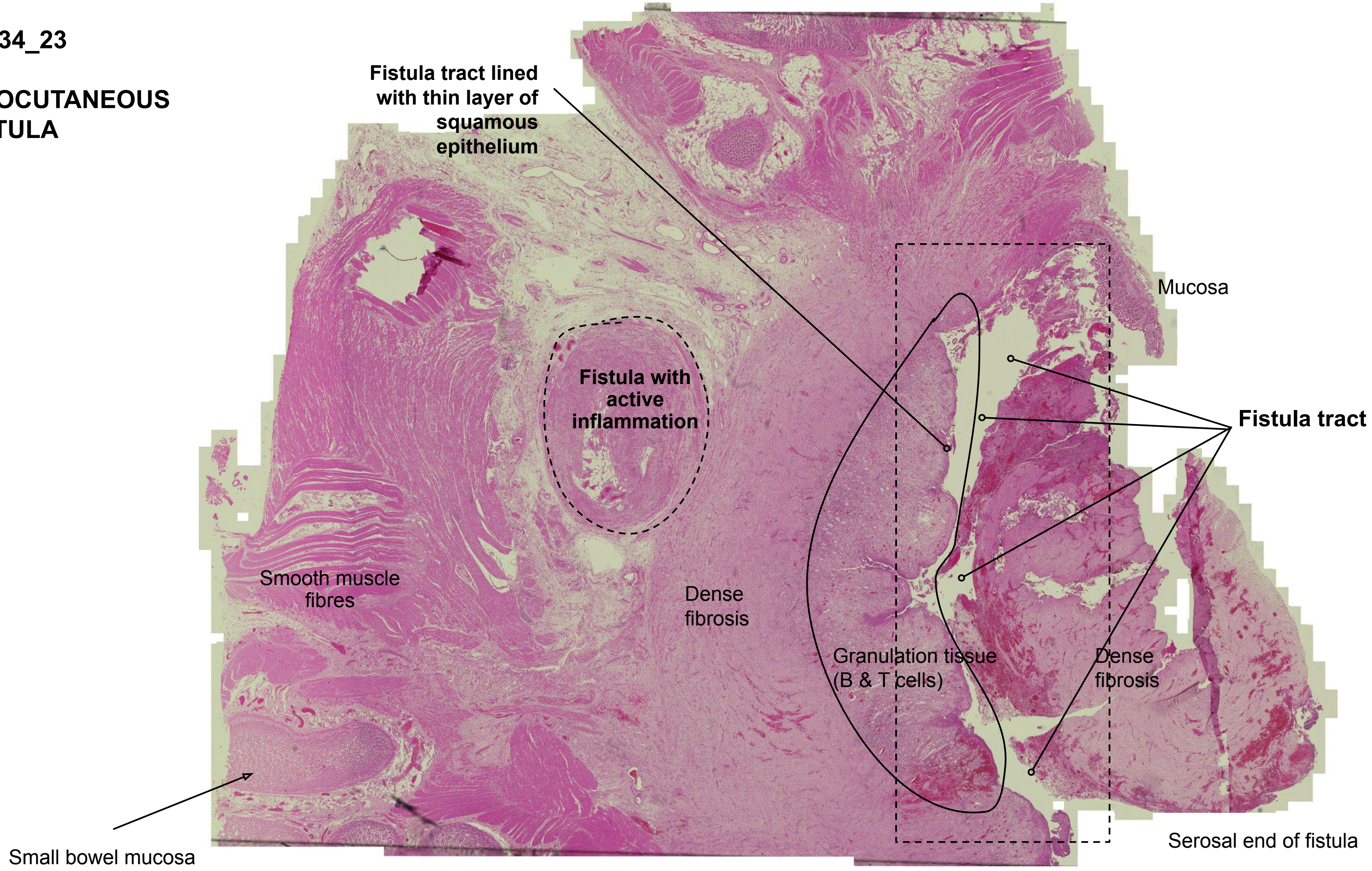

JR_23234_23

ENTEROCUTANEOUS
CD FISTULA

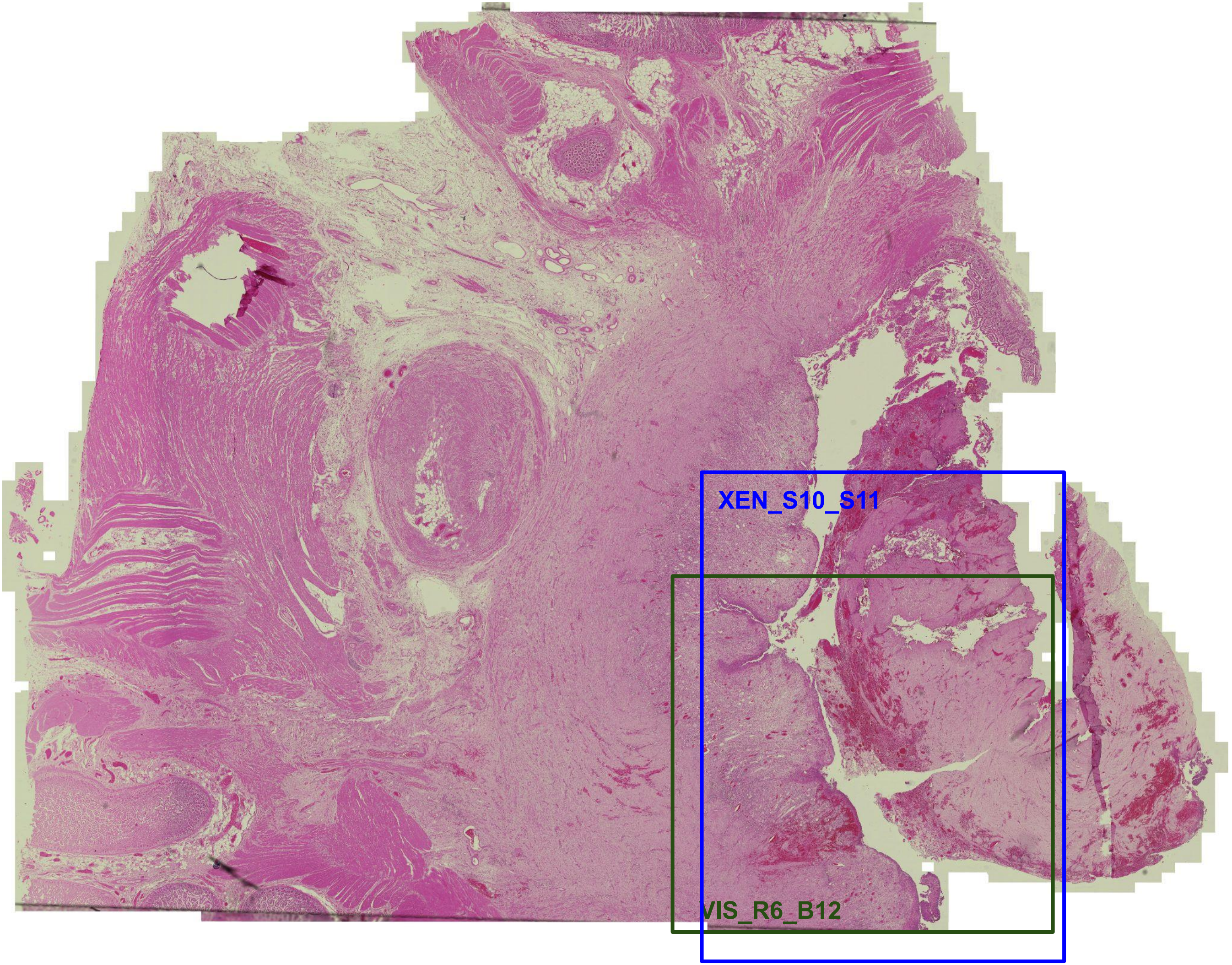

JR_23234_23

ENTEROCUTANEOUS
CD FISTULA

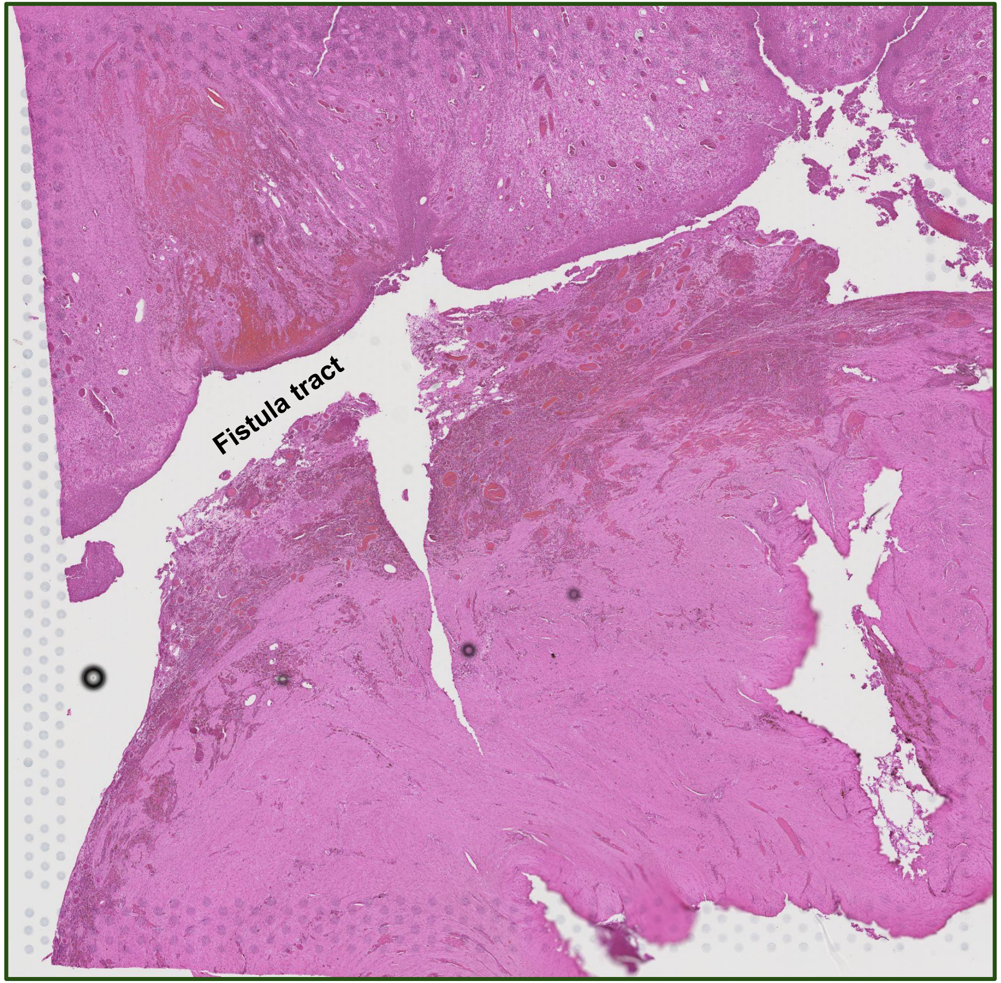

JR_31183_22

ILEAL CD FISTULA

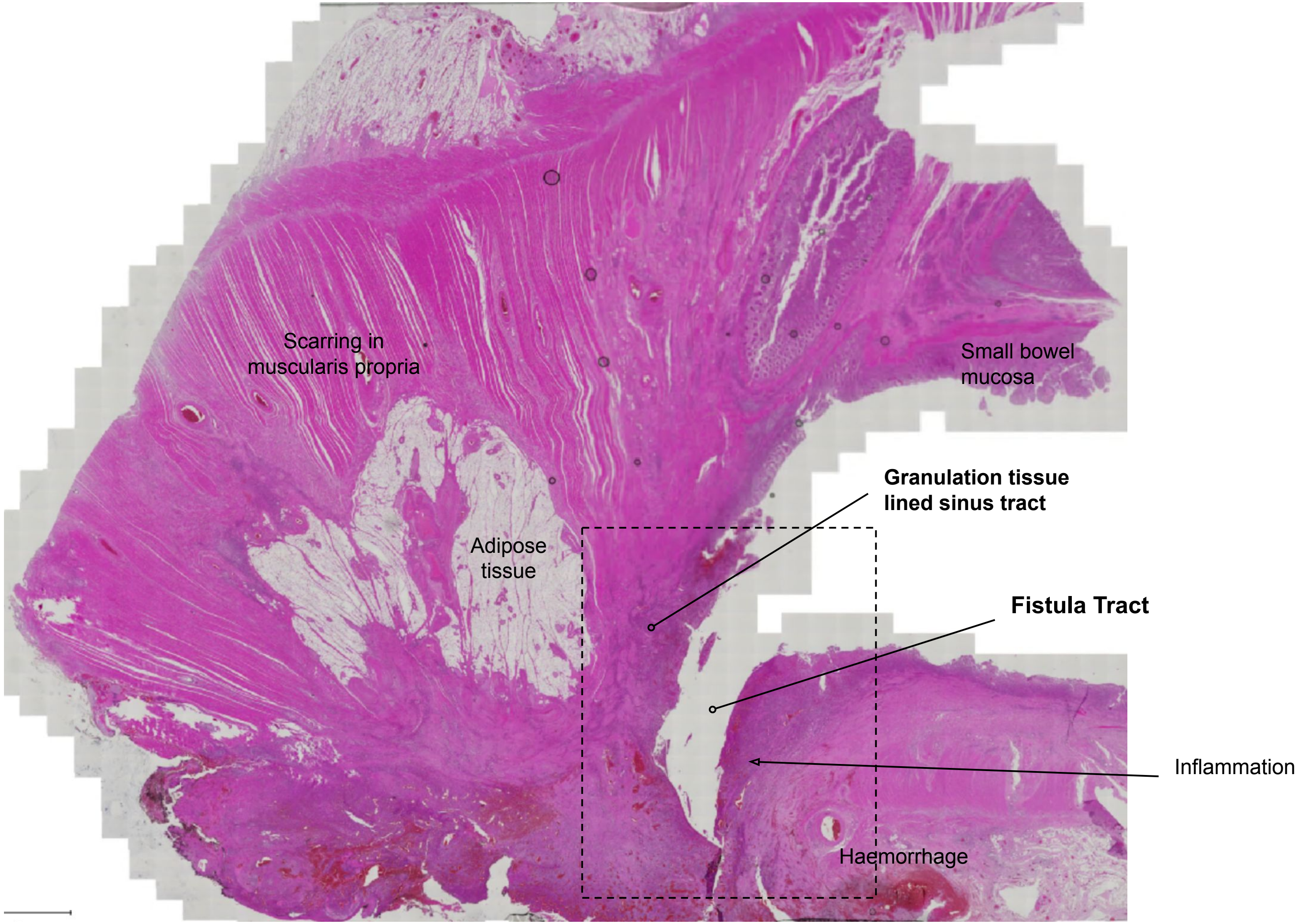

JR_31183_22

ILEAL CD FISTULA

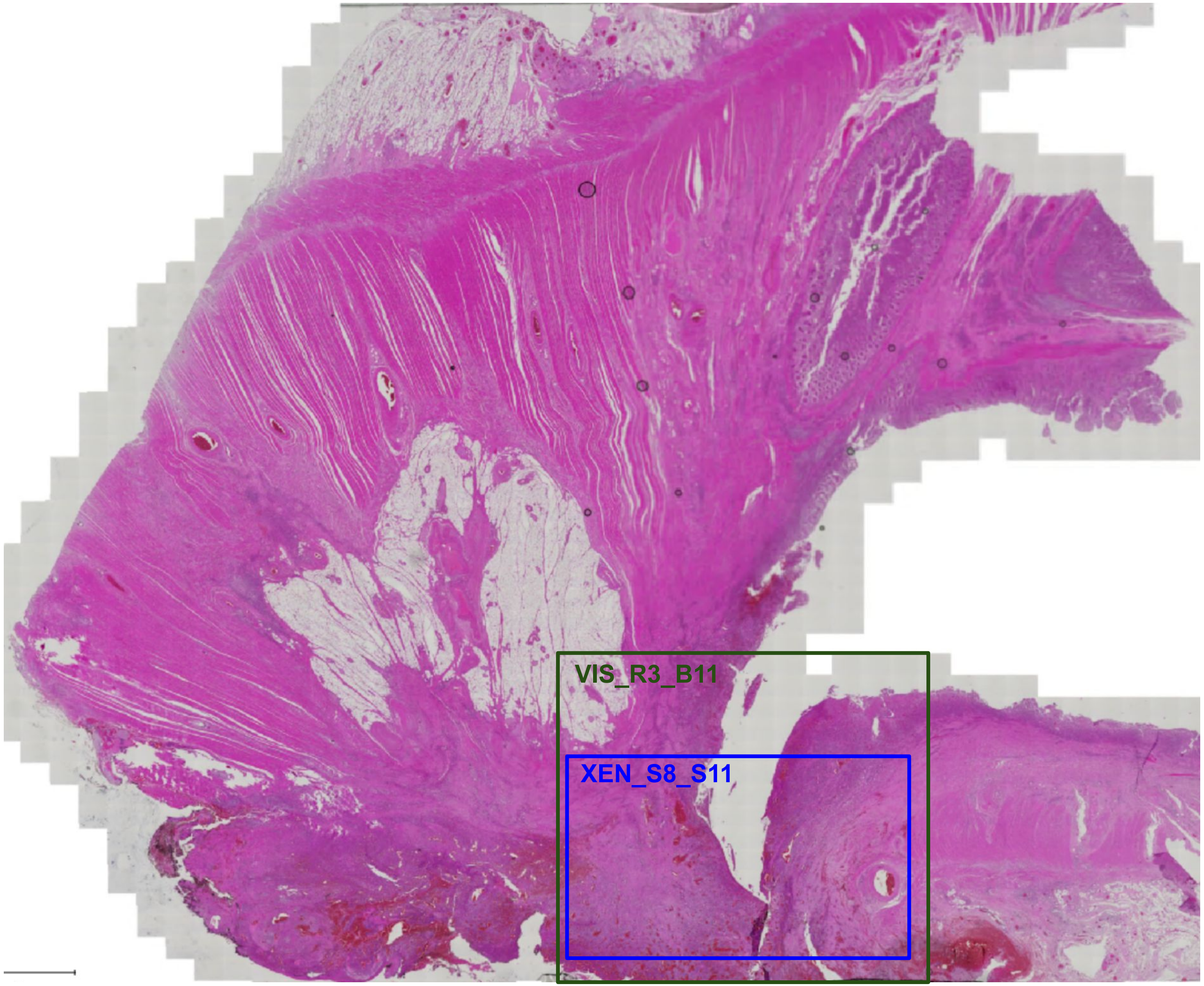

JR_31183_22

ILEAL CD FISTULA

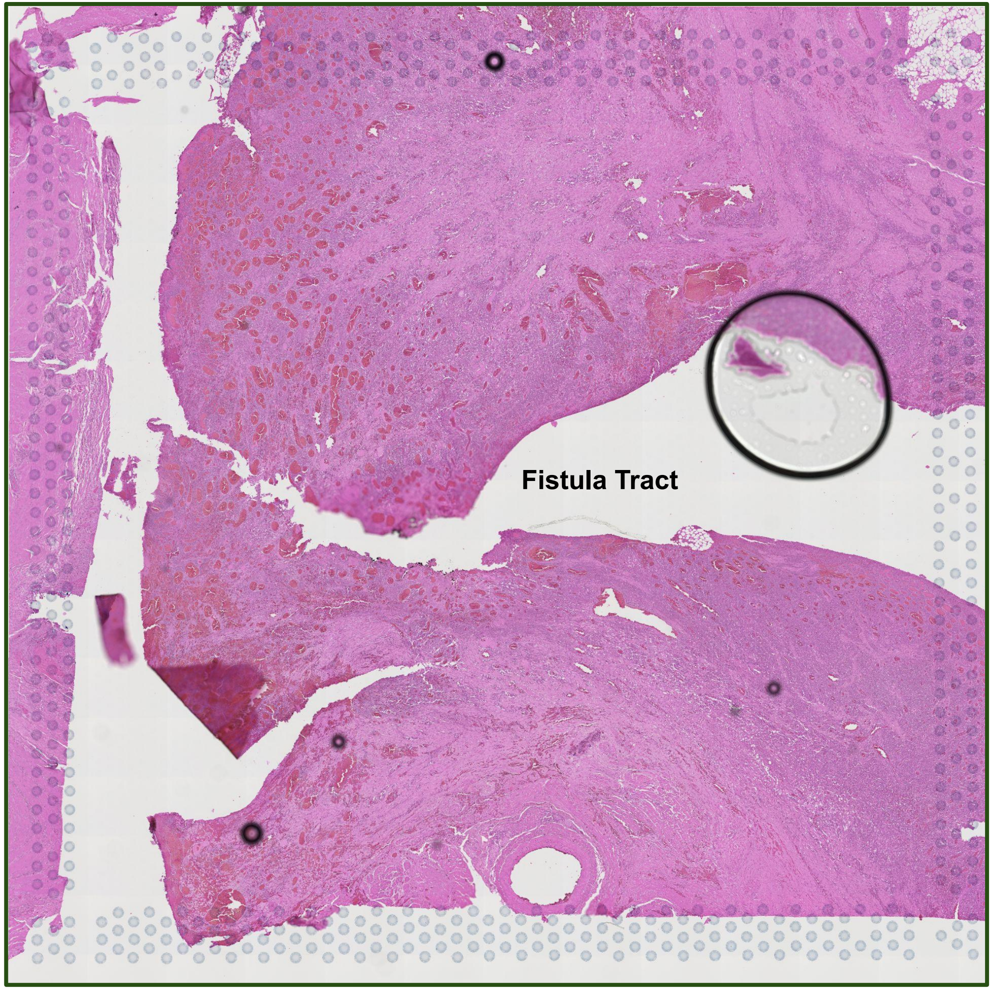

JR_35259_13

ILEOSIGMOID CD FISTULA

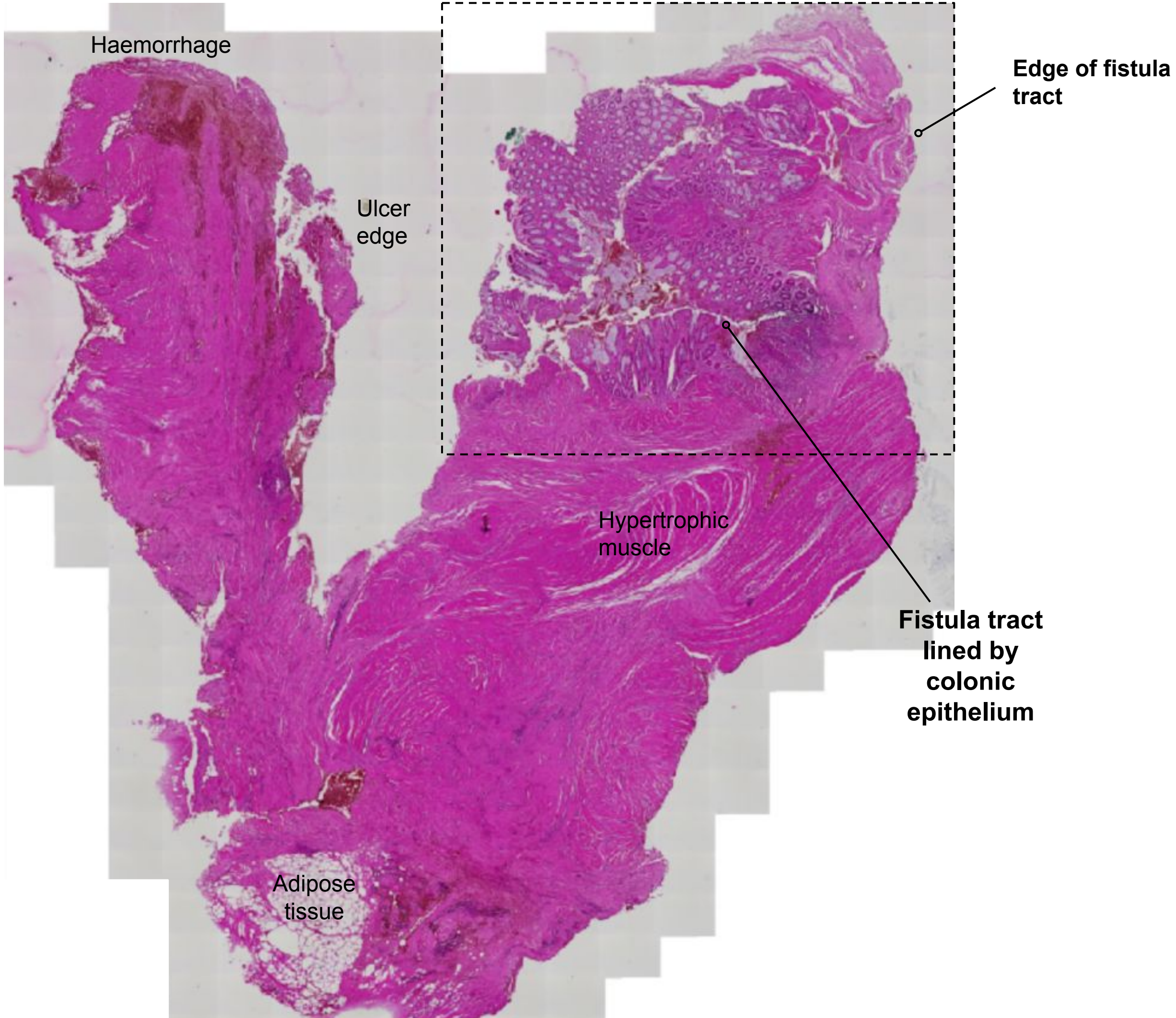

JR_35259_13

ILEOSIGMOID CD
FISTULA

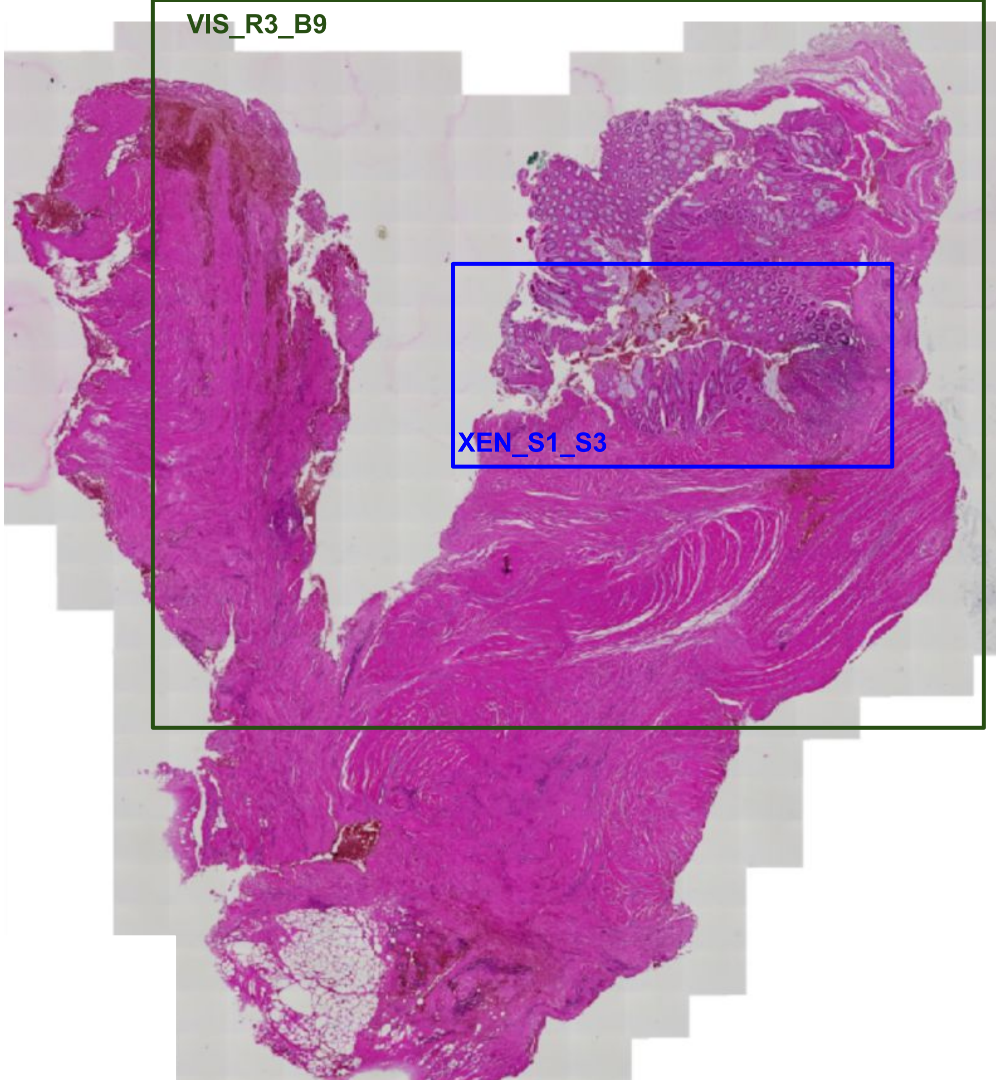

JR_35259_13

ILEOSIGMOID CD
FISTULA

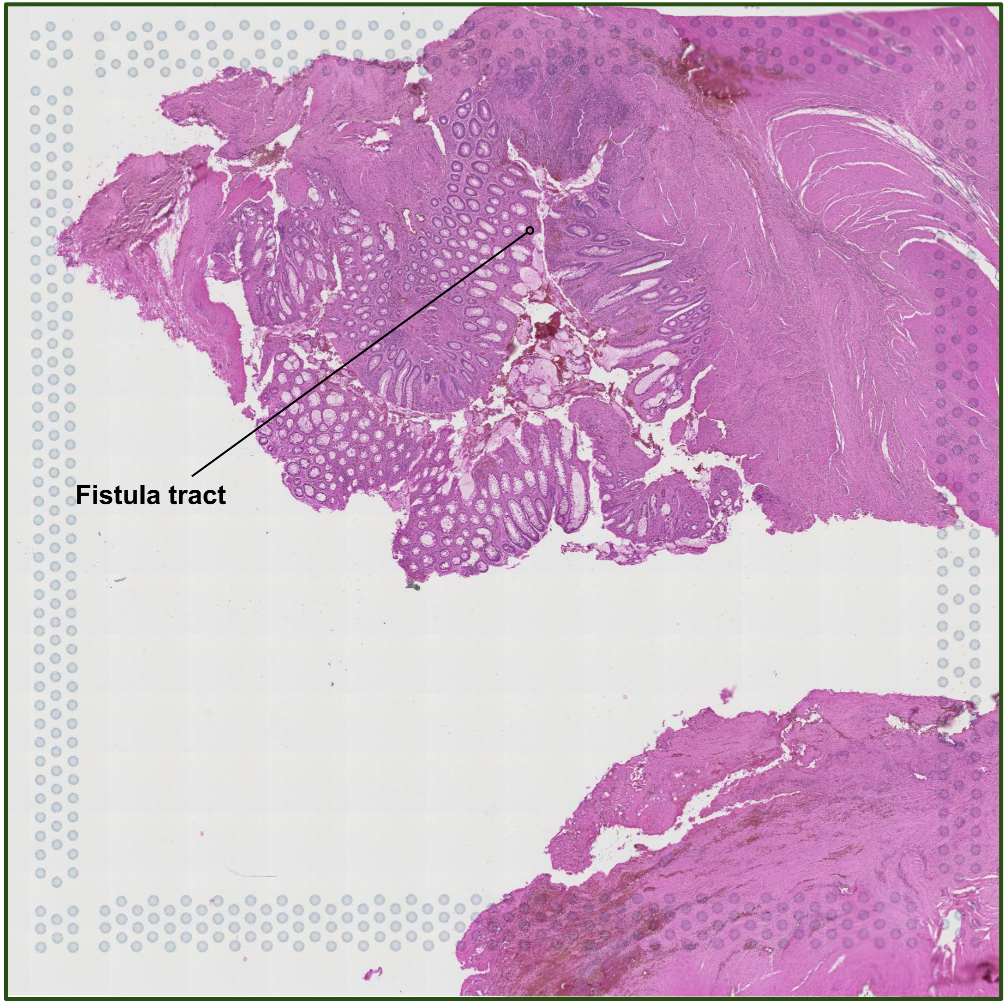

JR_37317_21

ILEAL CD FISTULA

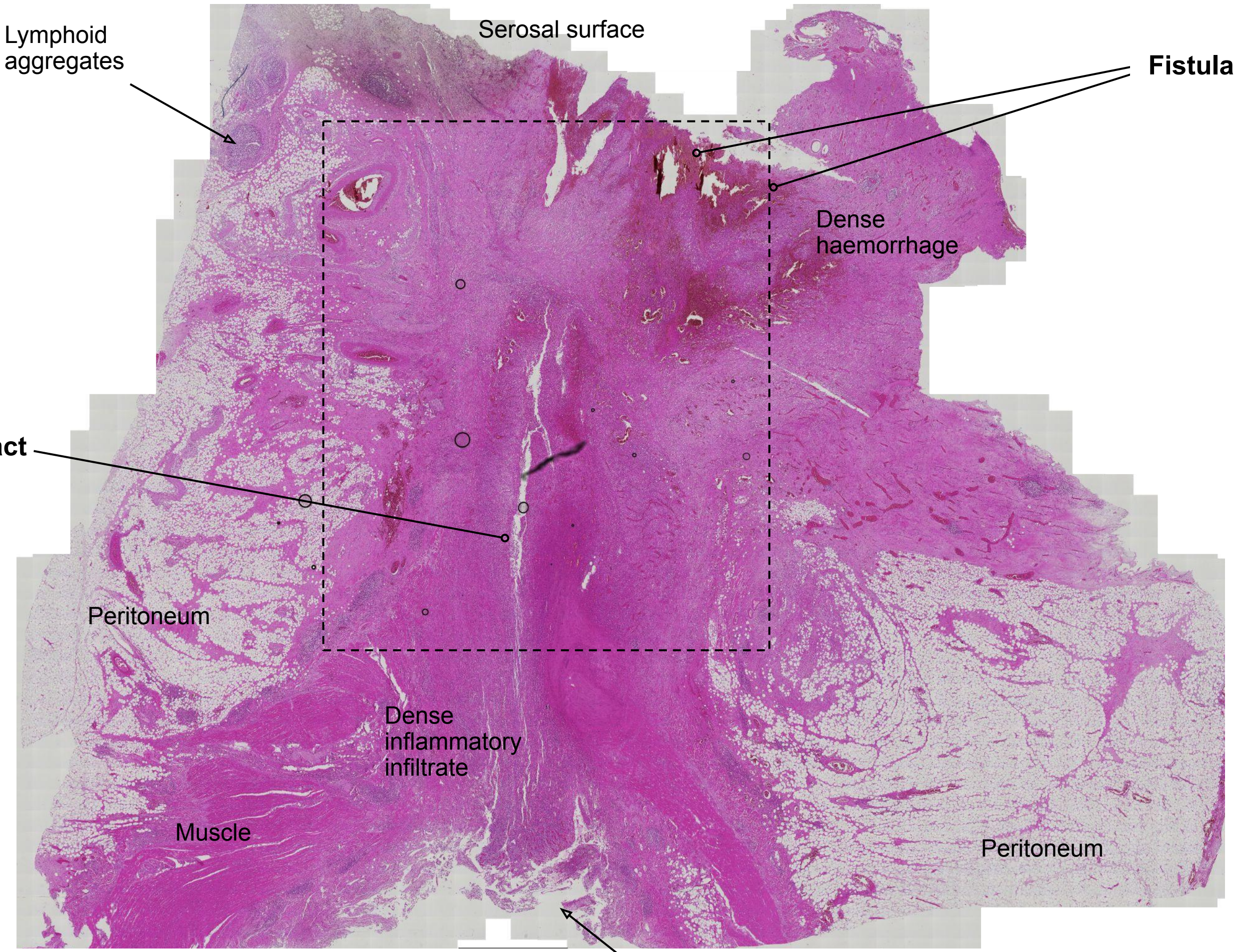

JR_37317_21

ILEAL CD FISTULA

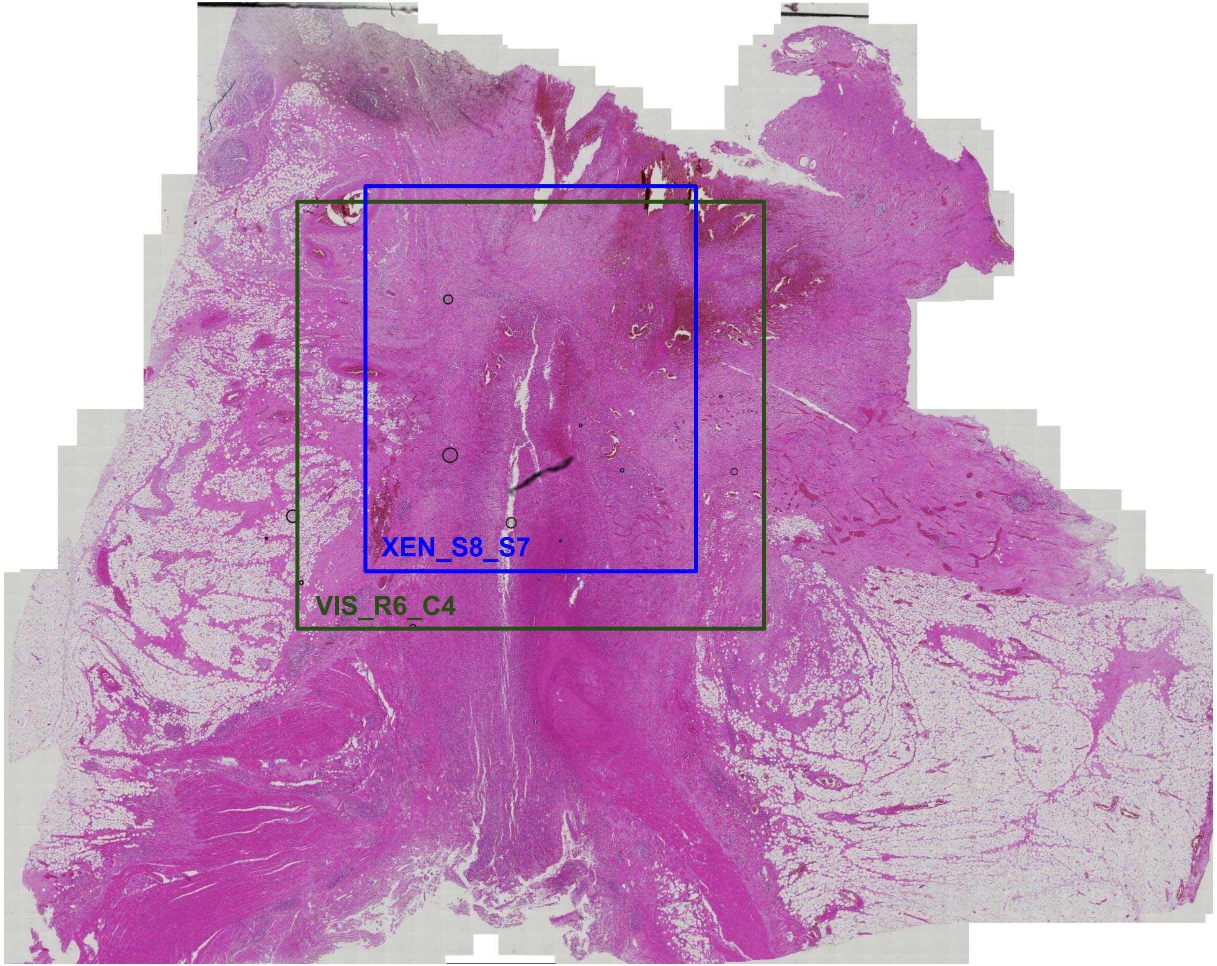

JR_37317_21

ILEAL CD FISTULA

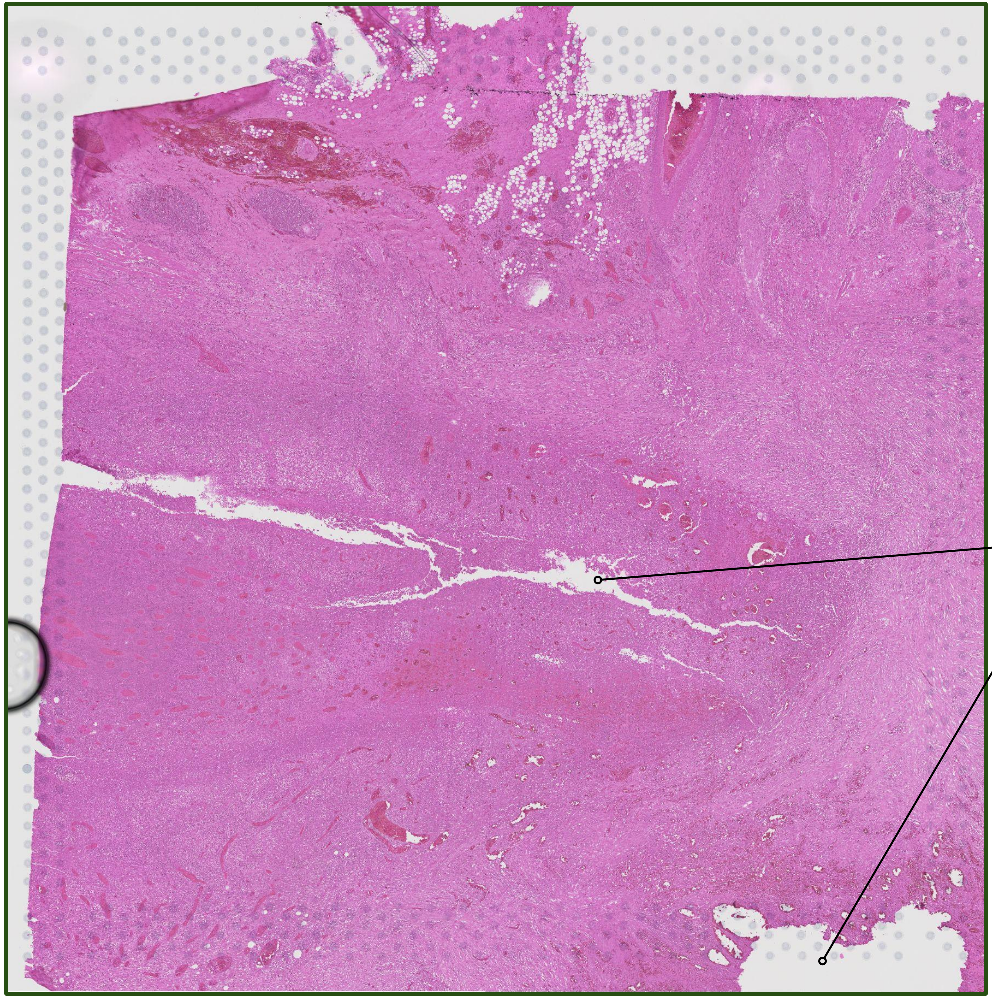

JR_47926_22

COLONIC CD FISTULA

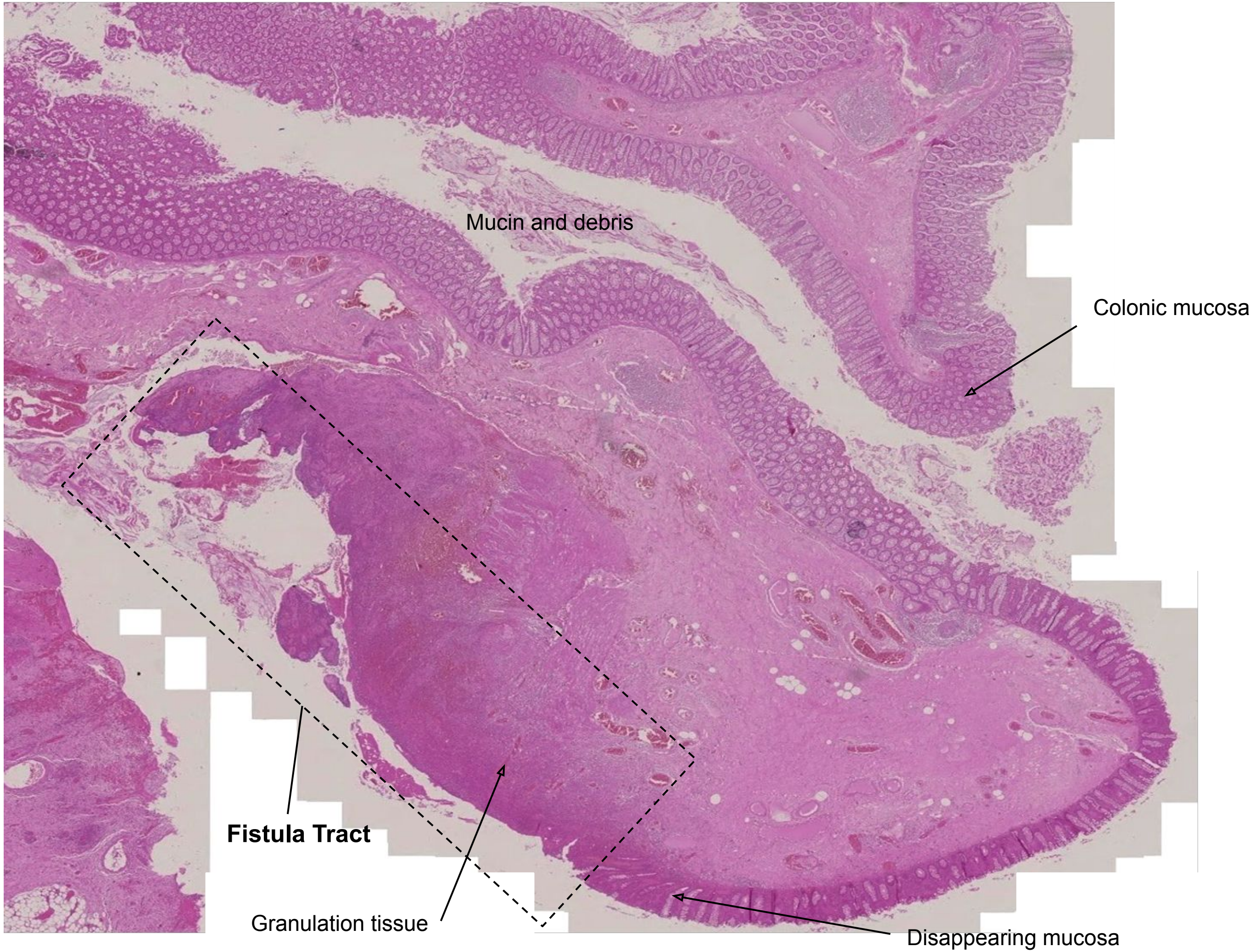

JR_47926_22

COLONIC CD FISTULA

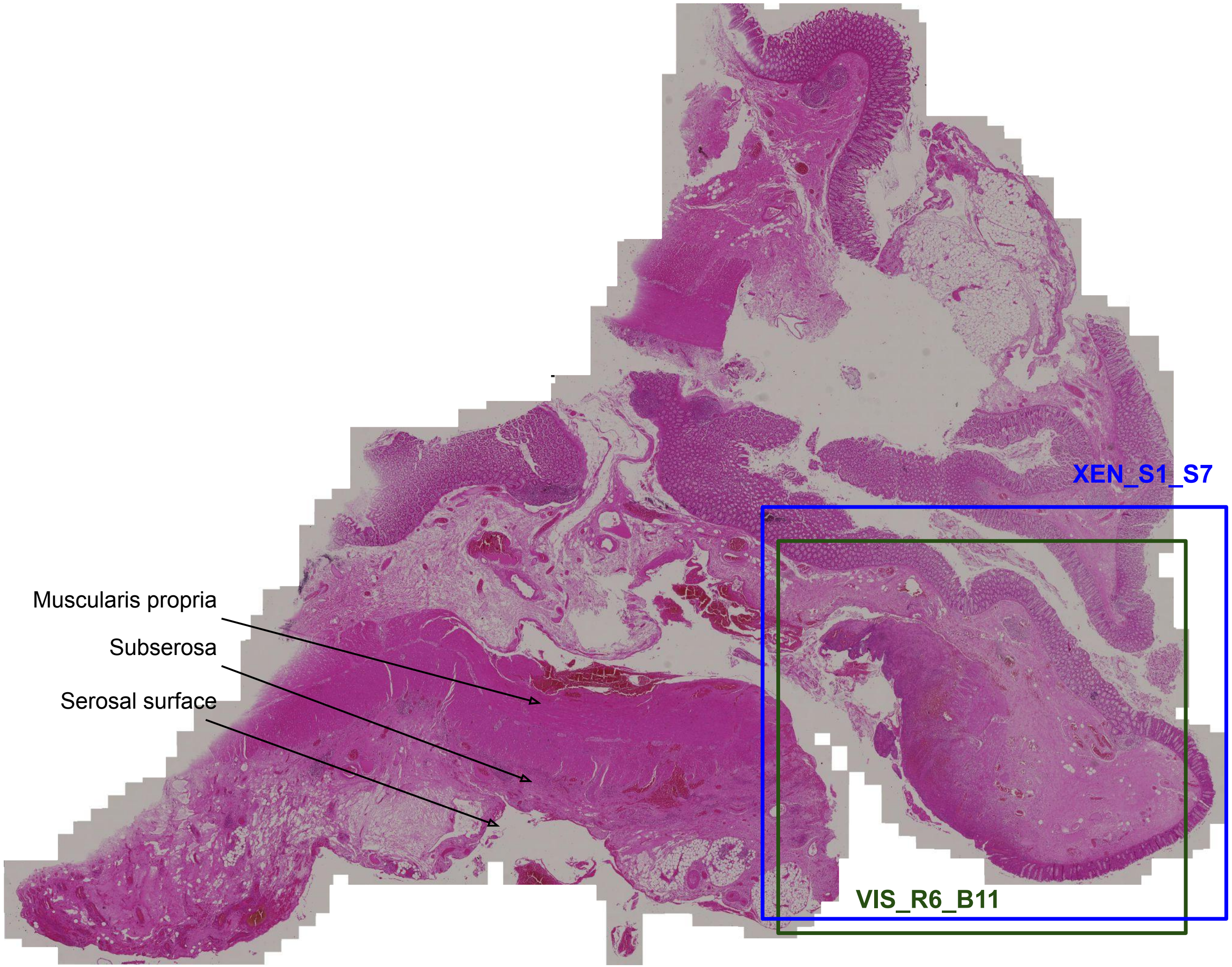

JR_47926_22

COLONIC CD FISTULA

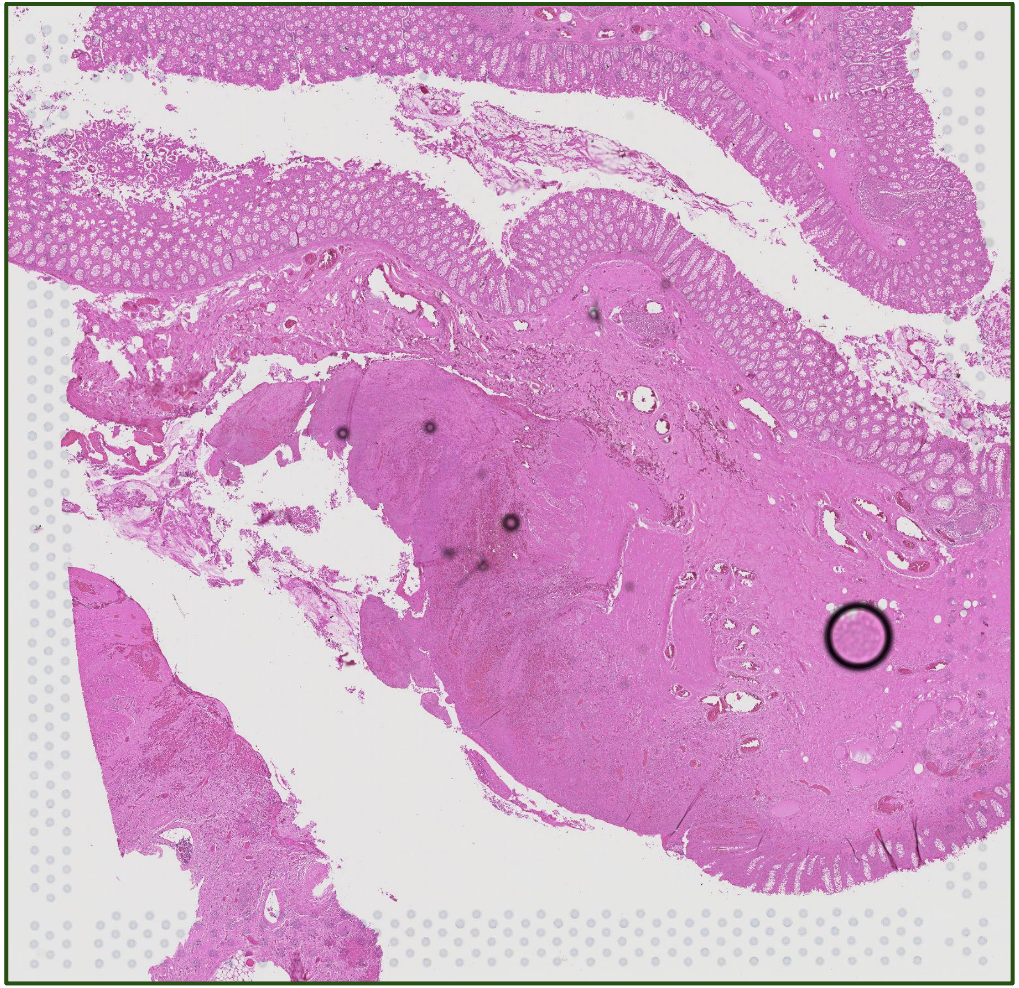

JR_49083_22

COLONIC CD FISTULA

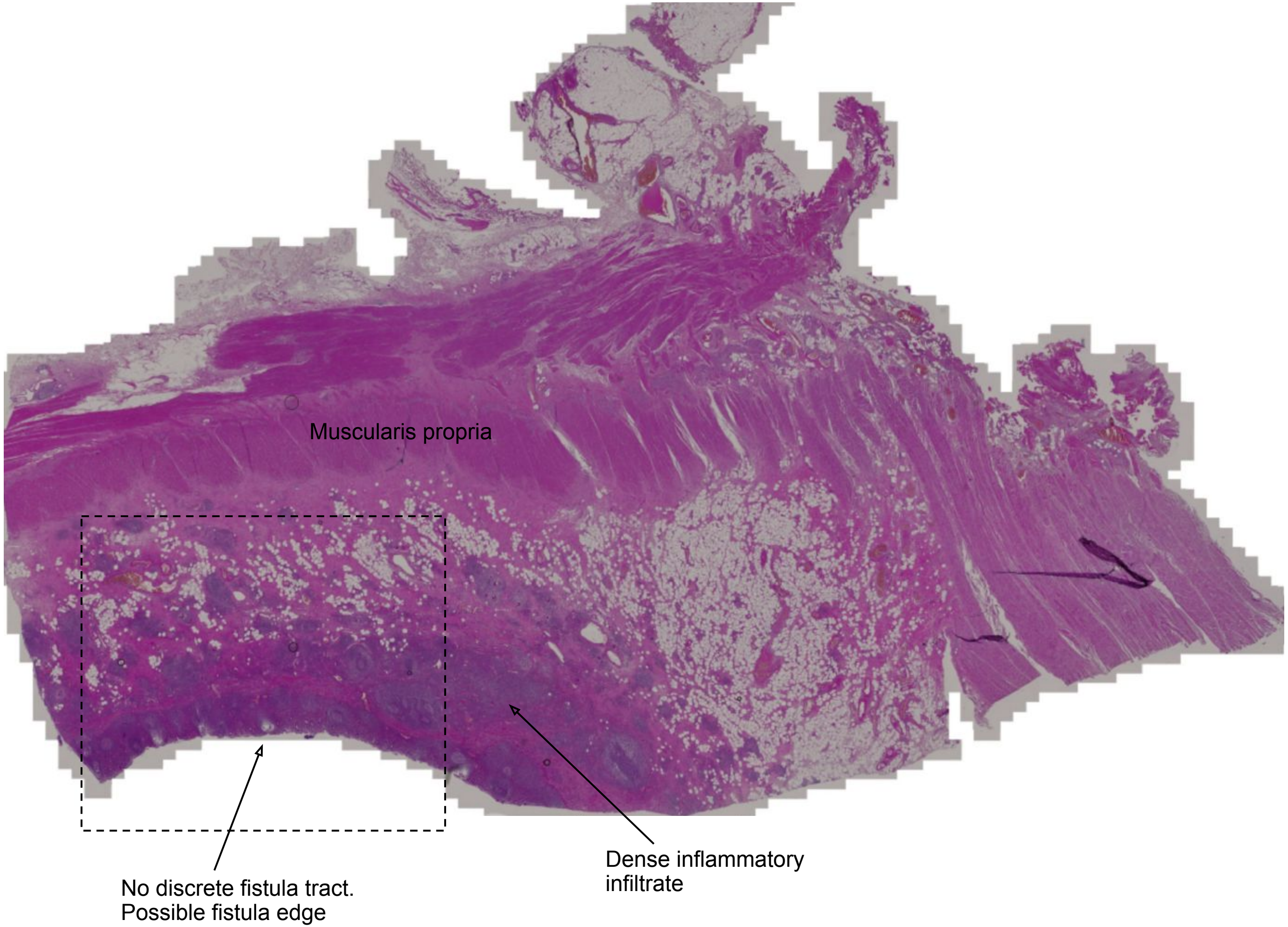

JR_49083_22

COLONIC CD FISTULA

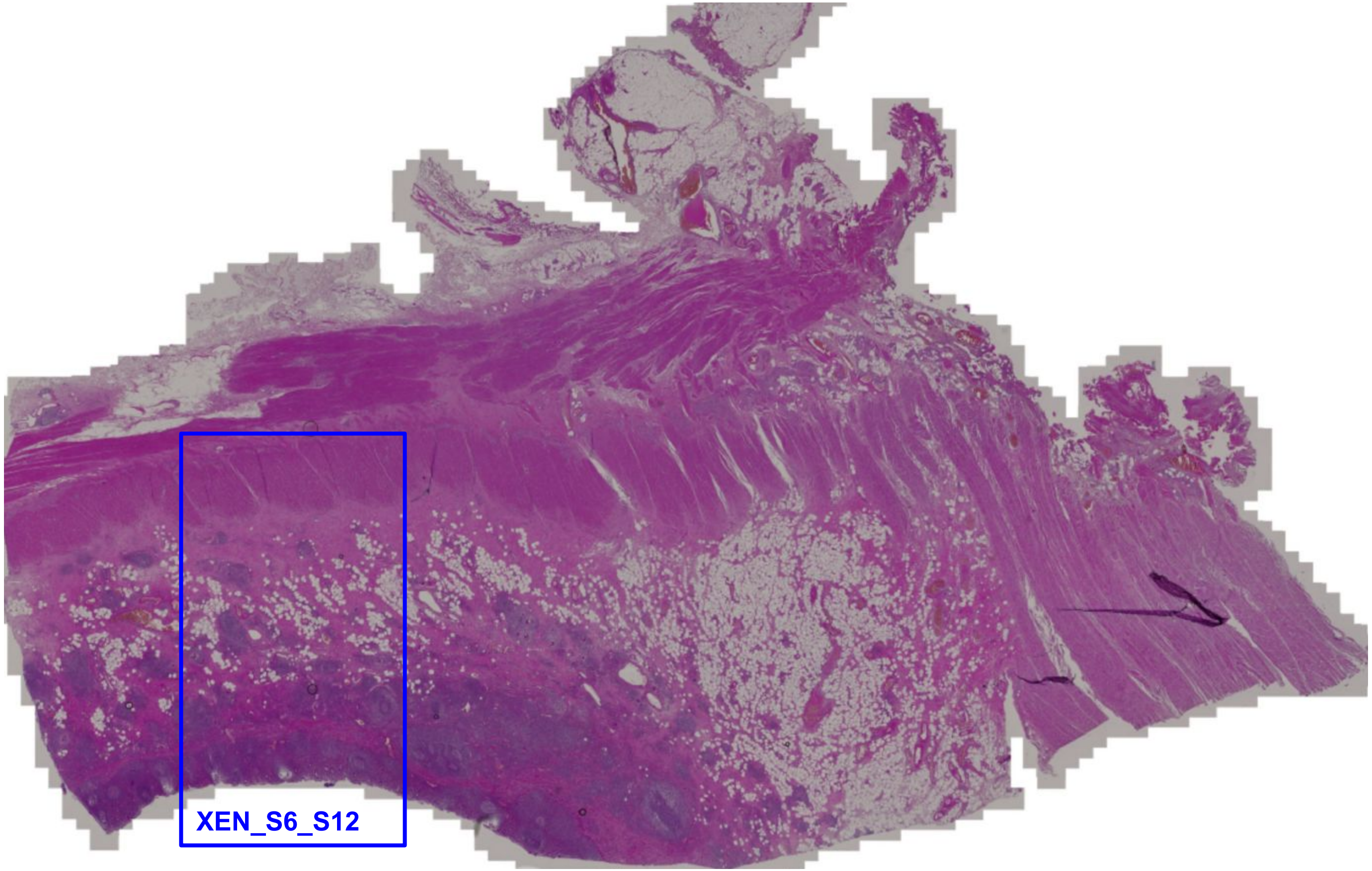

JR_50919_22

PERIANAL CD
FISTULA

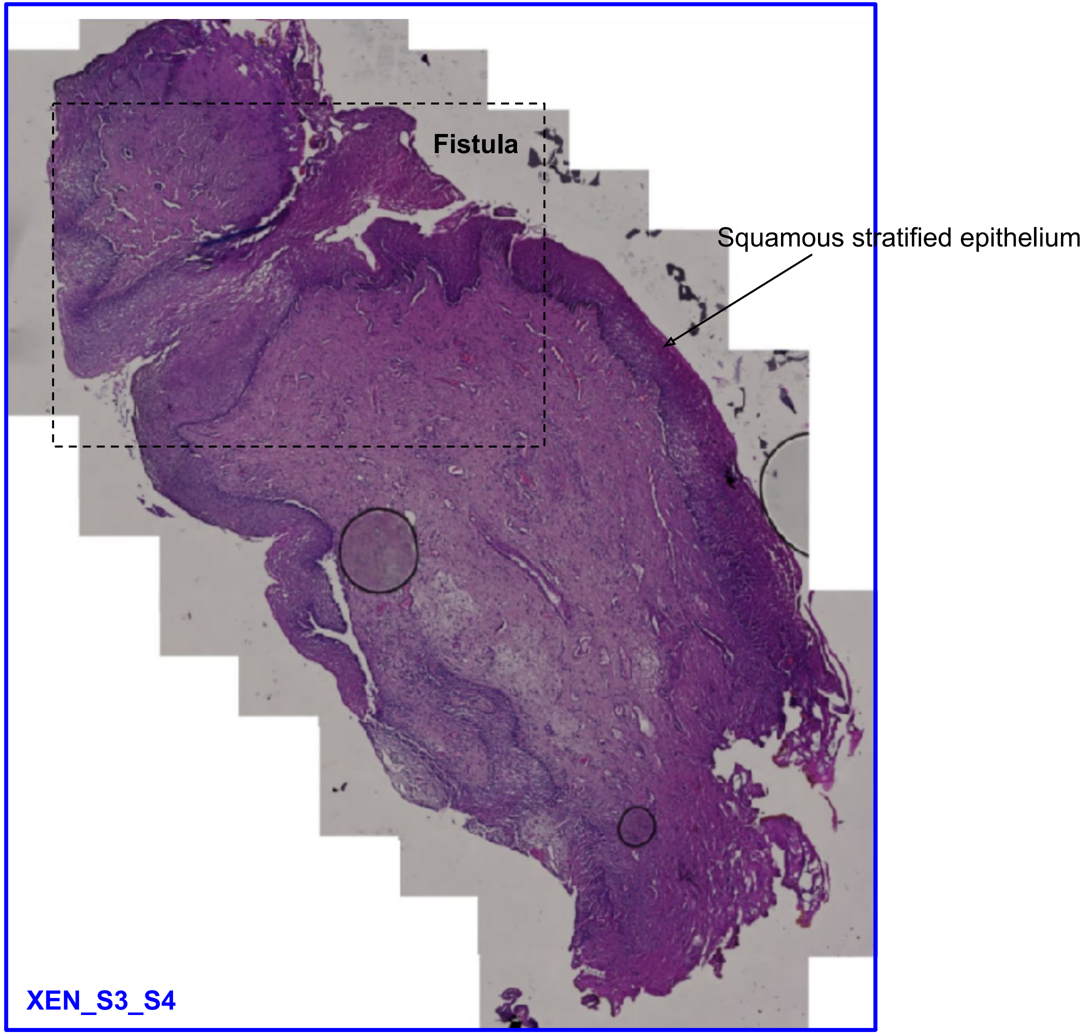

JR_51343_21

ILEOSIGMOID CD FISTULA

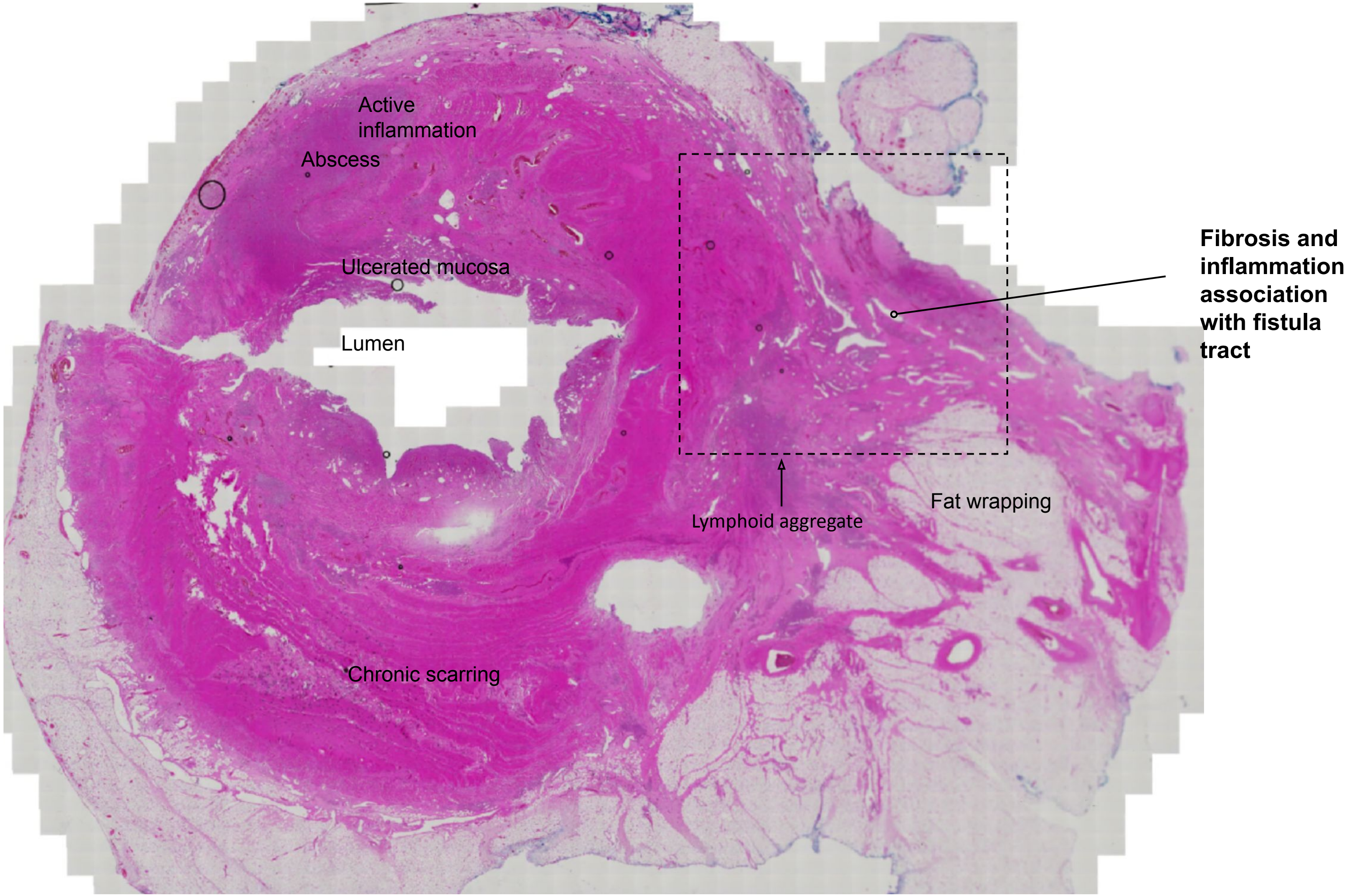

JR_51343_21

ILEOSIGMOID CD
FISTULA

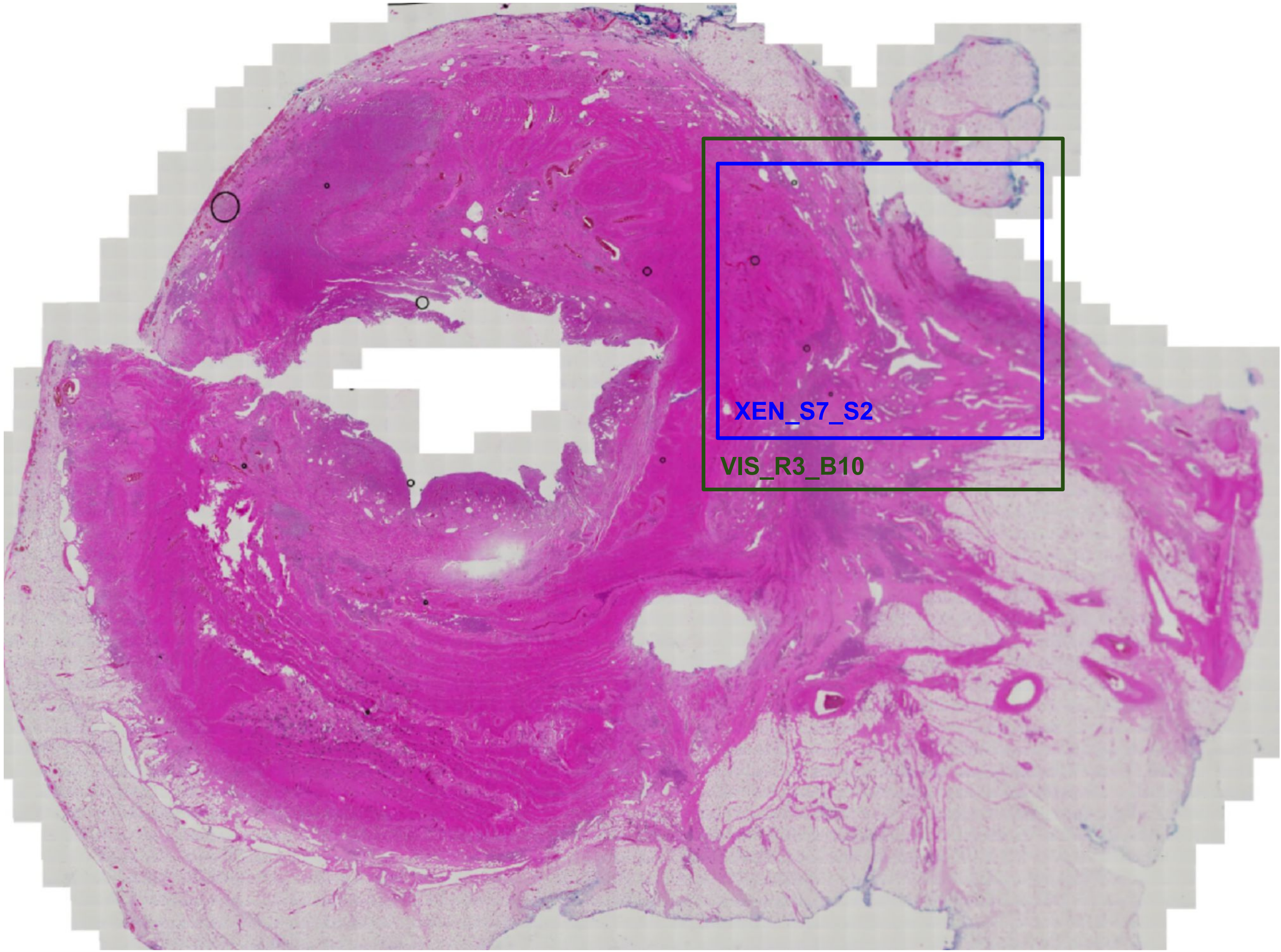

JR_51343_21

ILEOSIGMOID CD FISTULA

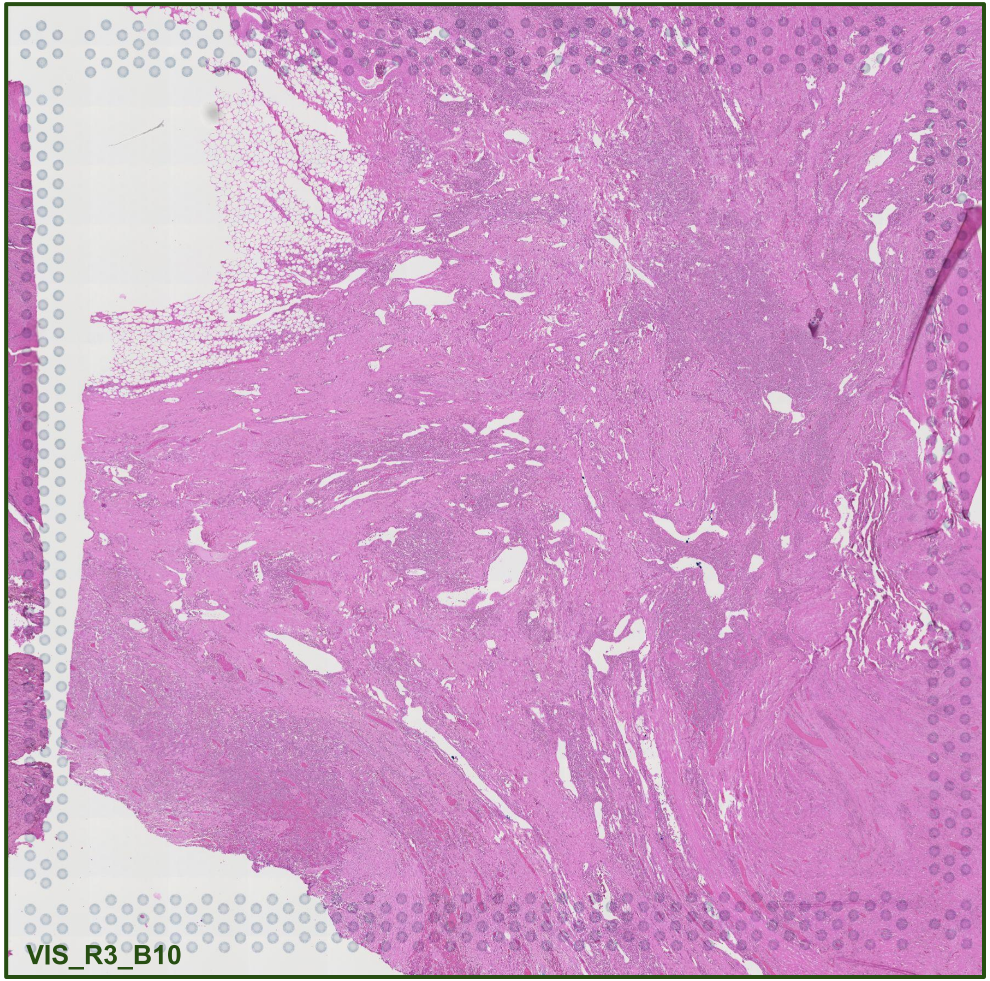

JR_7909_23

PERIANAL CD
FISTULA

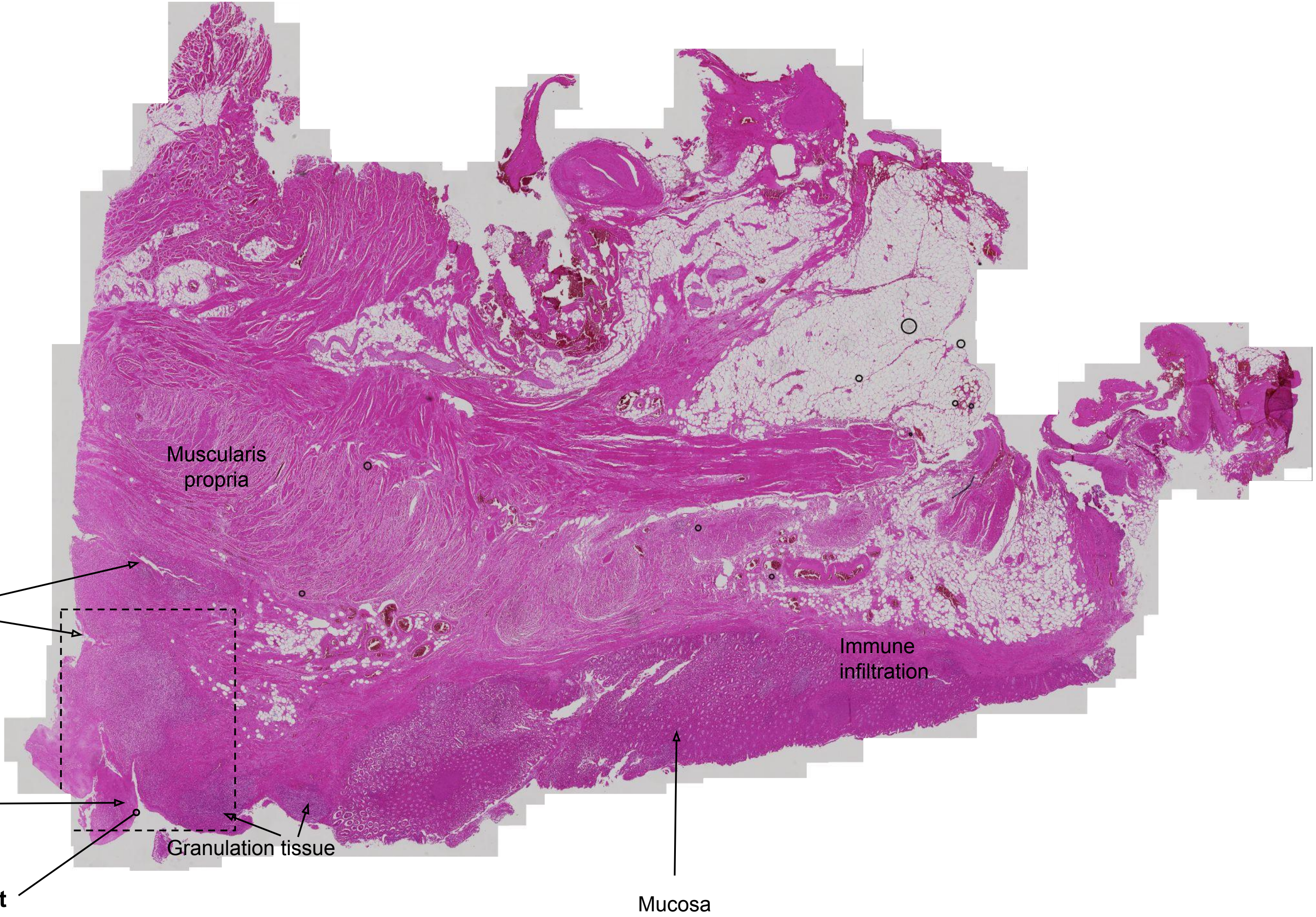

JR_7909_23

PERIANAL CD
FISTULA

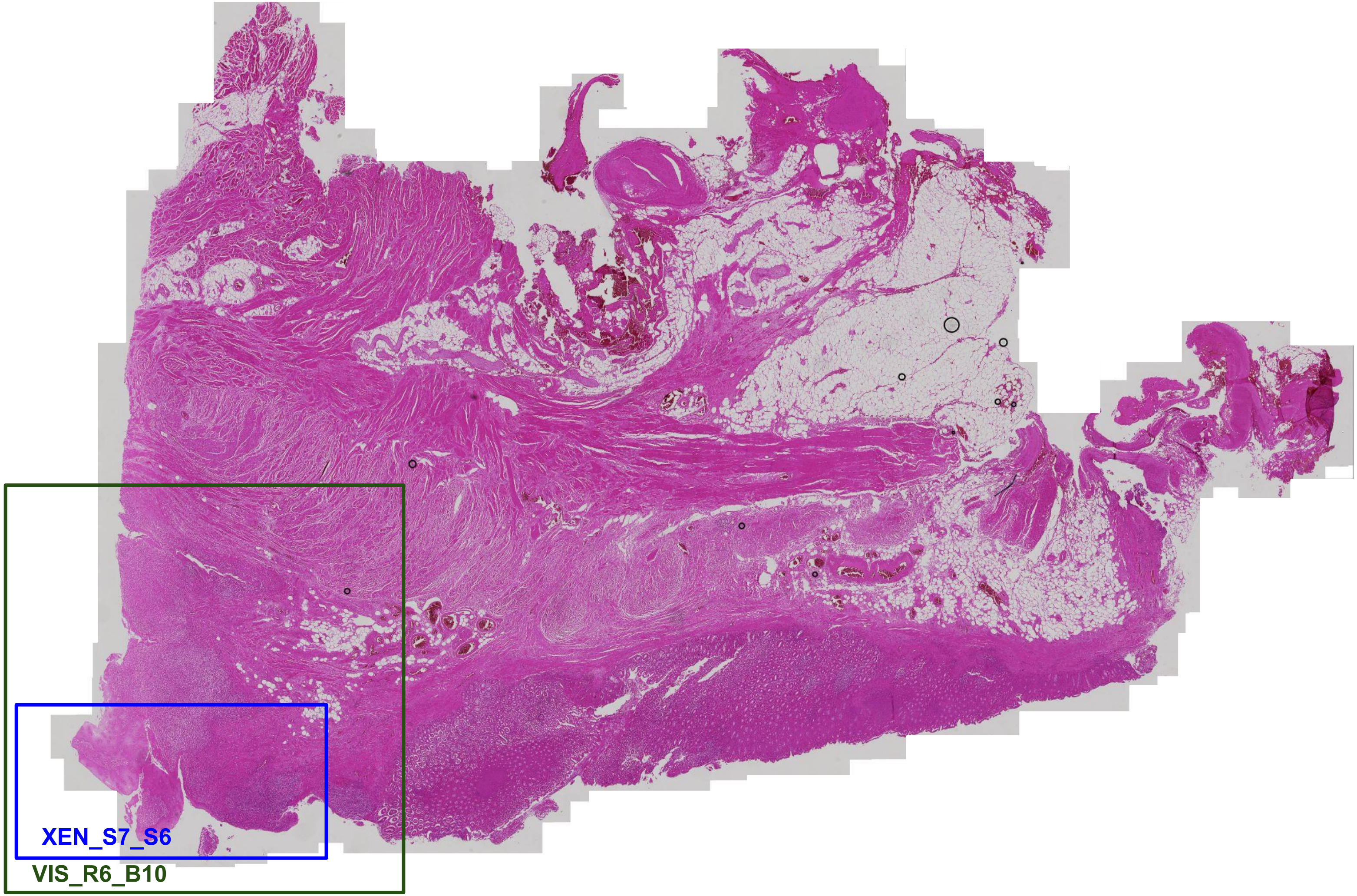

JR_7909_23

PERIANAL CD FISTULA

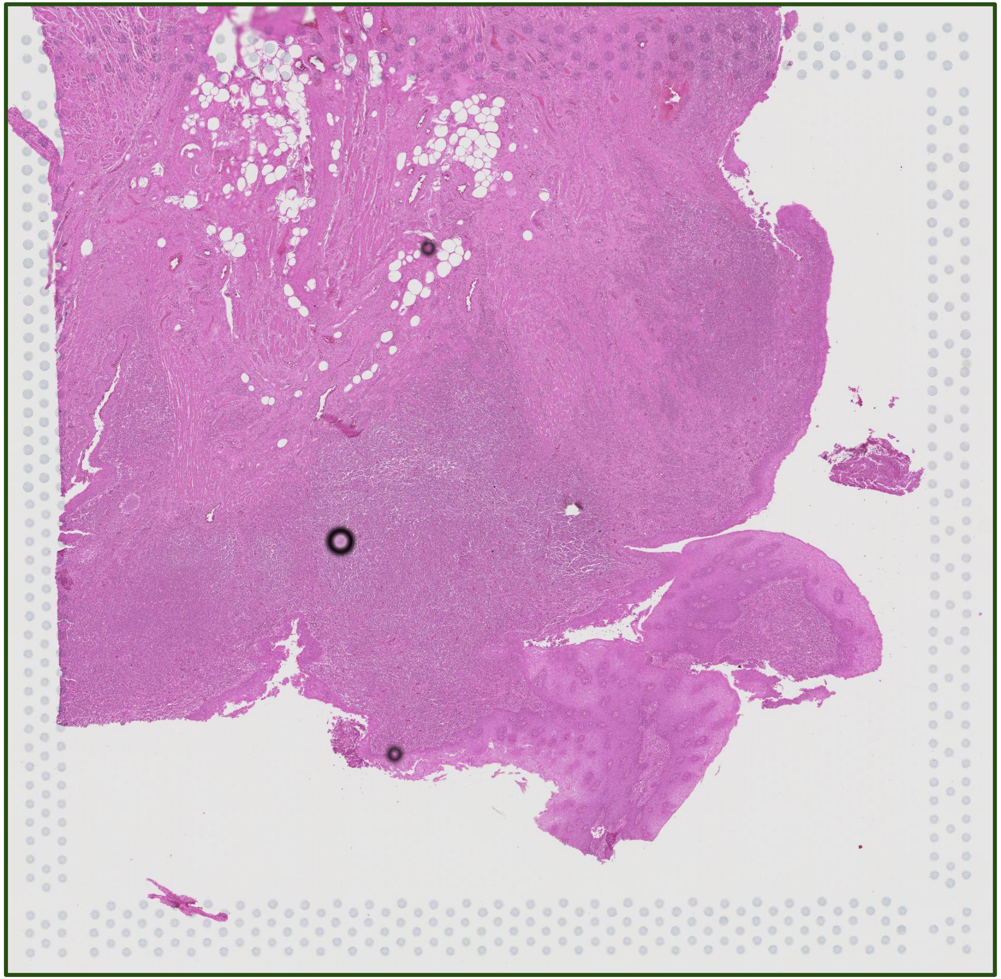

JR_8610_23

PERIANAL CD FISTULA

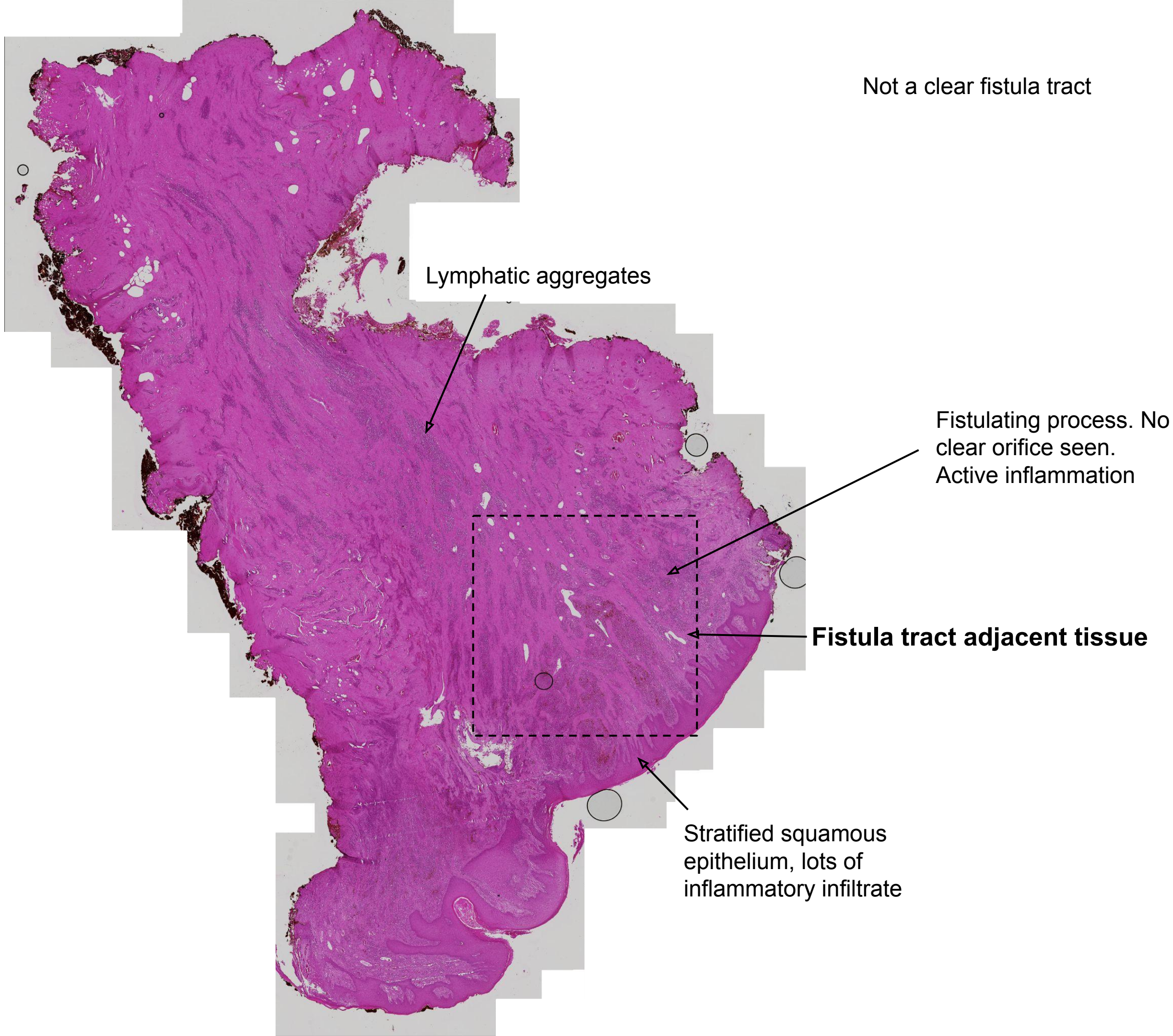

JR_8610_23

PERIANAL CD
FISTULA

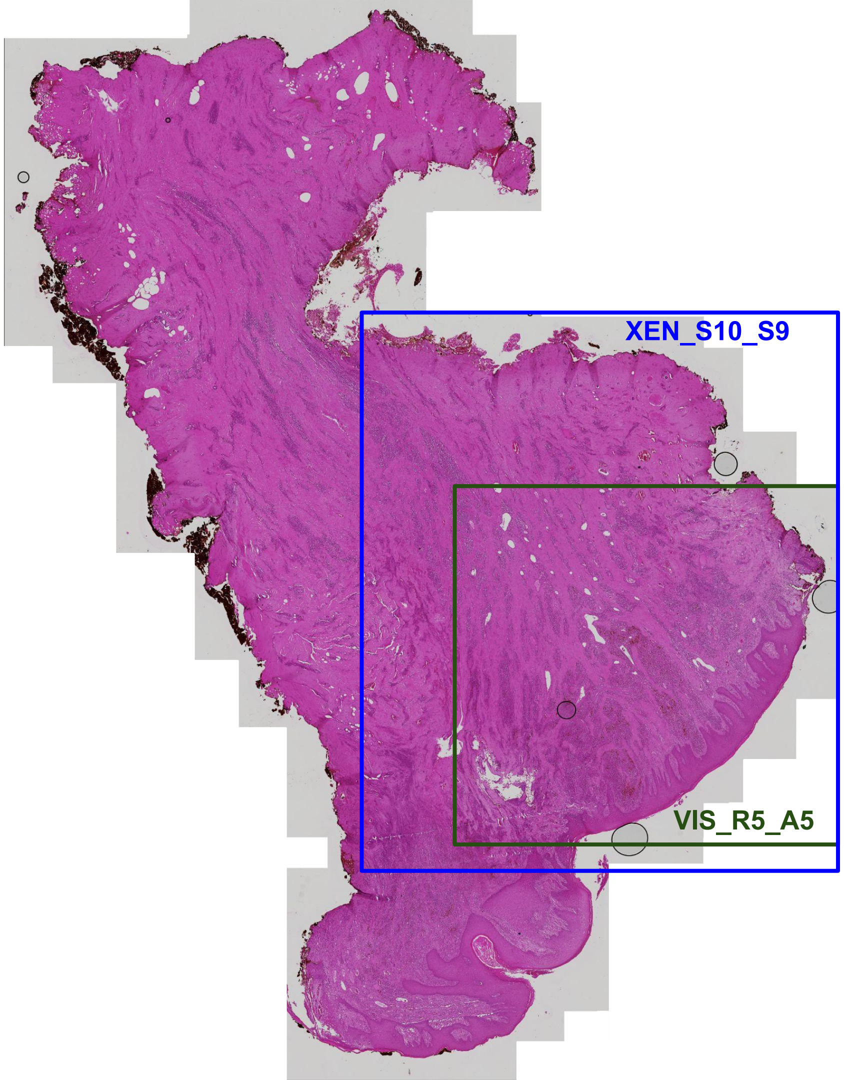

JR_8610_23

PERIANAL CD
FISTULA

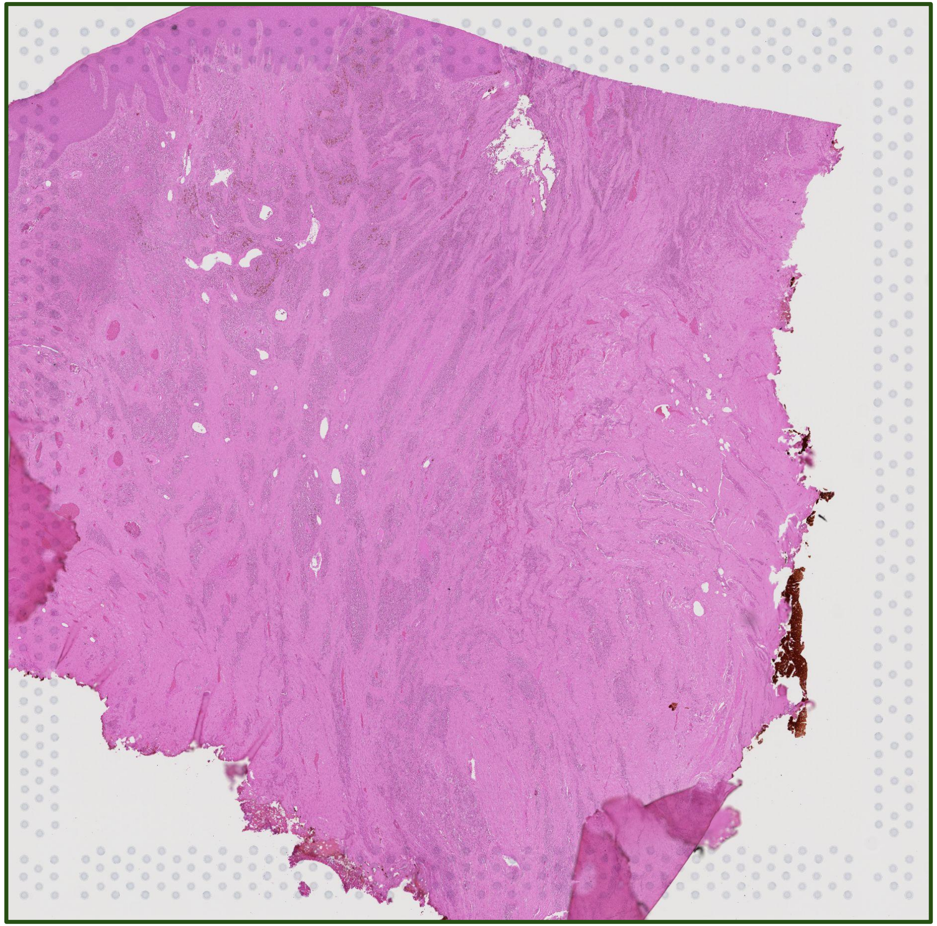

JR 28598_21 (TIP 535)

ENTEROCUTANEOUS CD FISTULA TRACT

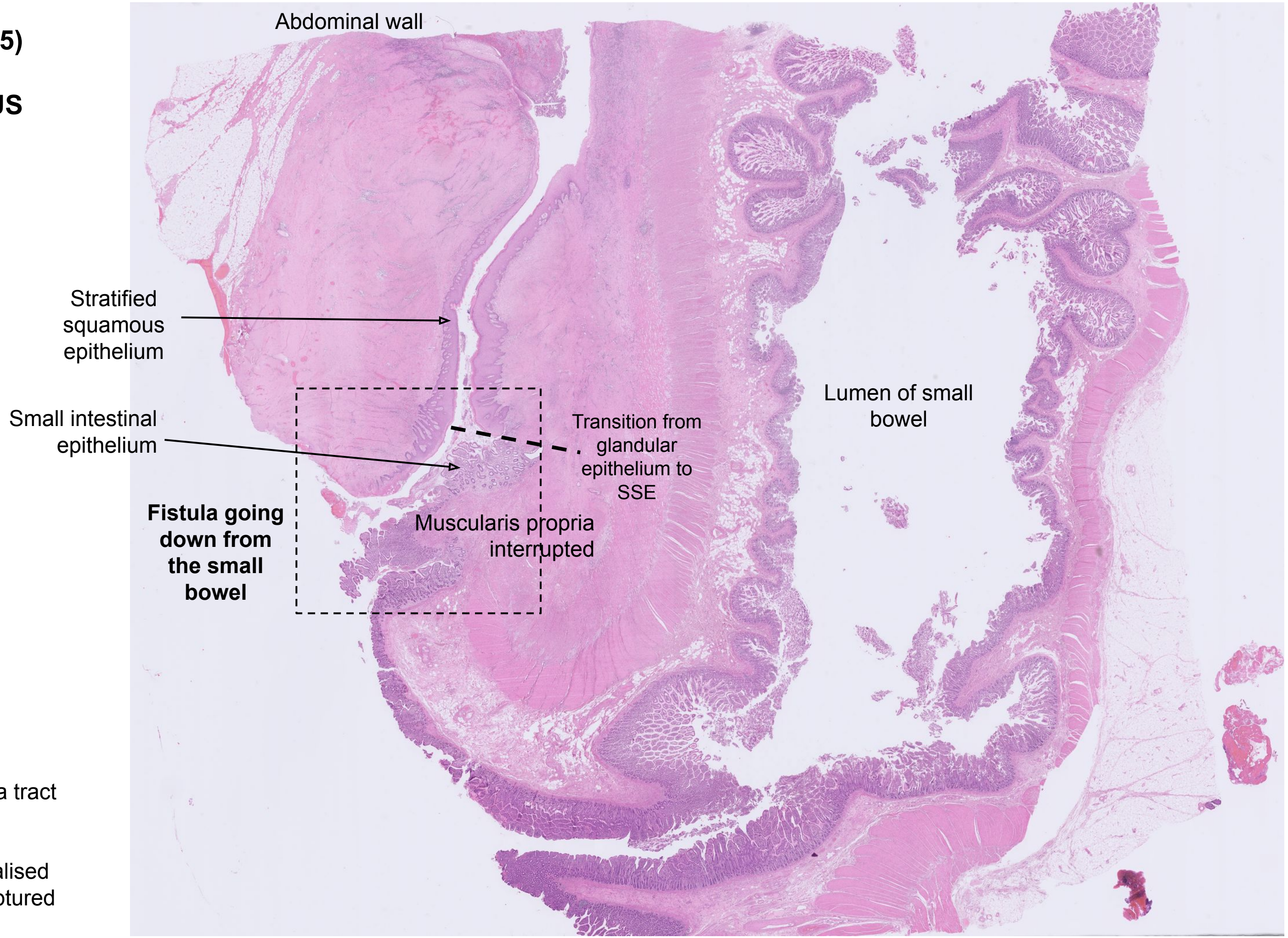

Entire fistula tract captured

Re-epithelialised process captured

JR 28598_21 (TIP 535)

ENTEROCUTANEOUS CD FISTULA TRACT

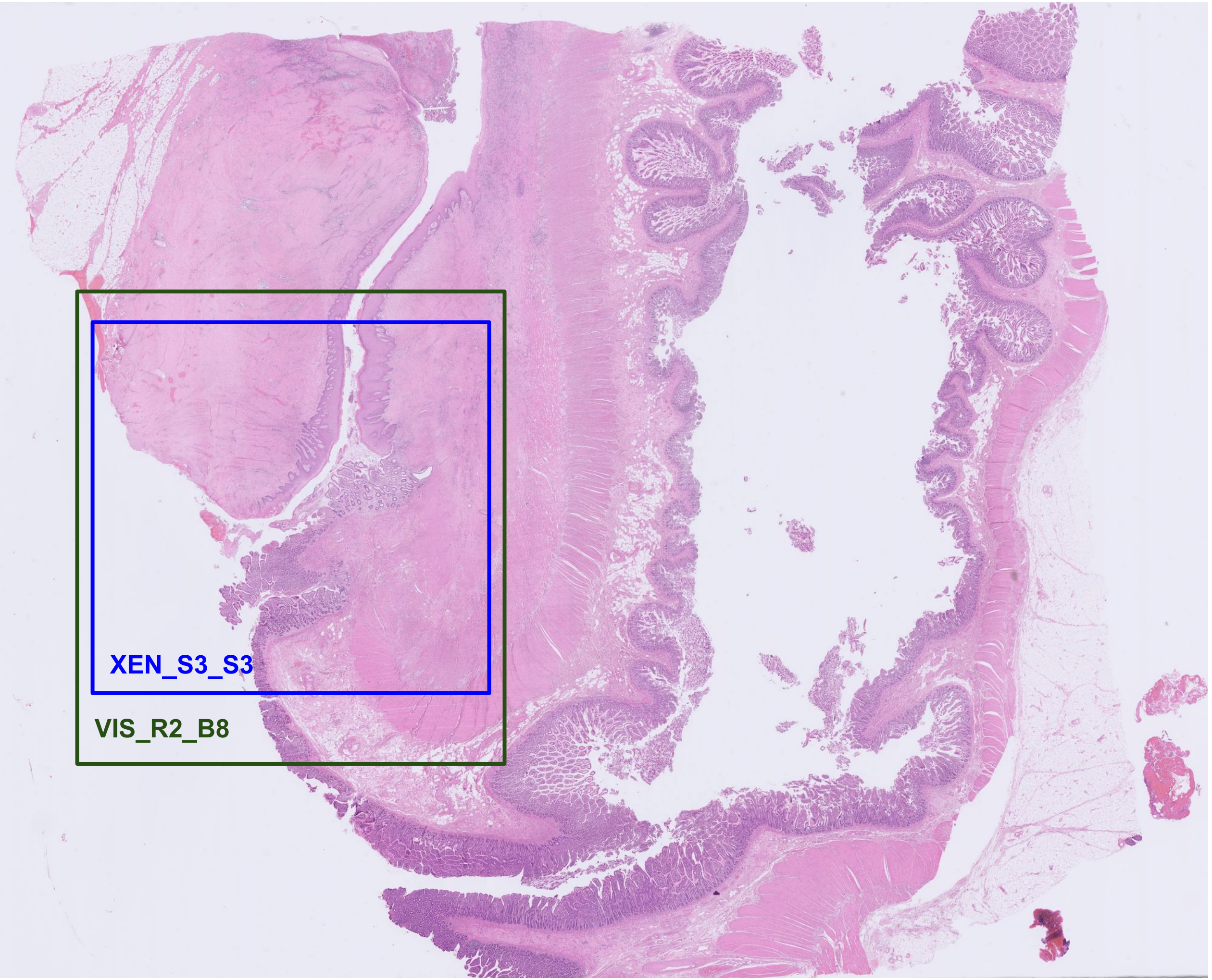

JR 28598_21 (TIP 535)

ENTEROCUTANEOUS CD FISTULA

Small bowel has an inflammatory reaction which perforates the skin

Abrupt transition point between columnar epithelium and SSE

Orifice of fistula lined by squamous epithelium

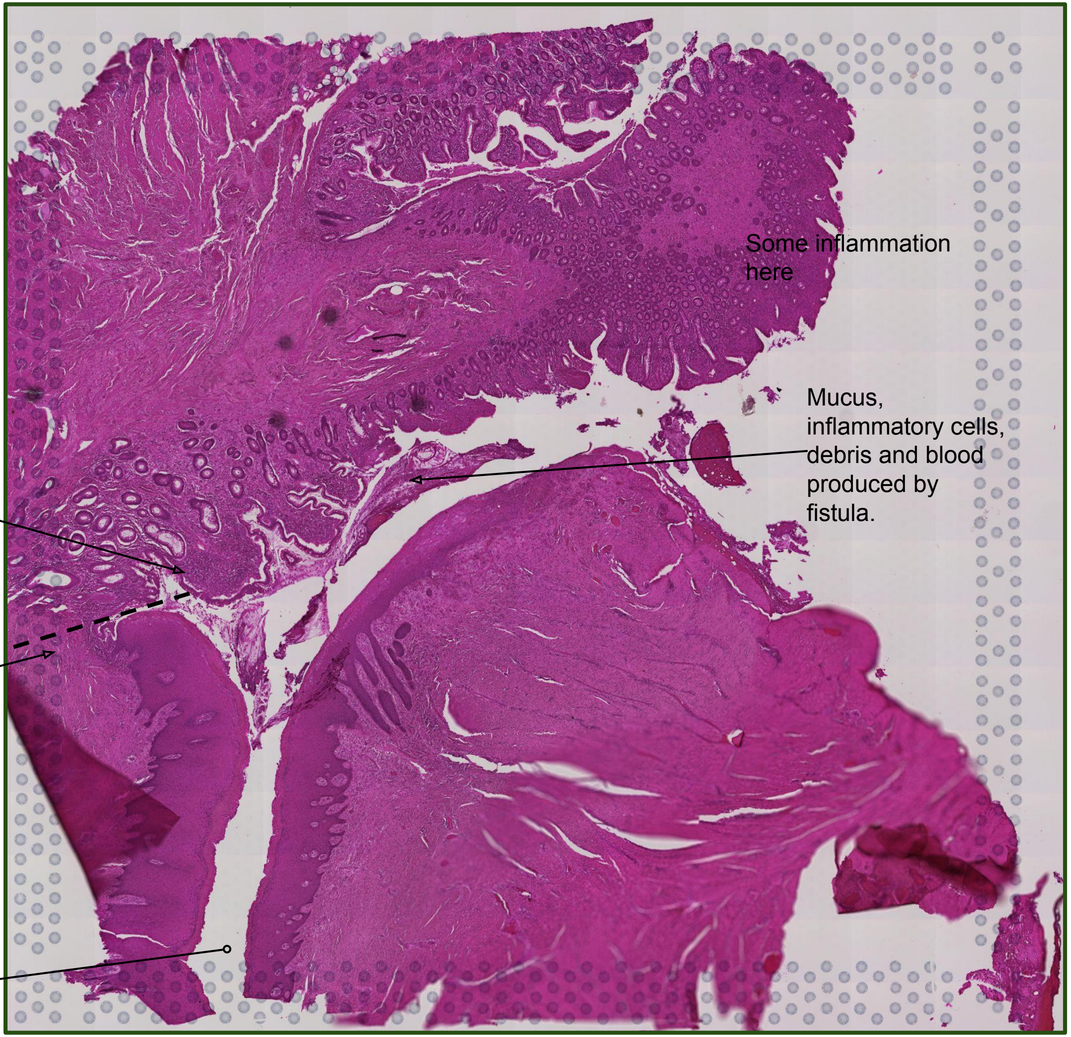

Fistula lined by intestinal epithelium

TIP 535

ENTEROCUTANEOUS CD FISTULA TRACT

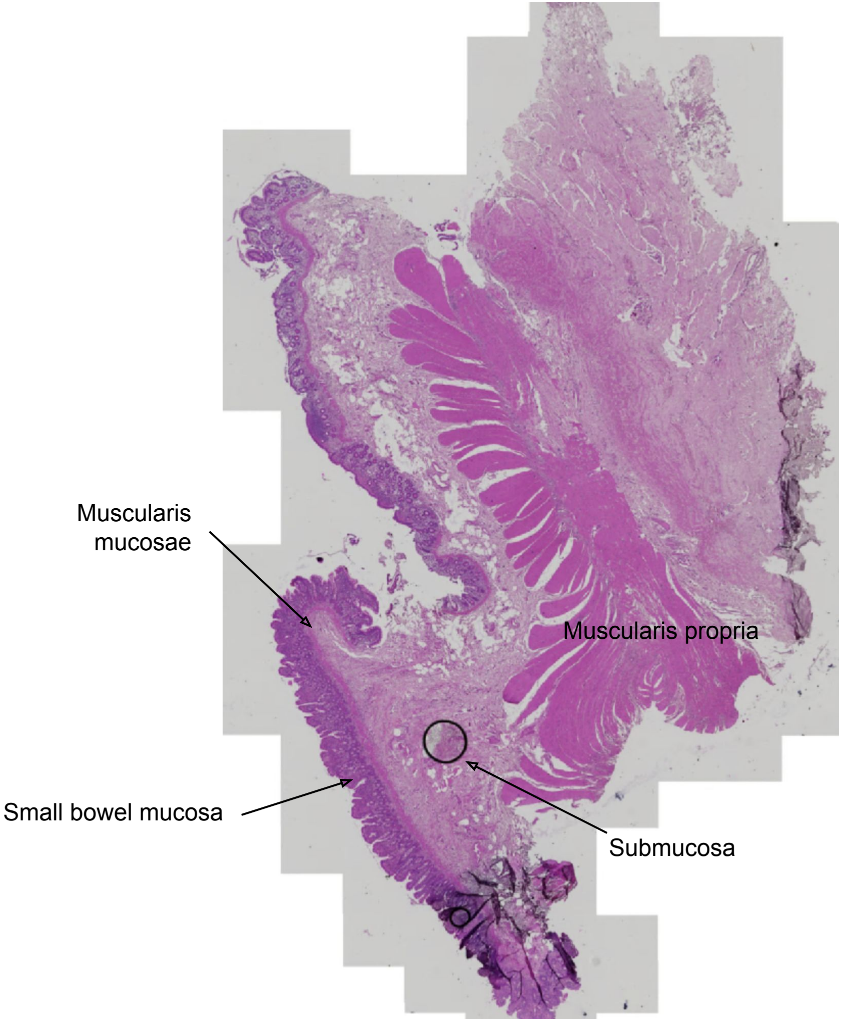

TIP 535

ENTEROCUTANEOUS
CD FISTULA TRACT

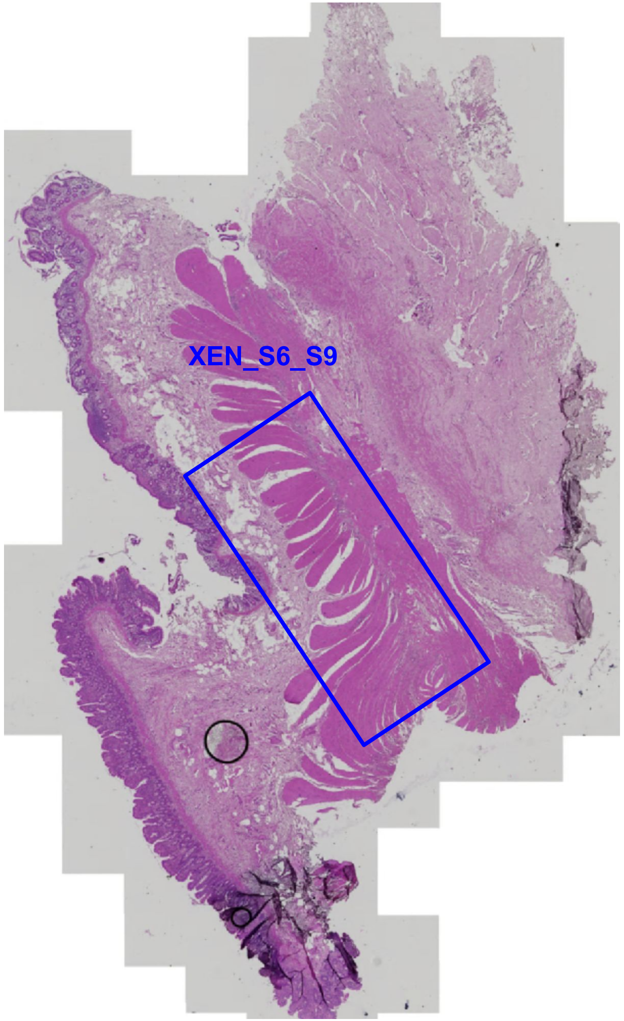

TIP 559

ILEOCOLIC CD FISTULA

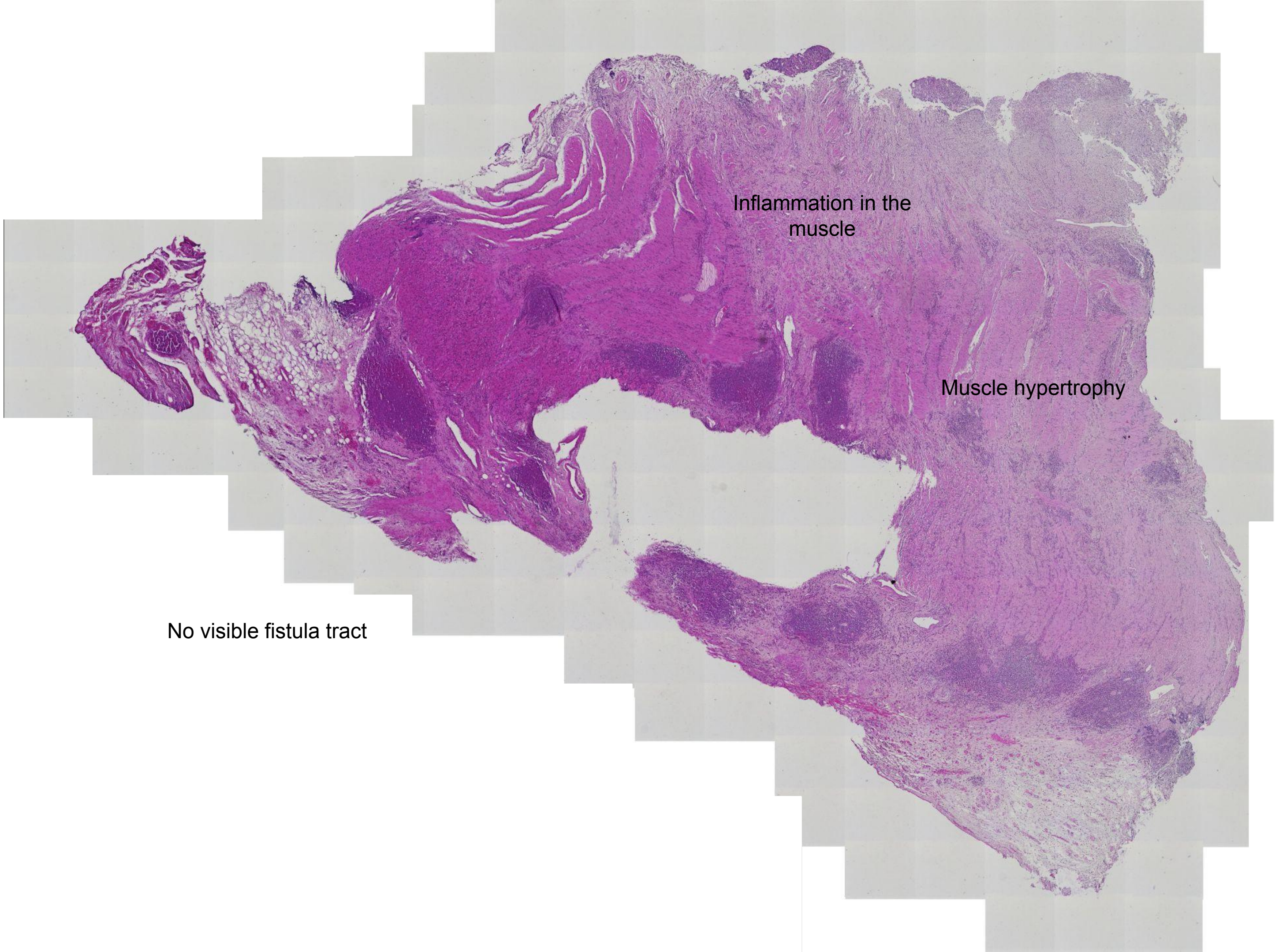

TIP 559

ILEOCOLIC CD
FISTULA

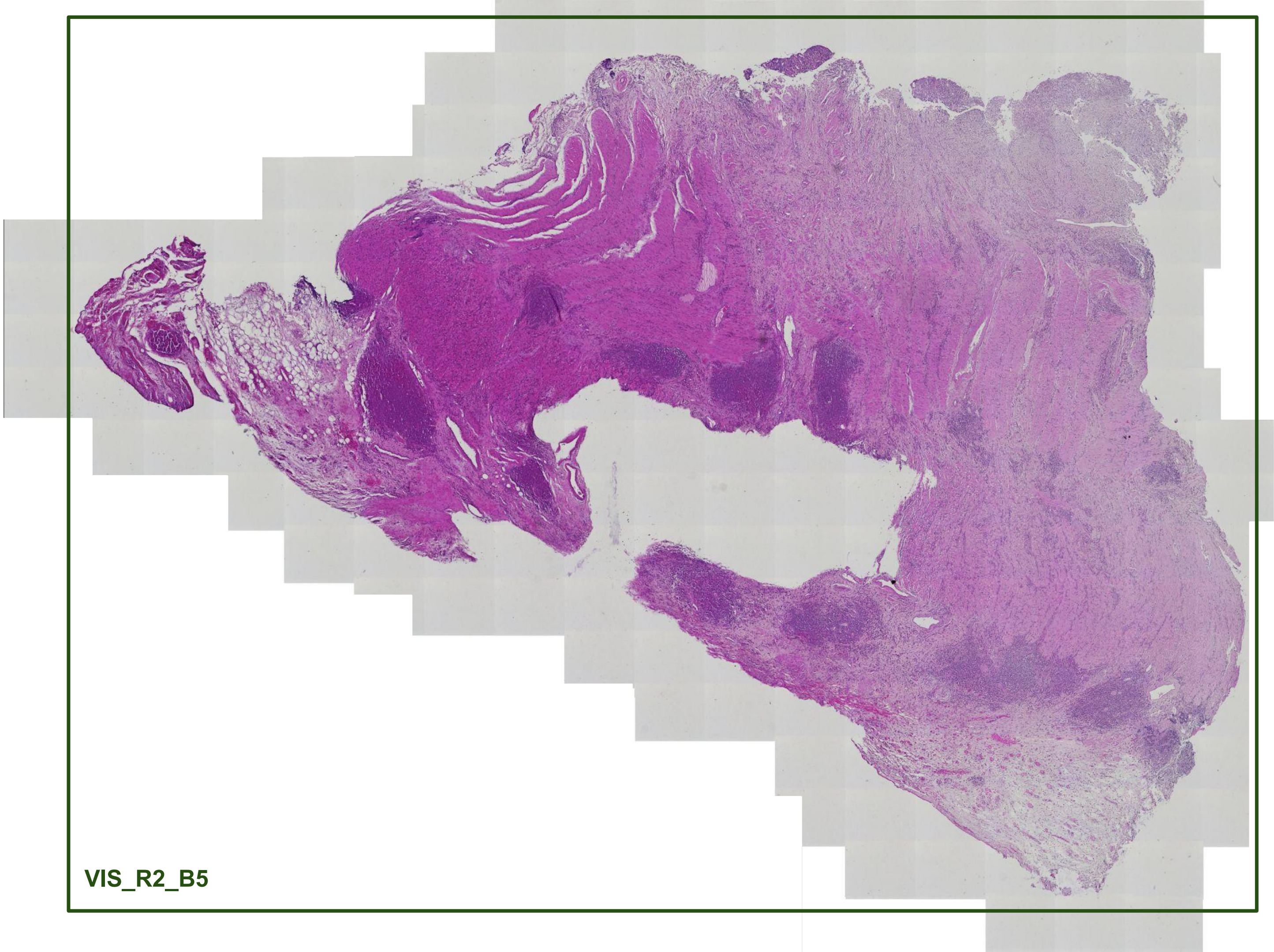

TIP 559

ILEOCOLIC CD
FISTULA

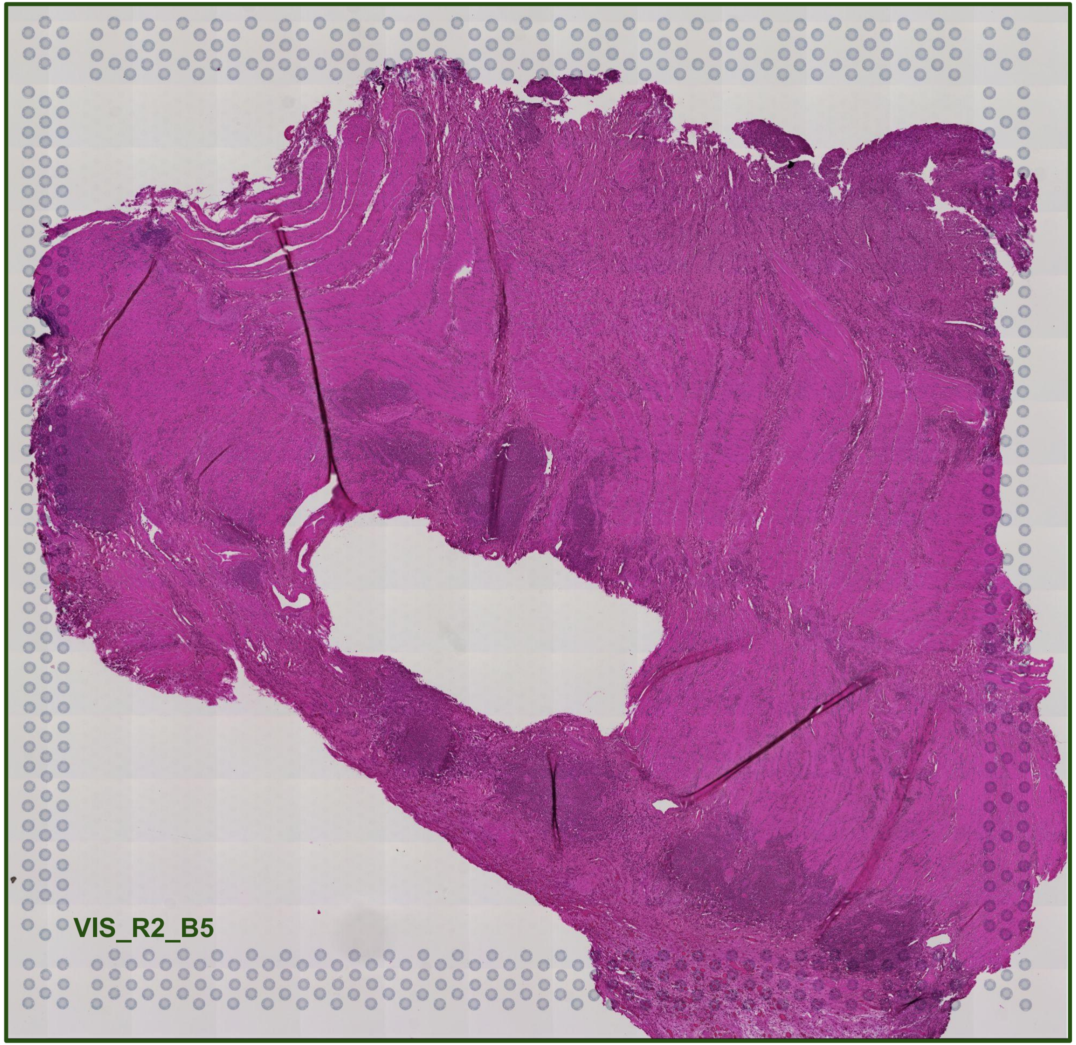

JR_51747_22

DIVERTICULAR FISTULA

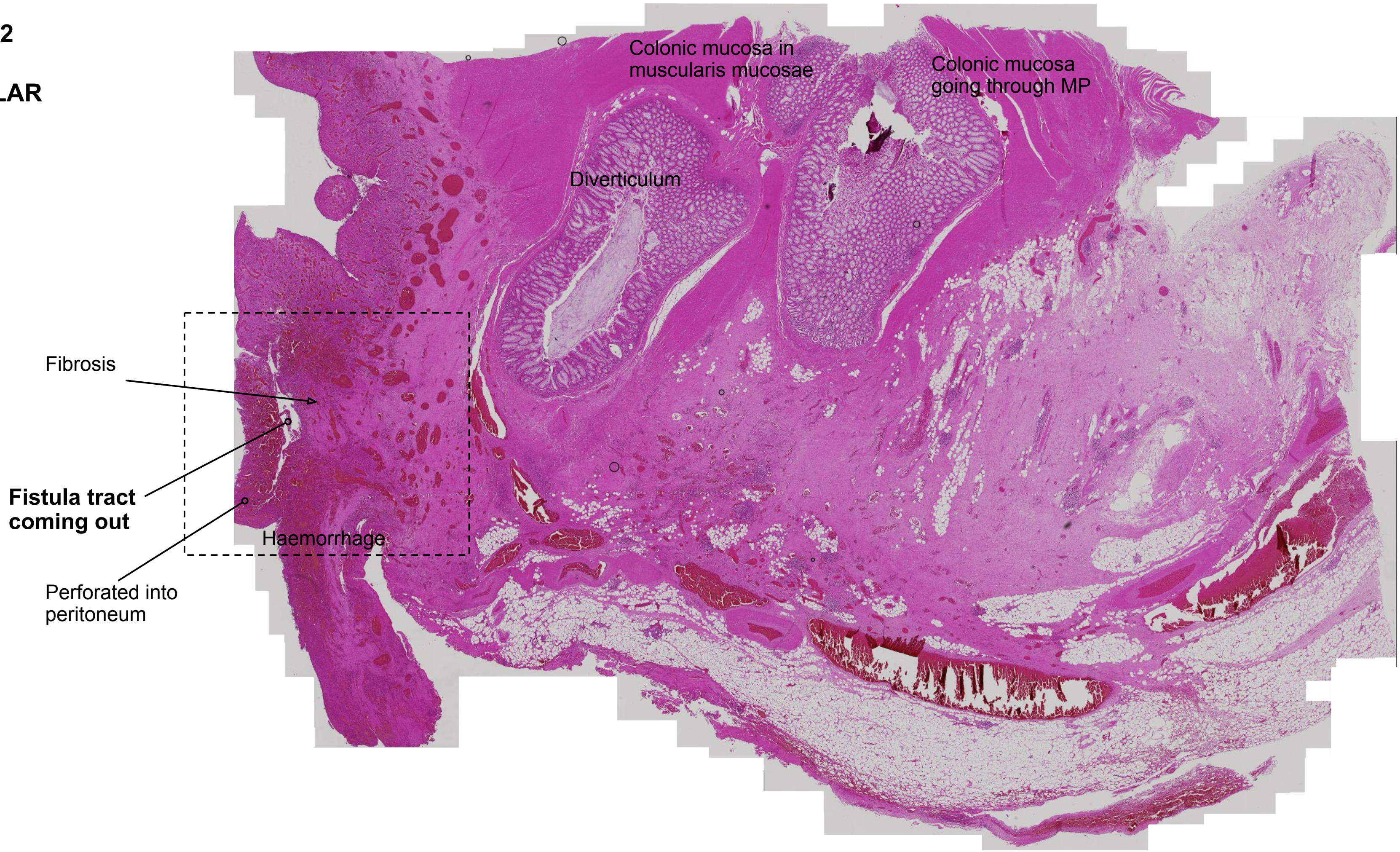

JR_51747_22

DIVERTICULAR FISTULA

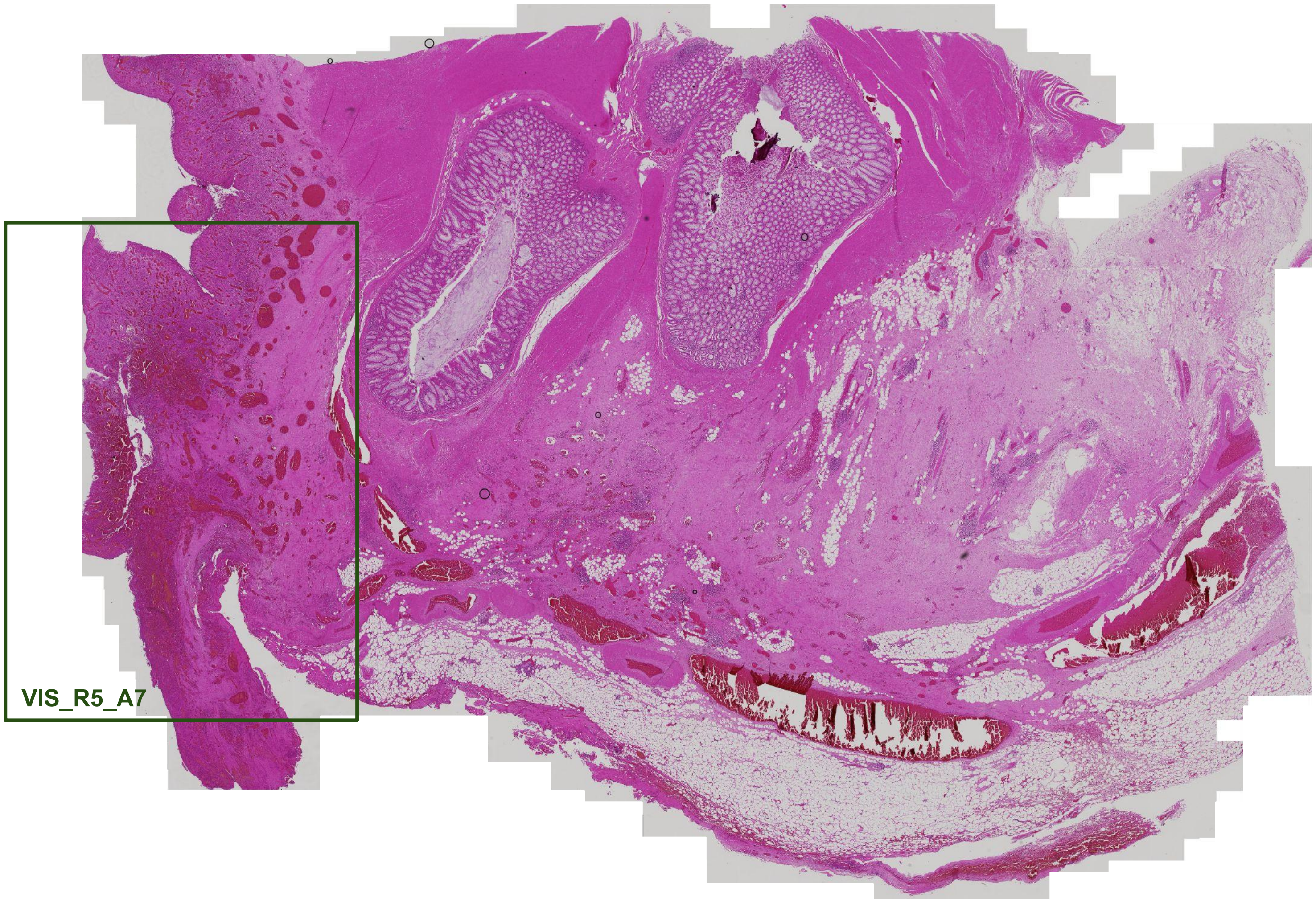

JR_51747_22

DIVERTICULAR
FISTULA

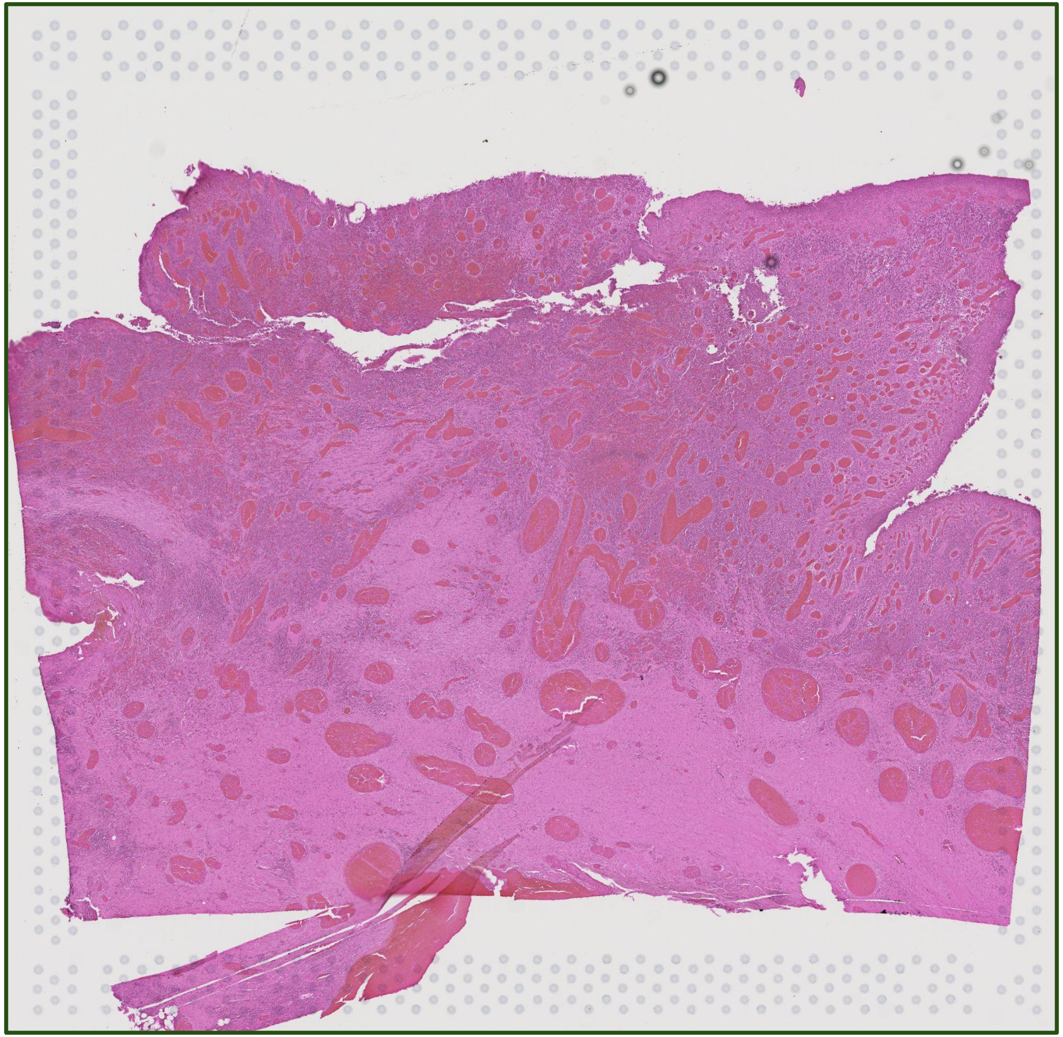

JR_50621_22

DIVERTICULAR FISTULA

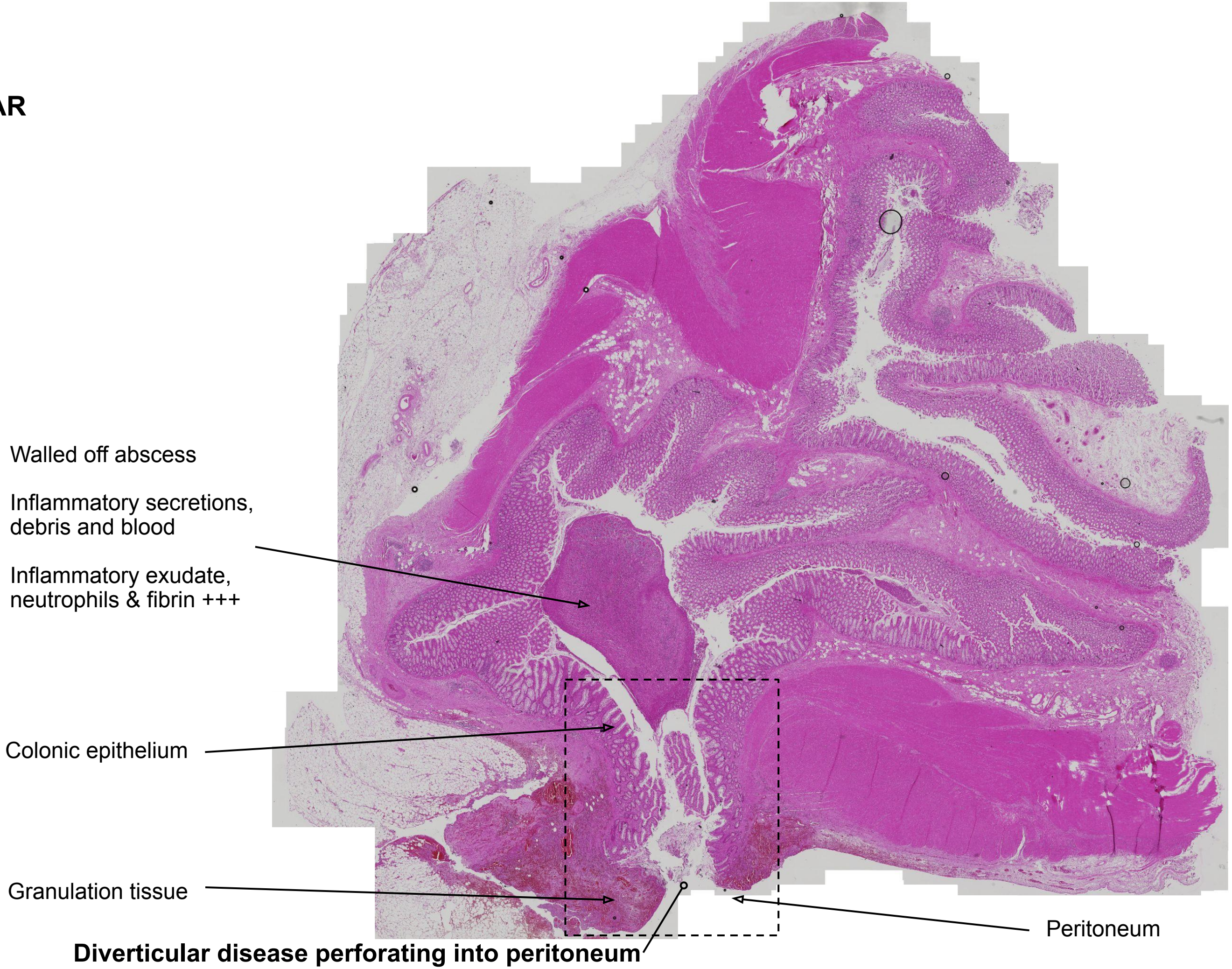

JR_50621_22

DIVERTICULAR
FISTULA

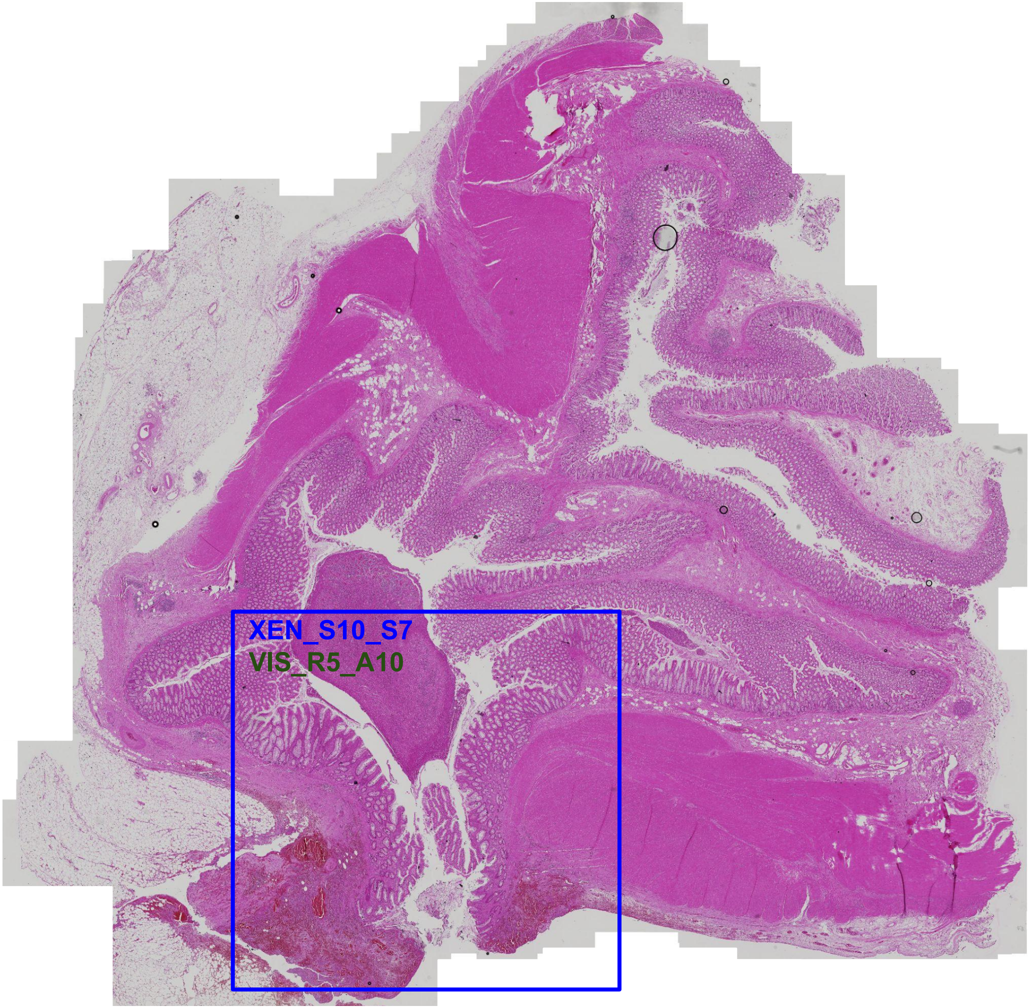

JR_50621_22

DIVERTICULAR FISTULA

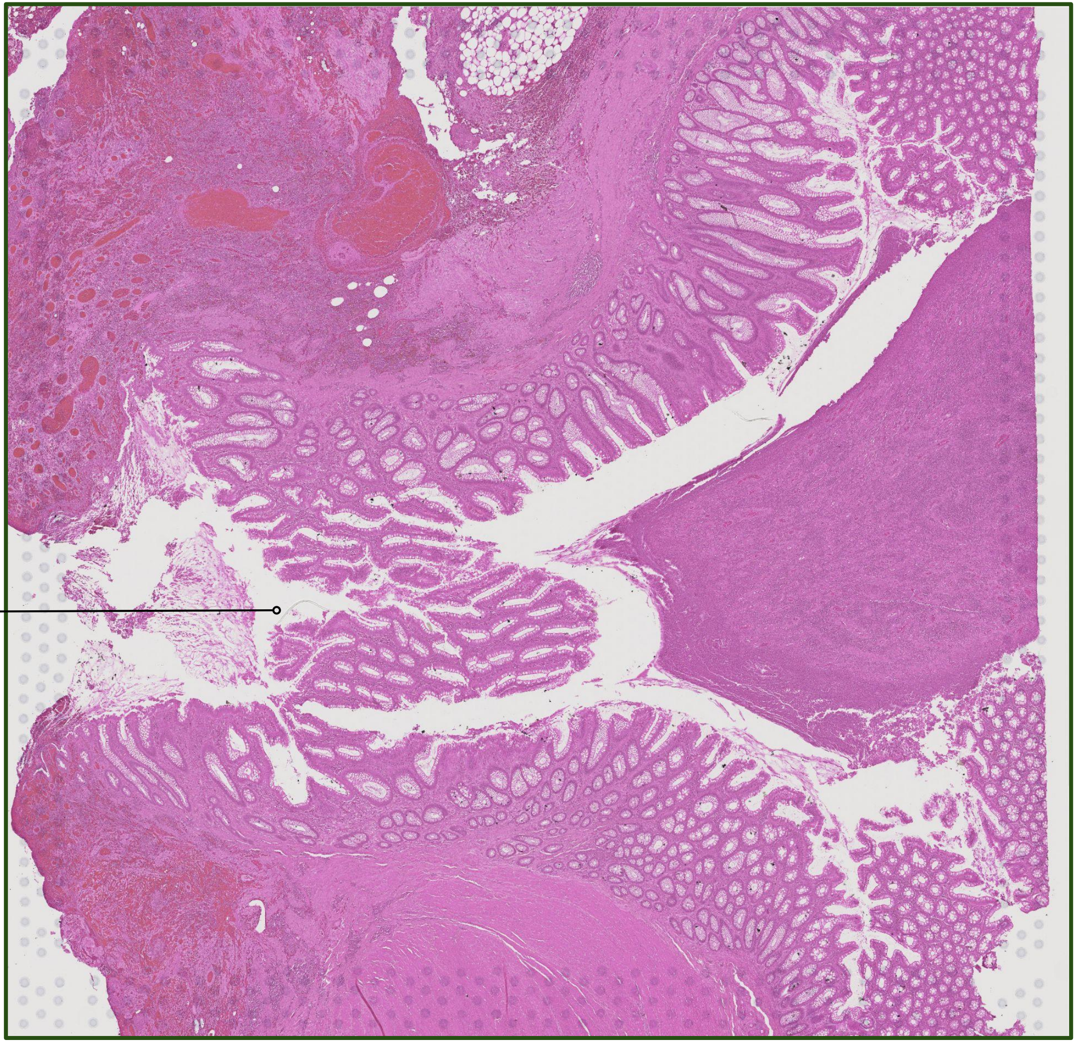
Fistula

JR_41618_20

DIVERTICULAR FISTULA

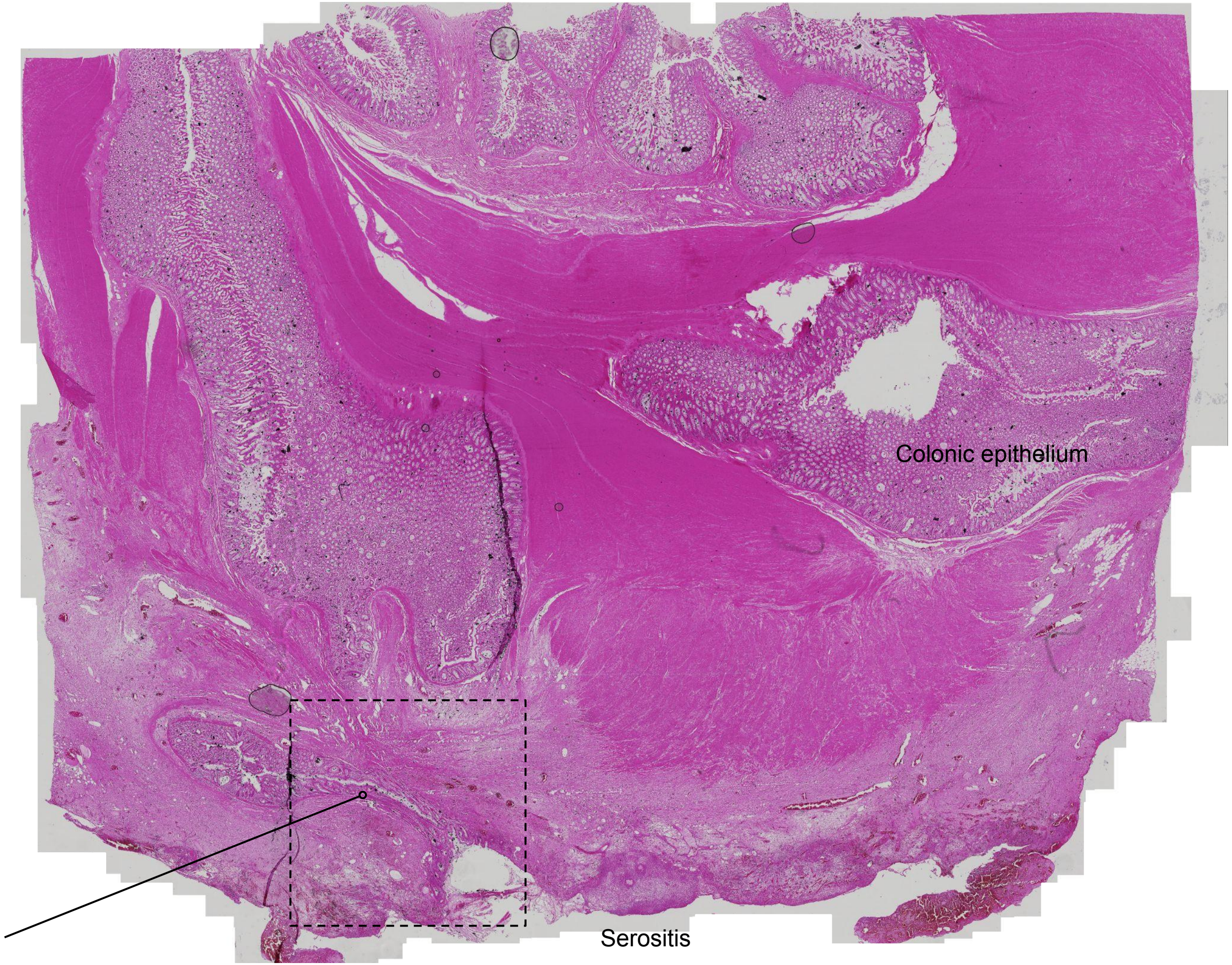

Granulation lined fistula tract

JR_41618_20

DIVERTICULAR
FISTULA

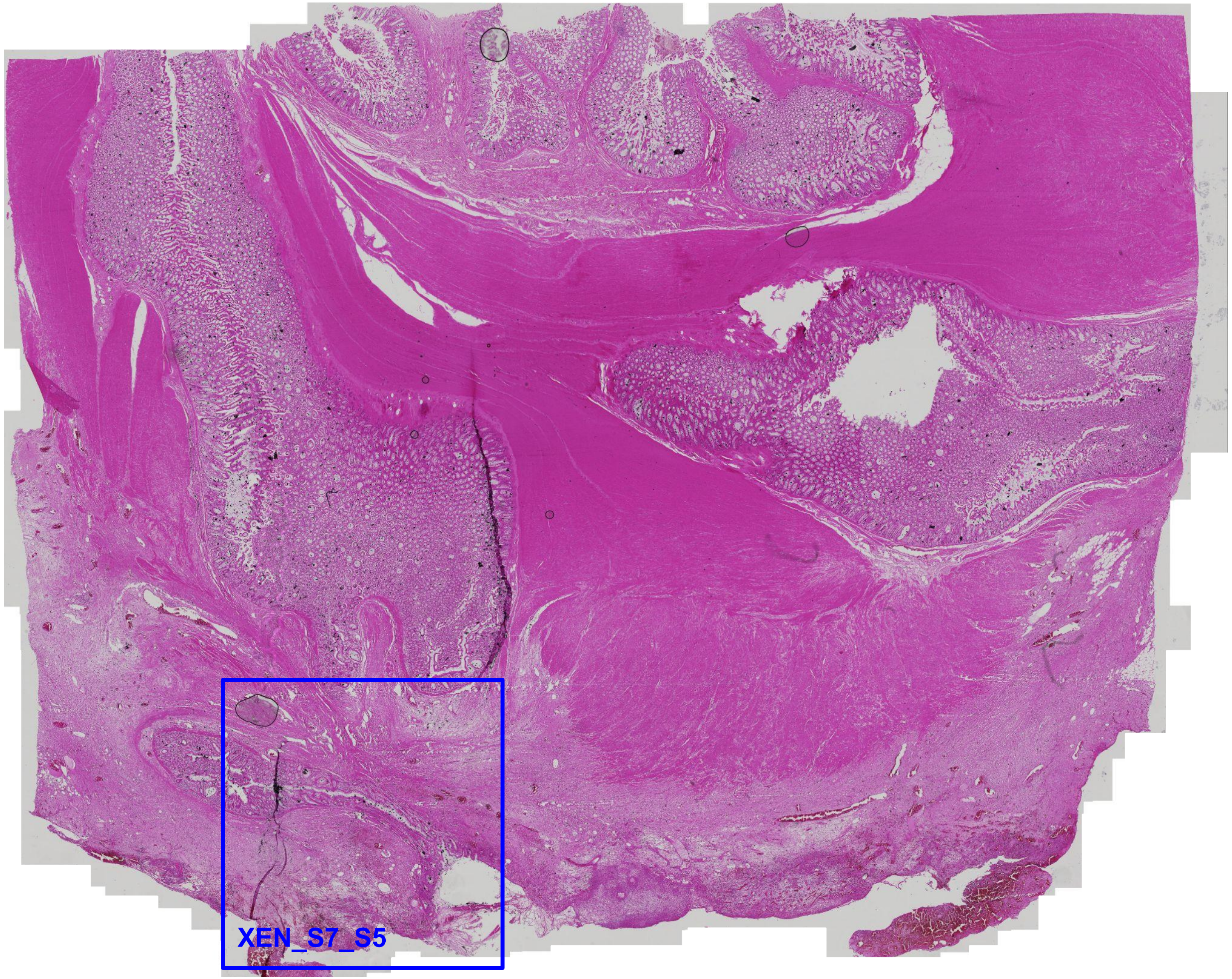

JR 9174_22

DIVERTICULAR FISTULA

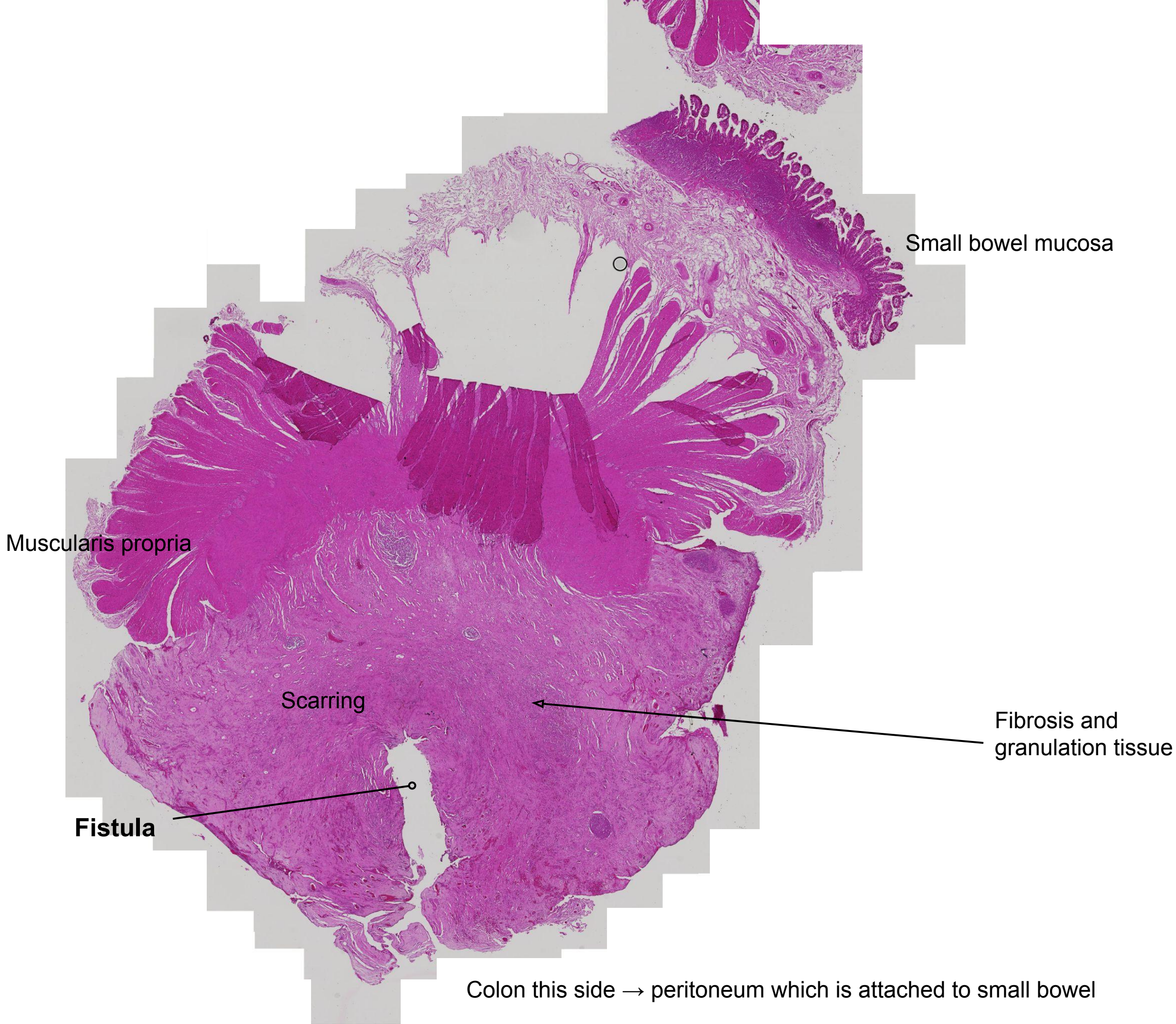

JR 9174_22

DIVERTICULAR FISTULA

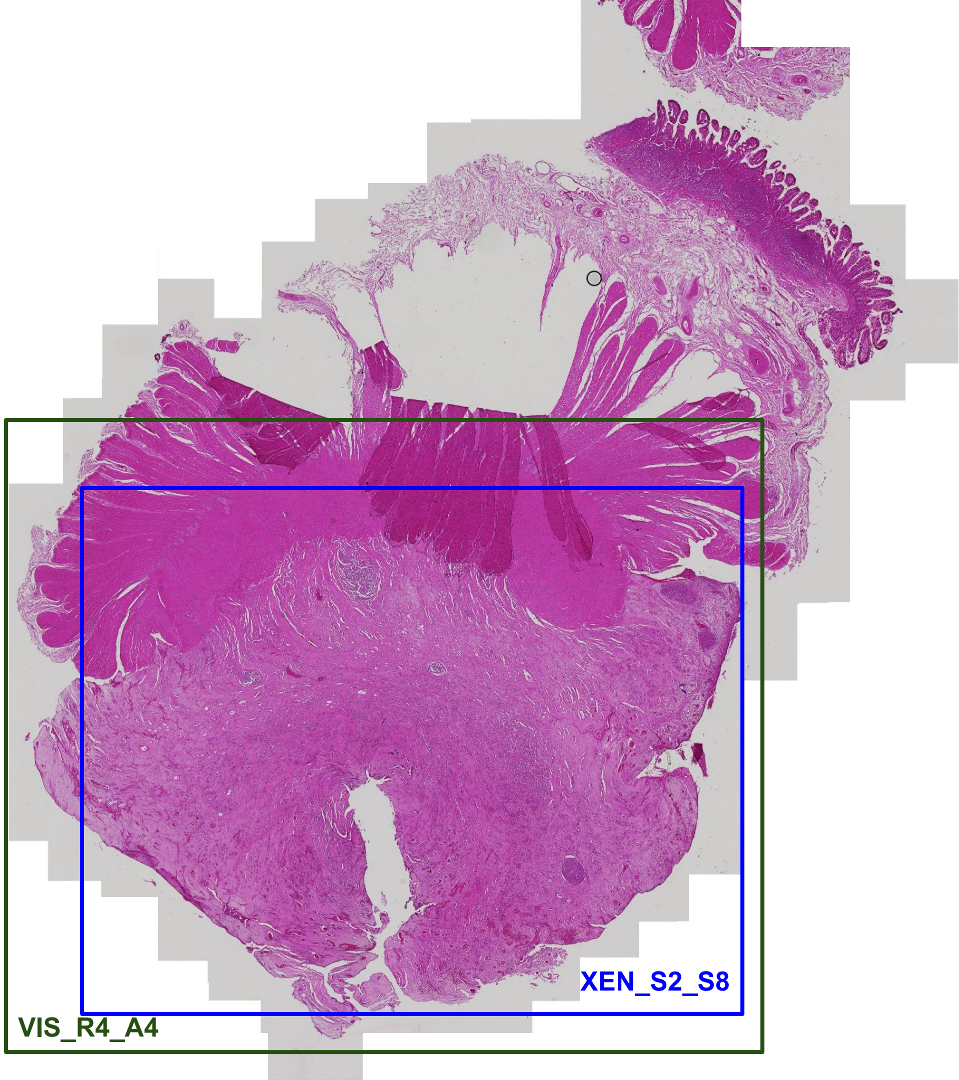

JR 9174_22

DIVERTICULAR
FISTULA

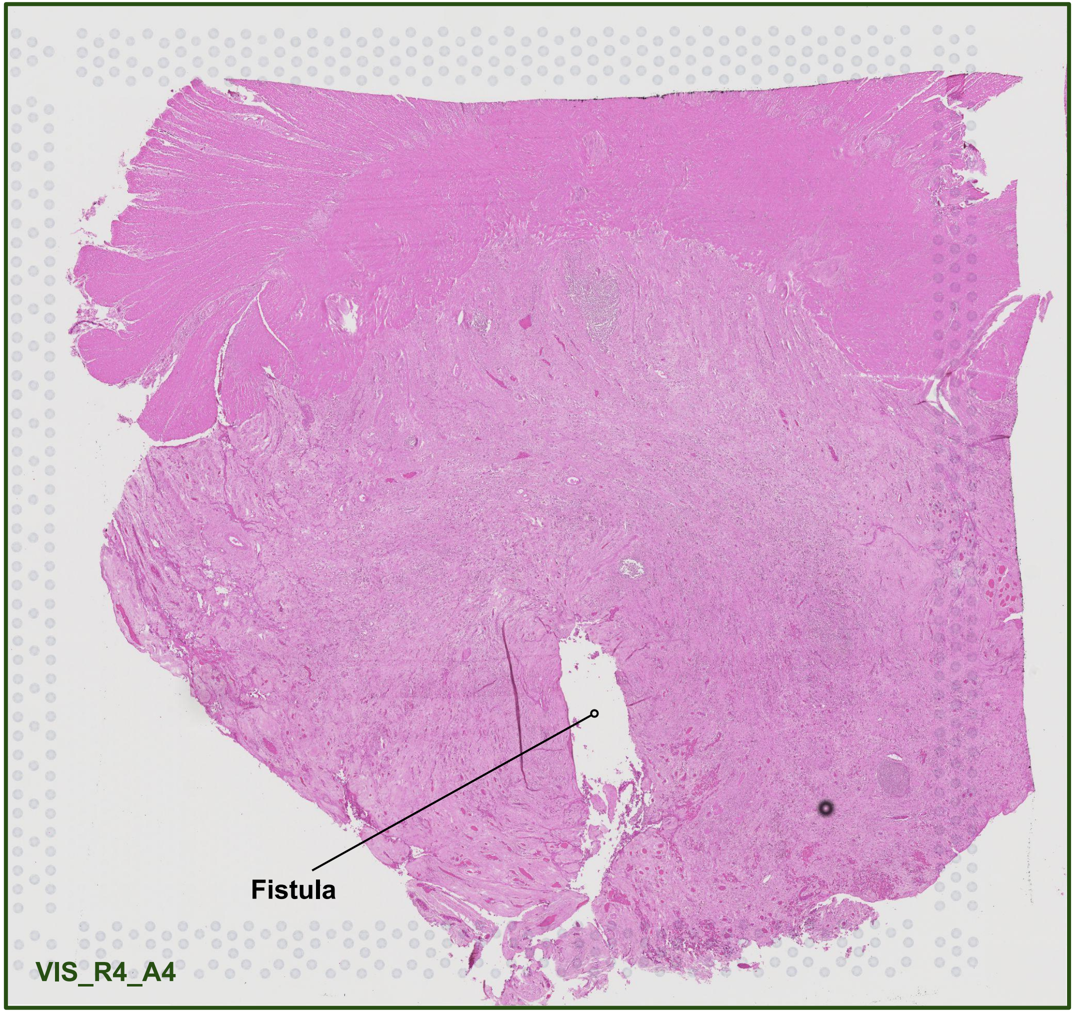

JR_18170_21

DIVERTICULAR FISTULA

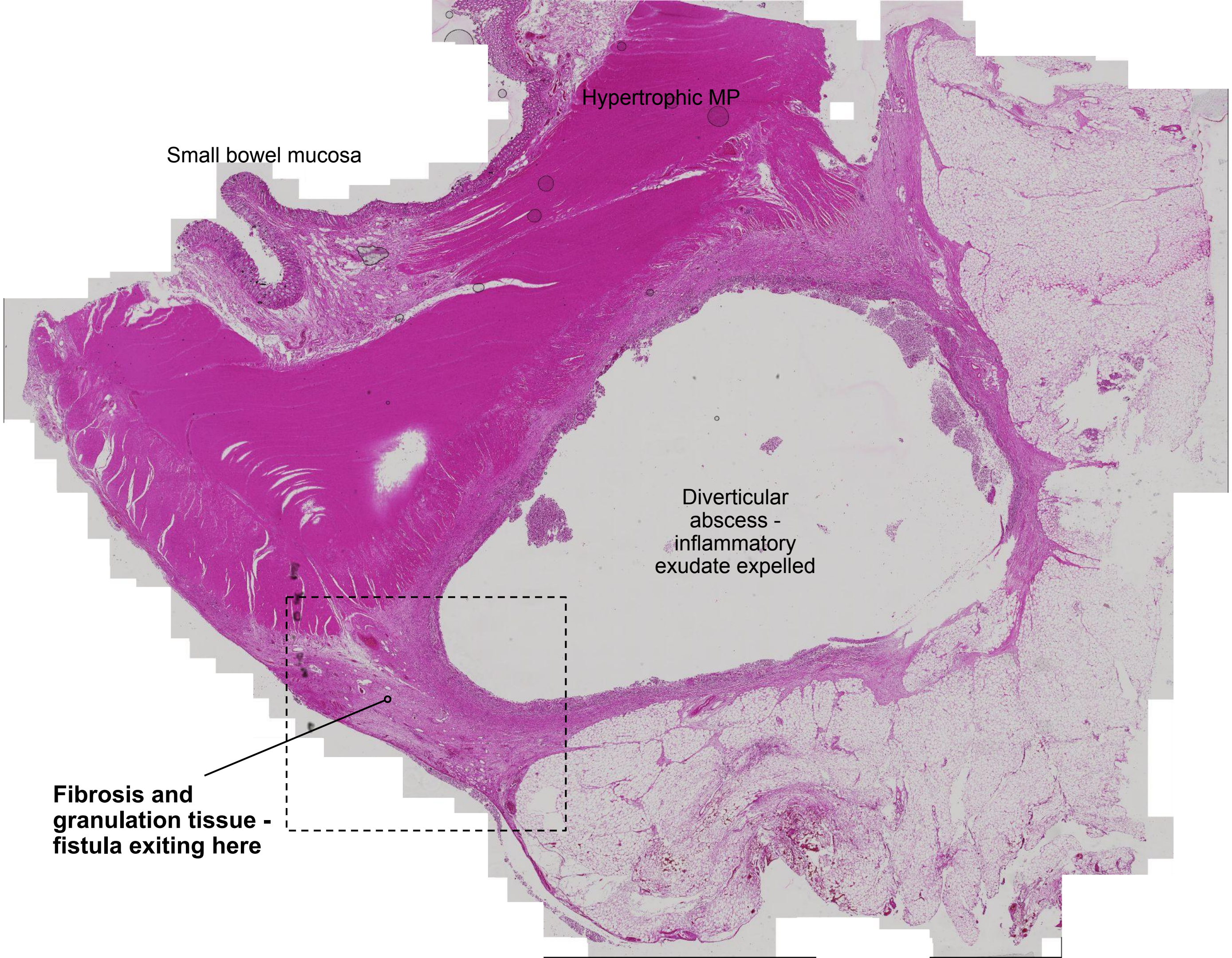

JR_18170_21

DIVERTICULAR
FISTULA

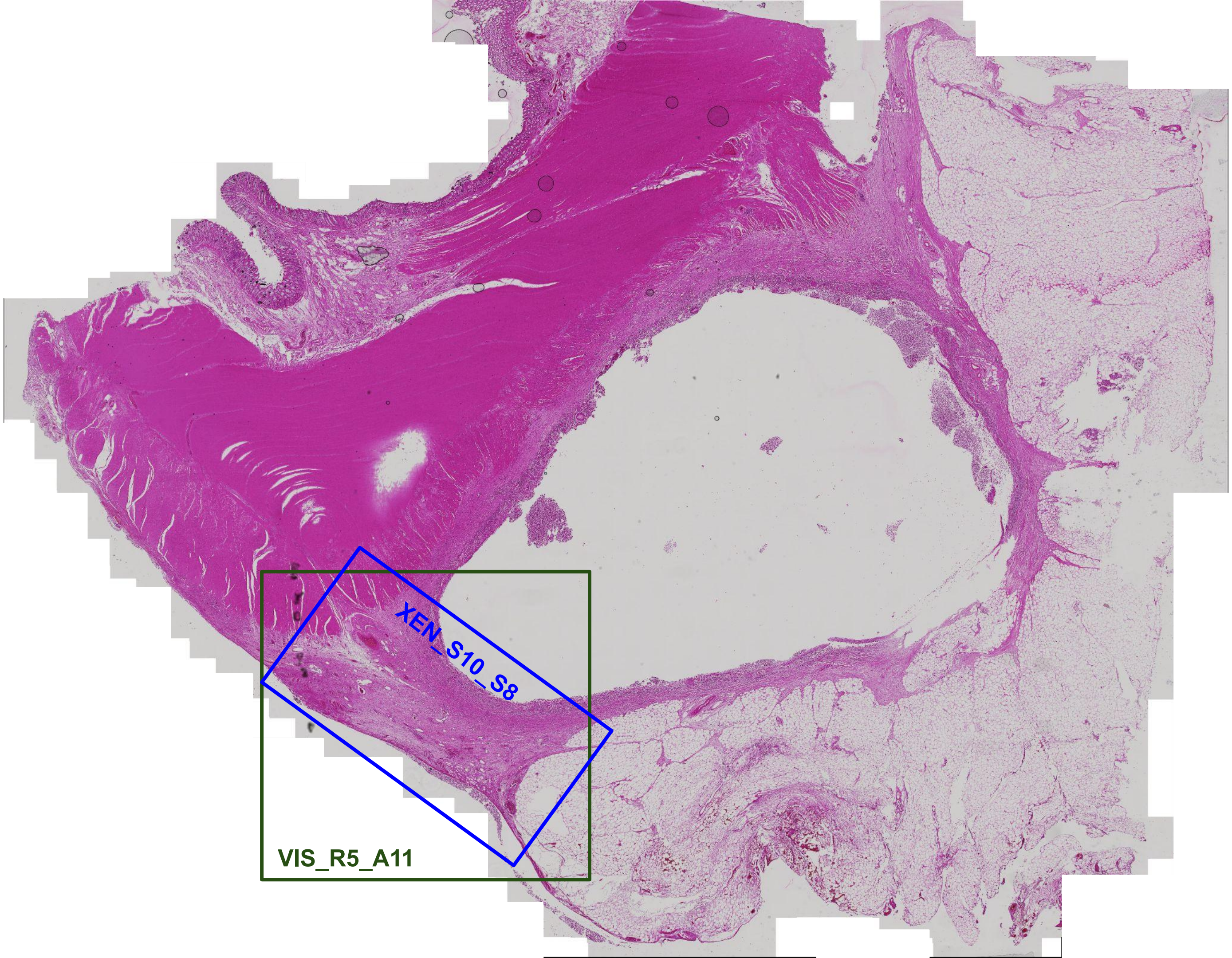

JR_18170_21

DIVERTICULAR
FISTULA

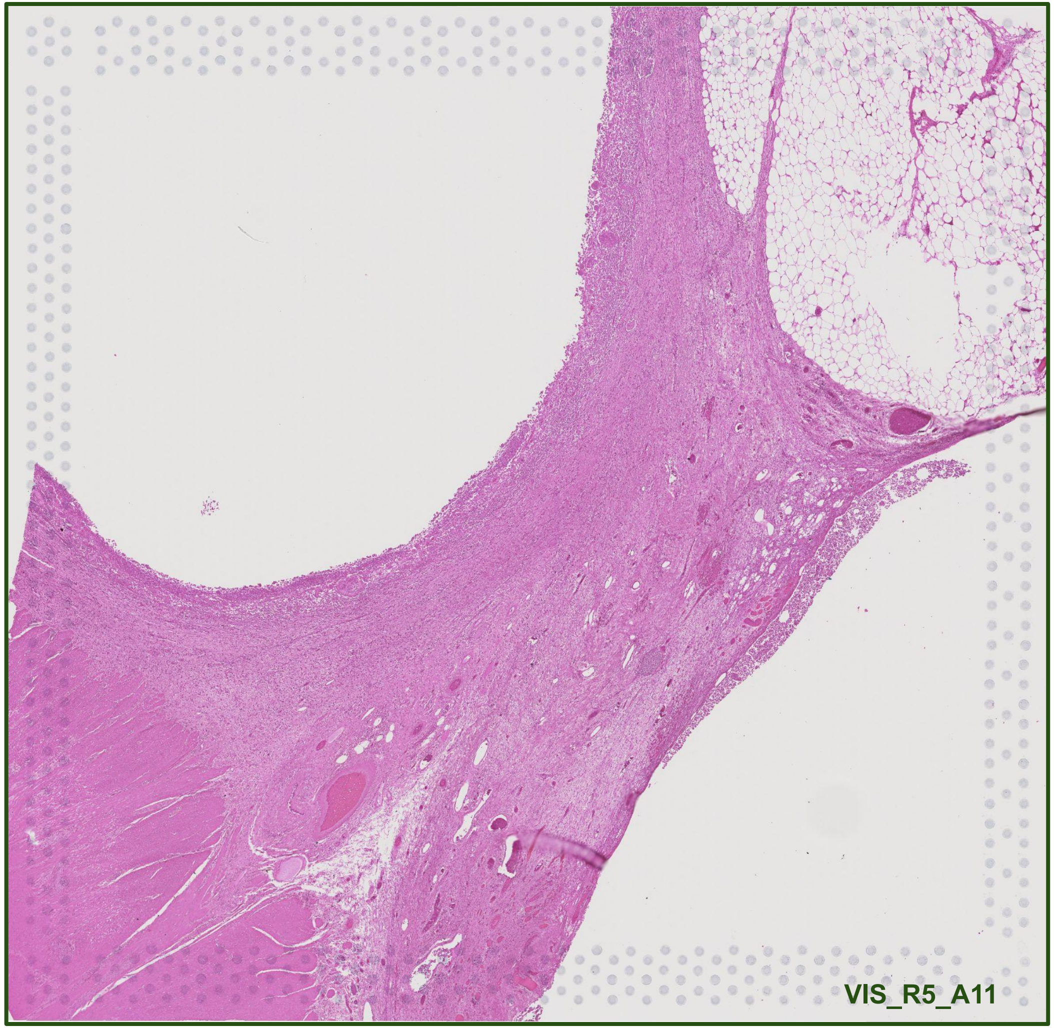

JR_50699_24

DIVERTICULAR FISTULA

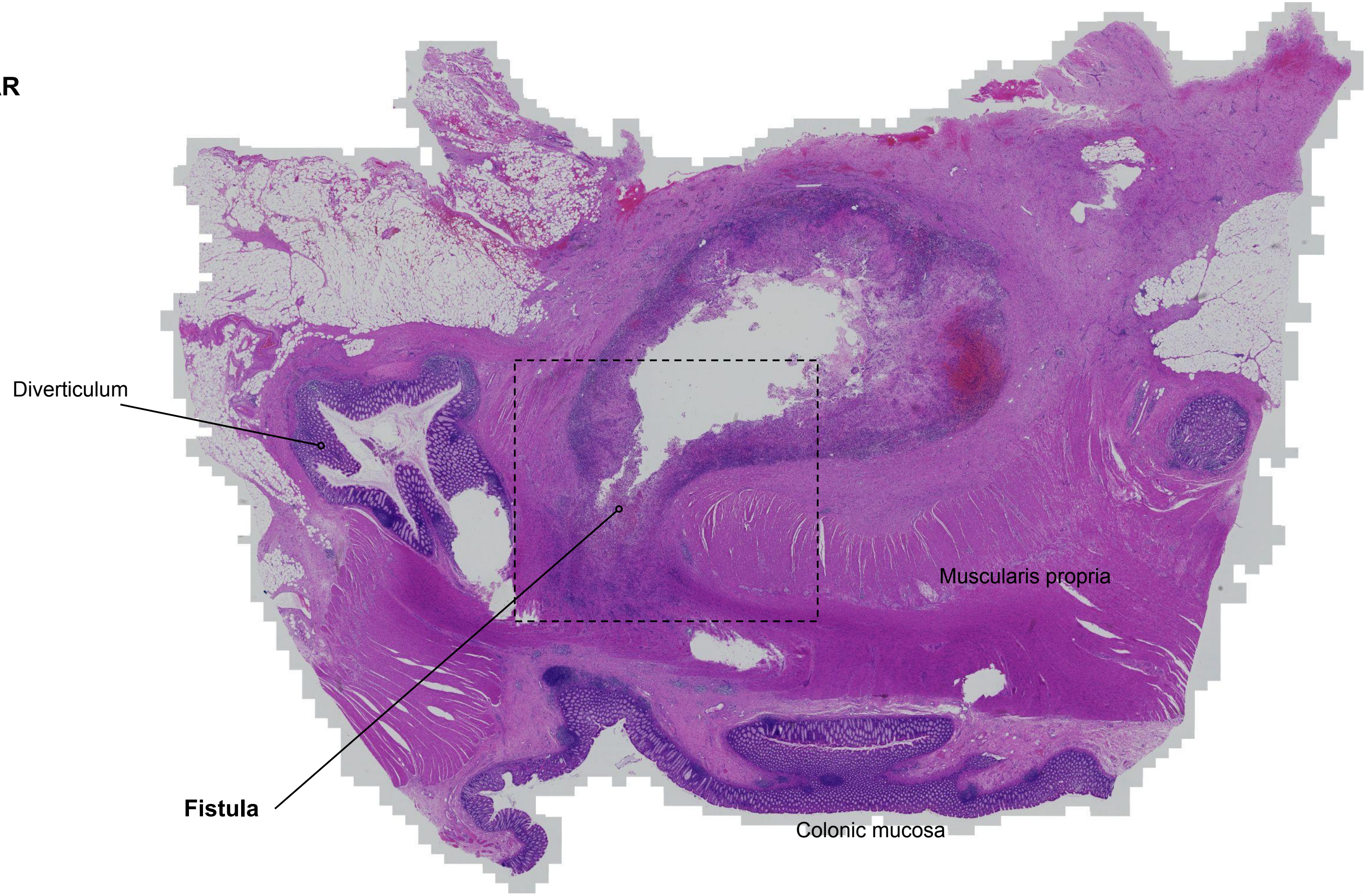

JR_50699_24

DIVERTICULAR
FISTULA

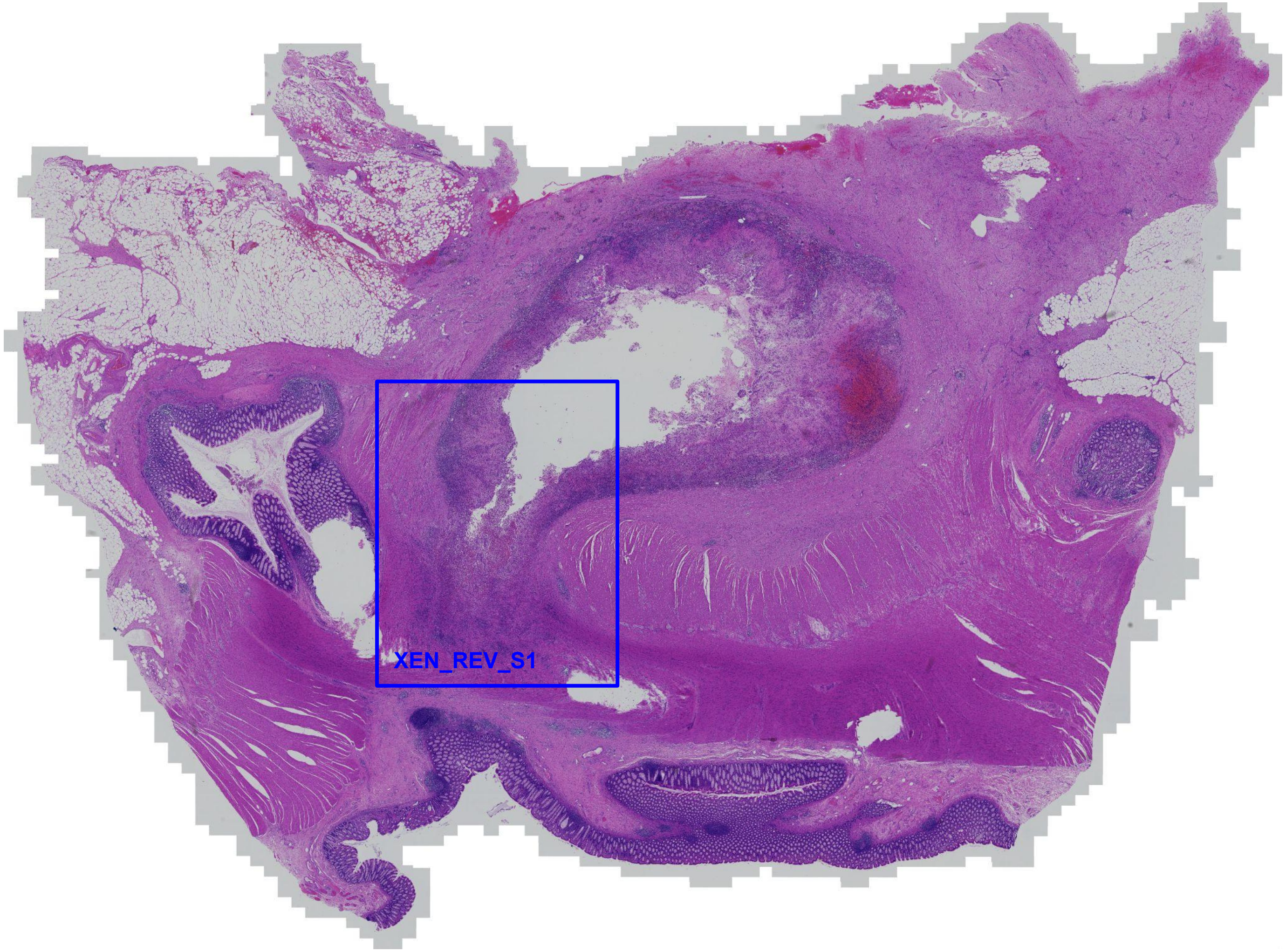

JR_22123_24

DIVERTICULAR FISTULA

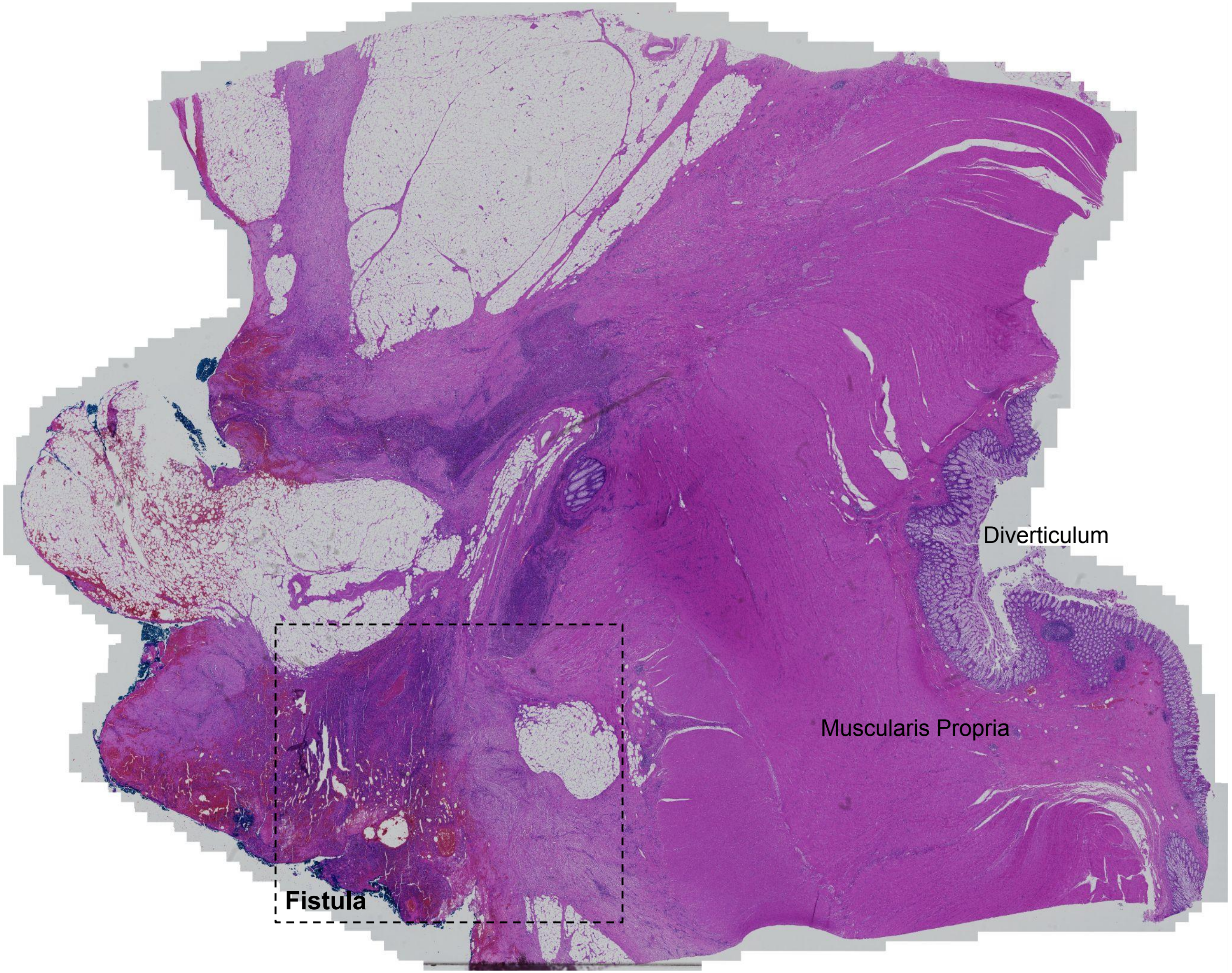

JR_22123_24

DIVERTICULAR FISTULA

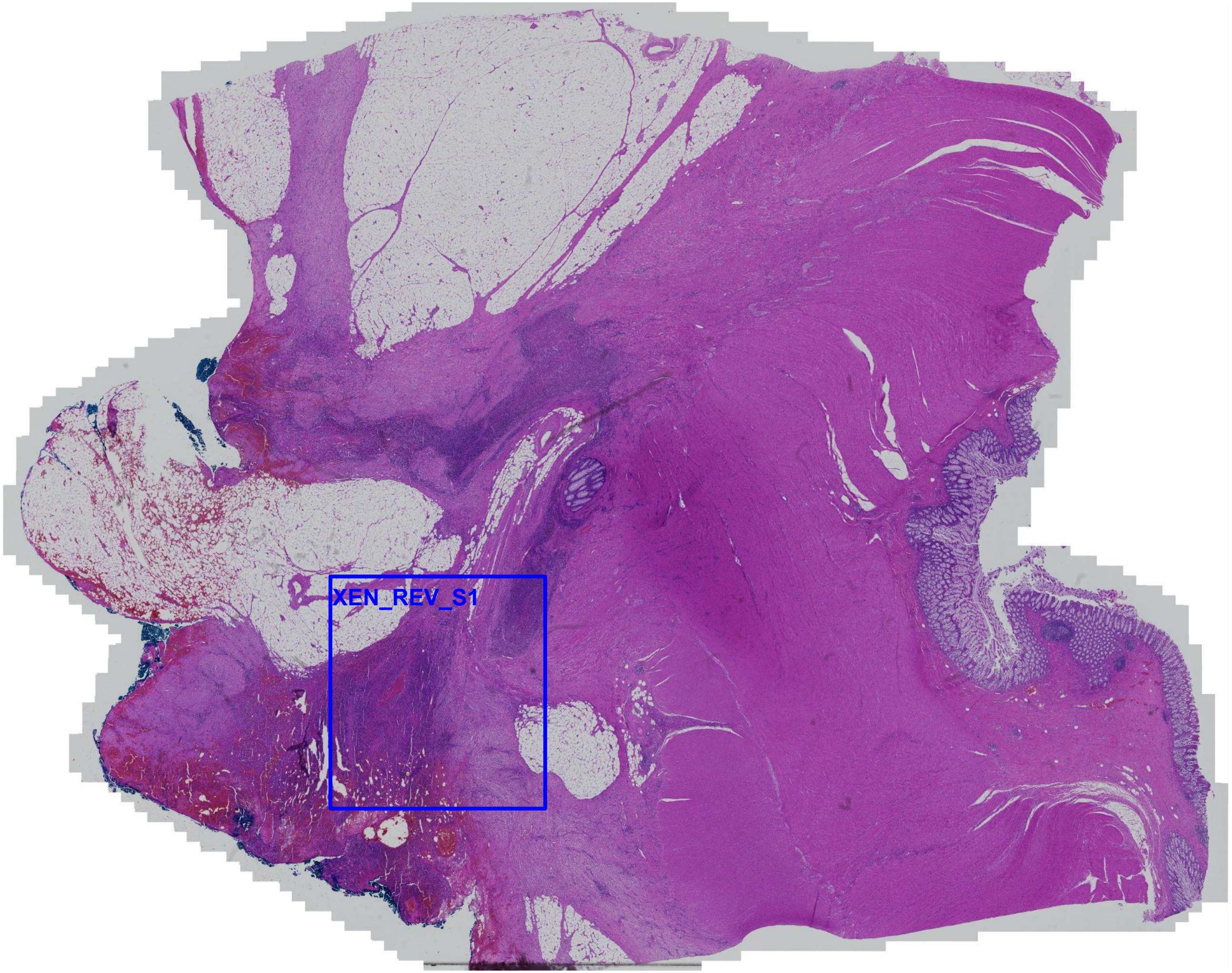

JR_37904_24

DIVERTICULAR FISTULA

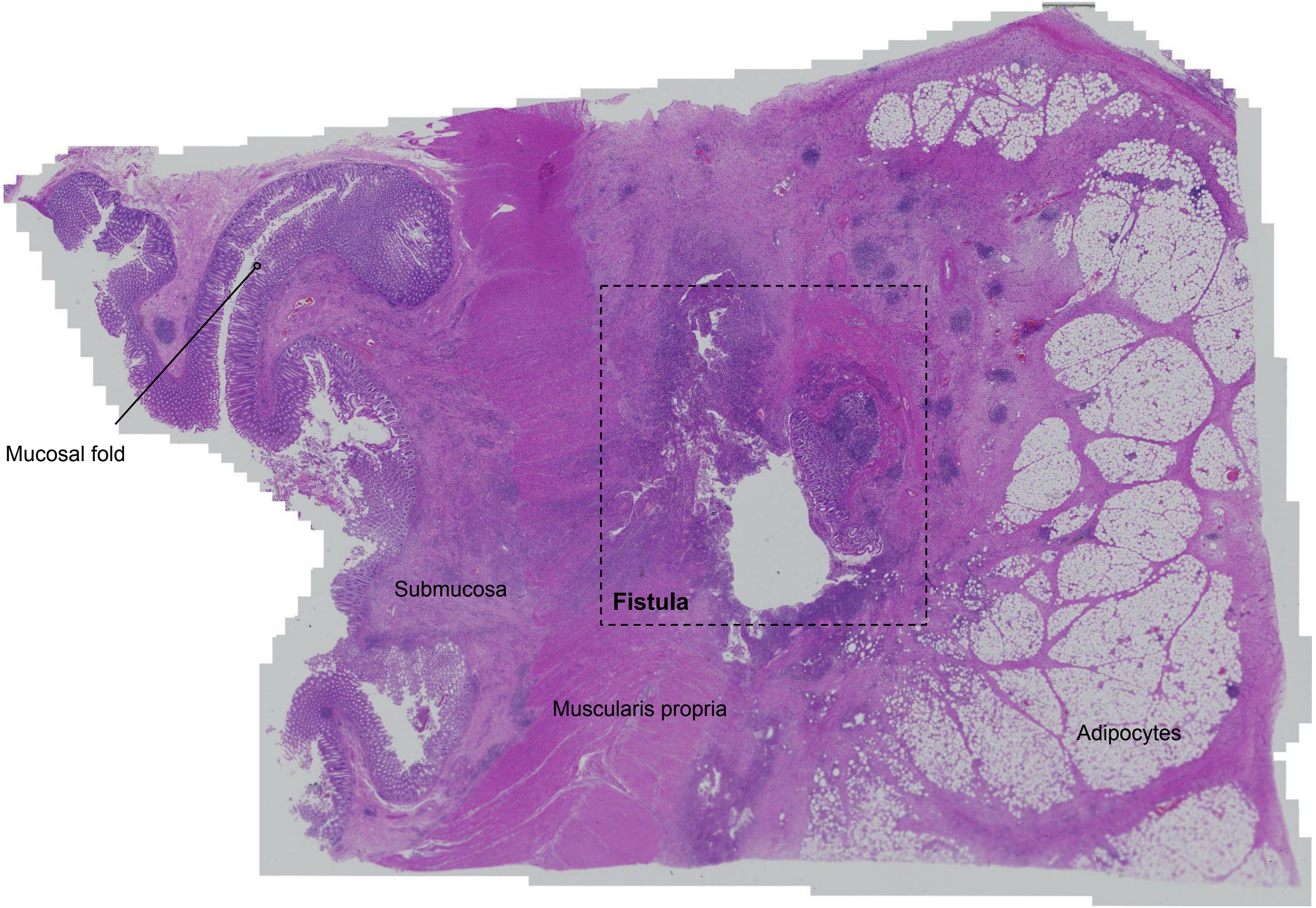

JR_37904_24

DIVERTICULAR FISTULA

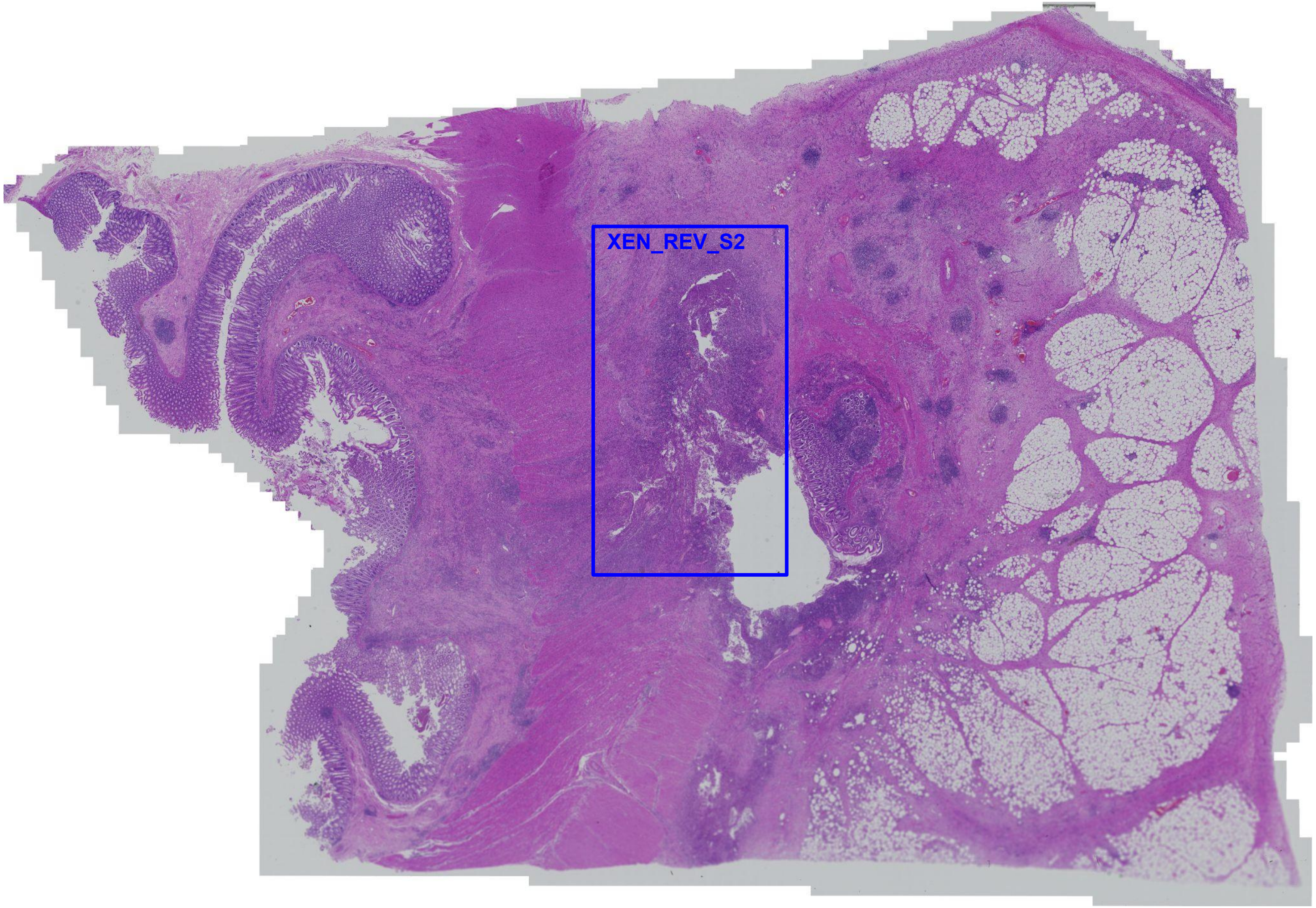

JR_47872_24

DIVERTICULAR FISTULA

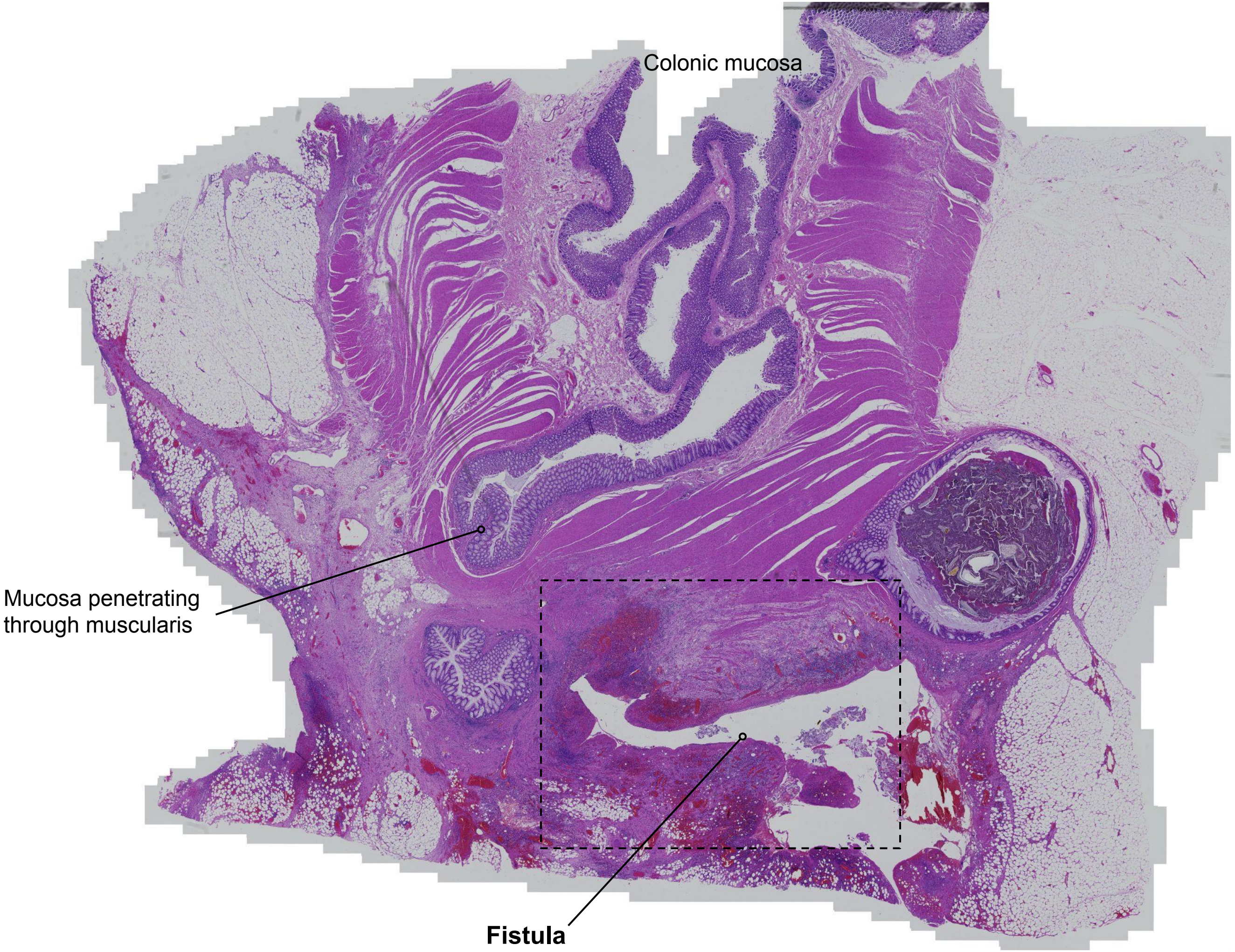

JR_47872_24

DIVERTICULAR
FISTULA

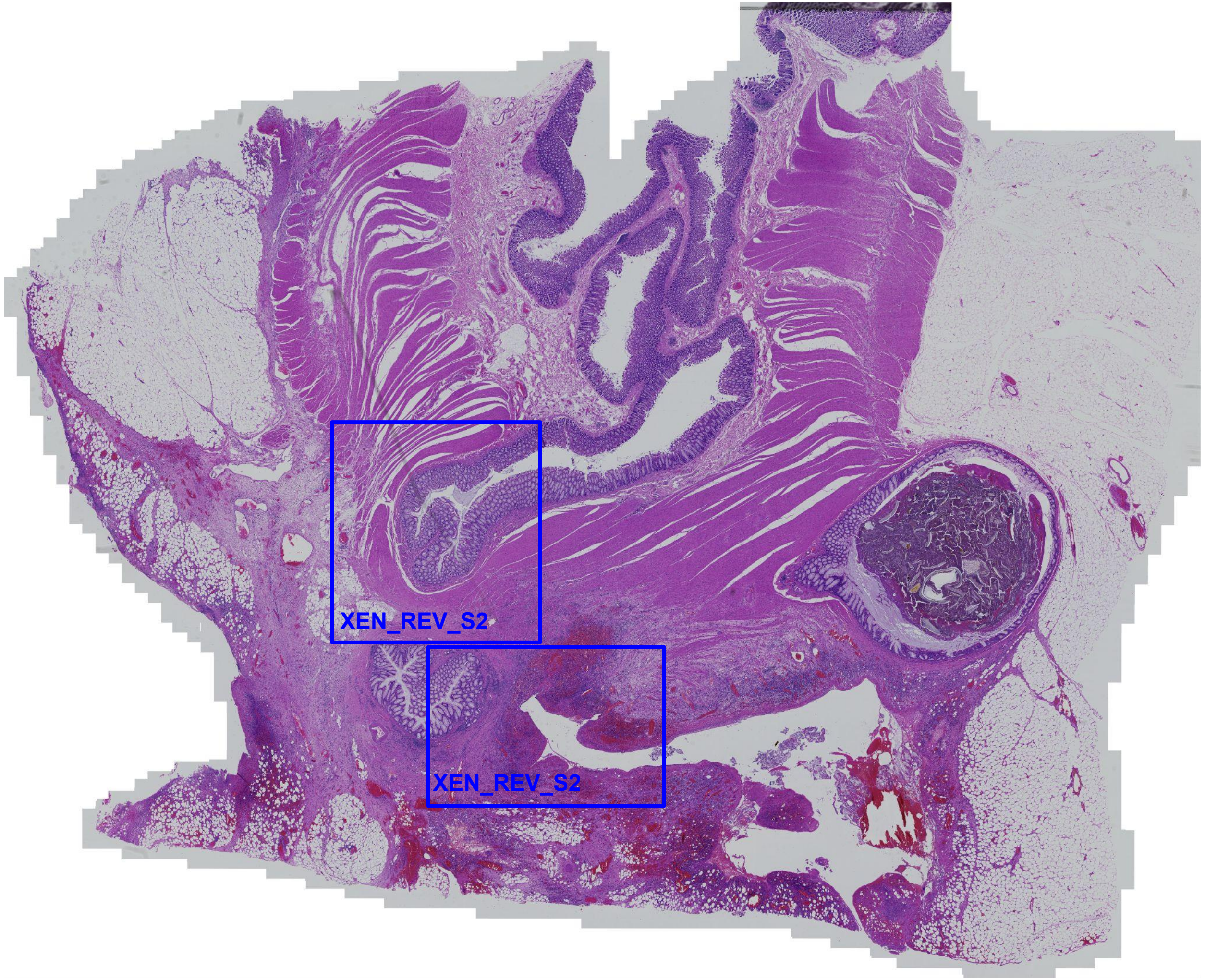

JR_42449_24

DIVERTICULAR FISTULA

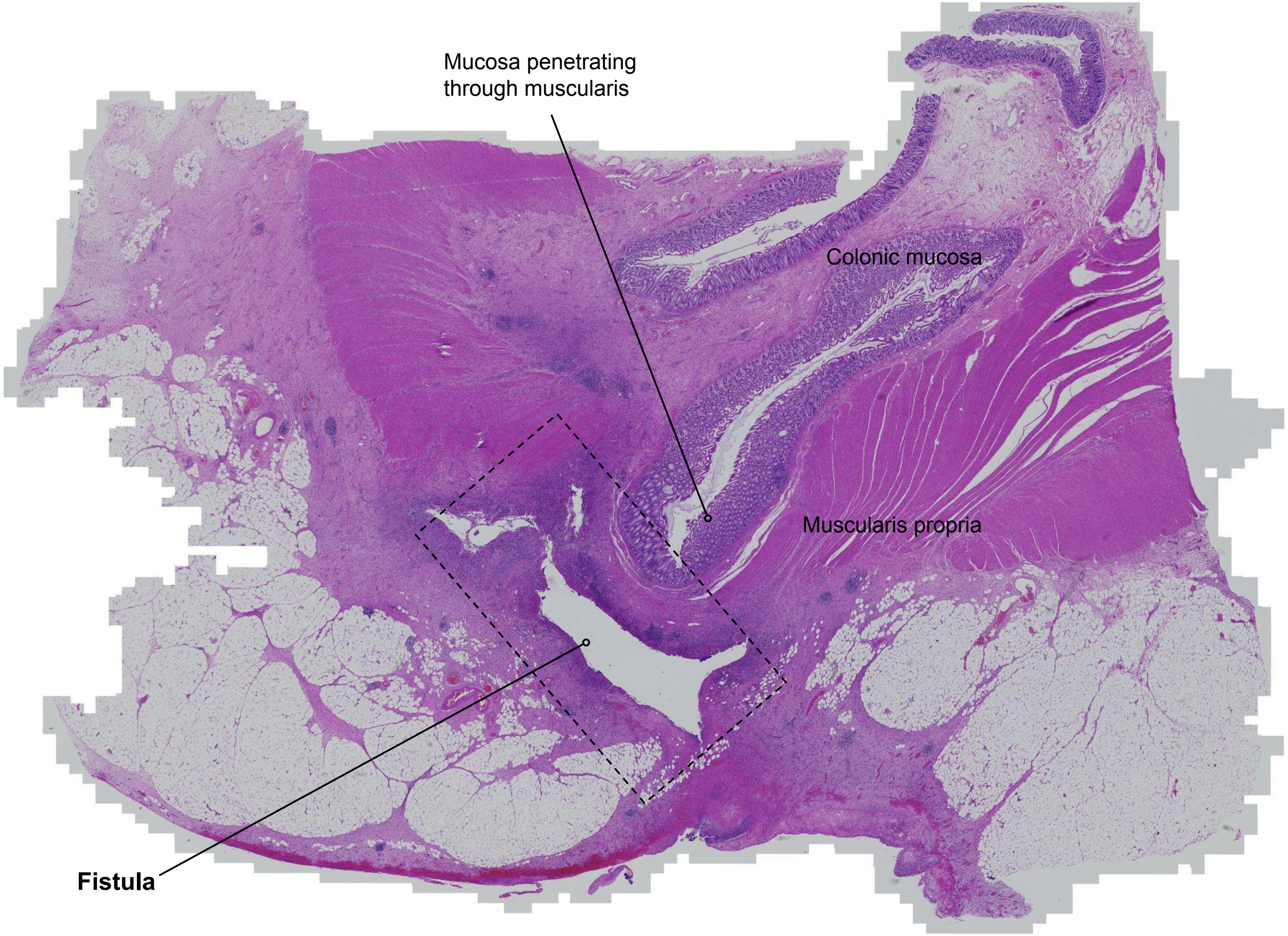

JR_42449_24

DIVERTICULAR FISTULA

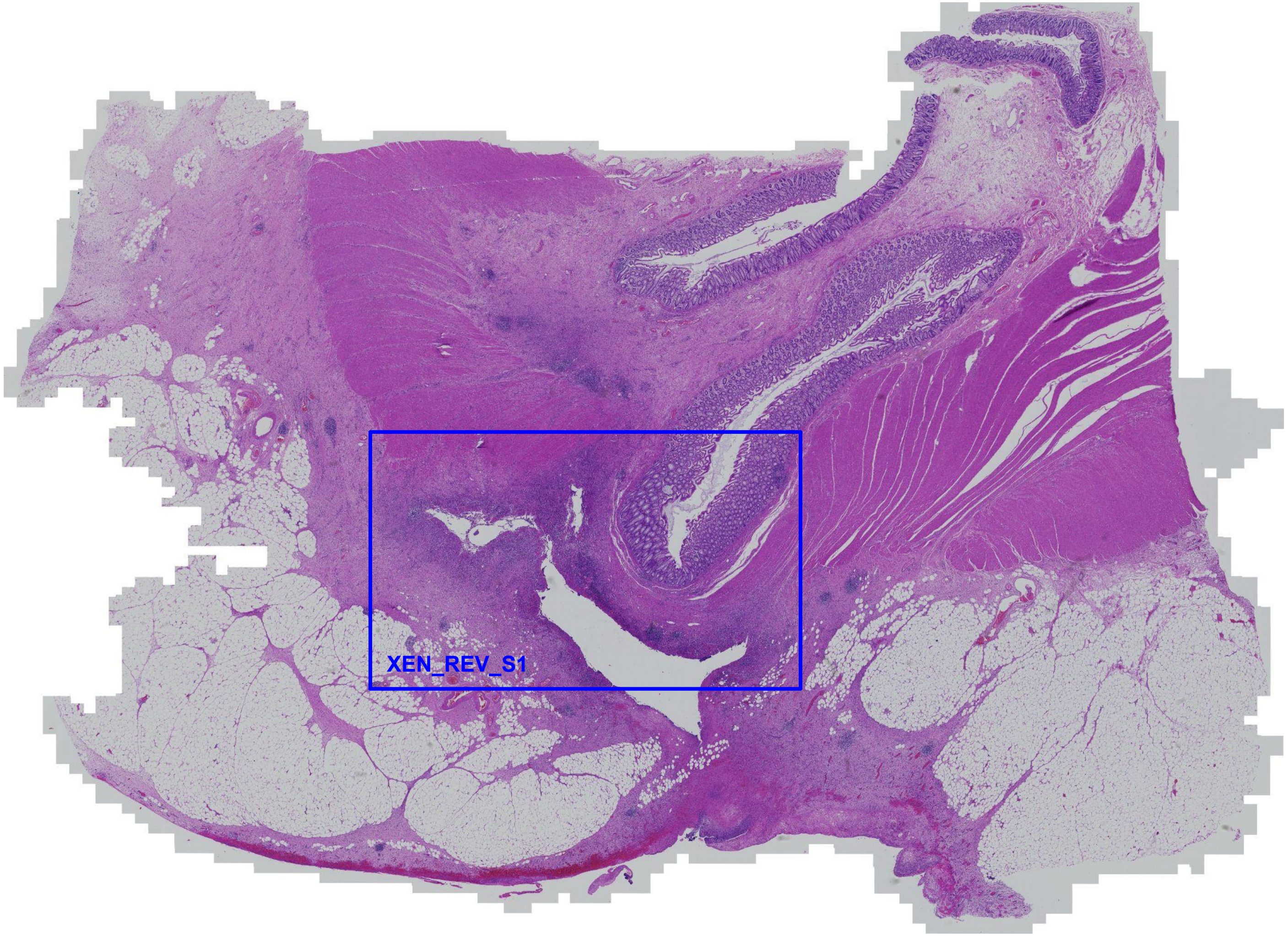

JR_45695_21

PERIANAL NON-CD FISTULA

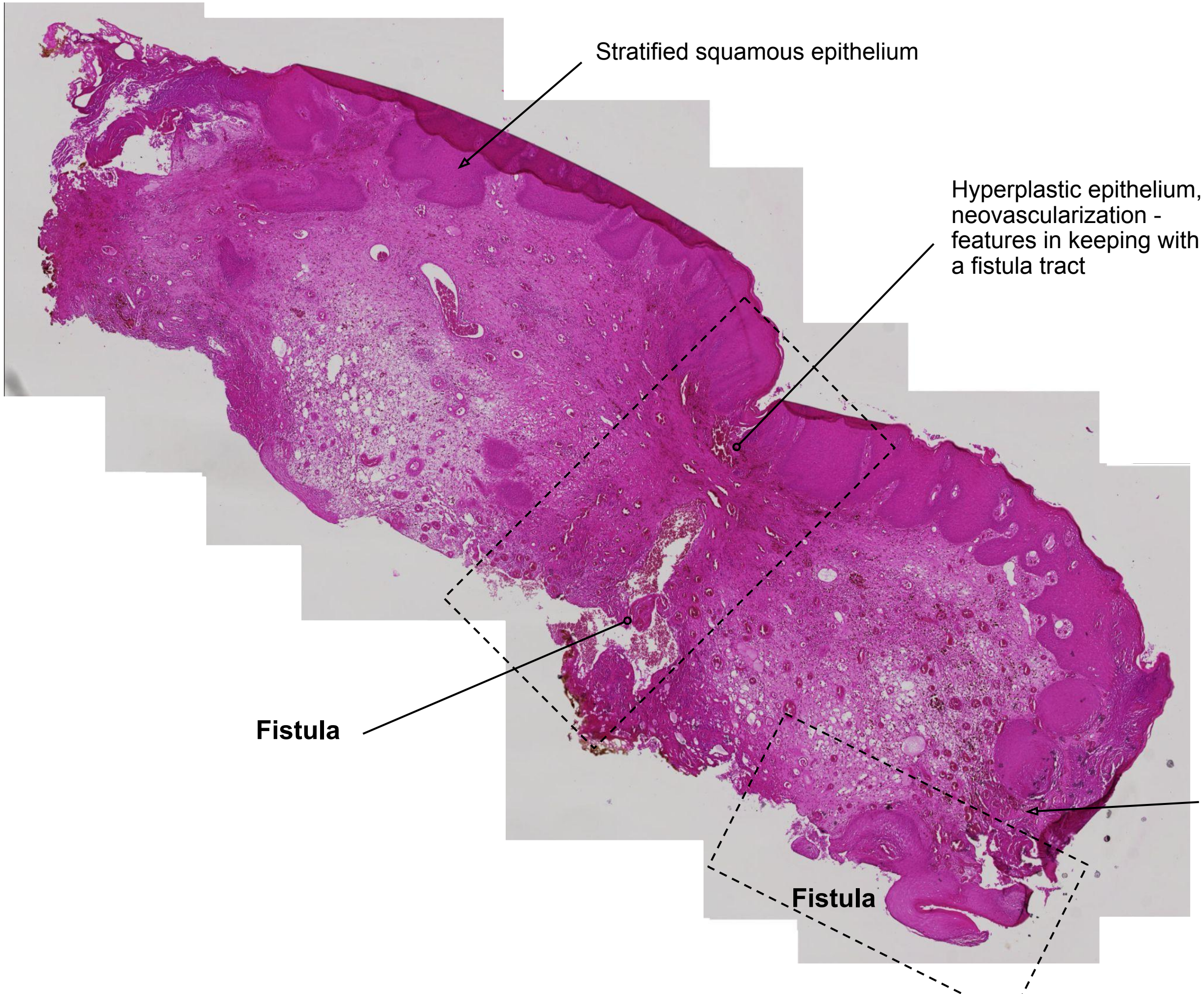

JR_45695_21

PERIANAL NON-CD
FISTULA

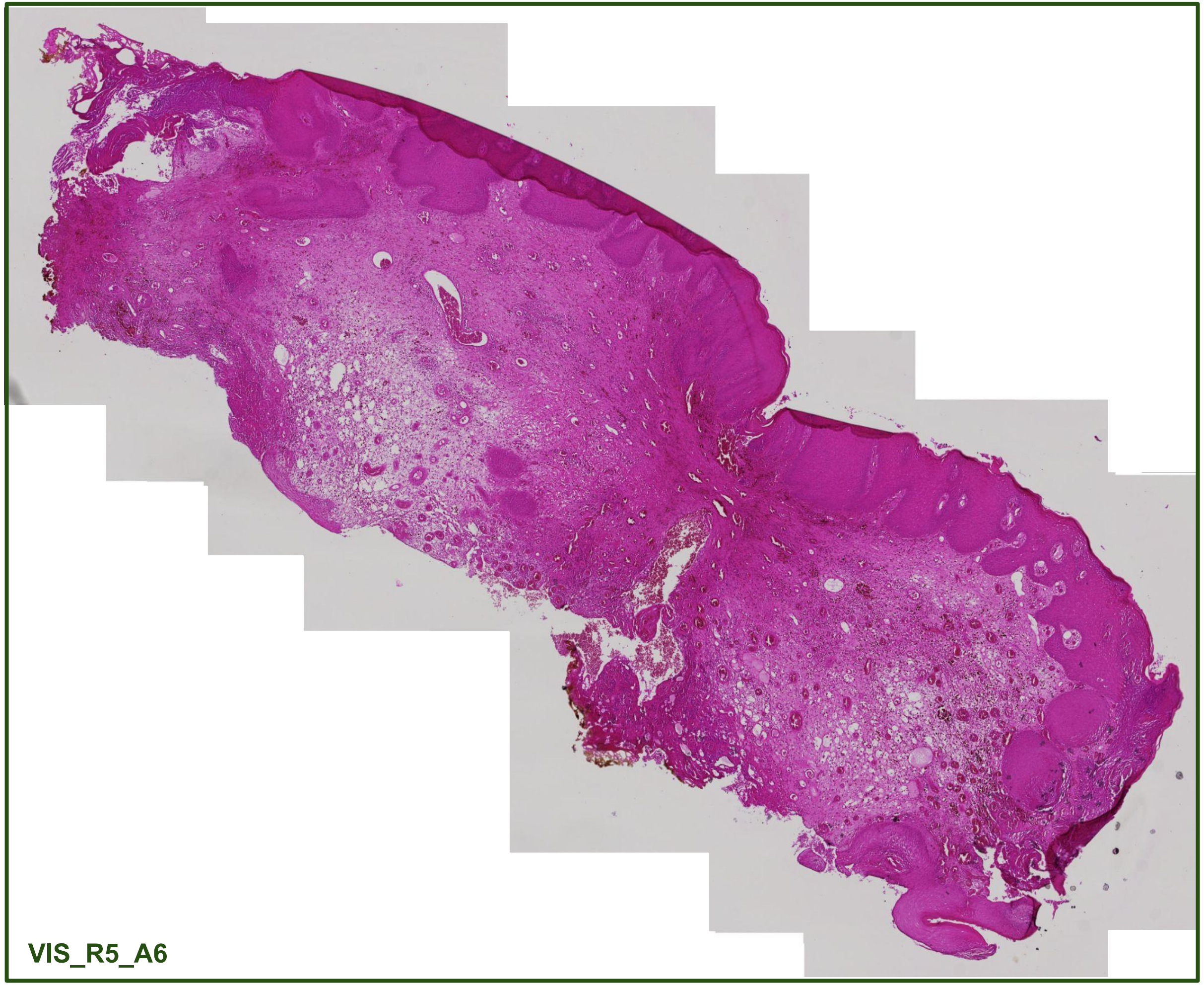

JR_45695_21

PERIANAL NON-CD
FISTULA

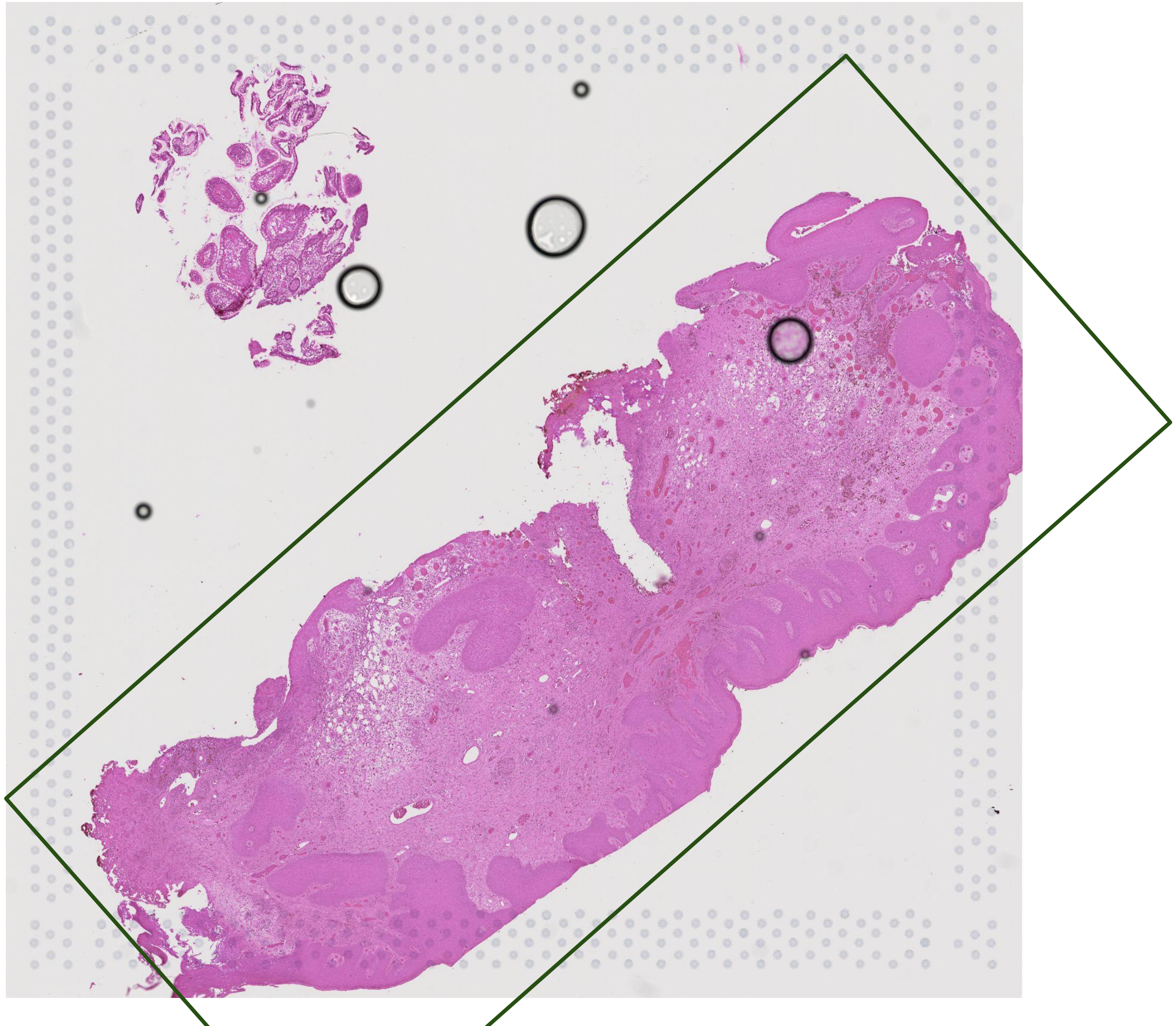

JR_15707_22

PERIANAL
NON-CD FISTULA

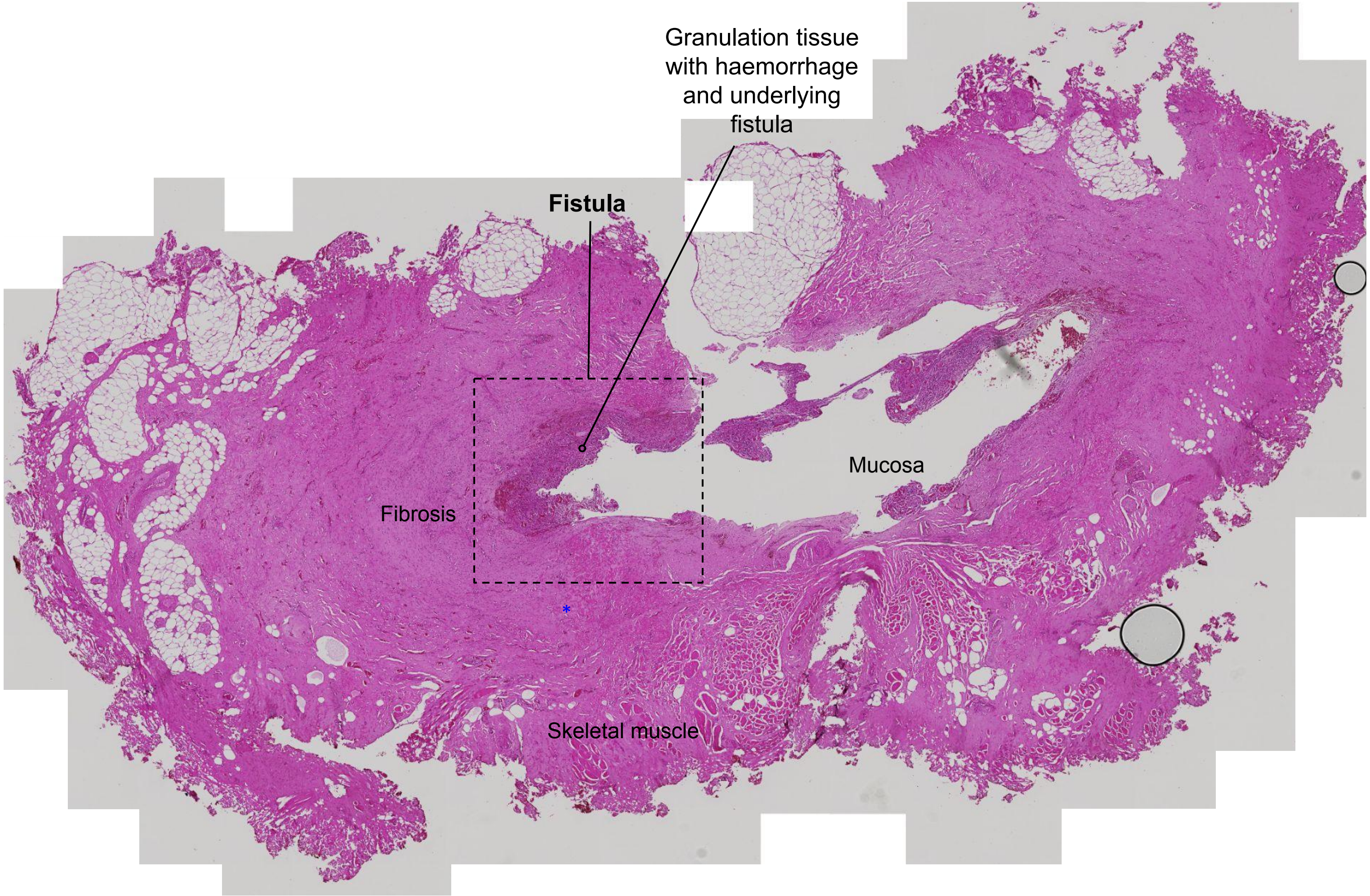

JR_15707_22

PERIANAL
NON-CD FISTULA

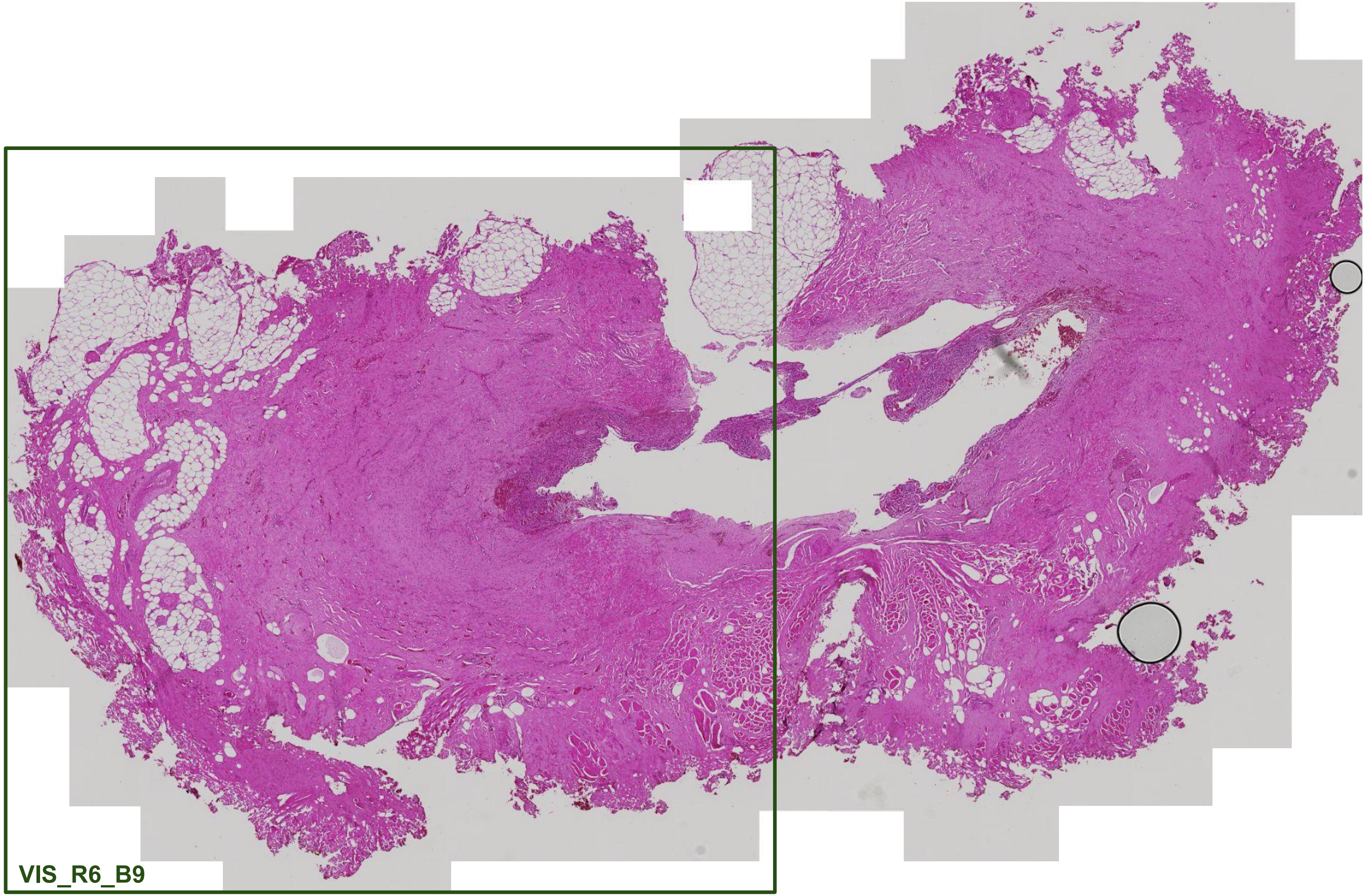

JR_15707_22

PERIANAL
NON-CD FISTULA

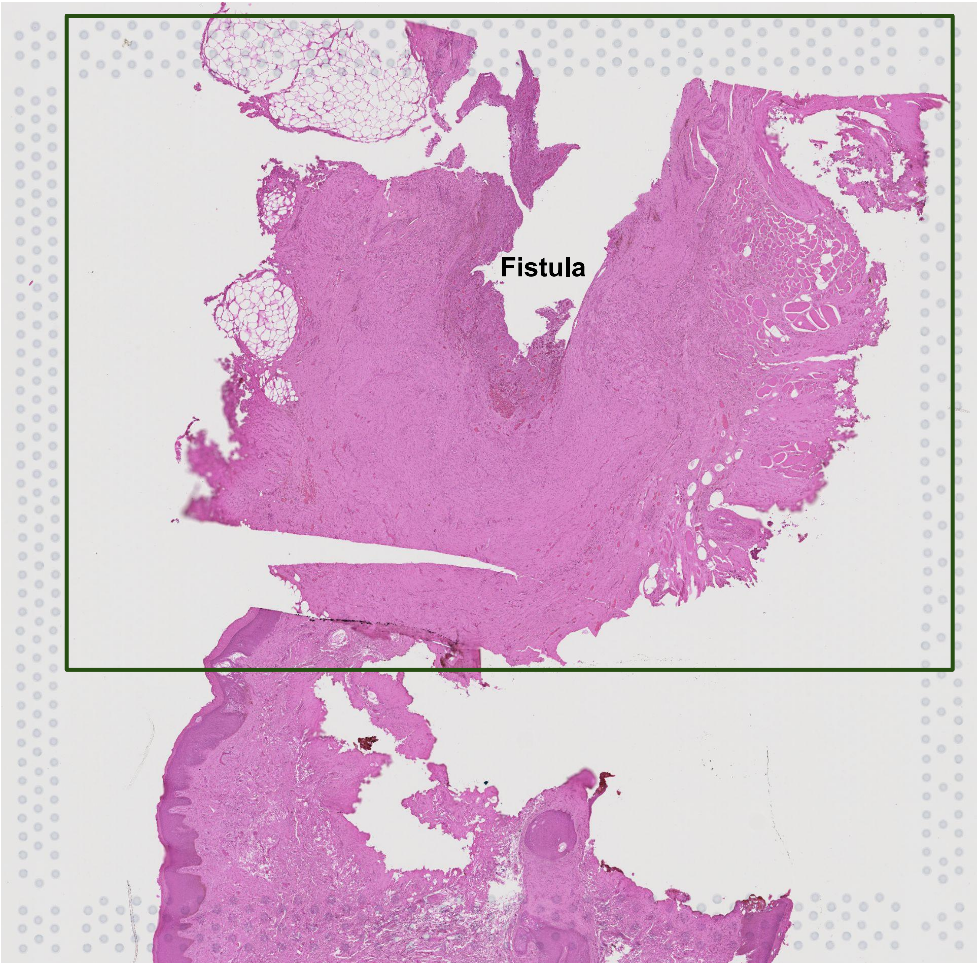

JR_20687_20

NON-CD FISTULA
DIVERTICULAR/PERIANAL

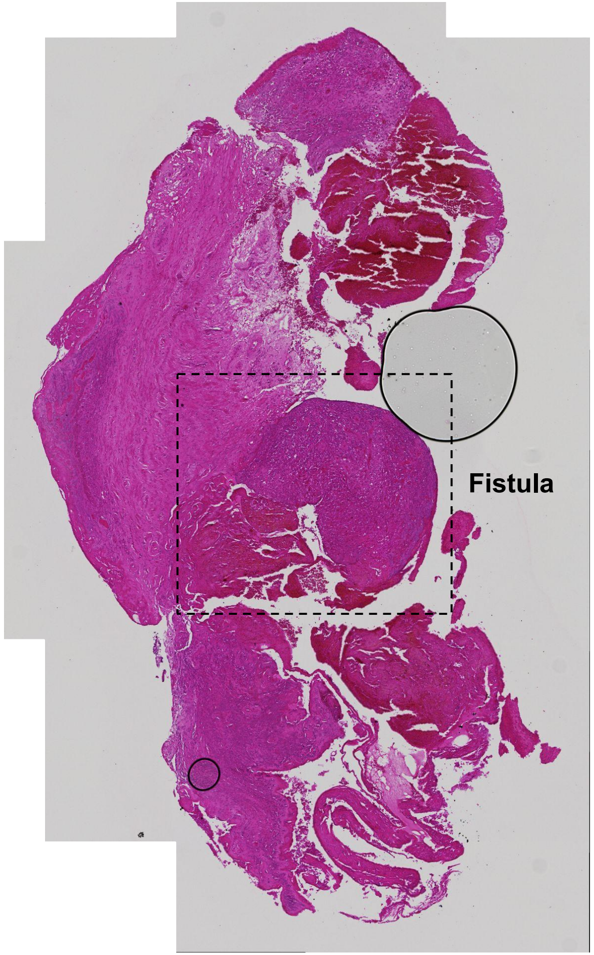

JR_20687_20

NON-CD FISTULA
DIVERTICULAR/PERIANAL

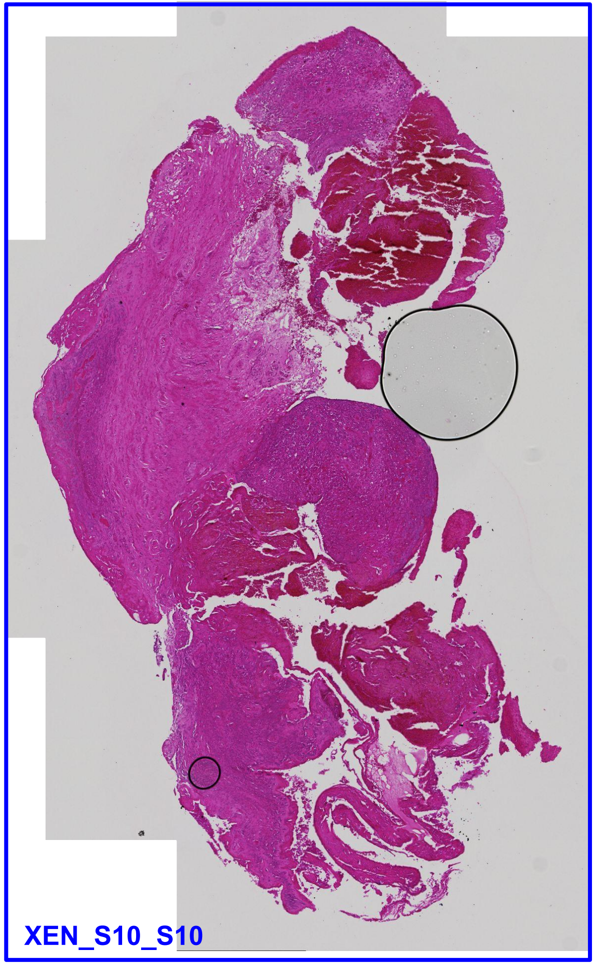

JR_29328_15

INFLAMMATORY CD

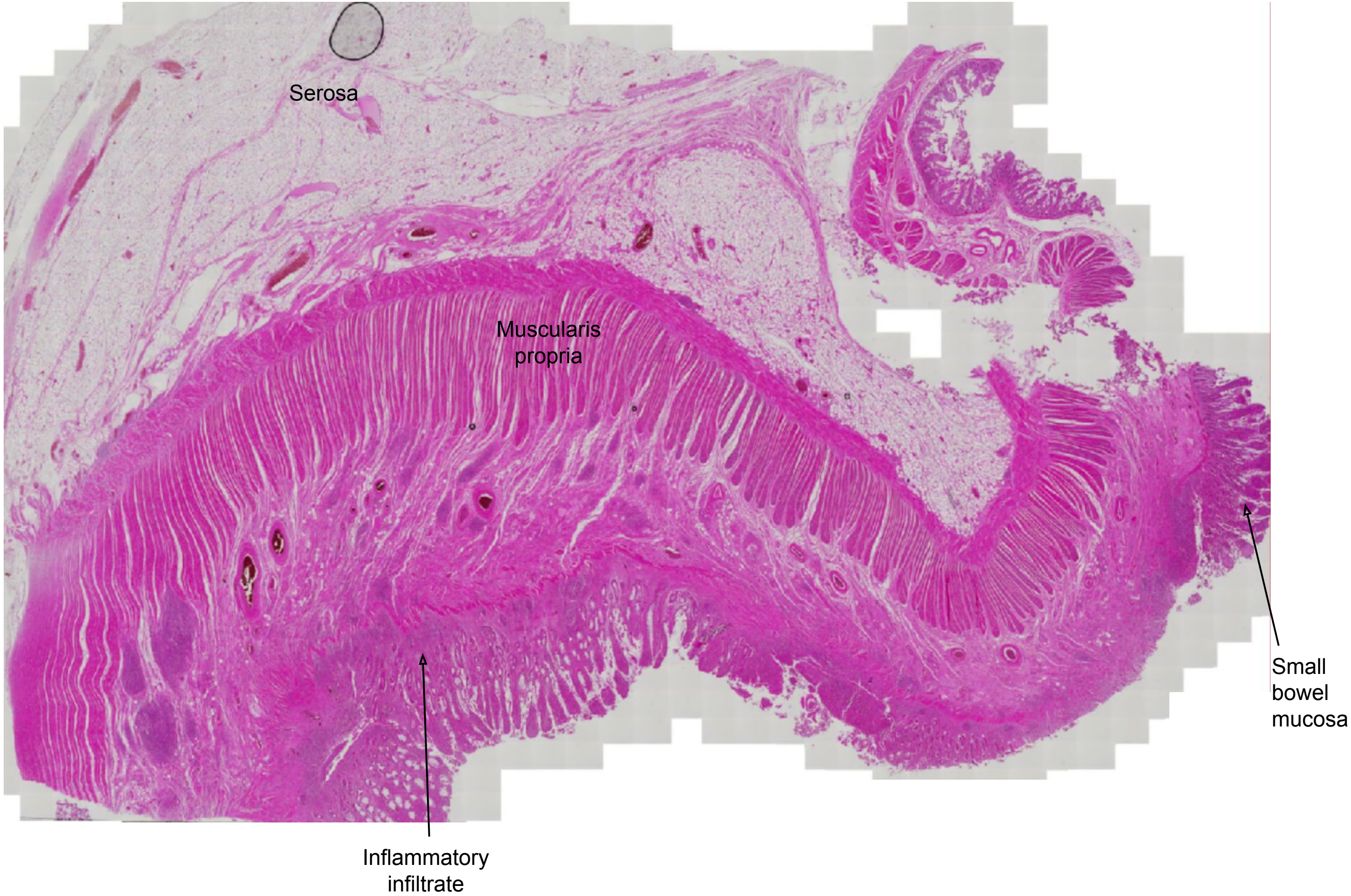

JR_29328_15

INFLAMMATORY CD

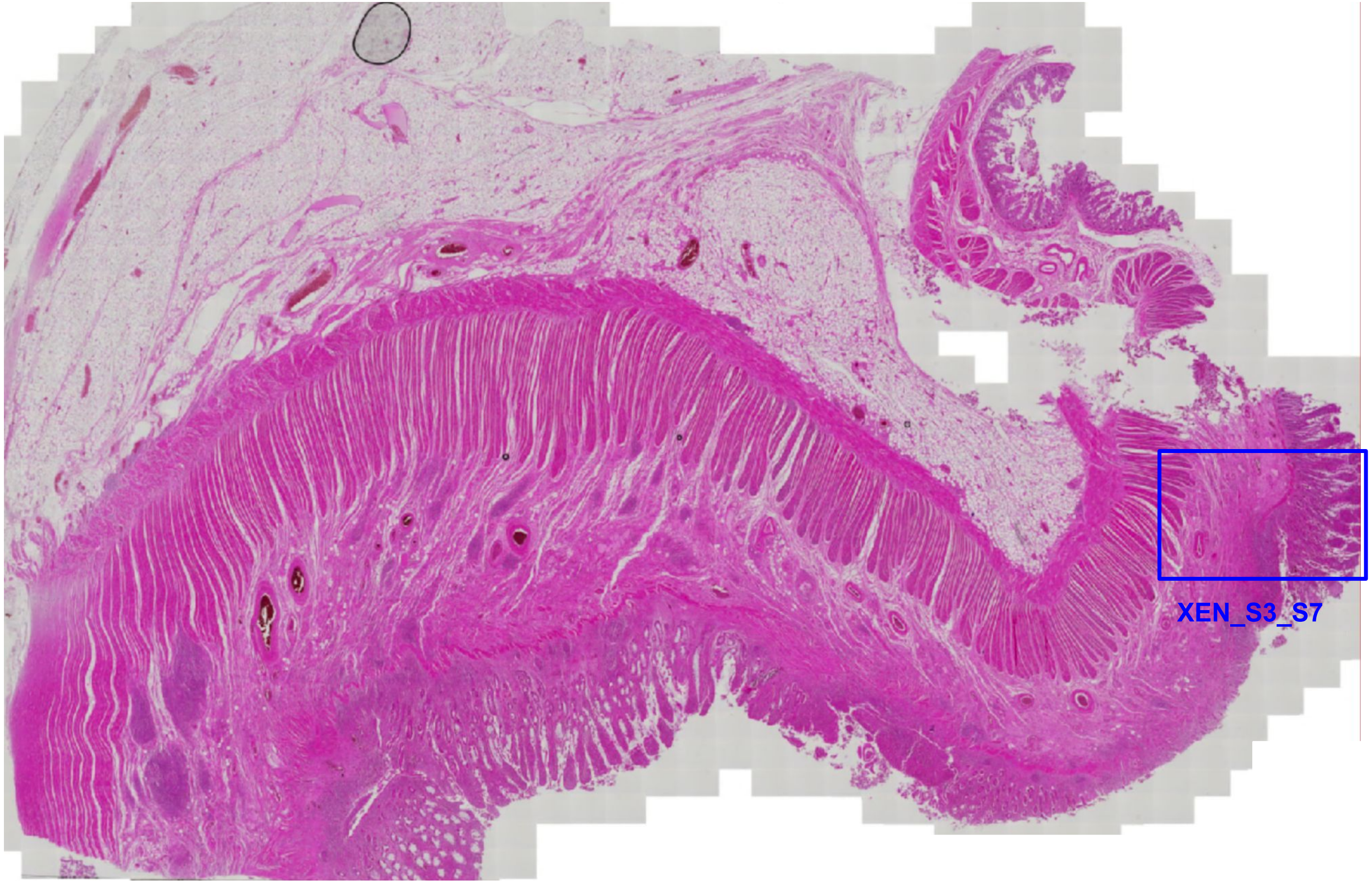

JR_32929_22

INFLAMMATORY CD

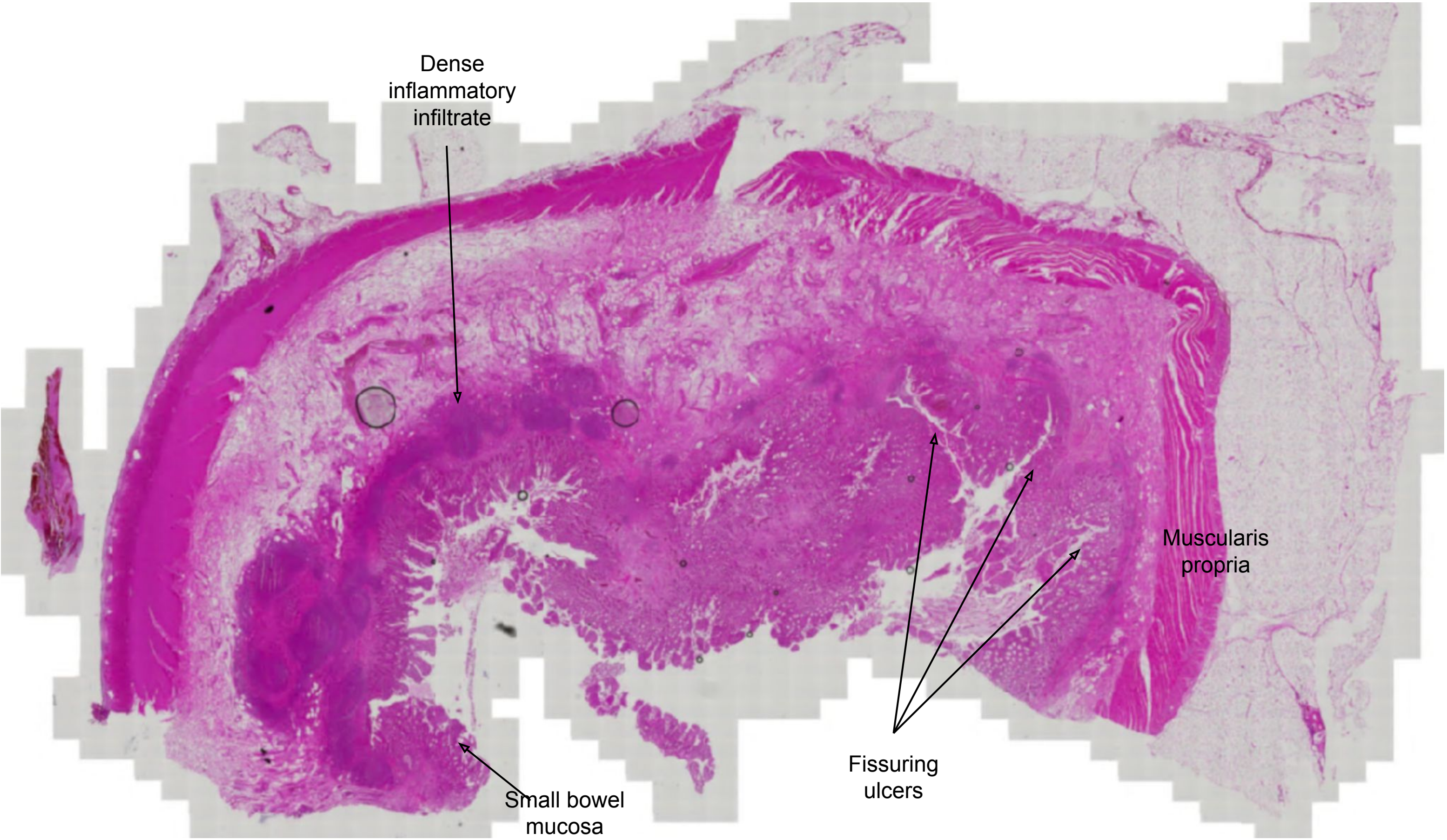

JR_32929_22

INFLAMMATORY CD

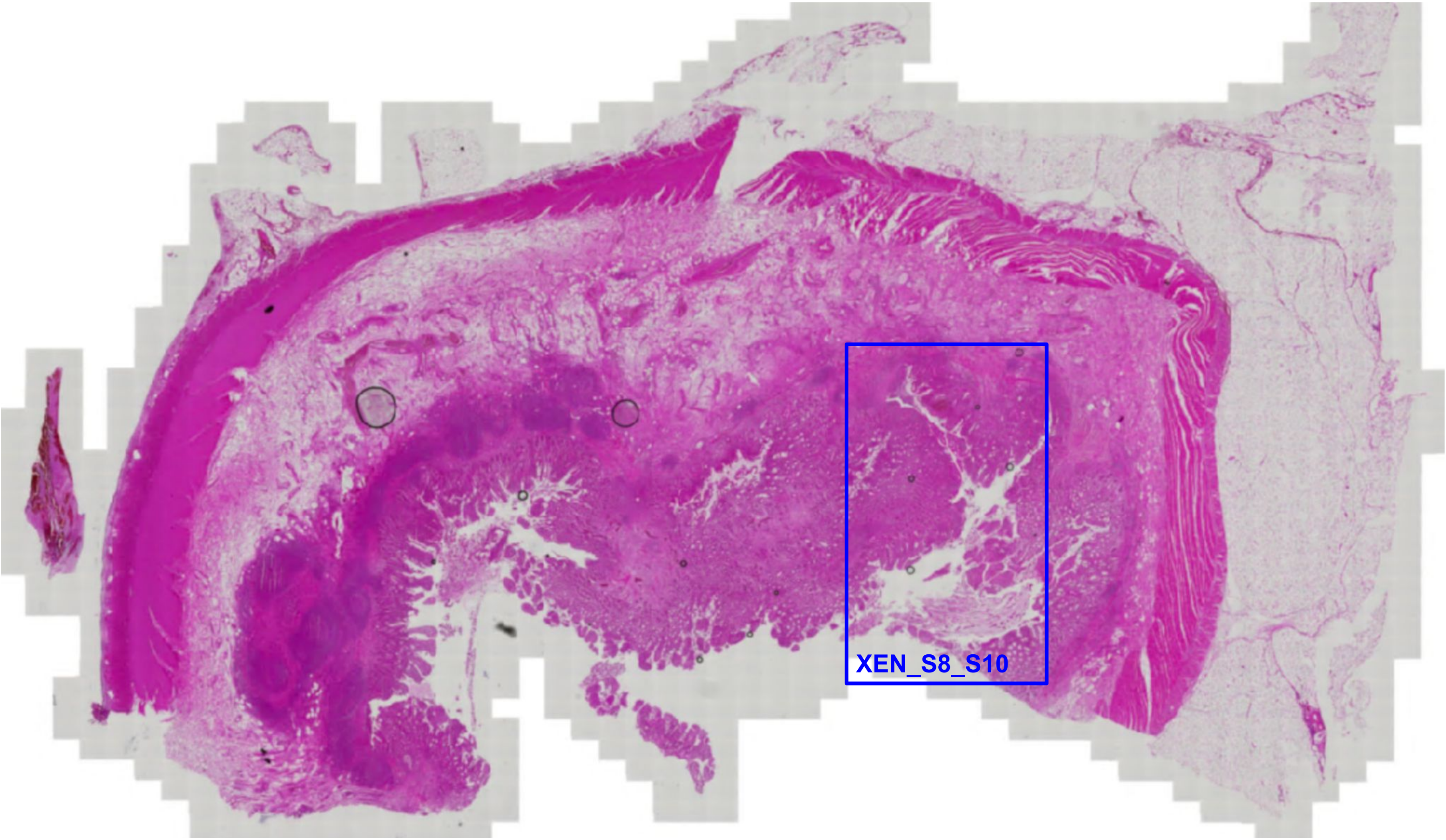

JR_6583_21

INFLAMMATORY CD

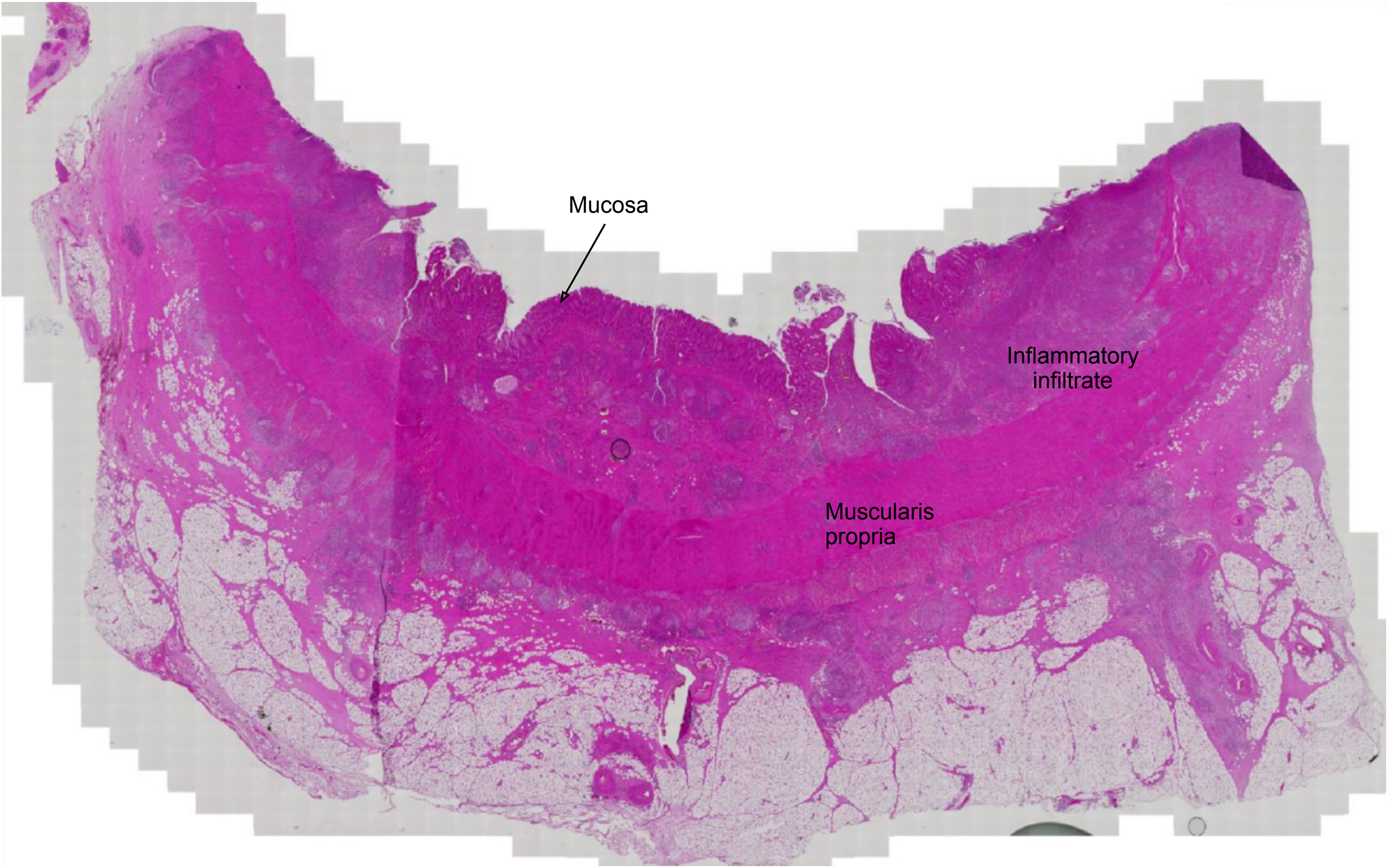

JR_6583_21

INFLAMMATORY CD

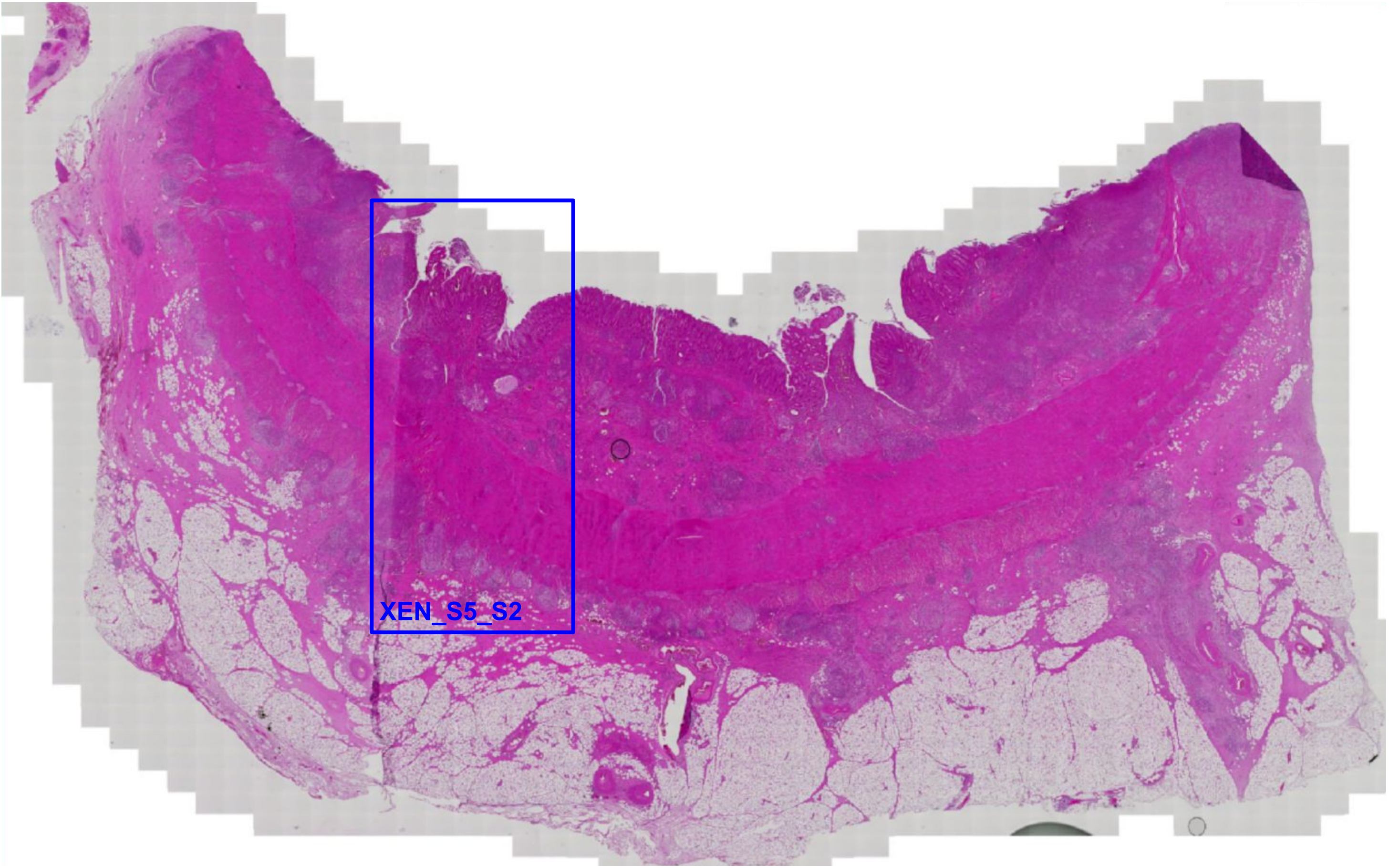

JR_28726_24

CD ULCER

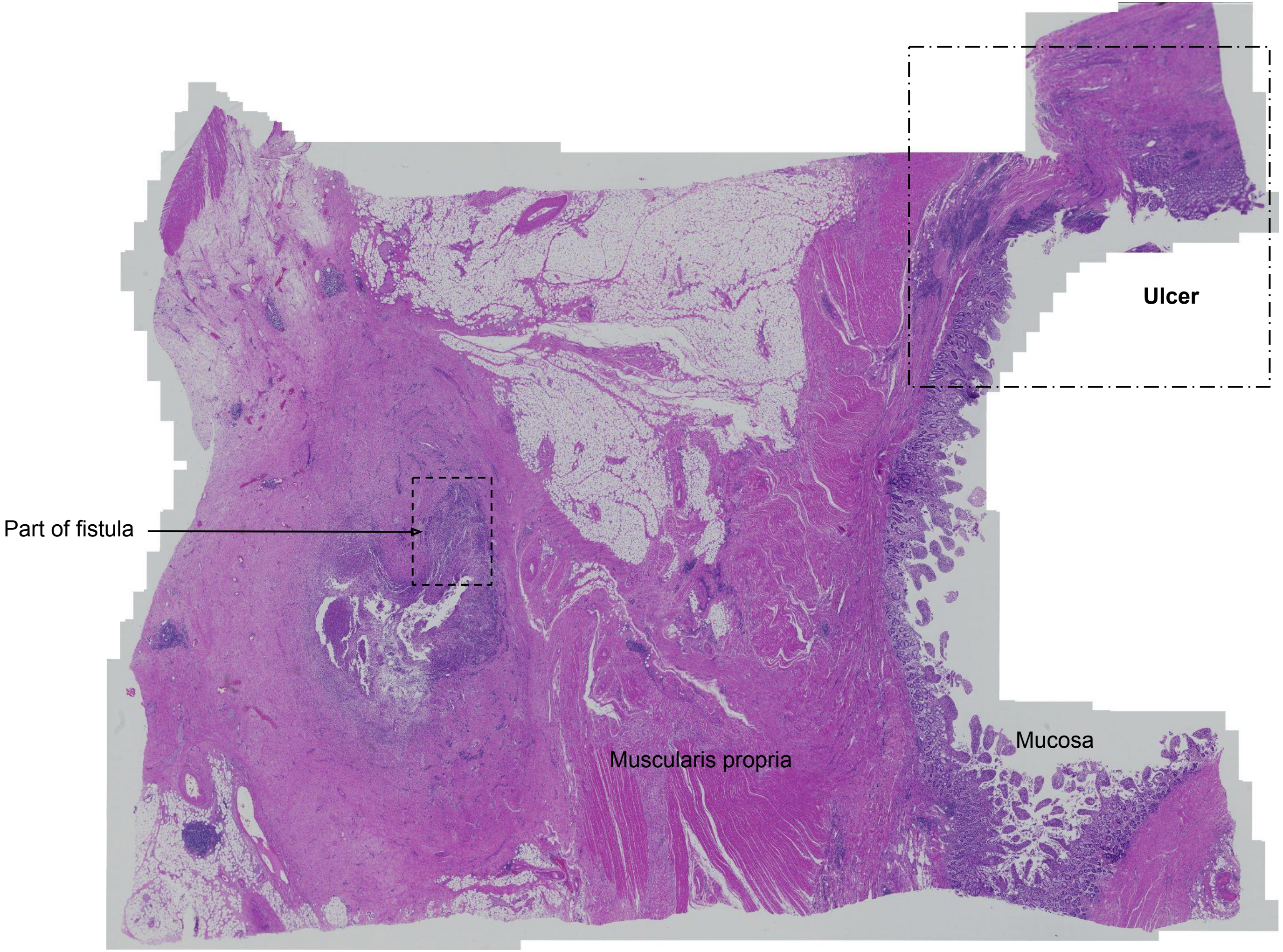

JR_28726_24

CD ULCER

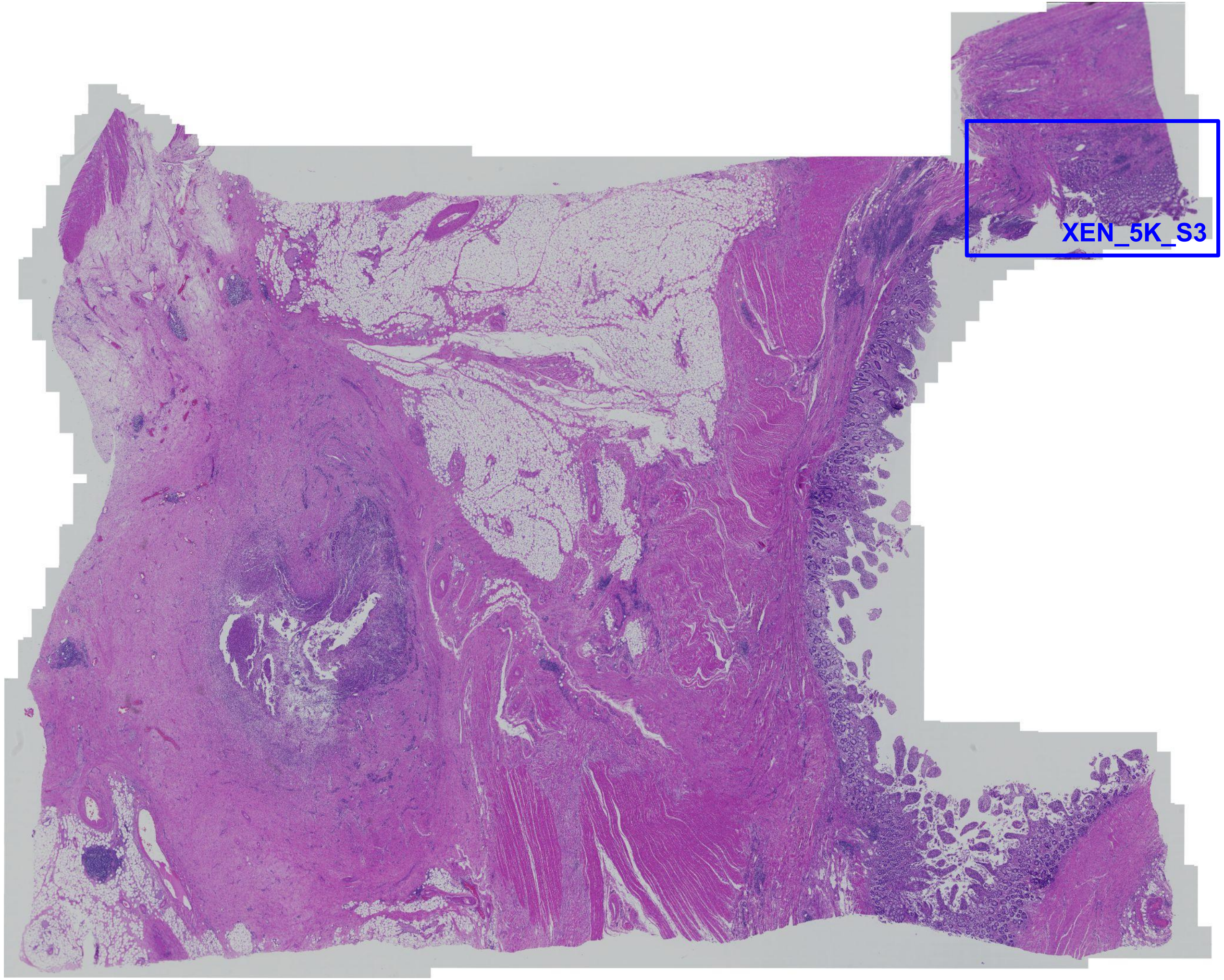

JR_36213_24

CD ULCER (NON-FISTULATING)

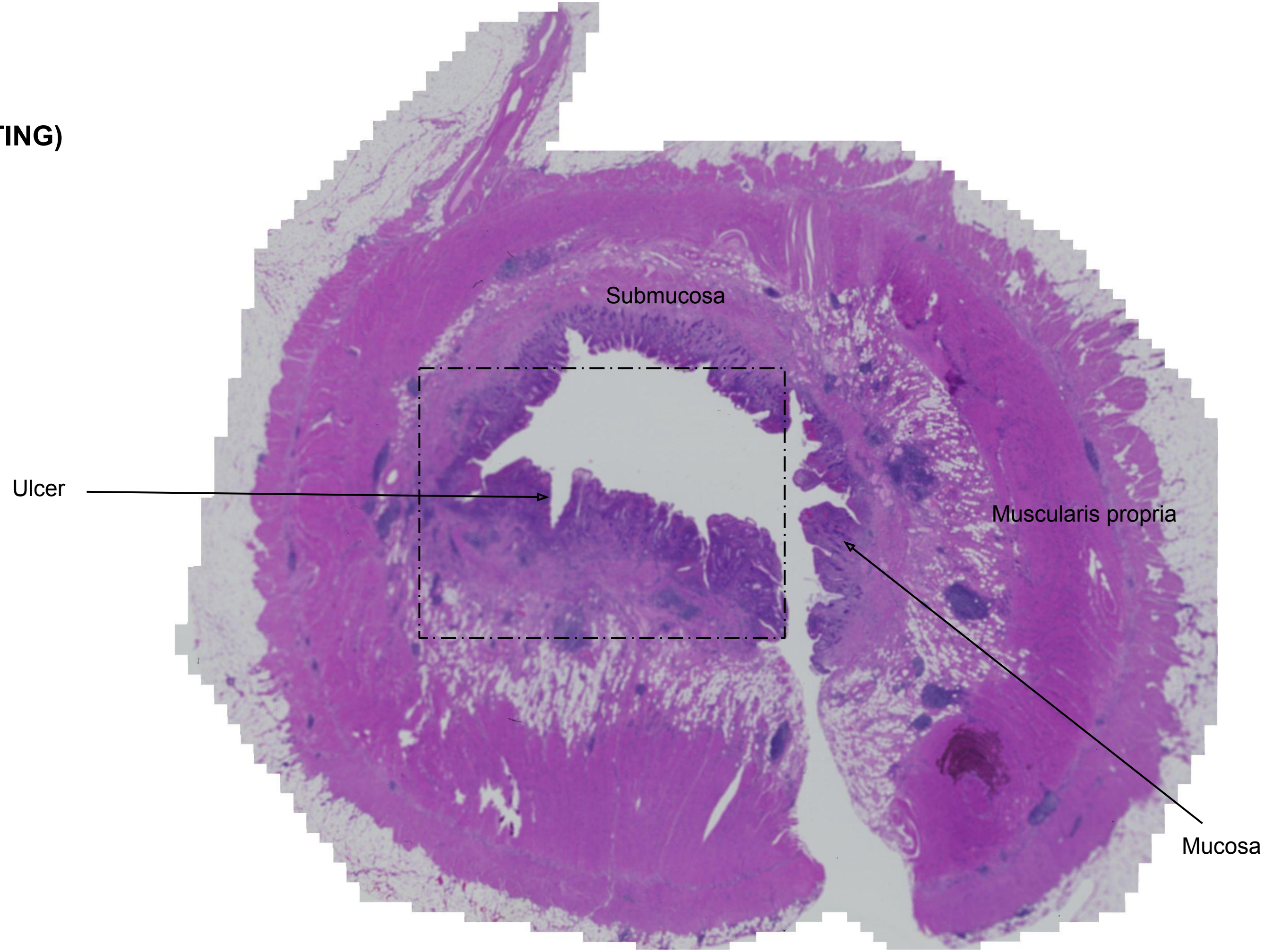

JR_36213_24

CD ULCER
(NON-FISTULATING)

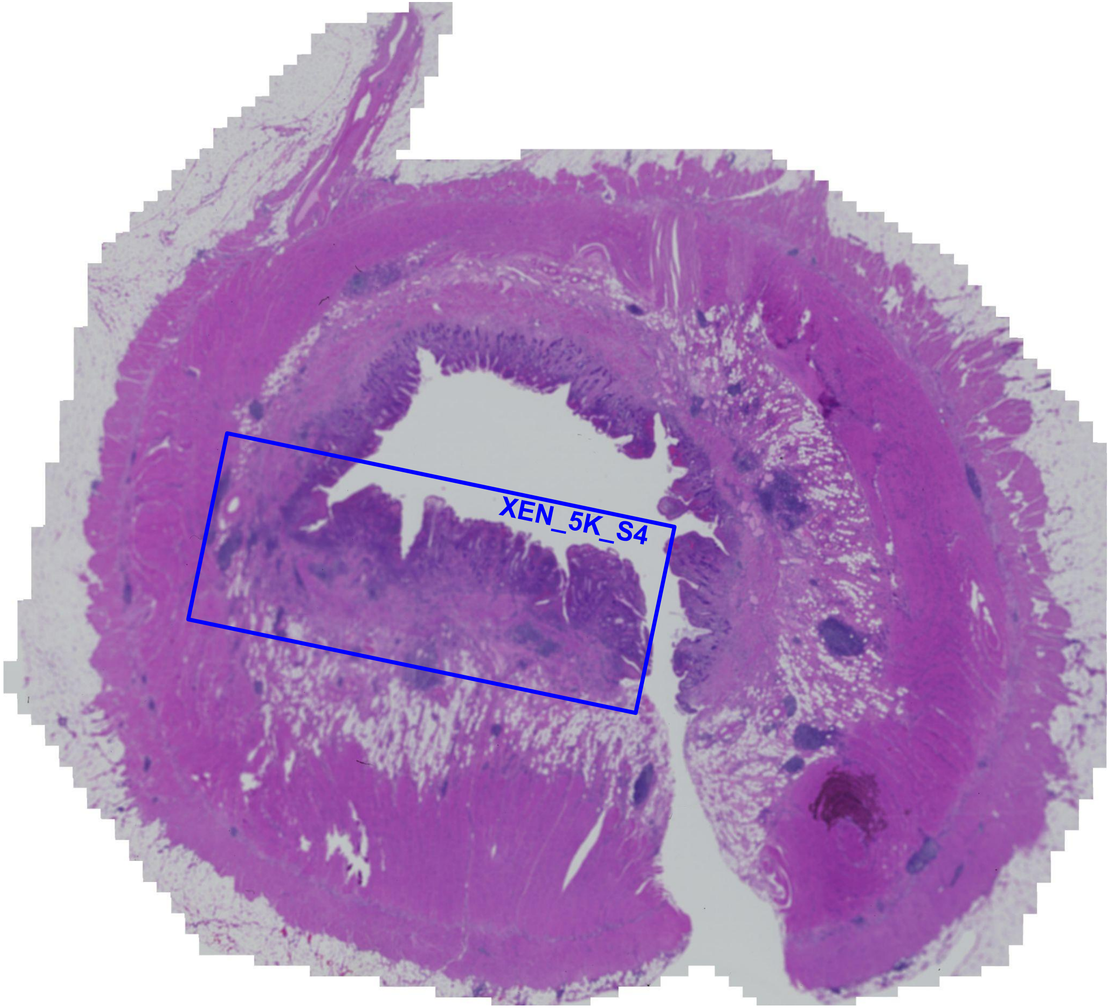

JR_39292_24

CD ULCER
(NON-FISTULATING)

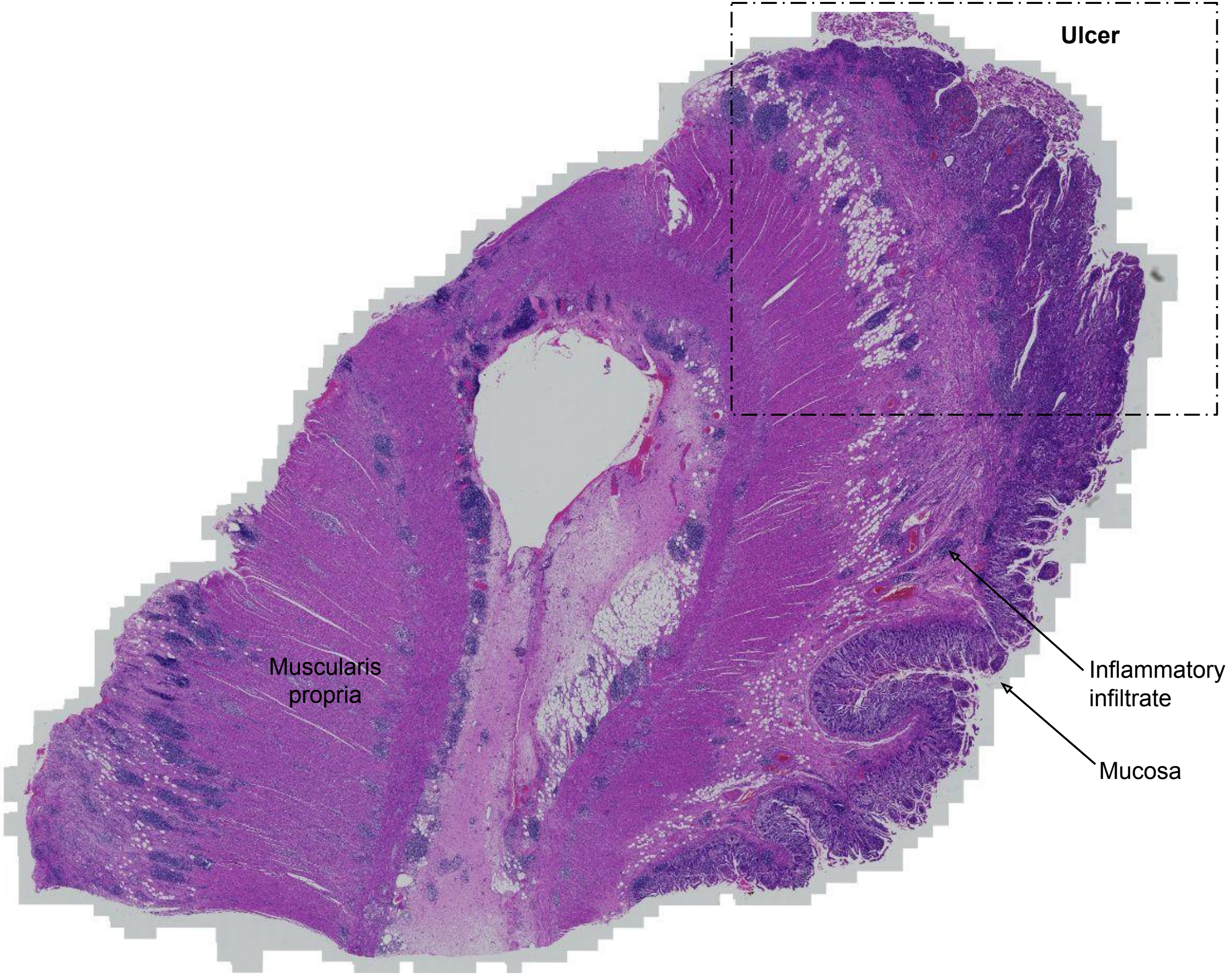

JR_39292_24

CD ULCER
(NON-FISTULATING)

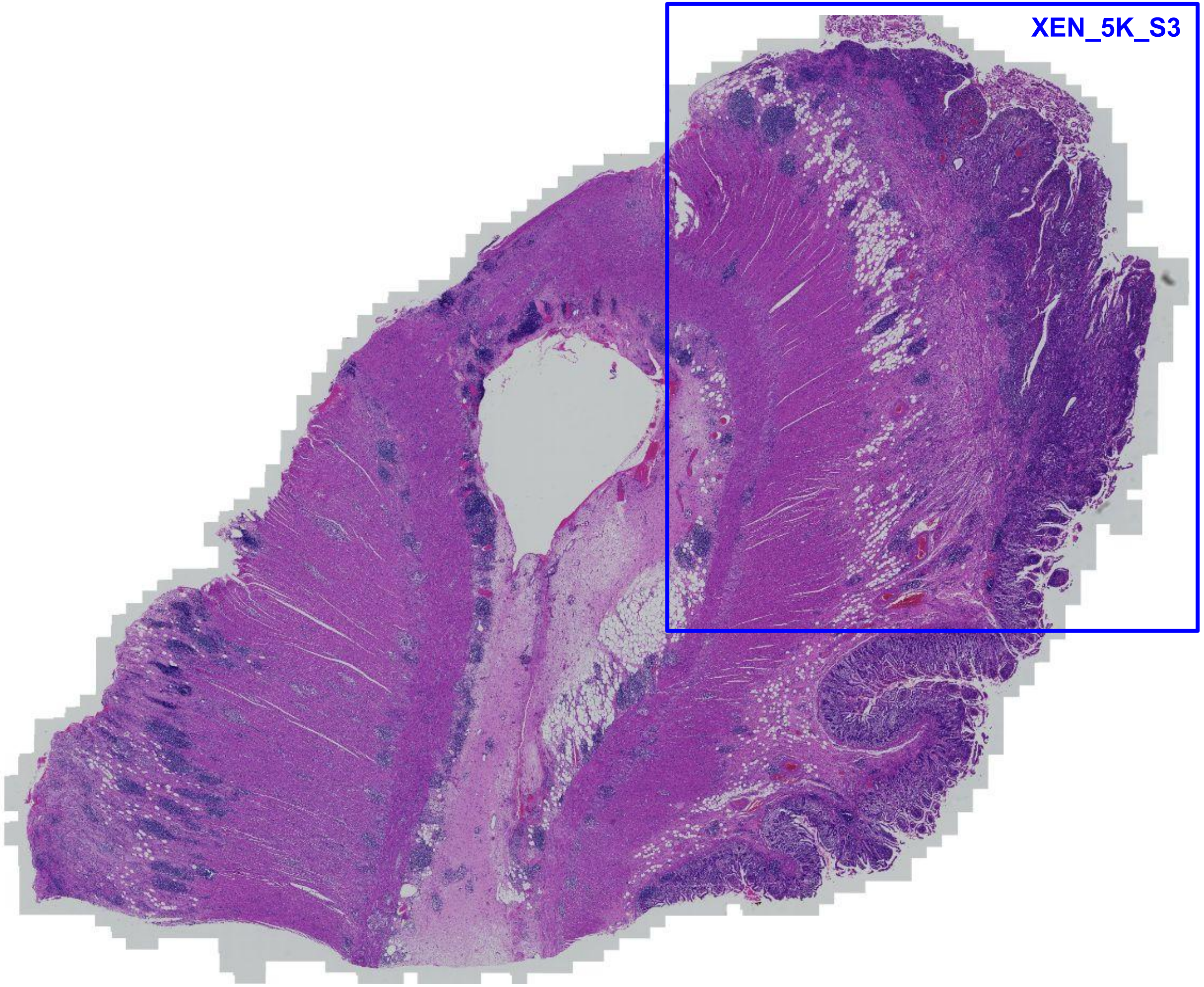

JR_41744_24

CD ULCER (NON-FISTULATING)

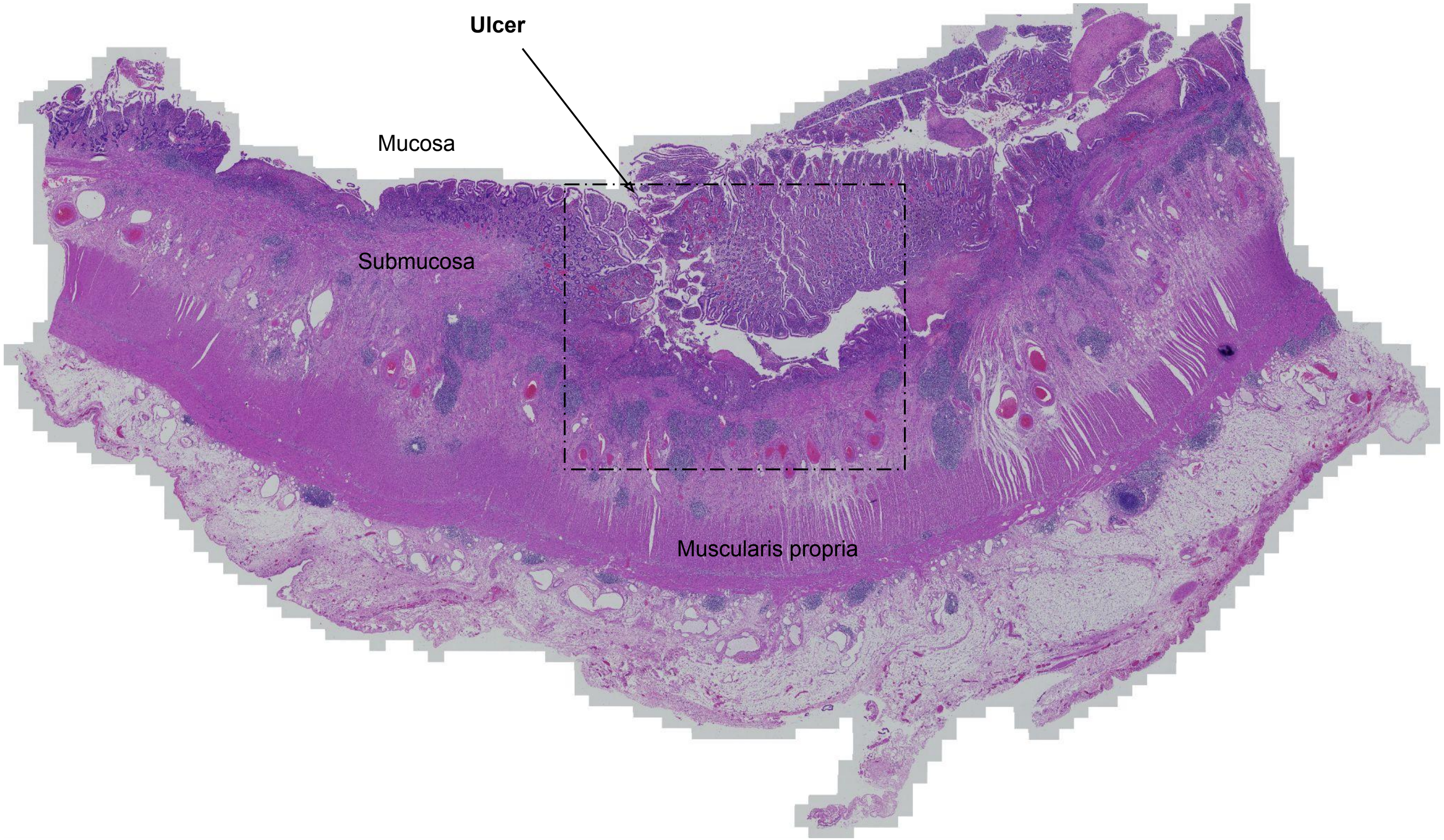

JR_41744_24

CD ULCER
(NON-FISTULATING)

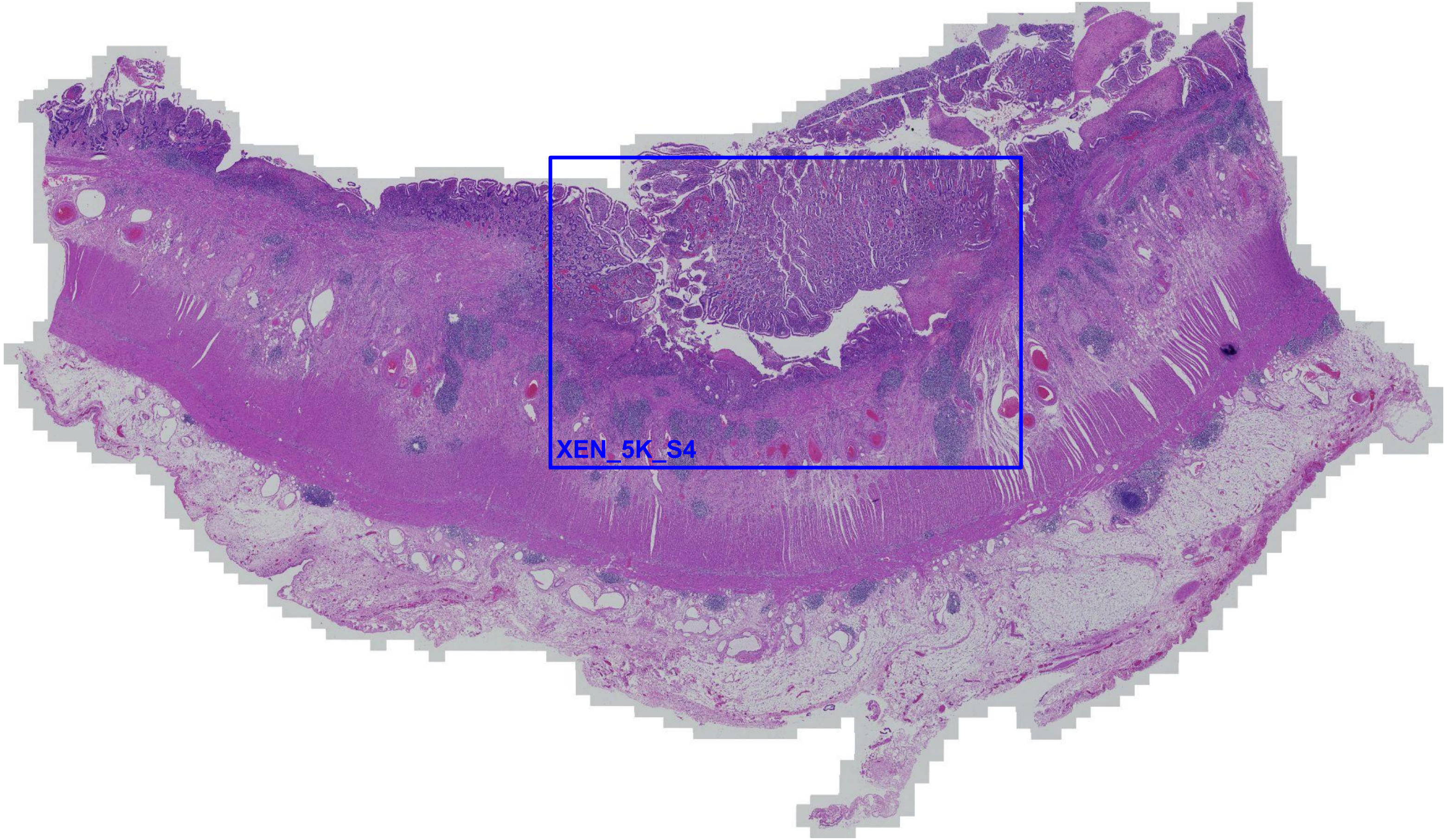

JR_45737_24

CD ULCER (NON-FISTULATING)

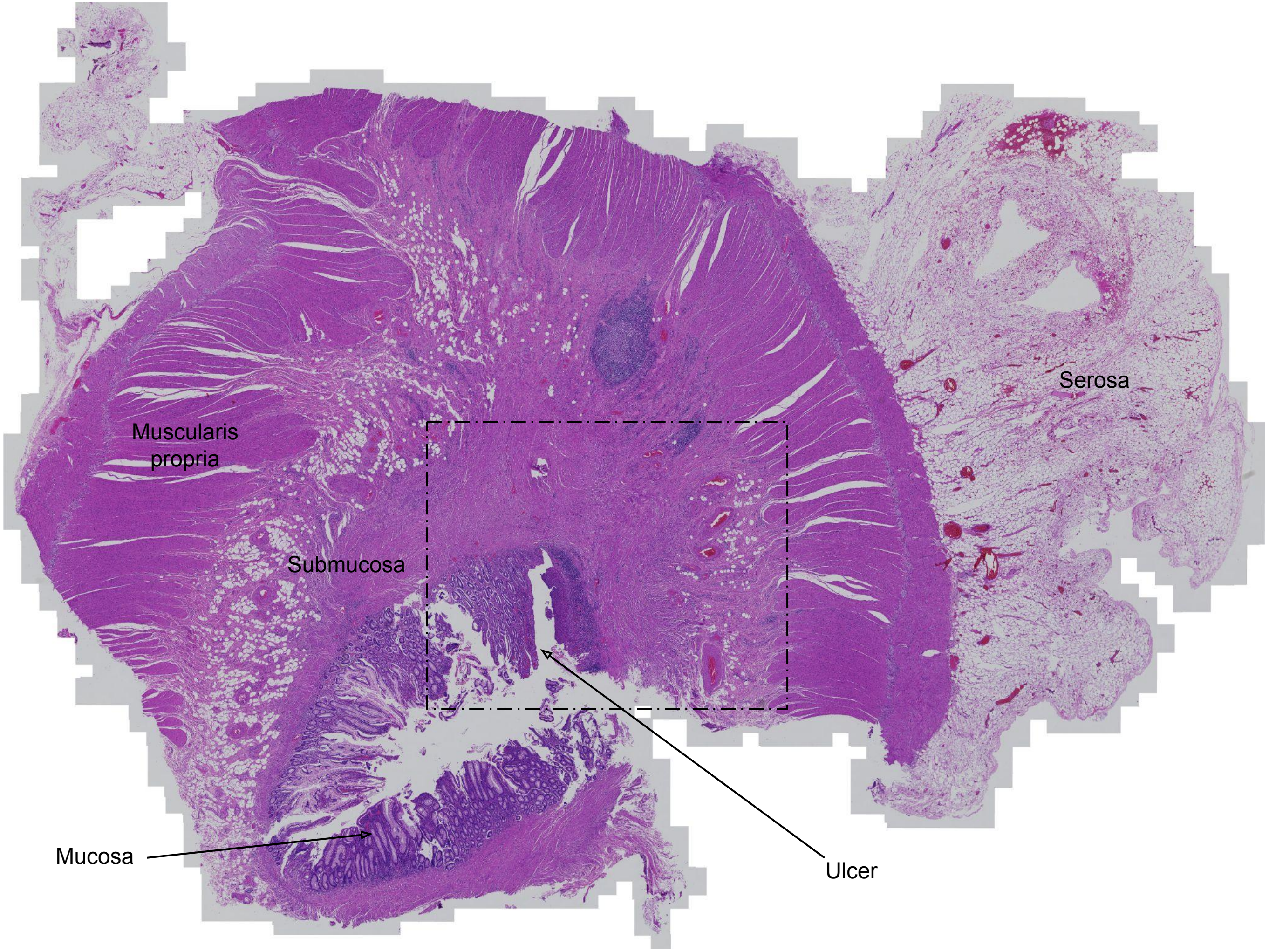

JR_45737_24

CD ULCER
(NON-FISTULATING)

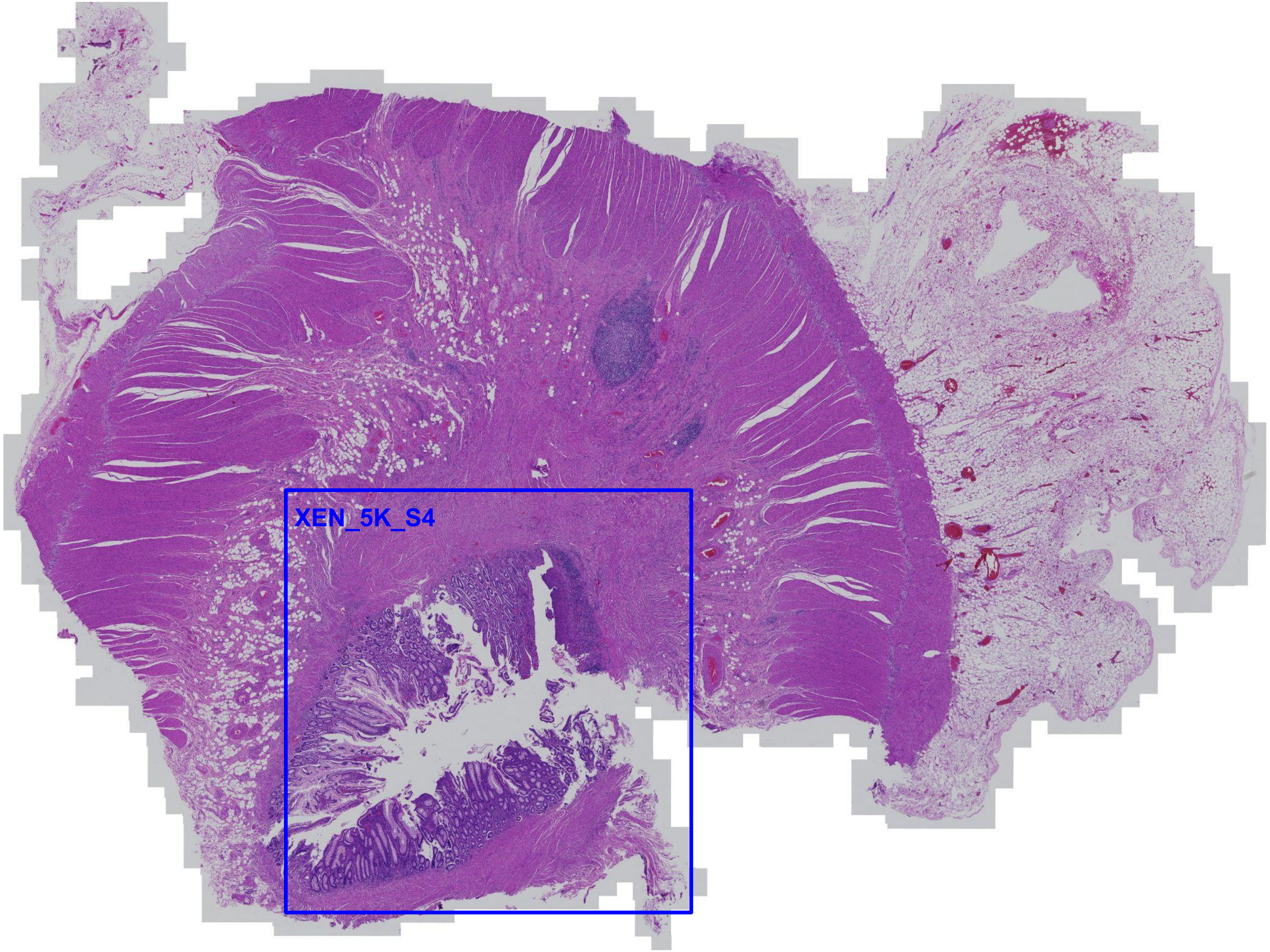

TIP 815

INFLAMMATORY CD ILEUM

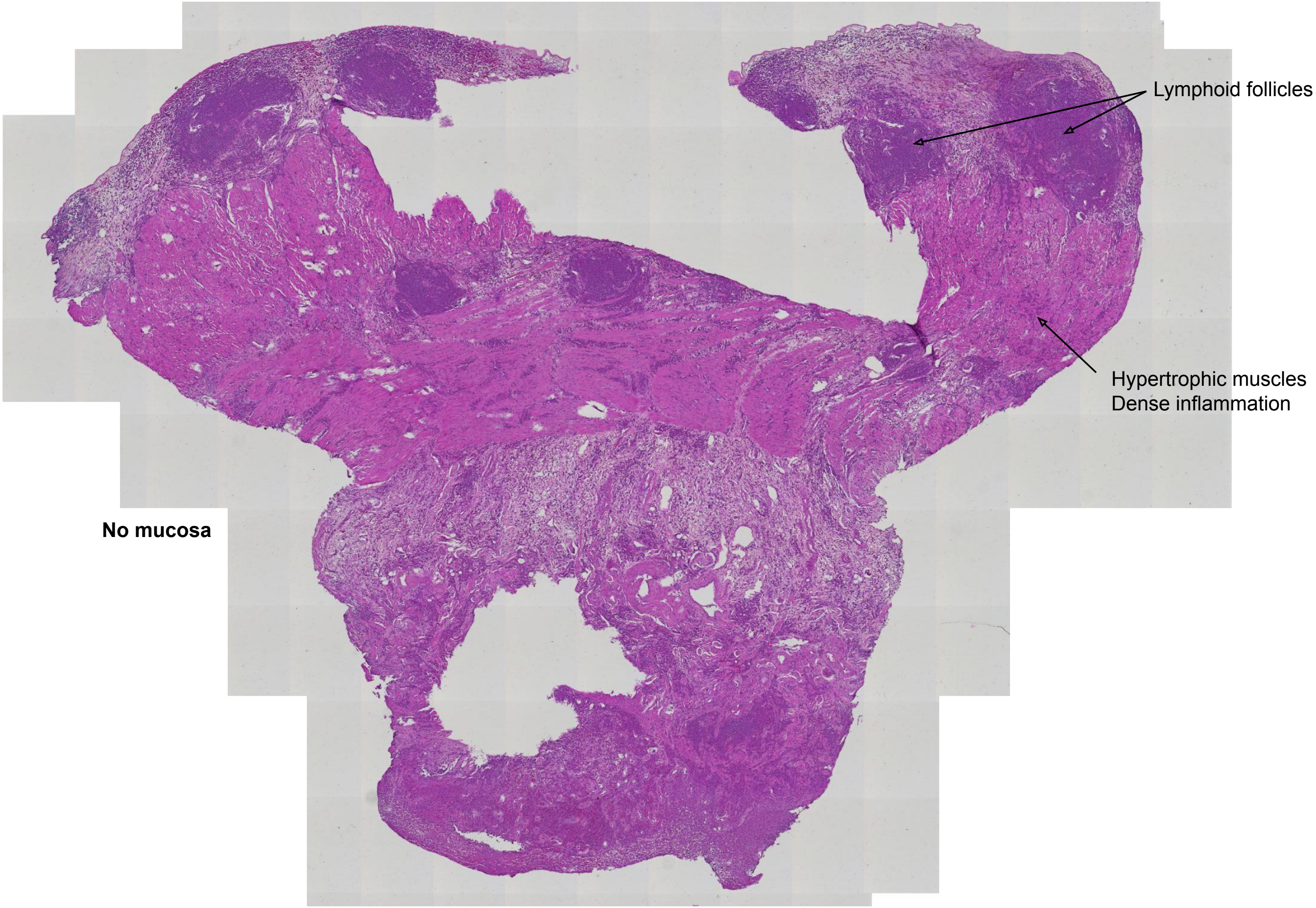

TIP 815

INFLAMMATORY CD ILEUM

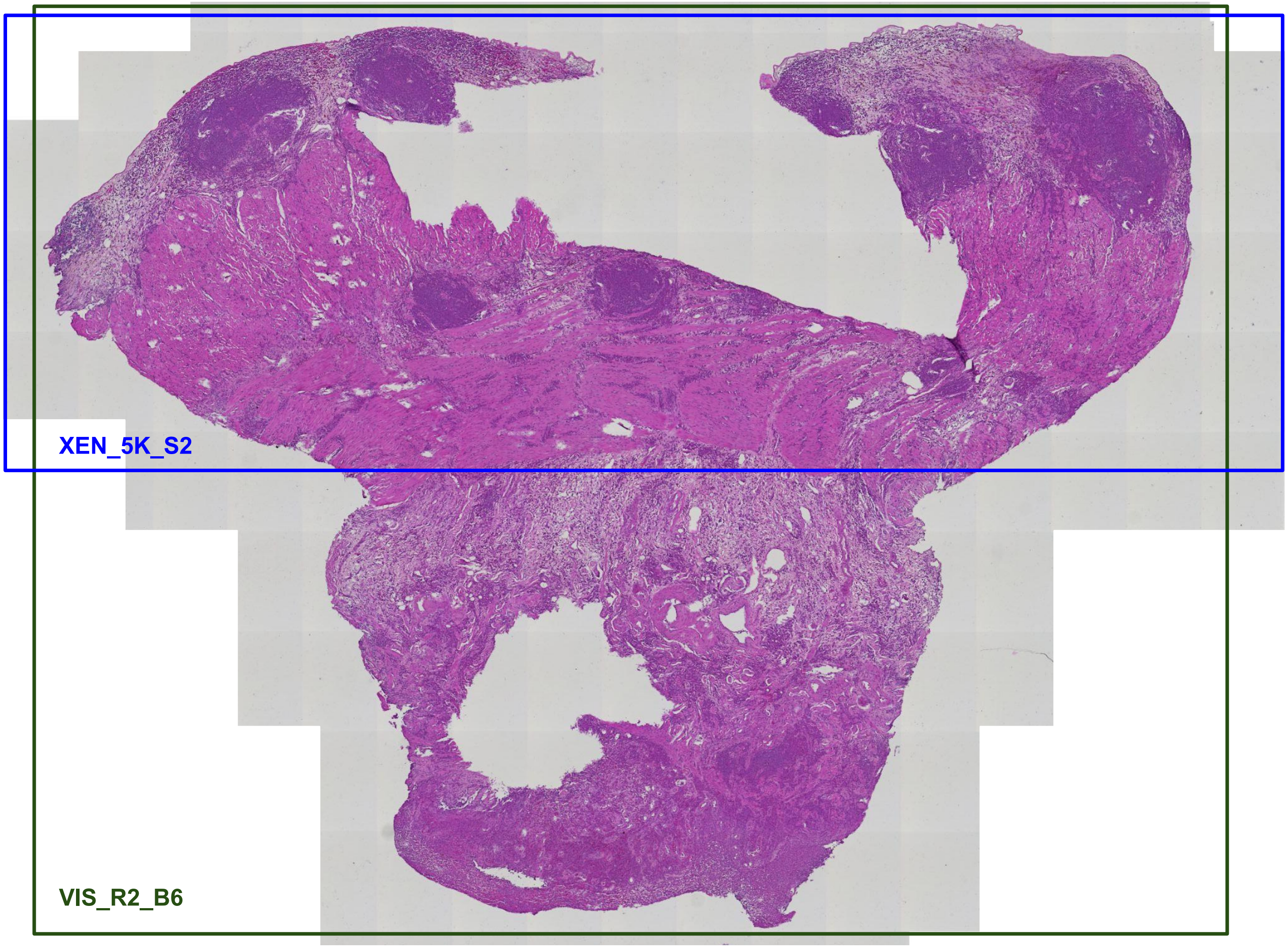

TIP 815

INFLAMMATORY CD
ILEUM

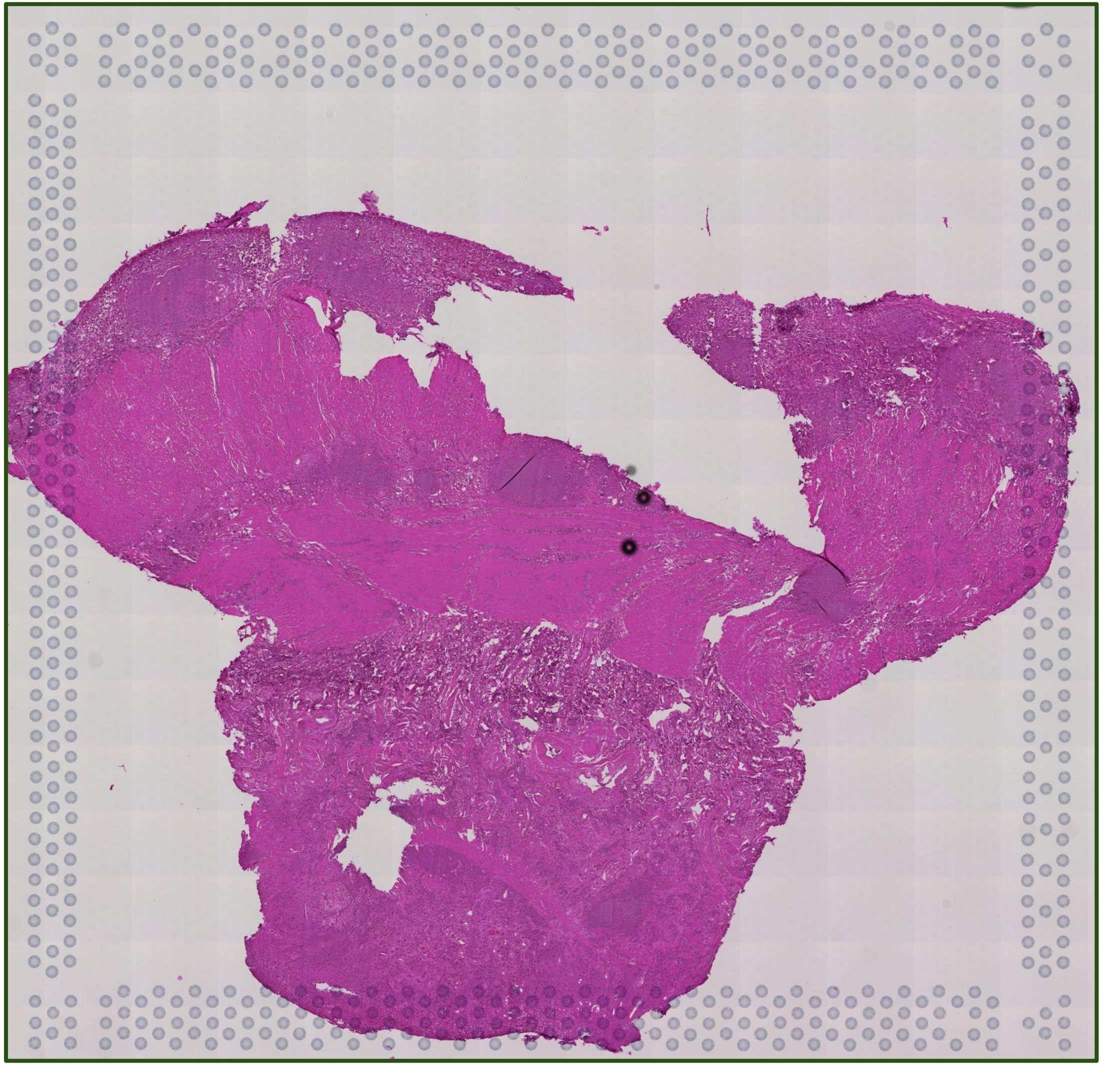

GI 6966

INFLAMMATORY CD
ILEUM

Inflammatory cells in
lamina propria

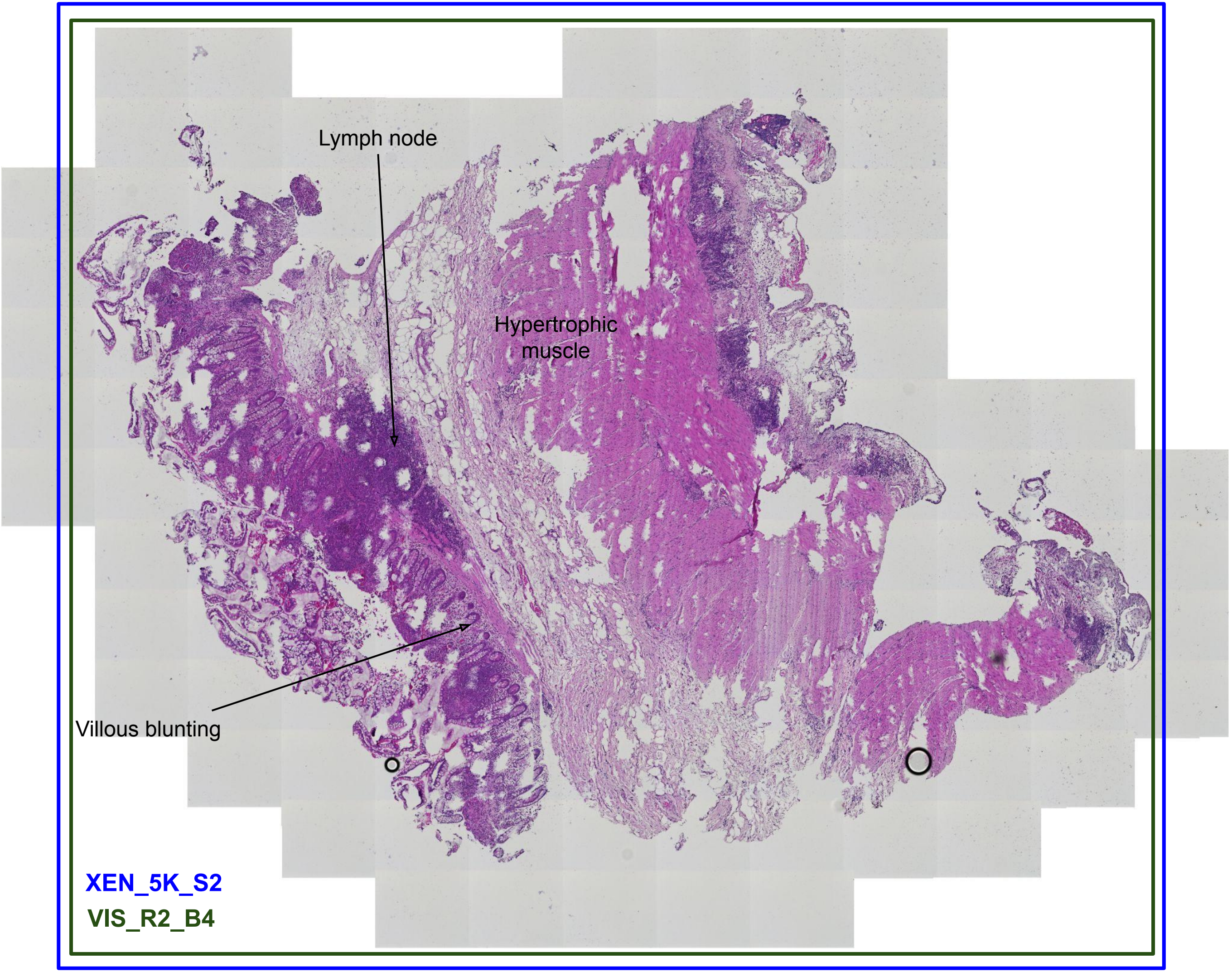

GI 6966

INFLAMMATORY CD
ILEUM

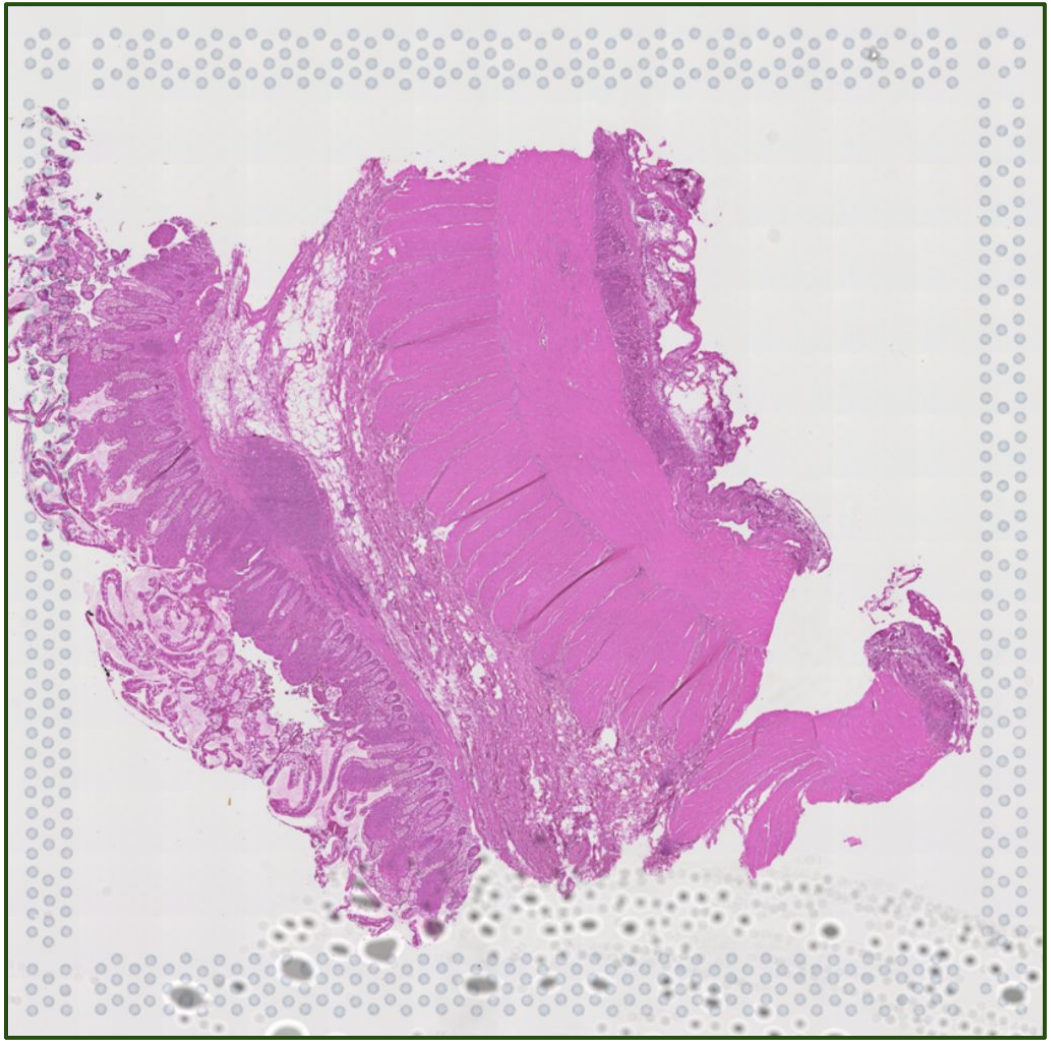

GI 9662

INFLAMMATORY CD

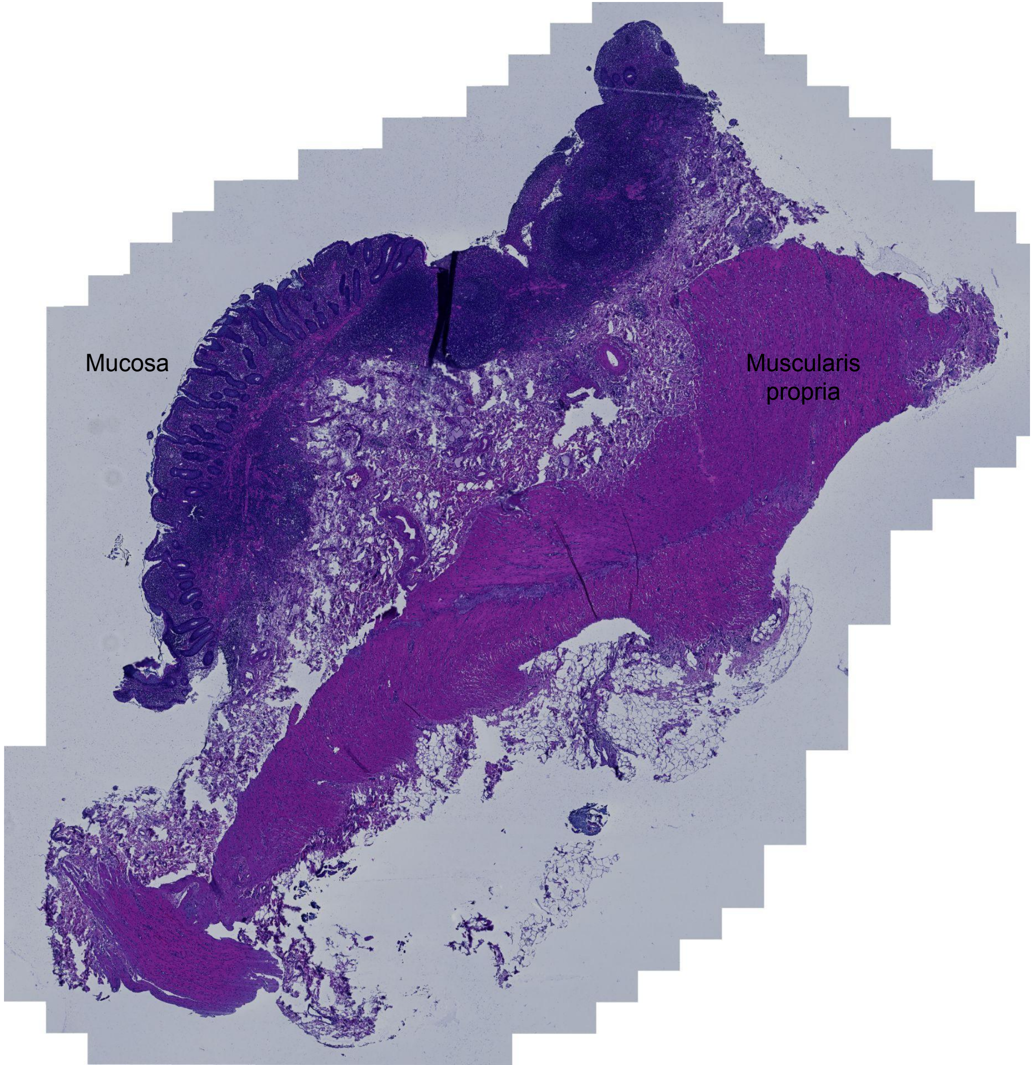

GI 9662

INFLAMMATORY CD

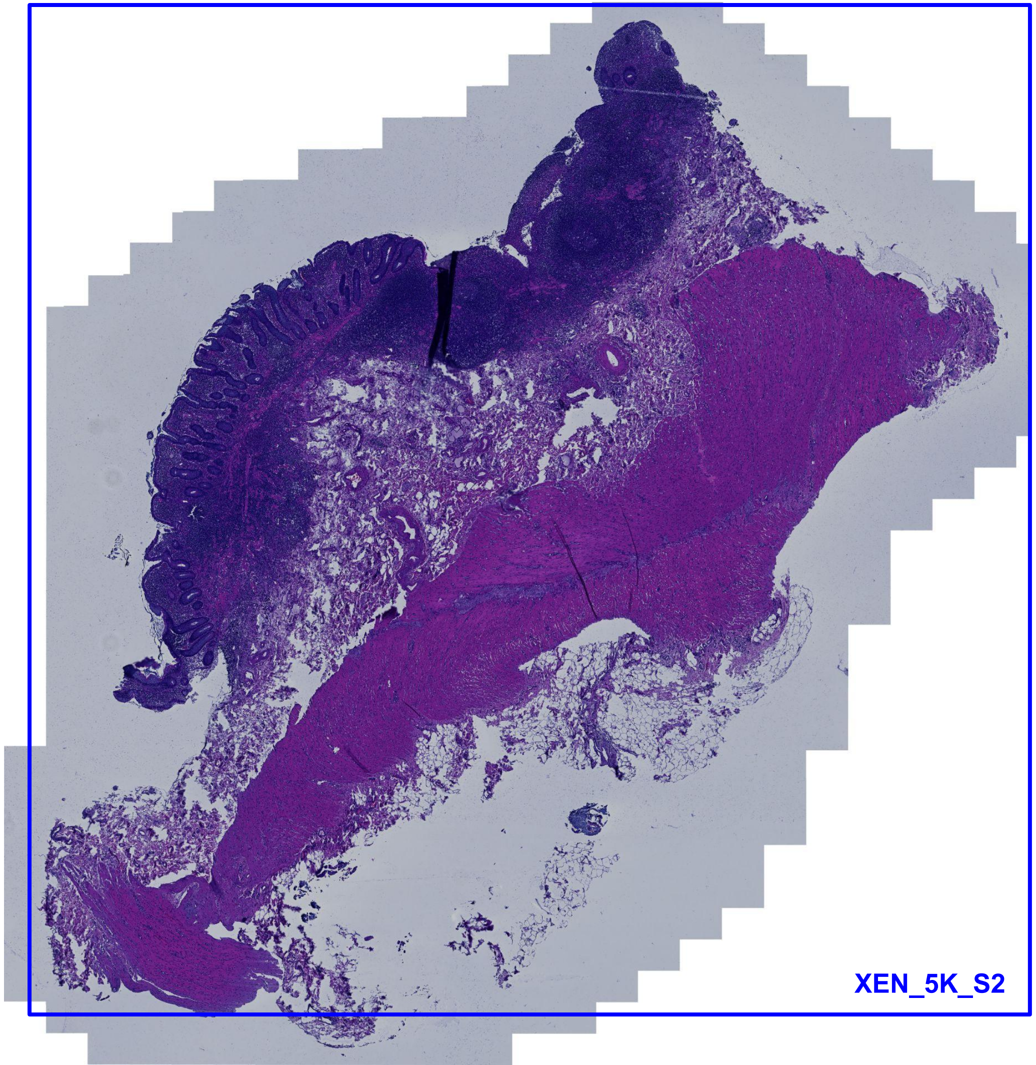

TIP 864

INFLAMMATORY CD ILEUM

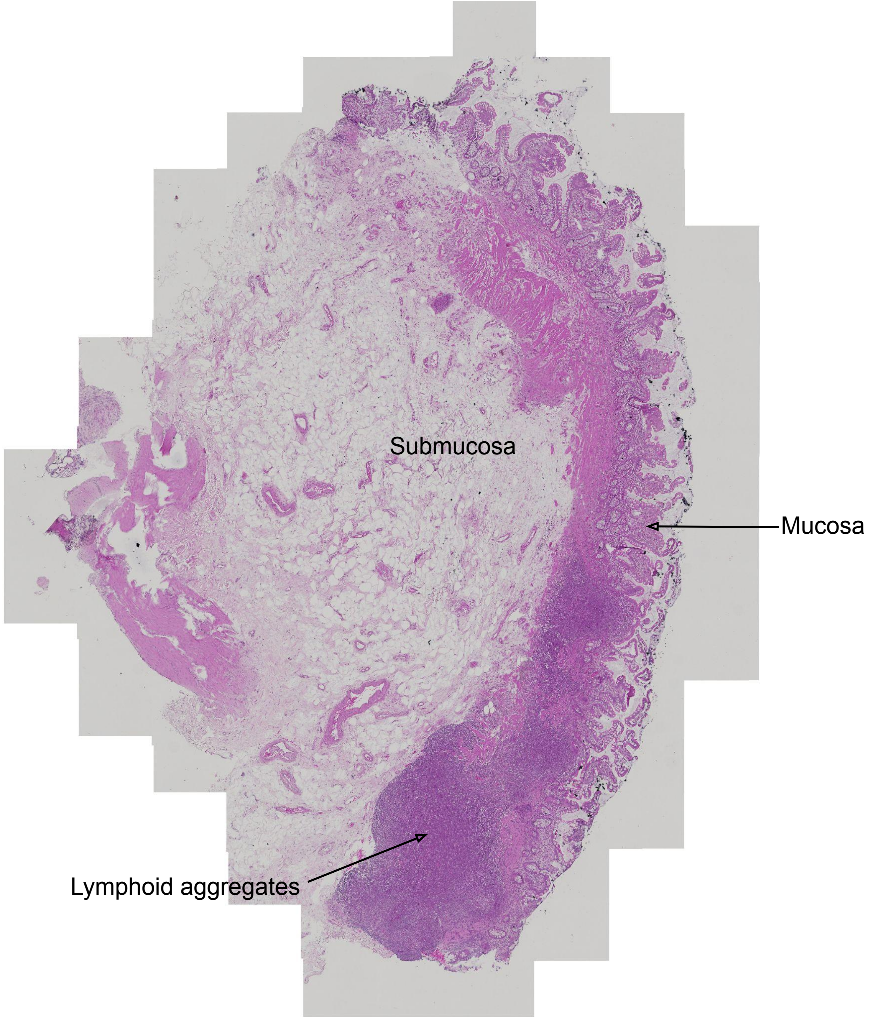

TIP 864

INFLAMMATORY CD
ILEUM

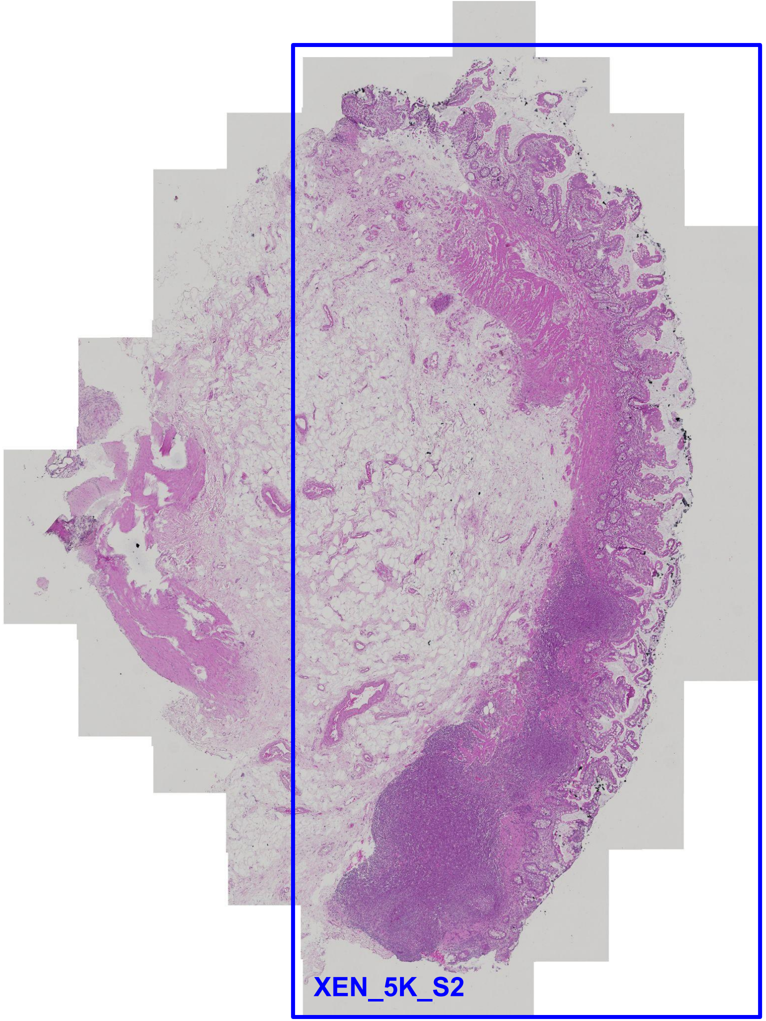

GI 7051

INFLAMMATORY CD ILEUM

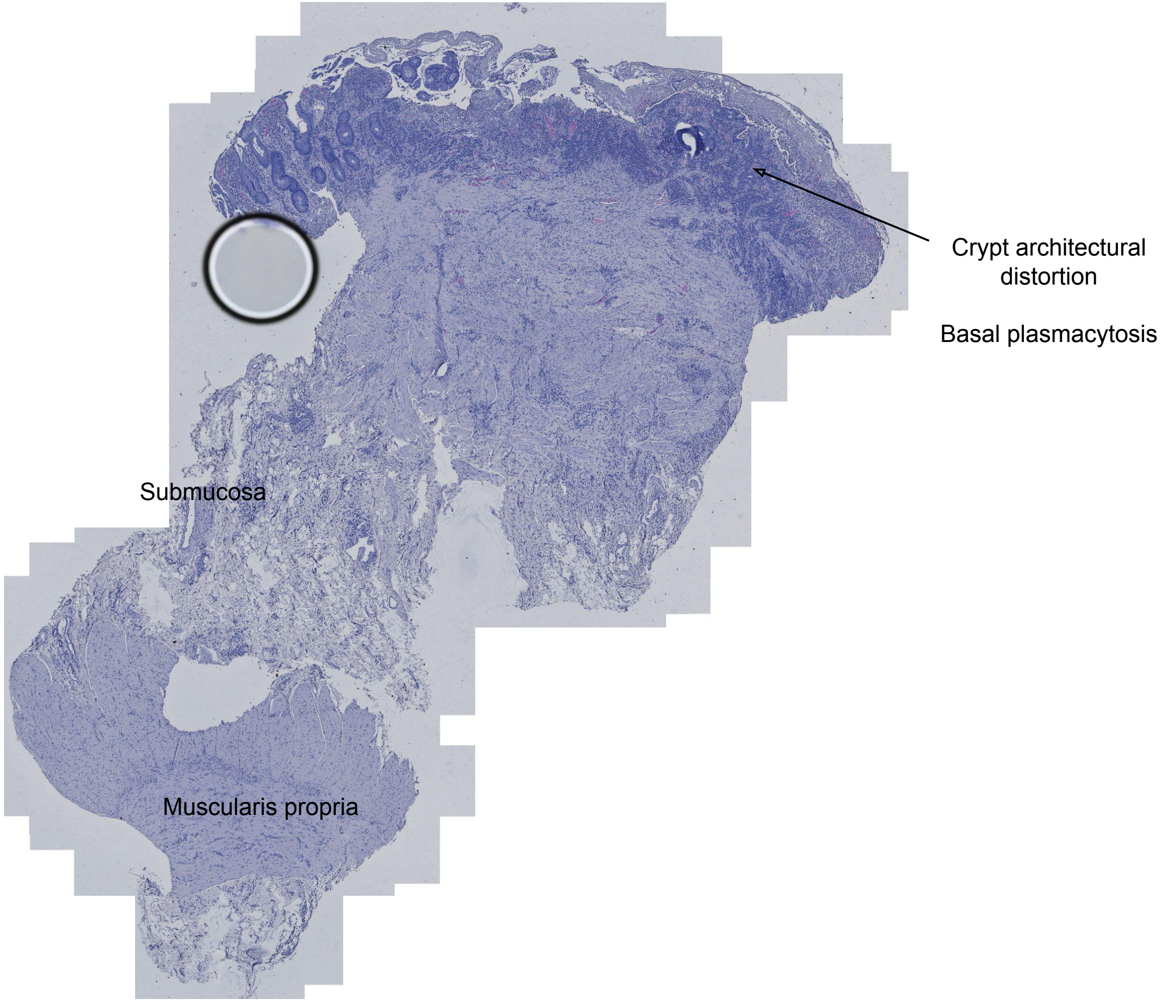

GI 7051

INFLAMMATORY CD
ILEUM

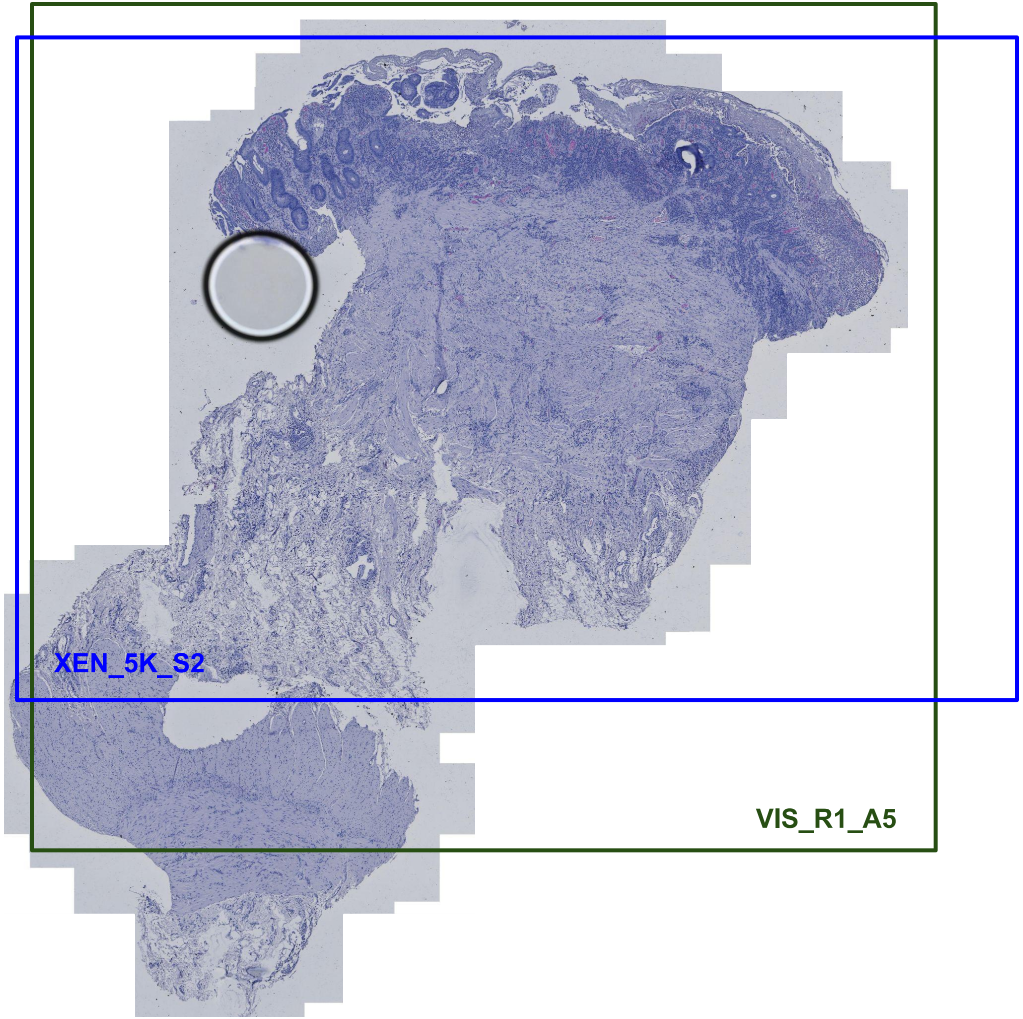

GI 7051

INFLAMMATORY CD
ILEUM

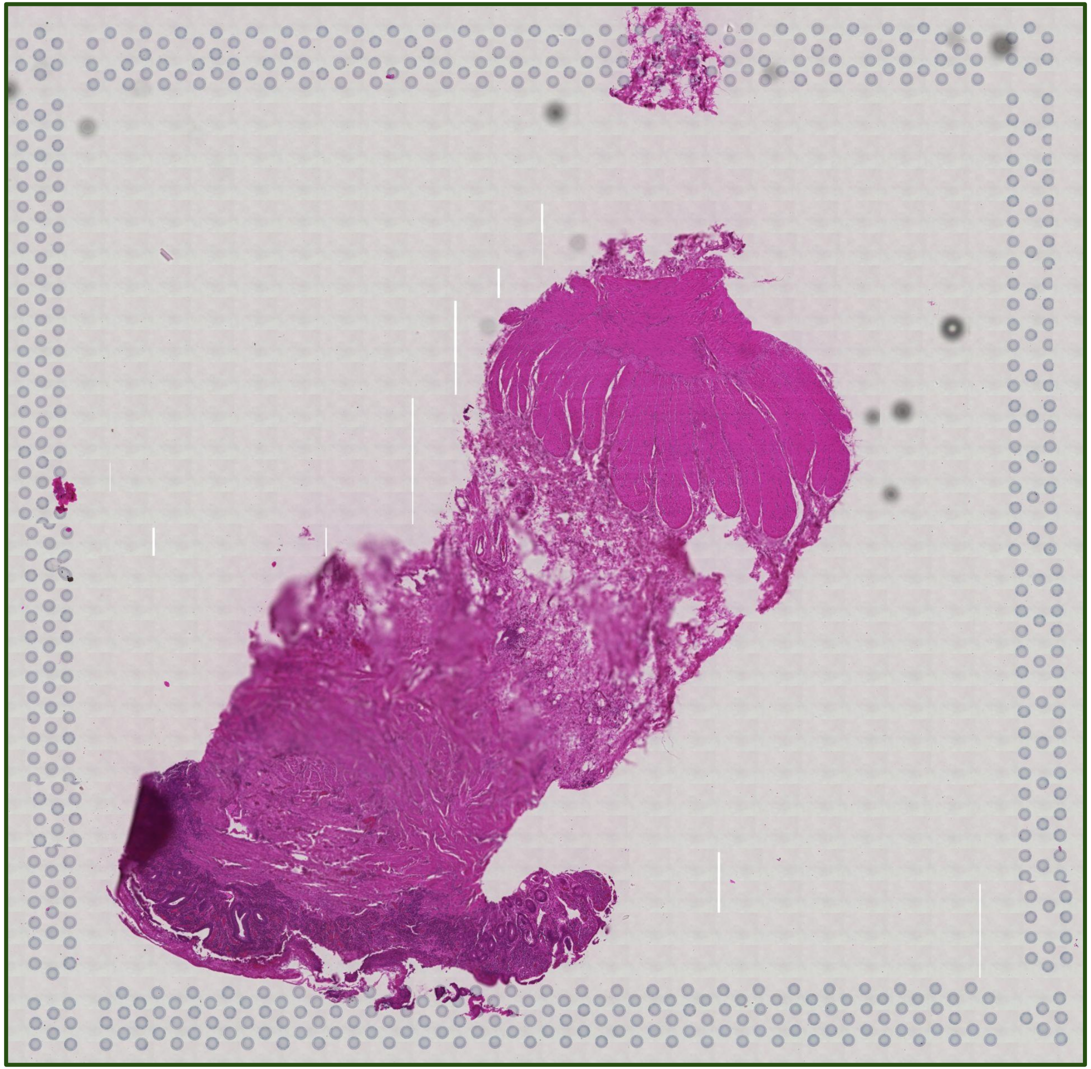

GI 7595

STRICTURED CD ILEUM

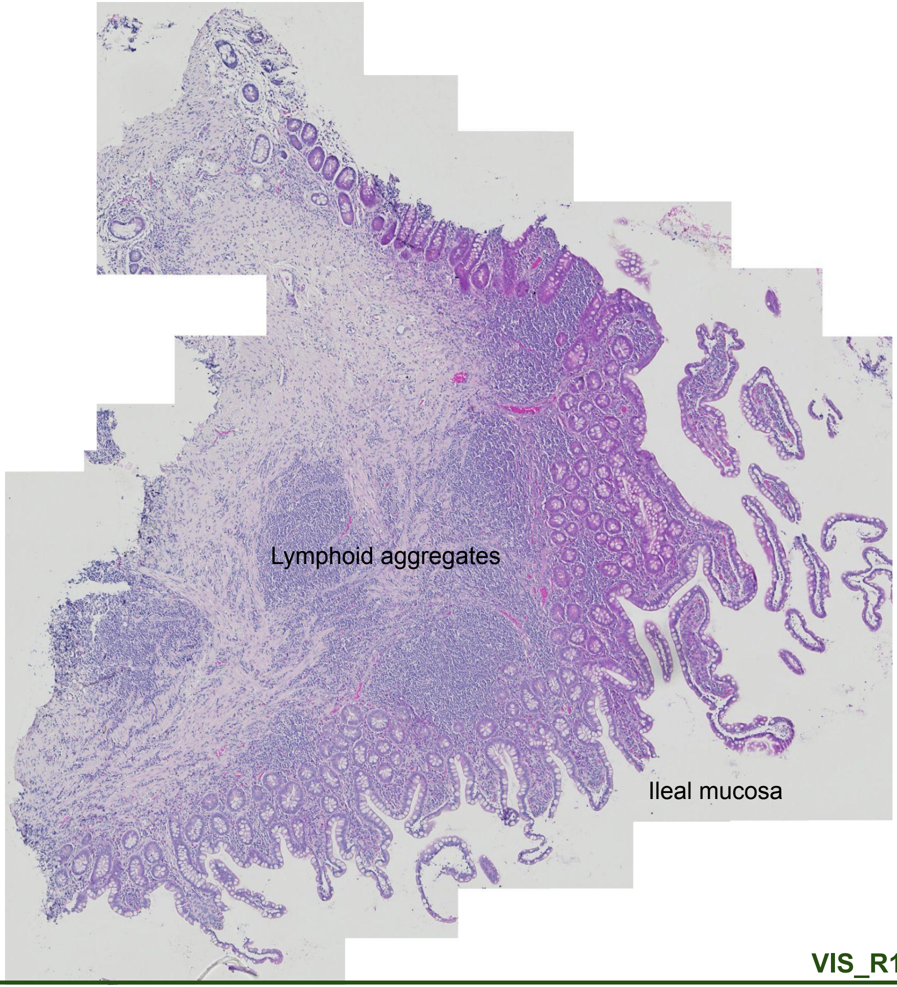

VIS_R1_A7

GI 7595

STRICTURED CD ILEUM

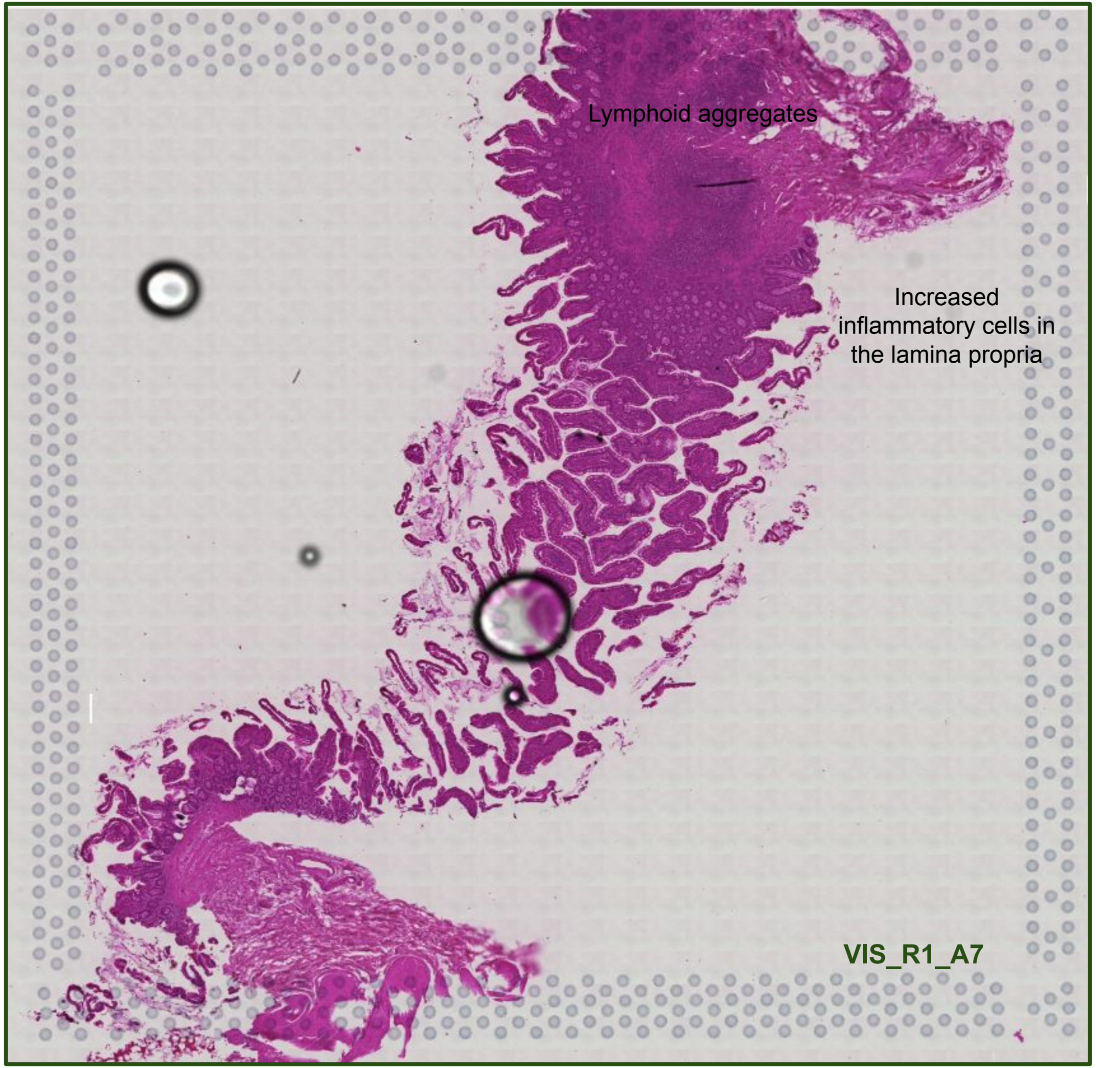

GI 9662

INFLAMMATORY CD

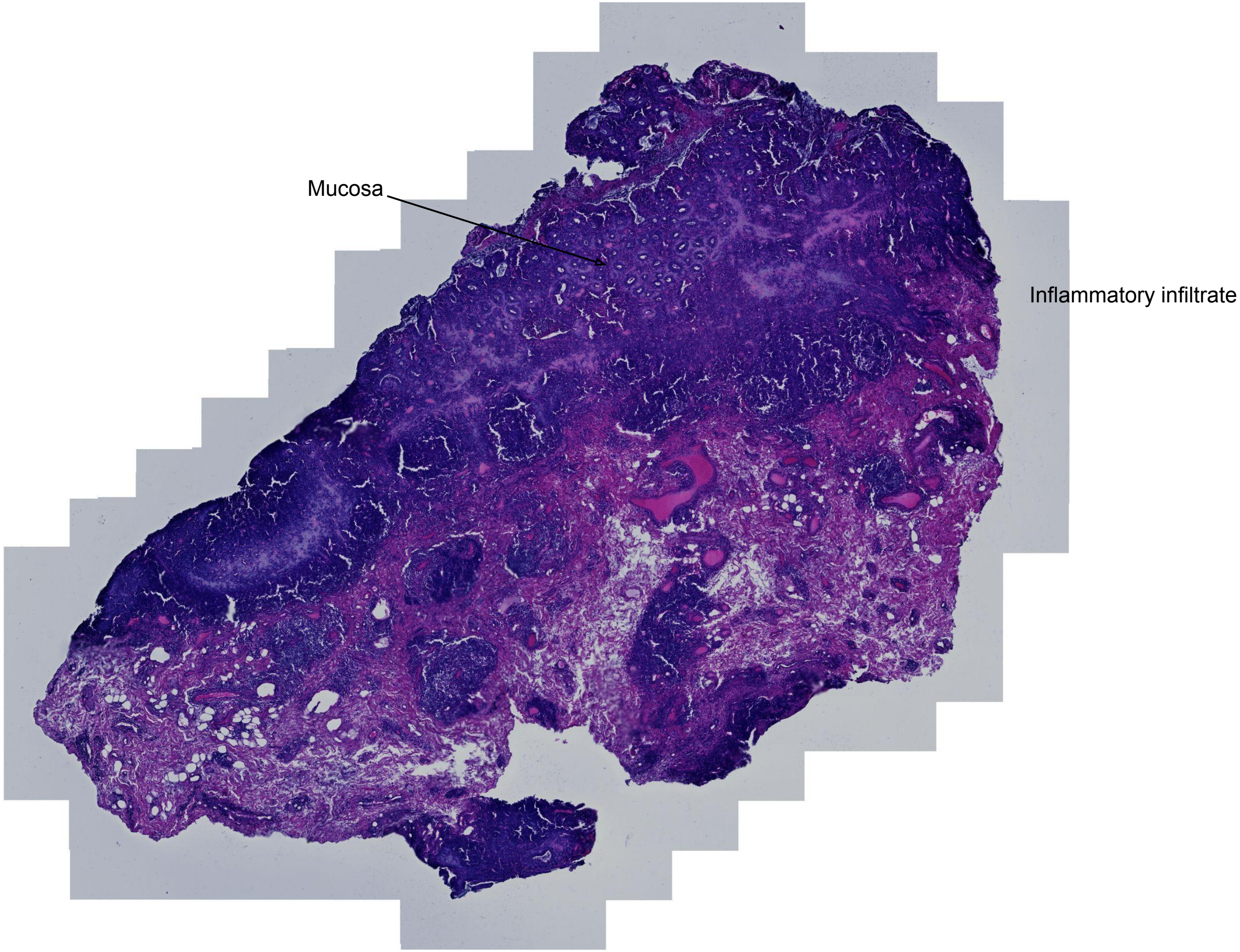

GI 9662

INFLAMMATORY CD

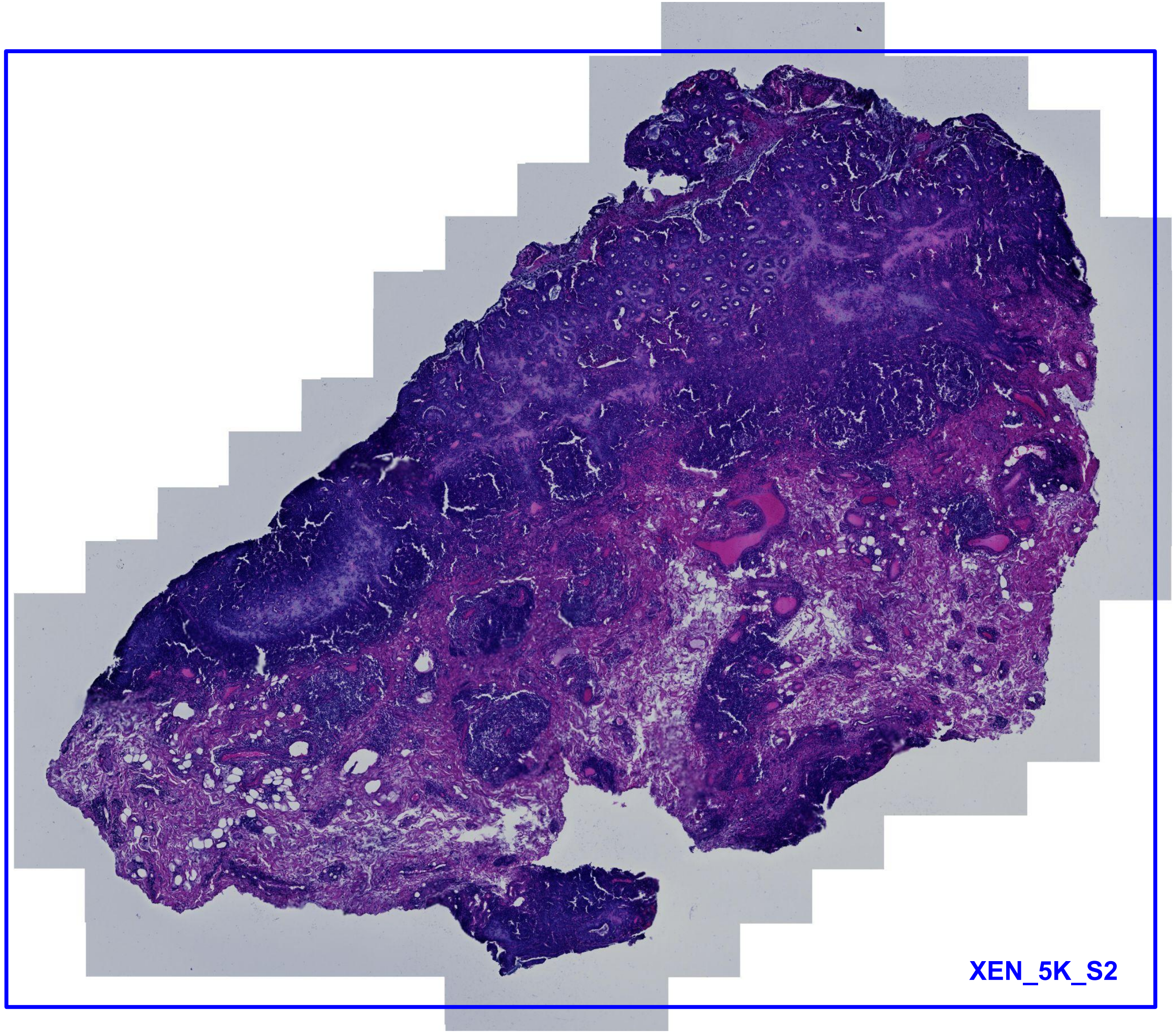

TIP 852

HEALTHY ILEUM

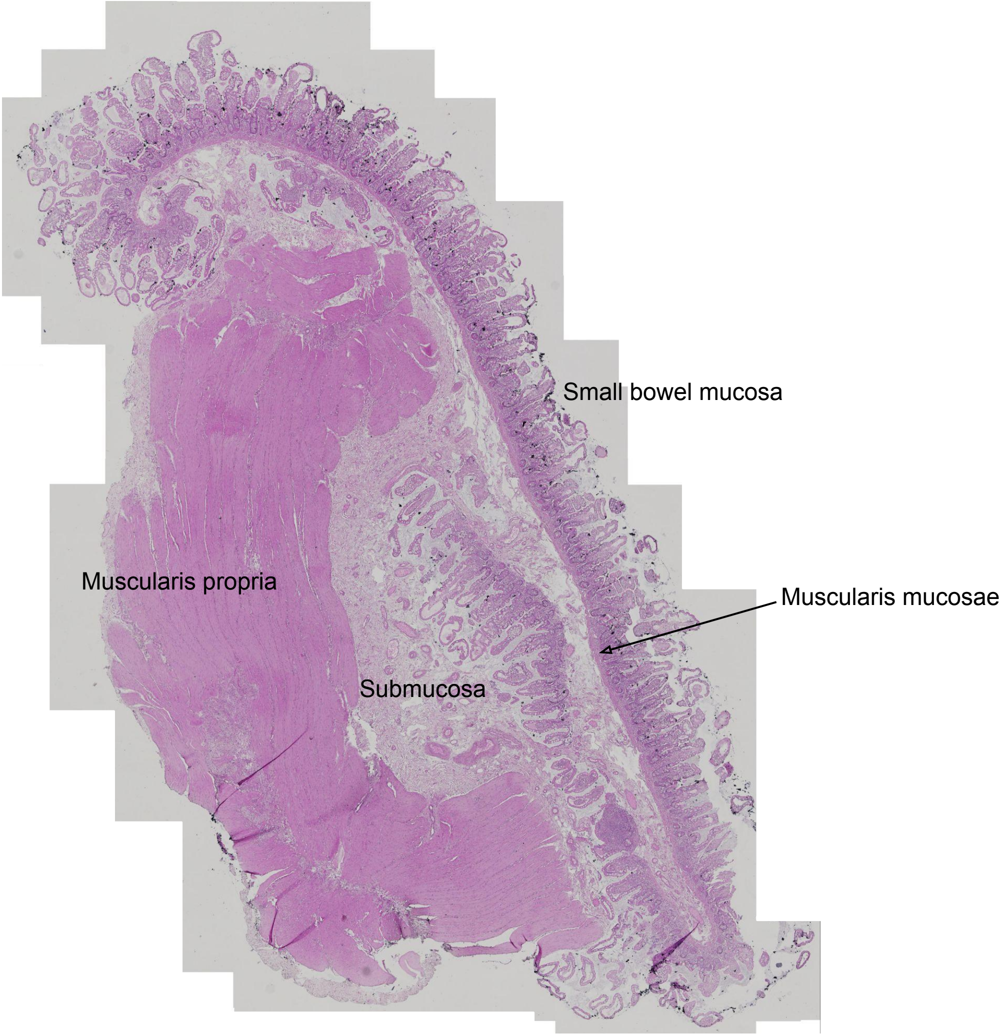

TIP 852

HEALTHY ILEUM

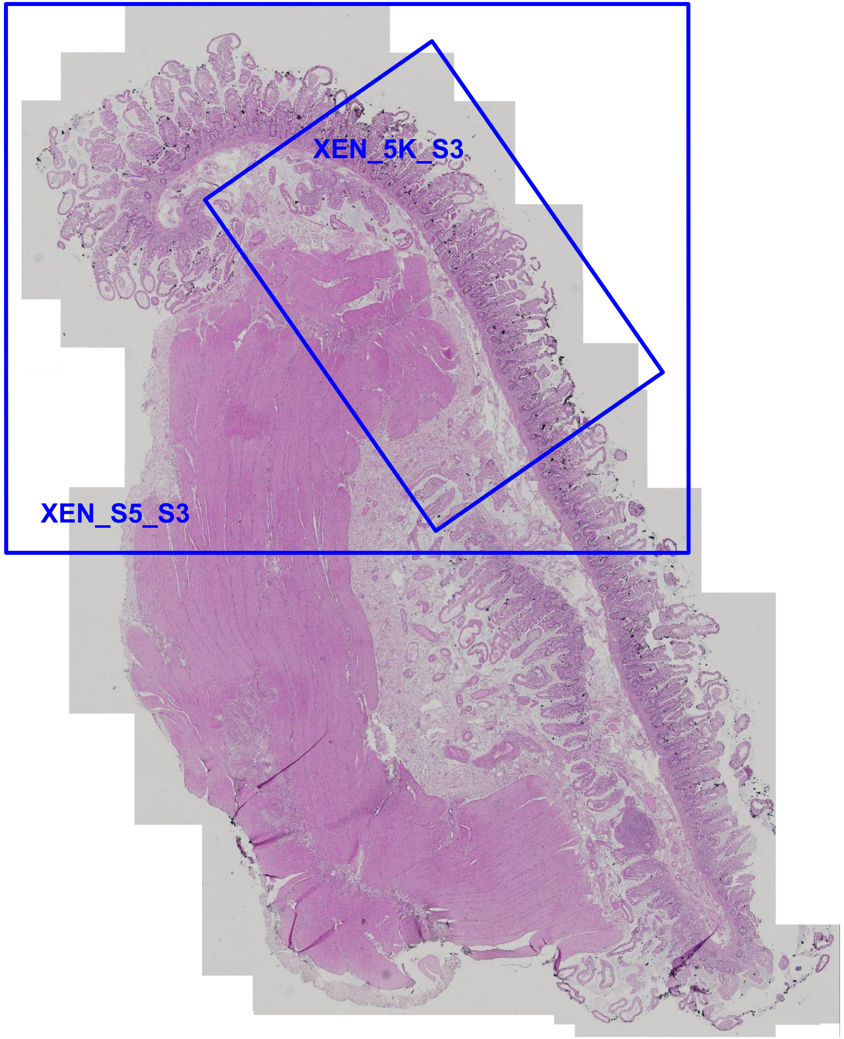

CAM 005

HEALTHY COLON

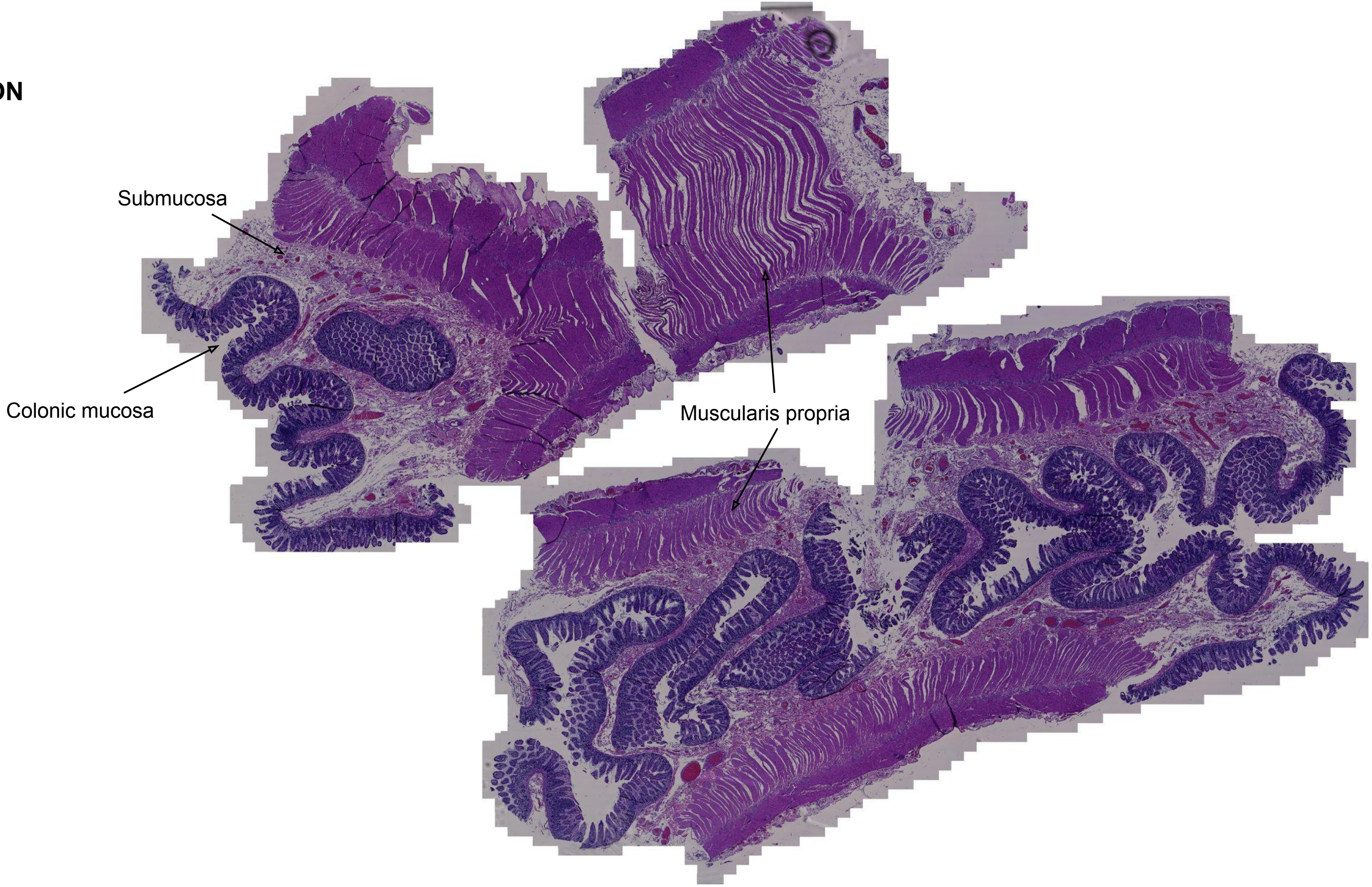

CAM 005

HEALTHY COLON

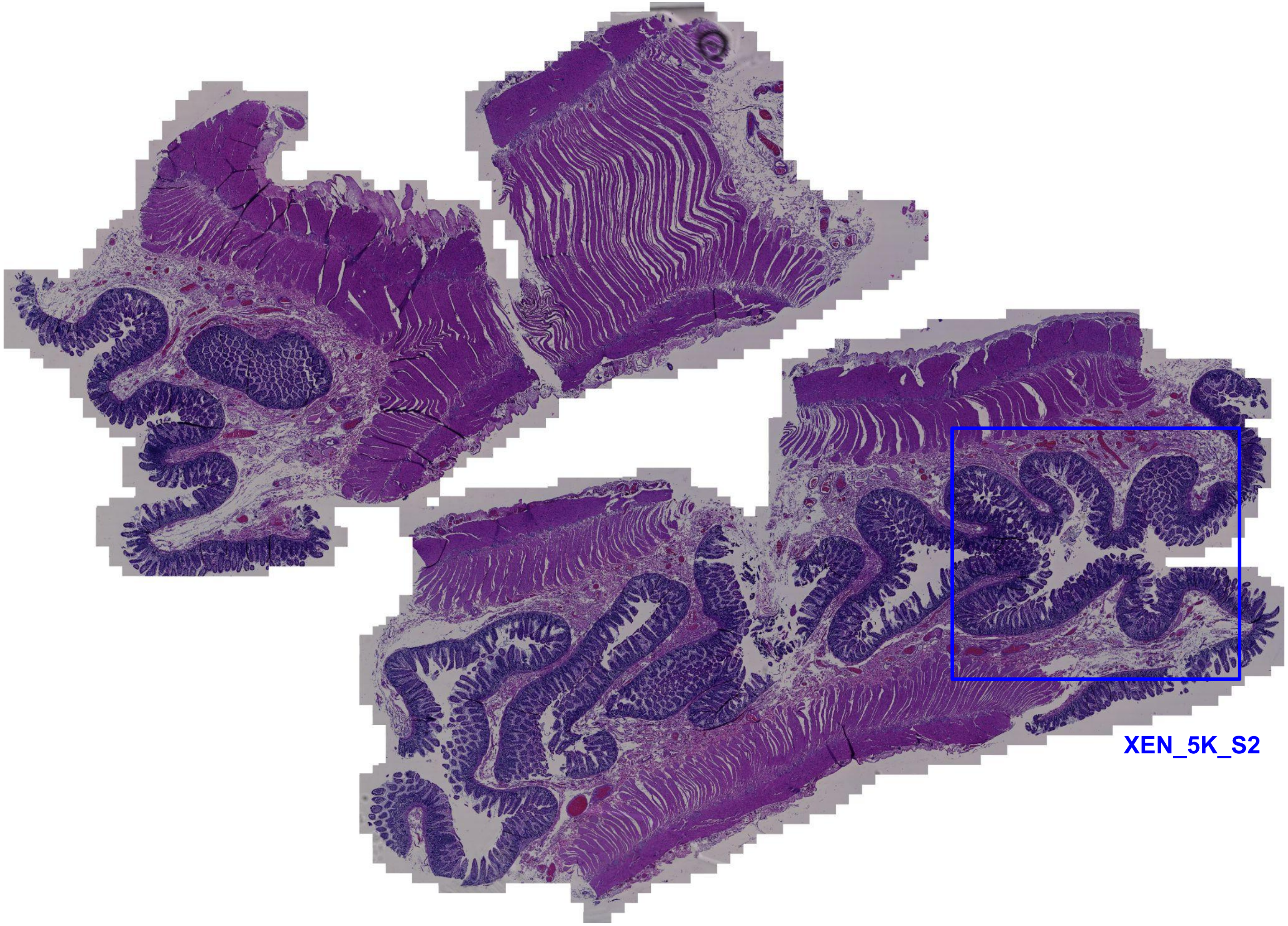

CAM 006

HEALTHY COLON

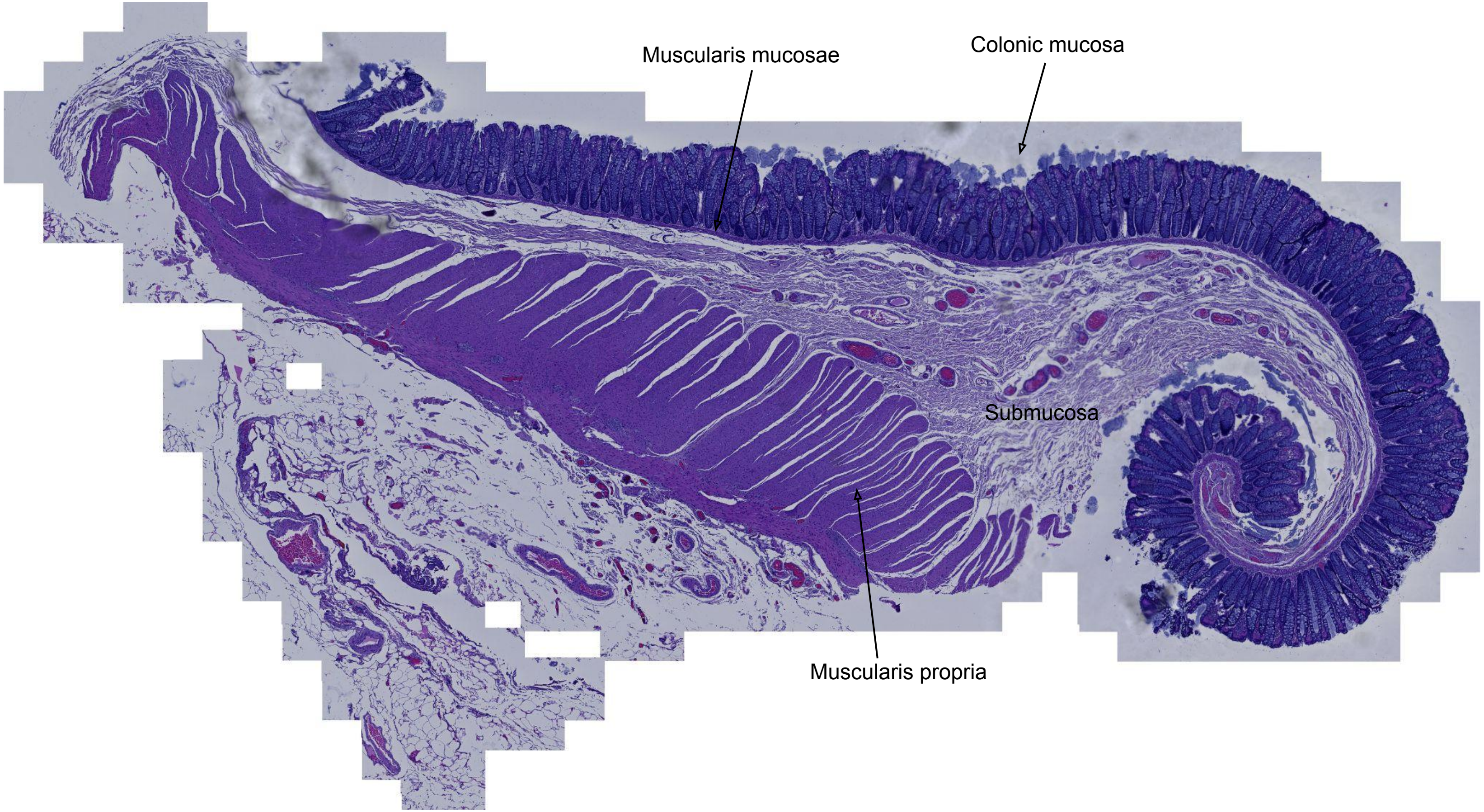

CAM 006

HEALTHY COLON

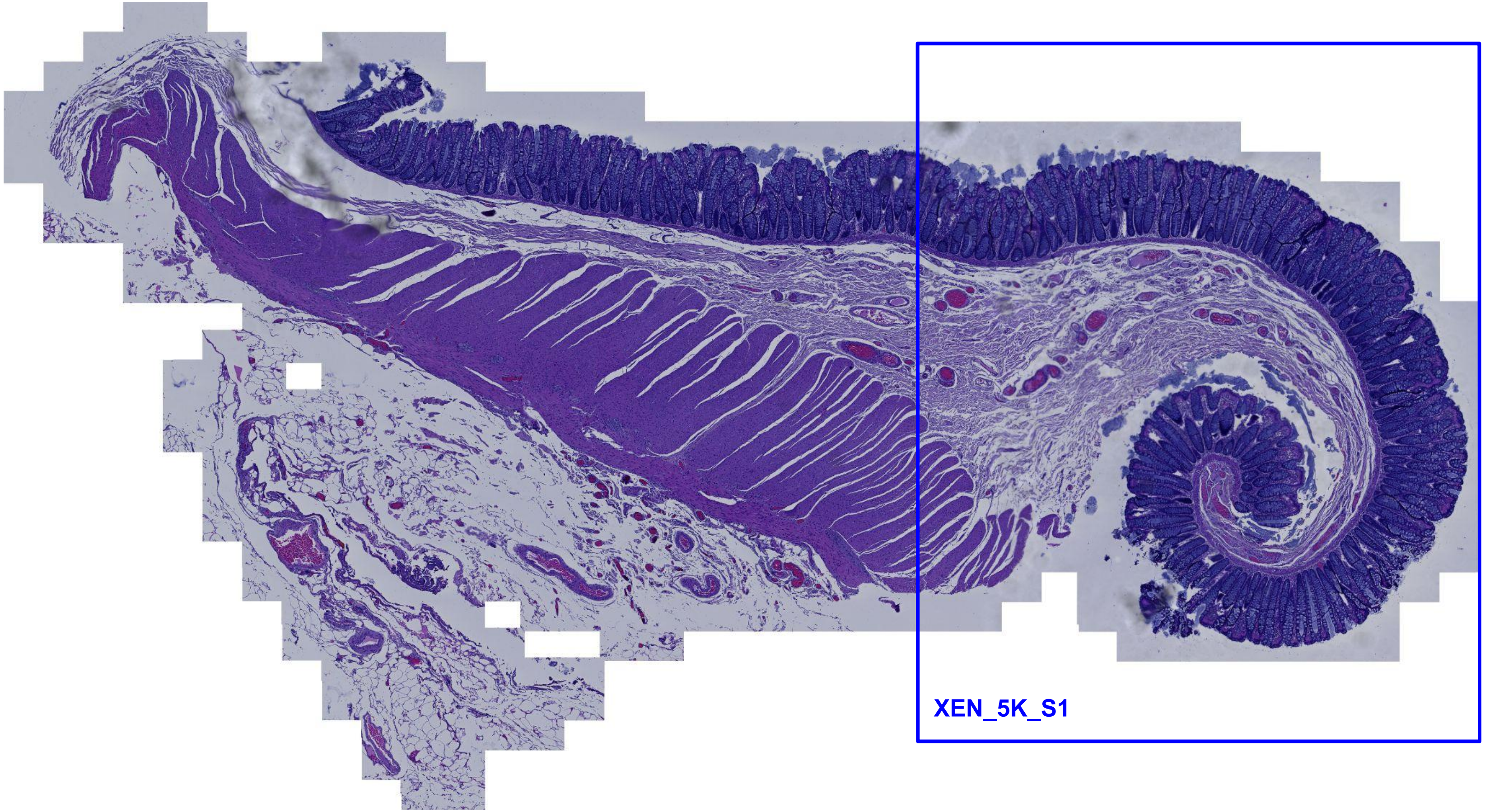

TIP 571

HEALTHY COLON

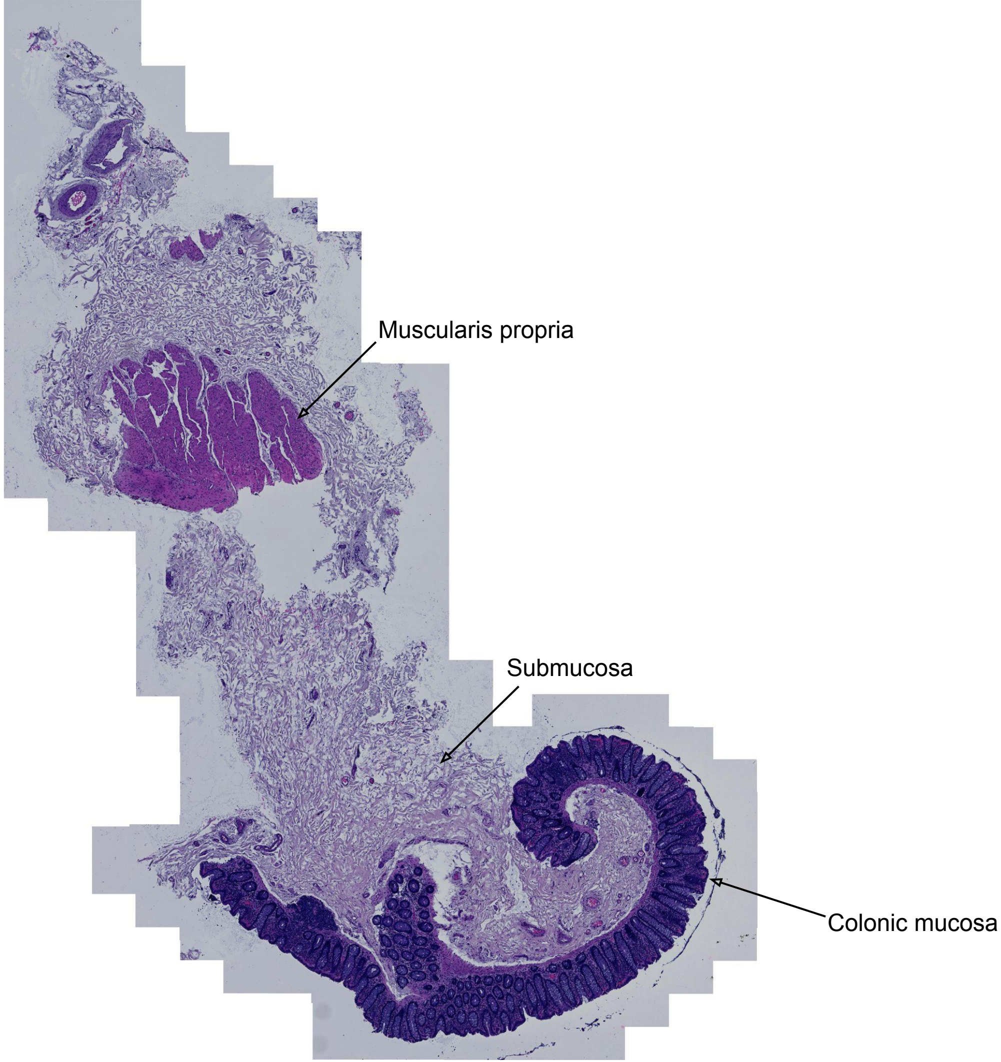

TIP 571

HEALTHY COLON

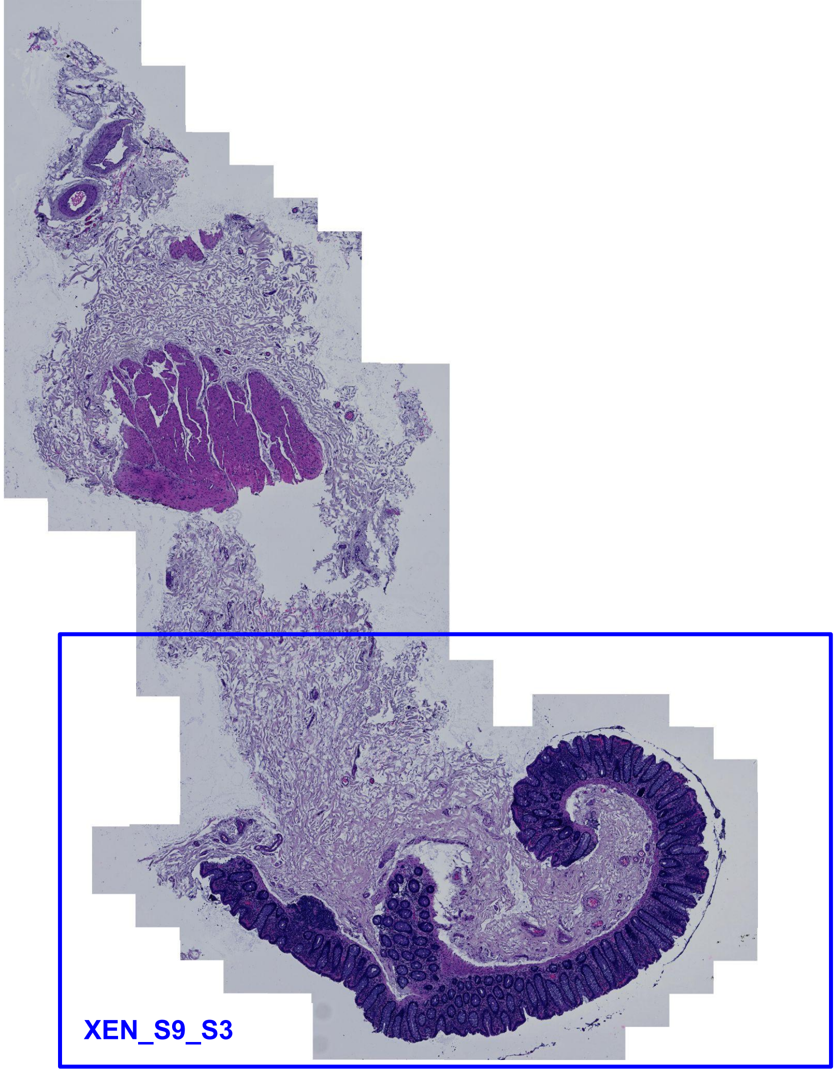

TIP 471

HEALTHY COLON

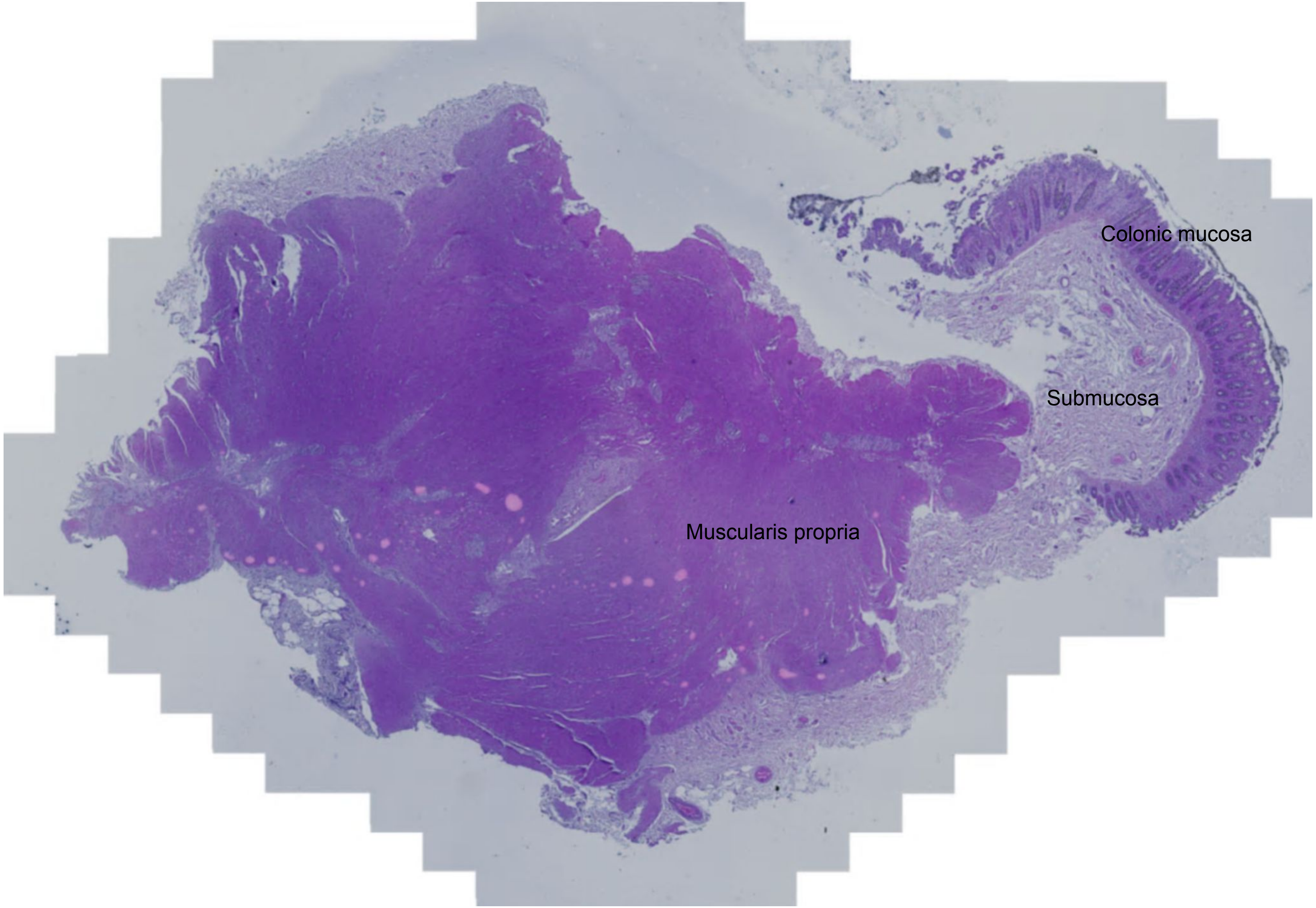

TIP 471

HEALTHY COLON

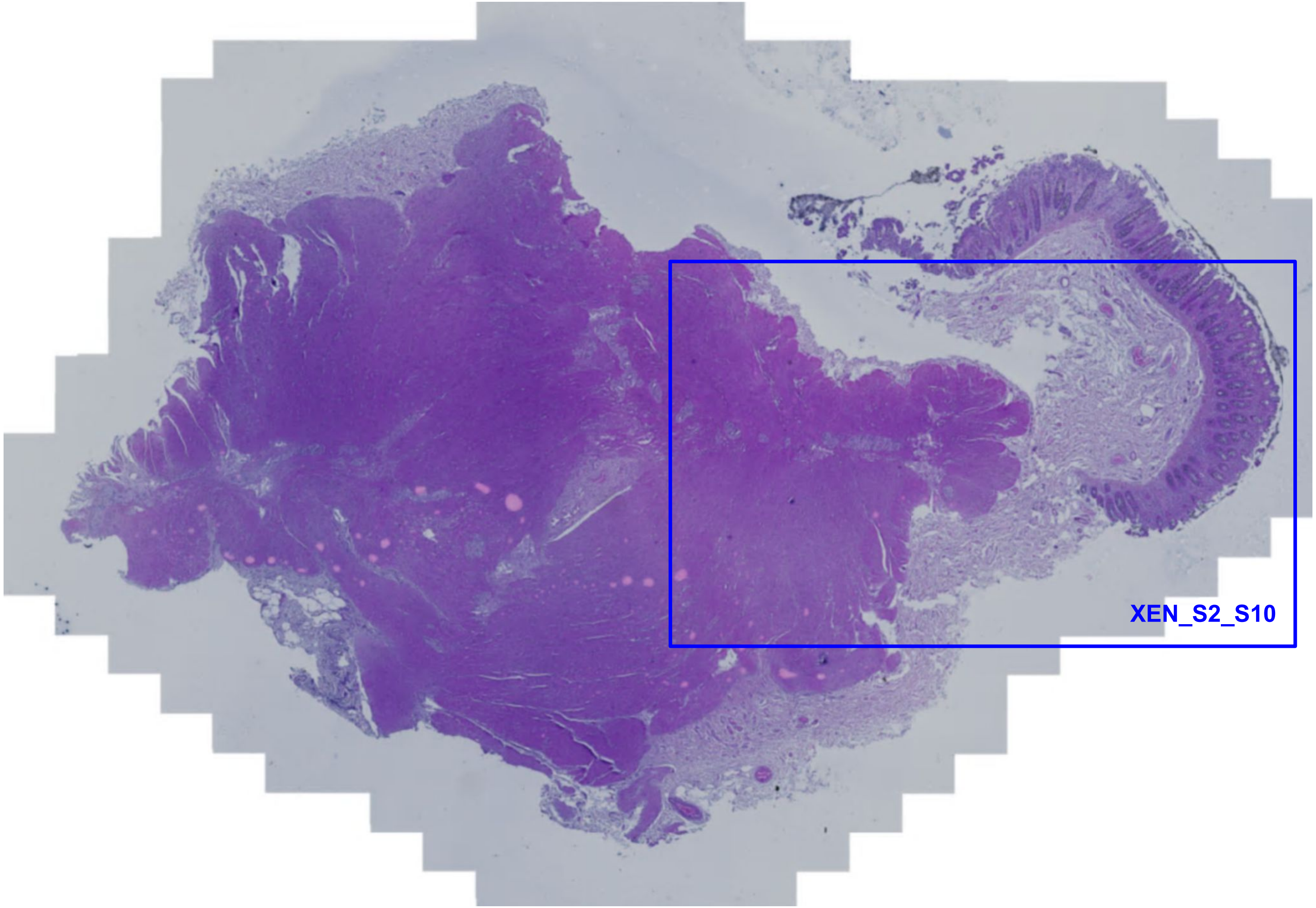

TIP 522

HEALTHY ILEUM

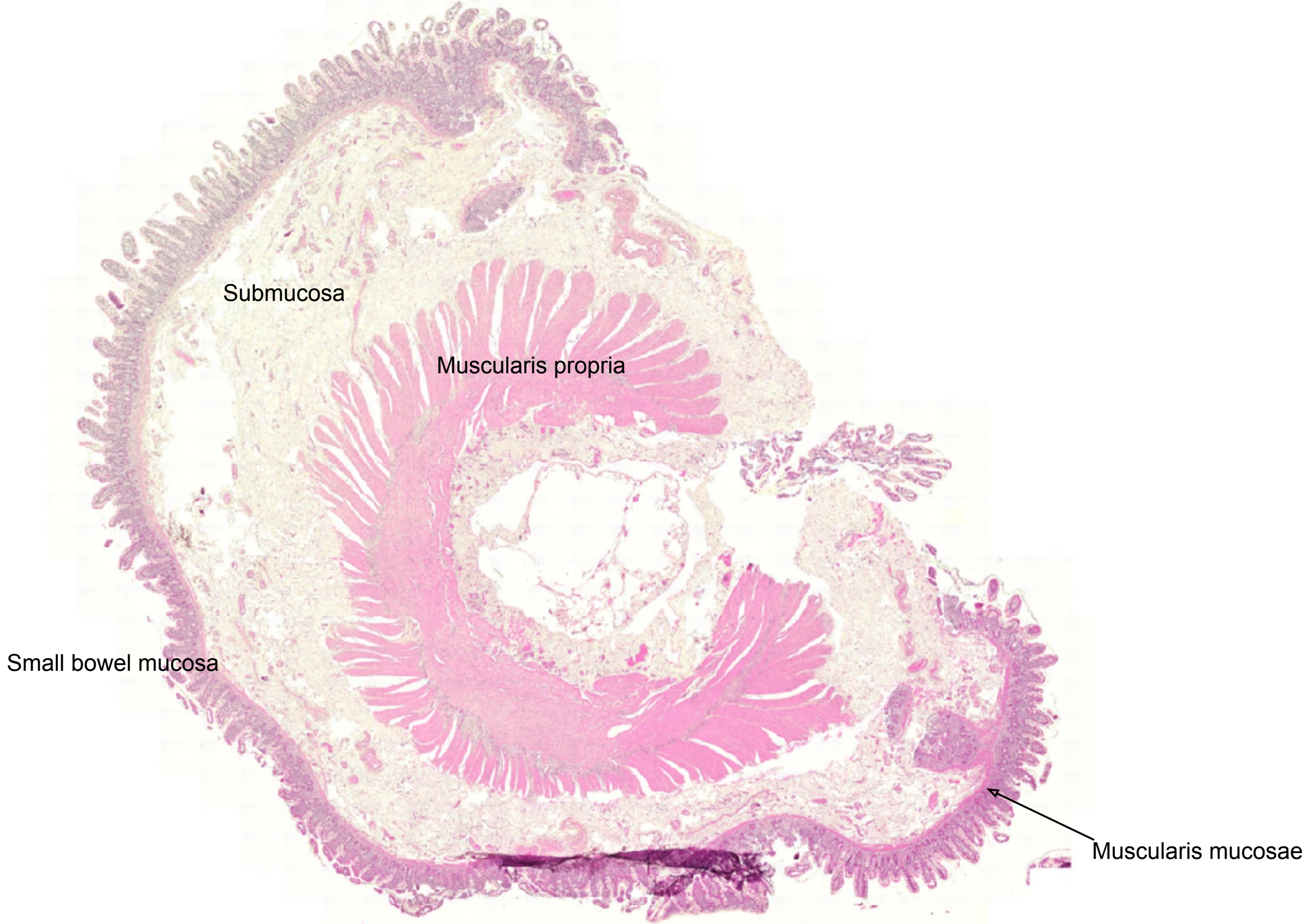

TIP 522

HEALTHY ILEUM

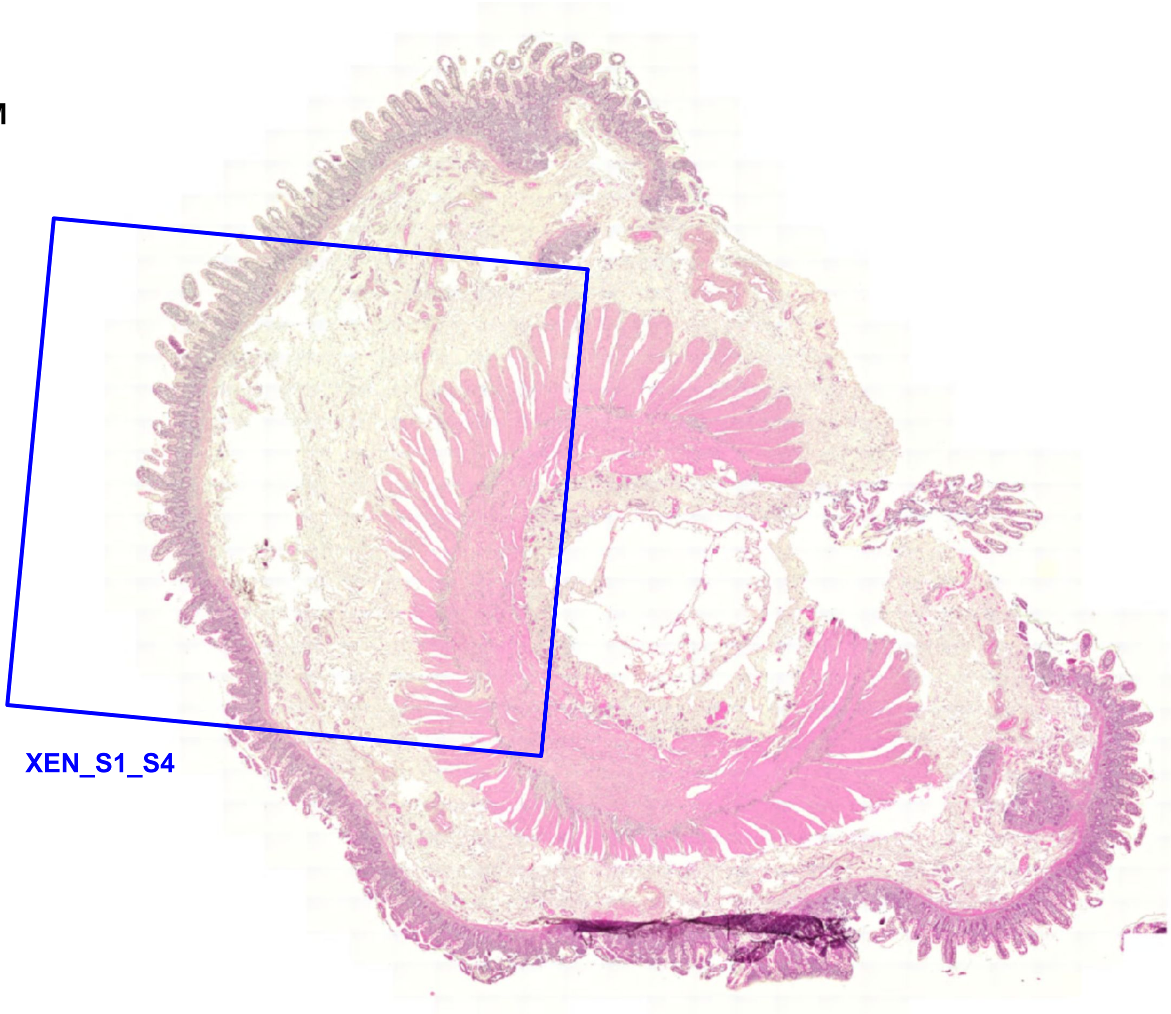

TIP 522

HEALTHY ILEUM MUCOSA

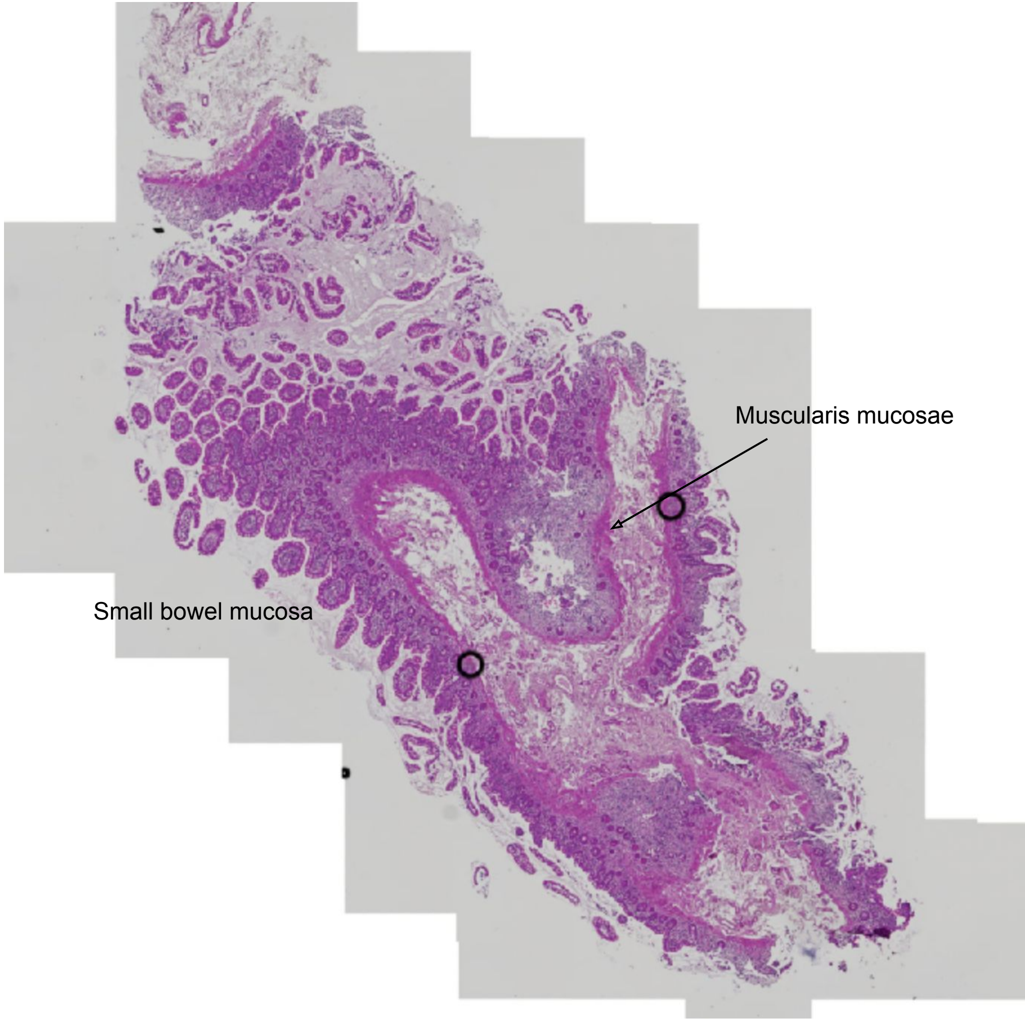

TIP 522

HEALTHY ILEUM MUCOSA

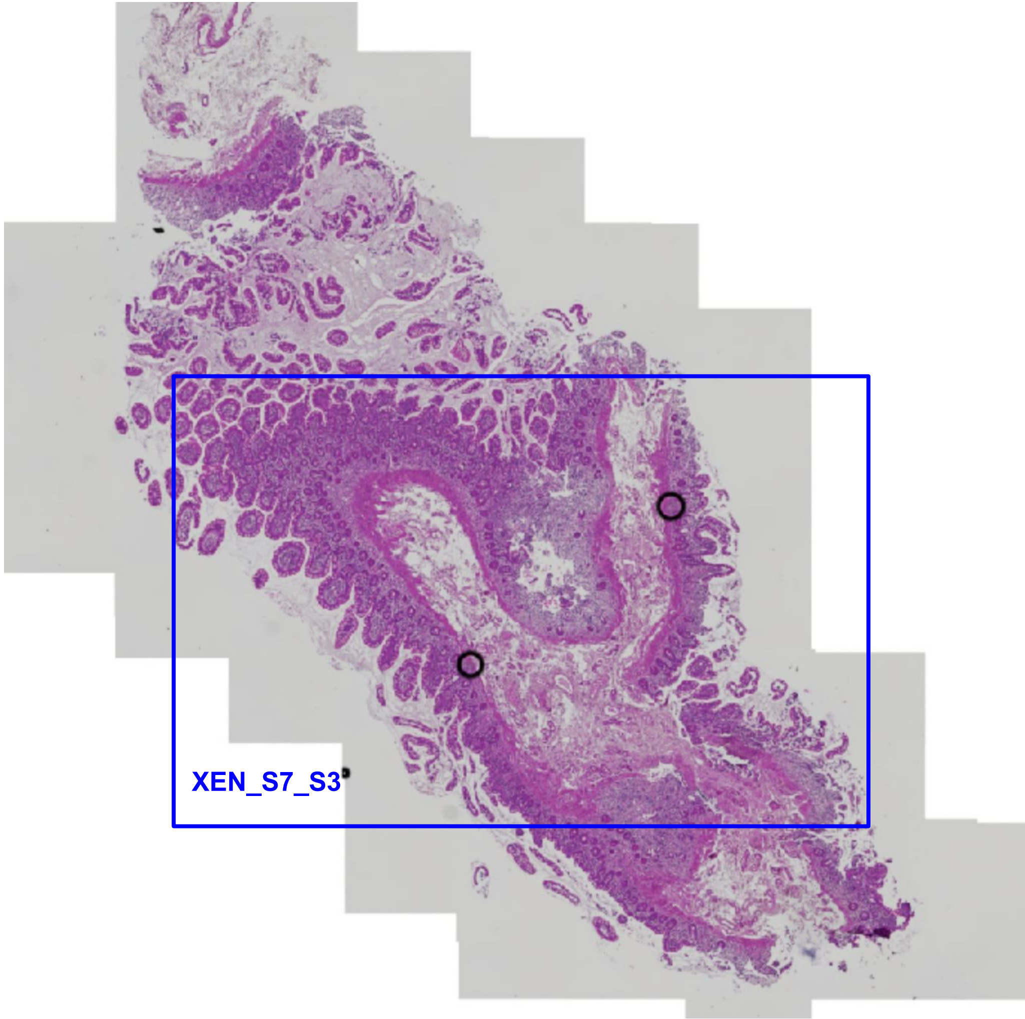

TIP 522

HEALTHY ILEUM

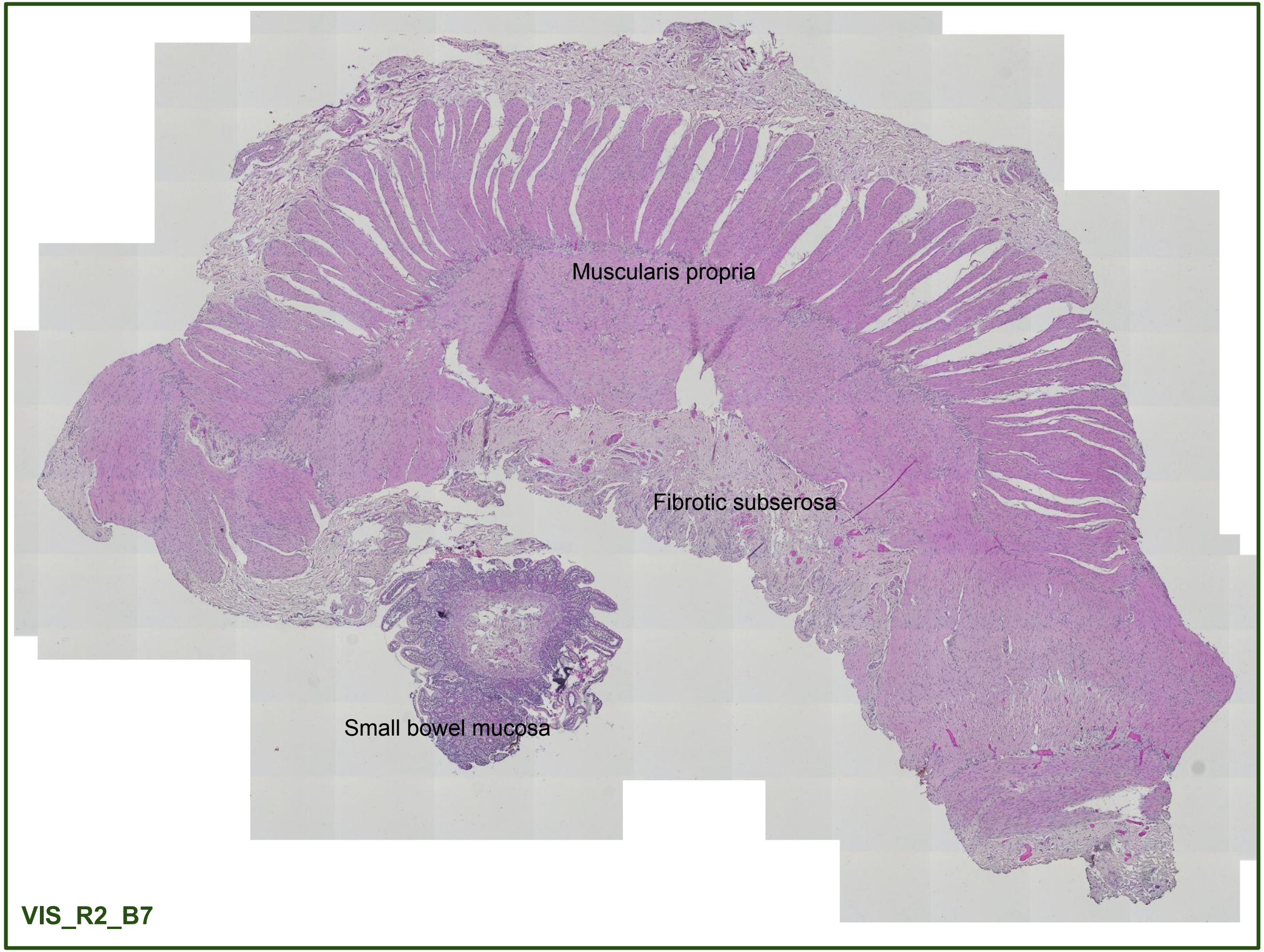

TIP 522

HEALTHY ILEUM

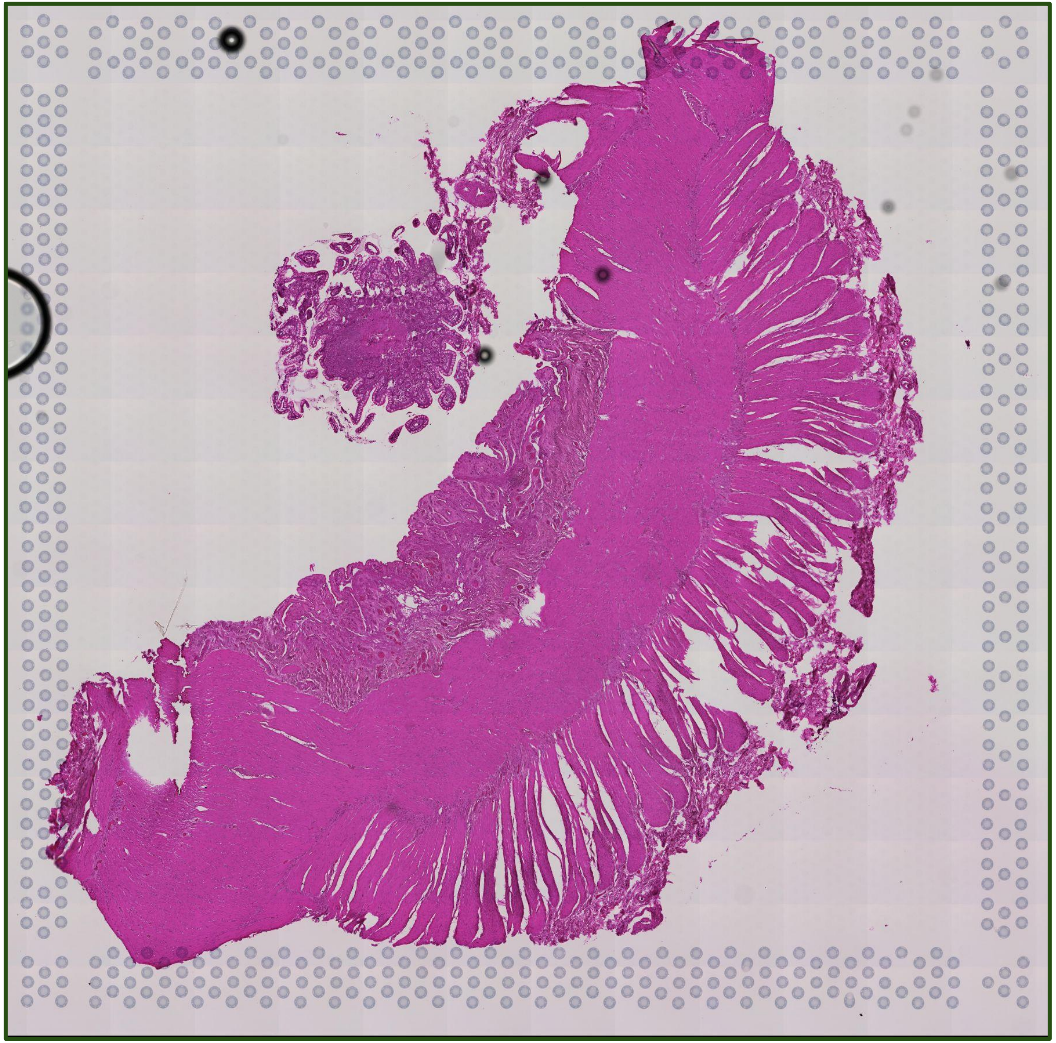

TIP 525

HEALTHY ILEUM

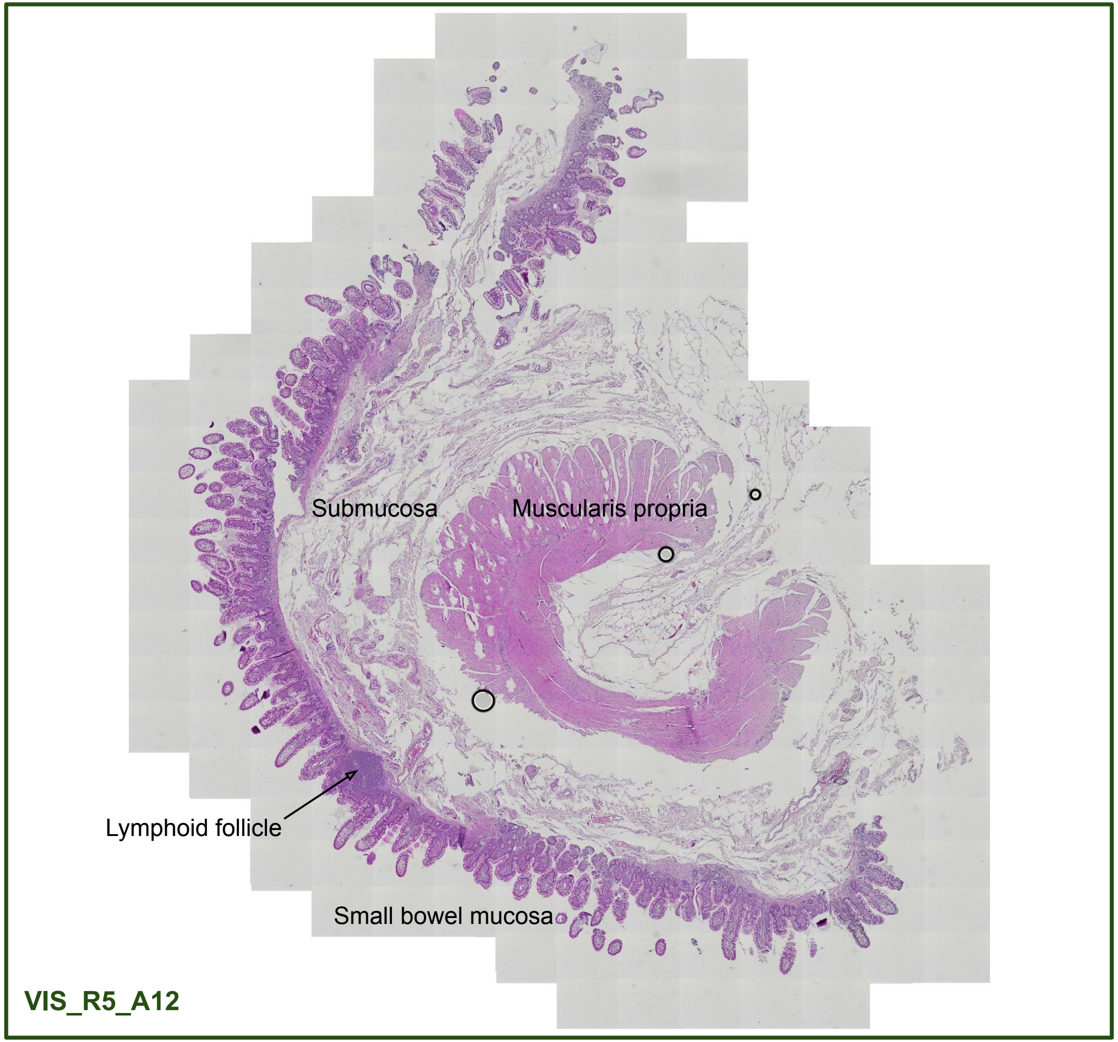

TIP 525

HEALTHY ILEUM

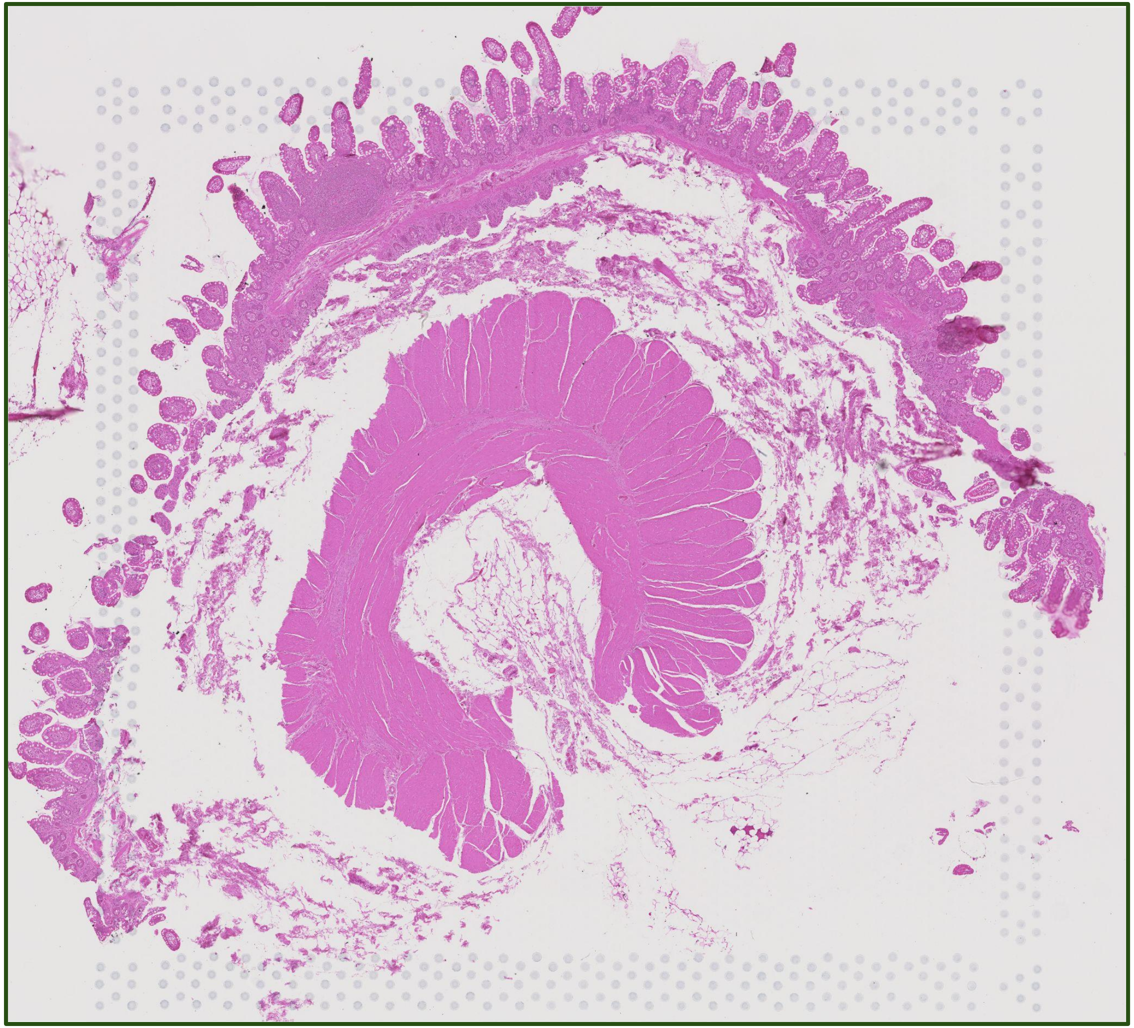

TIP 599

HEALTHY COLON

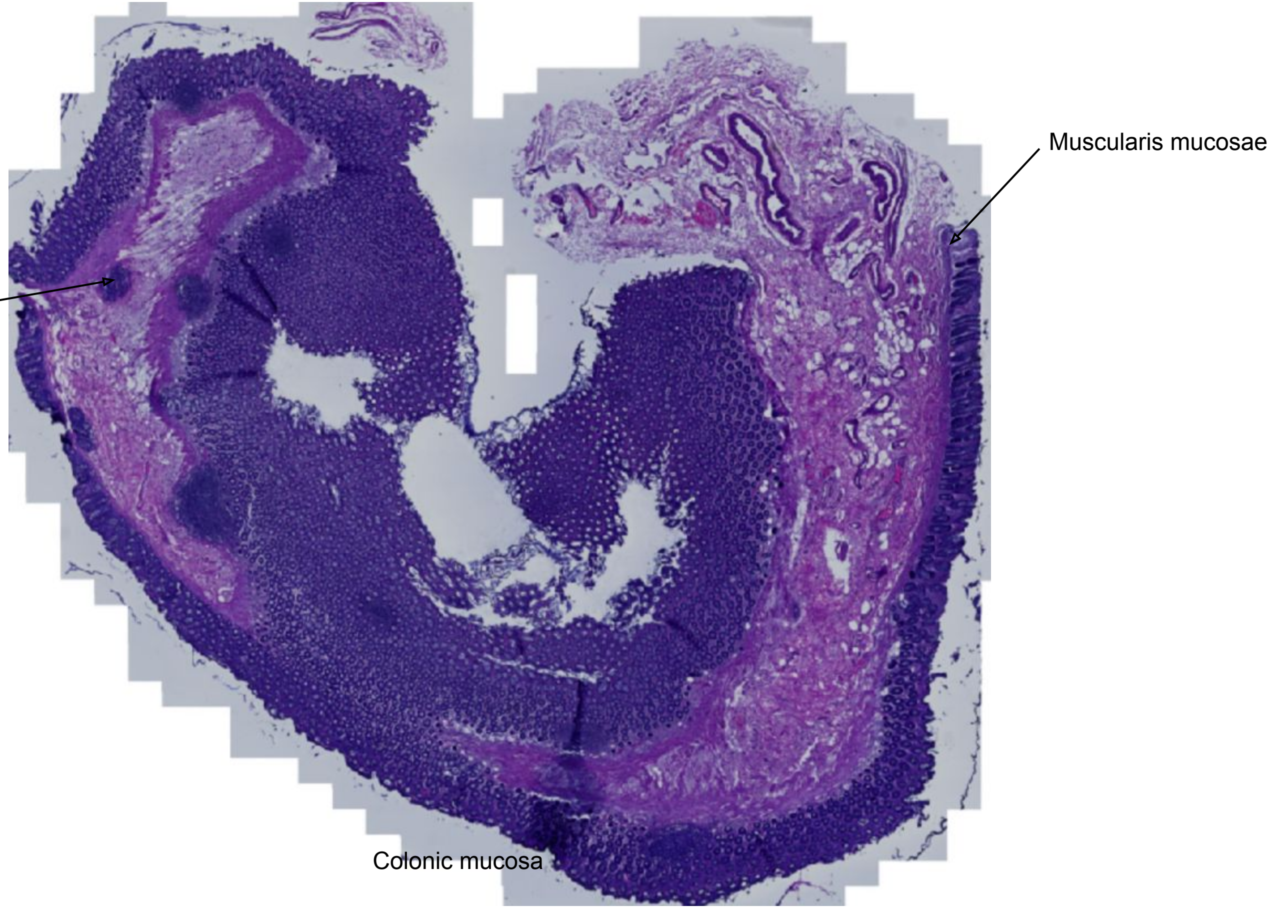

TIP 599

HEALTHY COLON

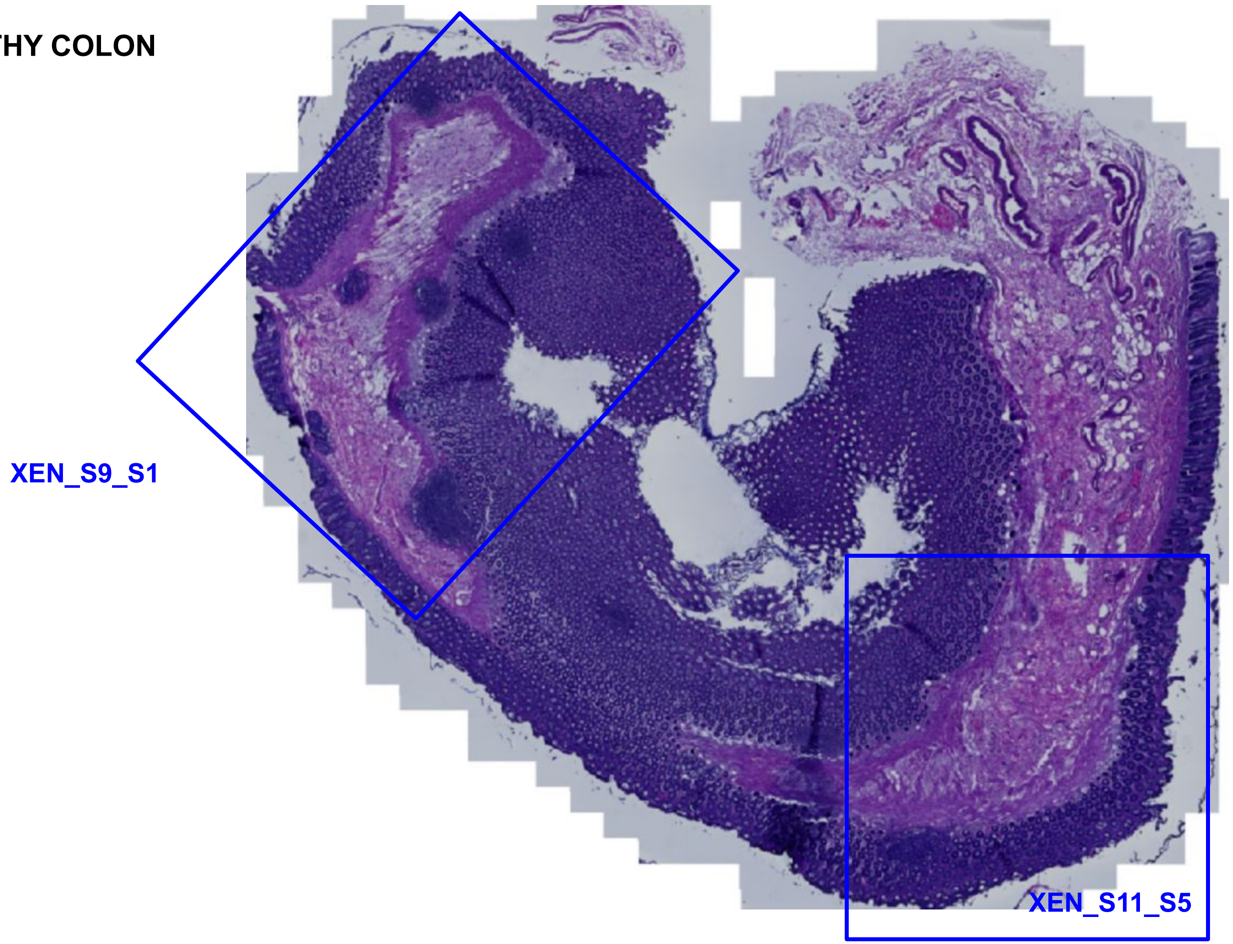

GI 6968

HEALTHY ILEUM

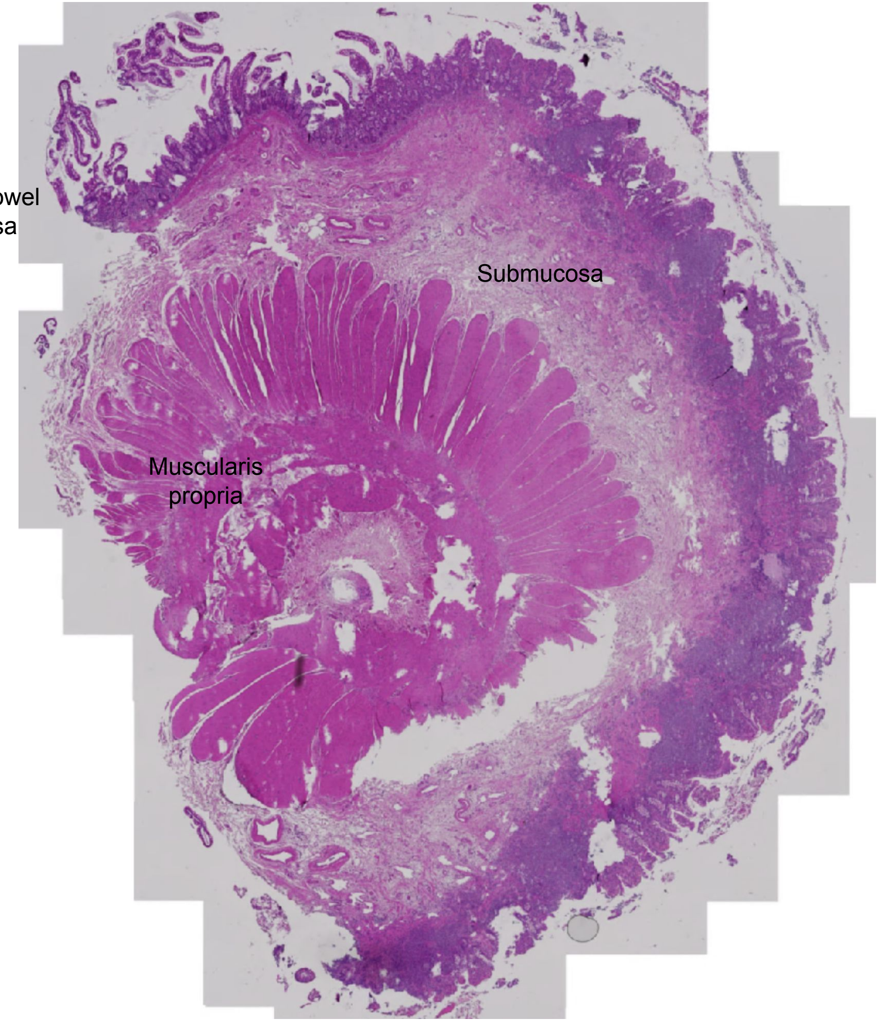

GI 6968

HEALTHY ILEUM

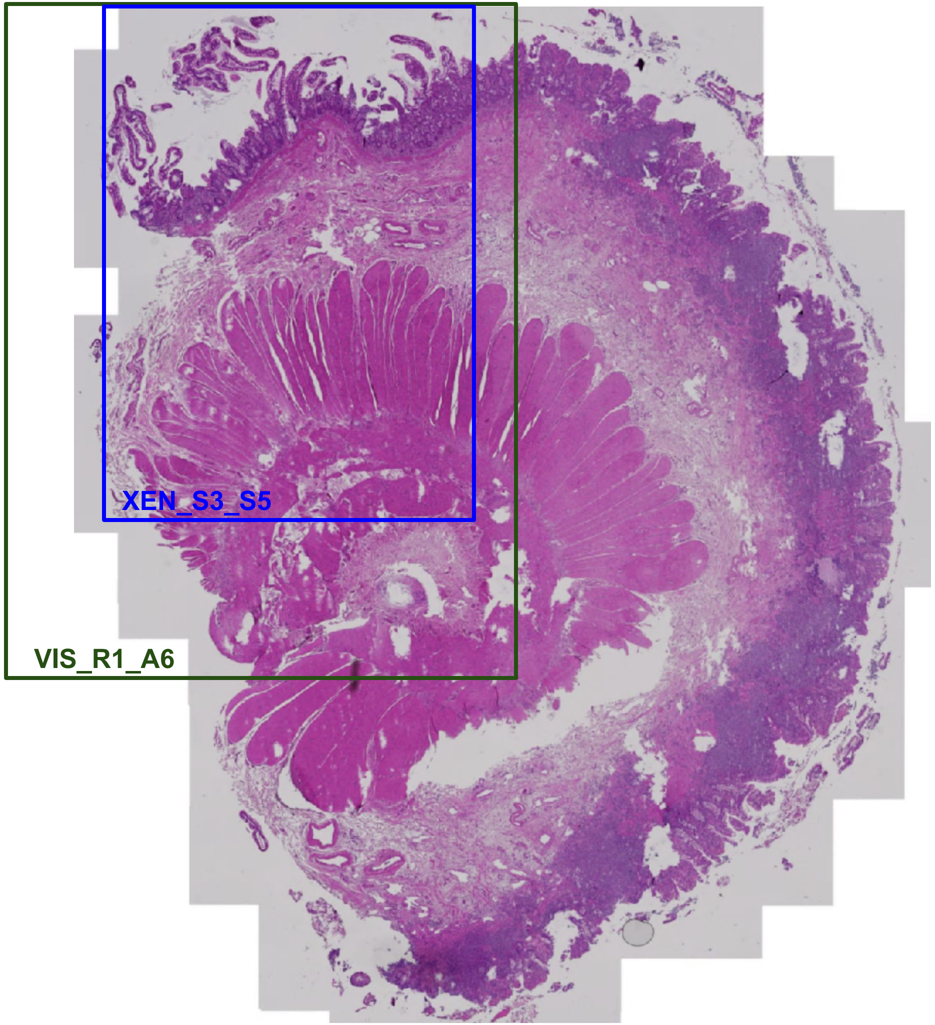

GI 6968

HEALTHY ILEUM

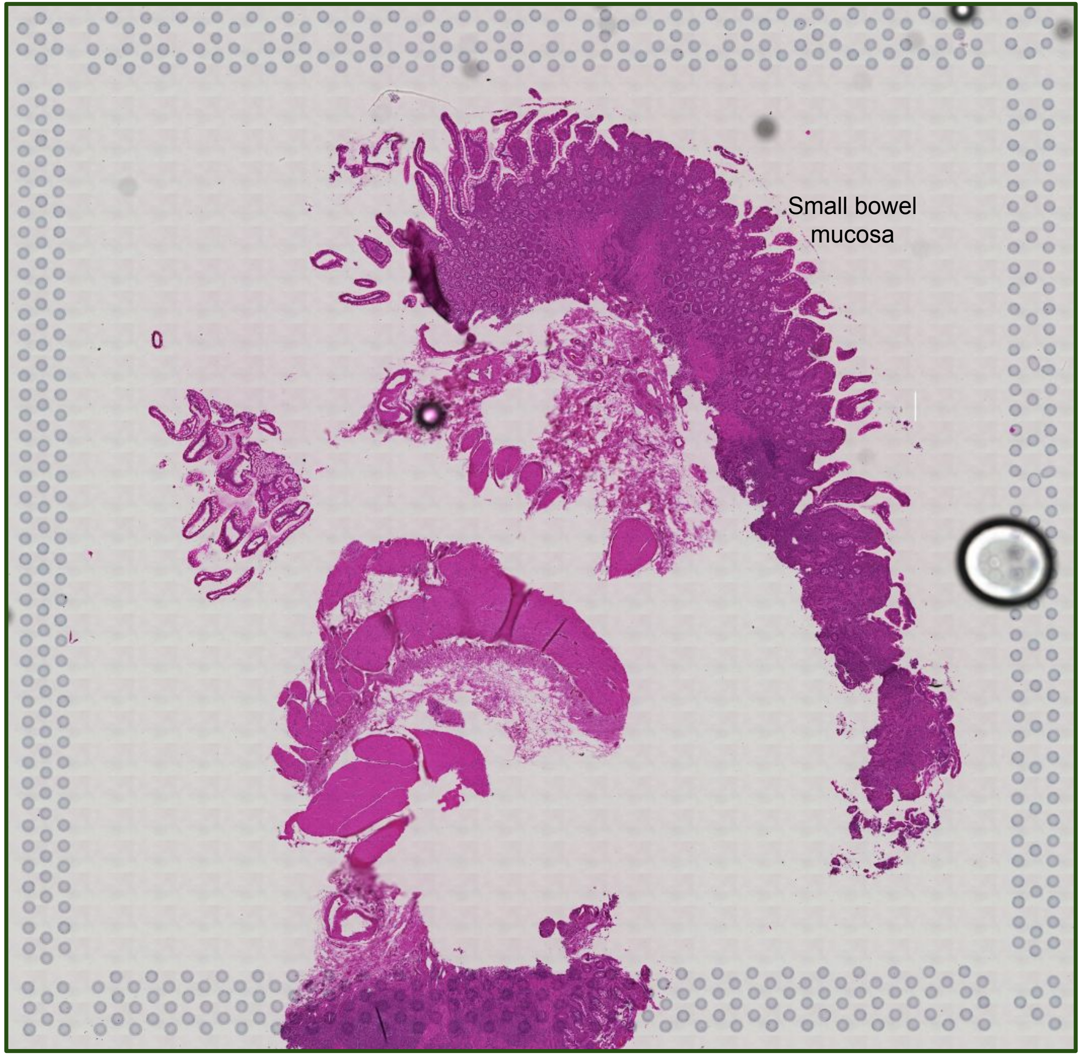

GI 3391

HEALTHY COLON

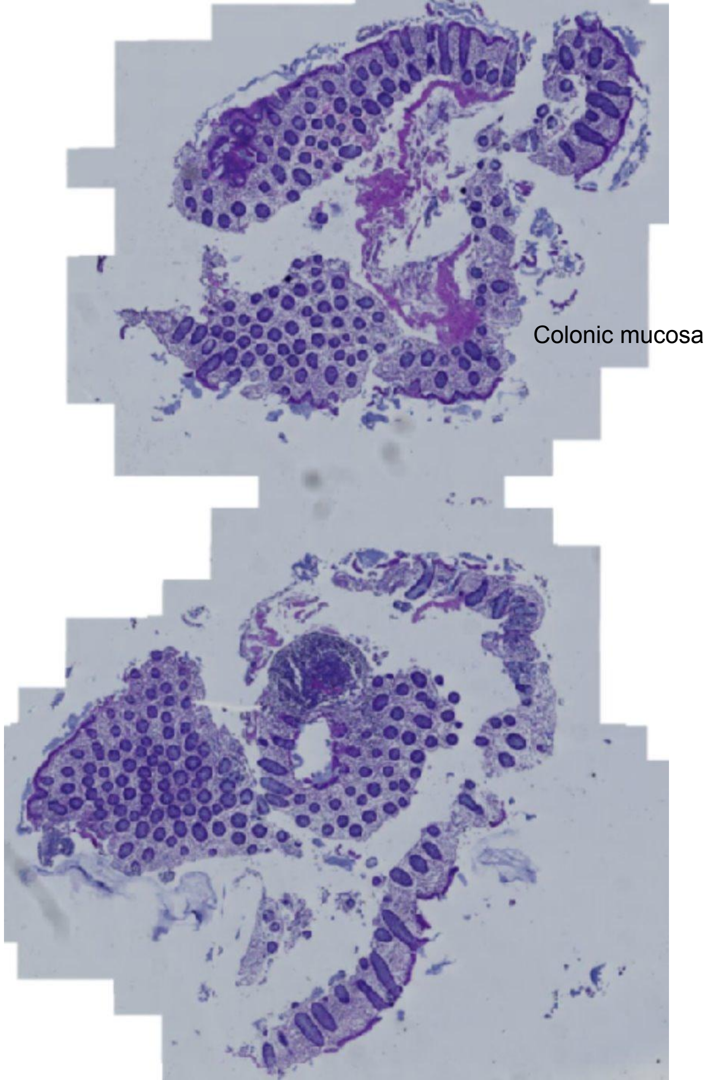

GI 3391

HEALTHY COLON

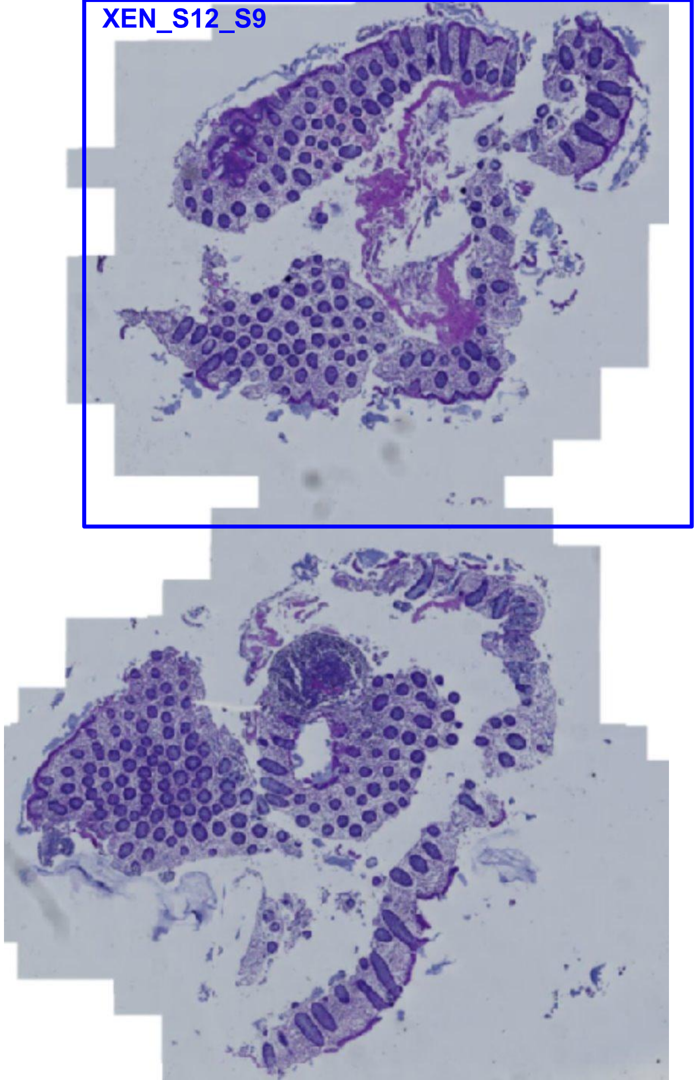

TIP 1071

HEALTHY COLON

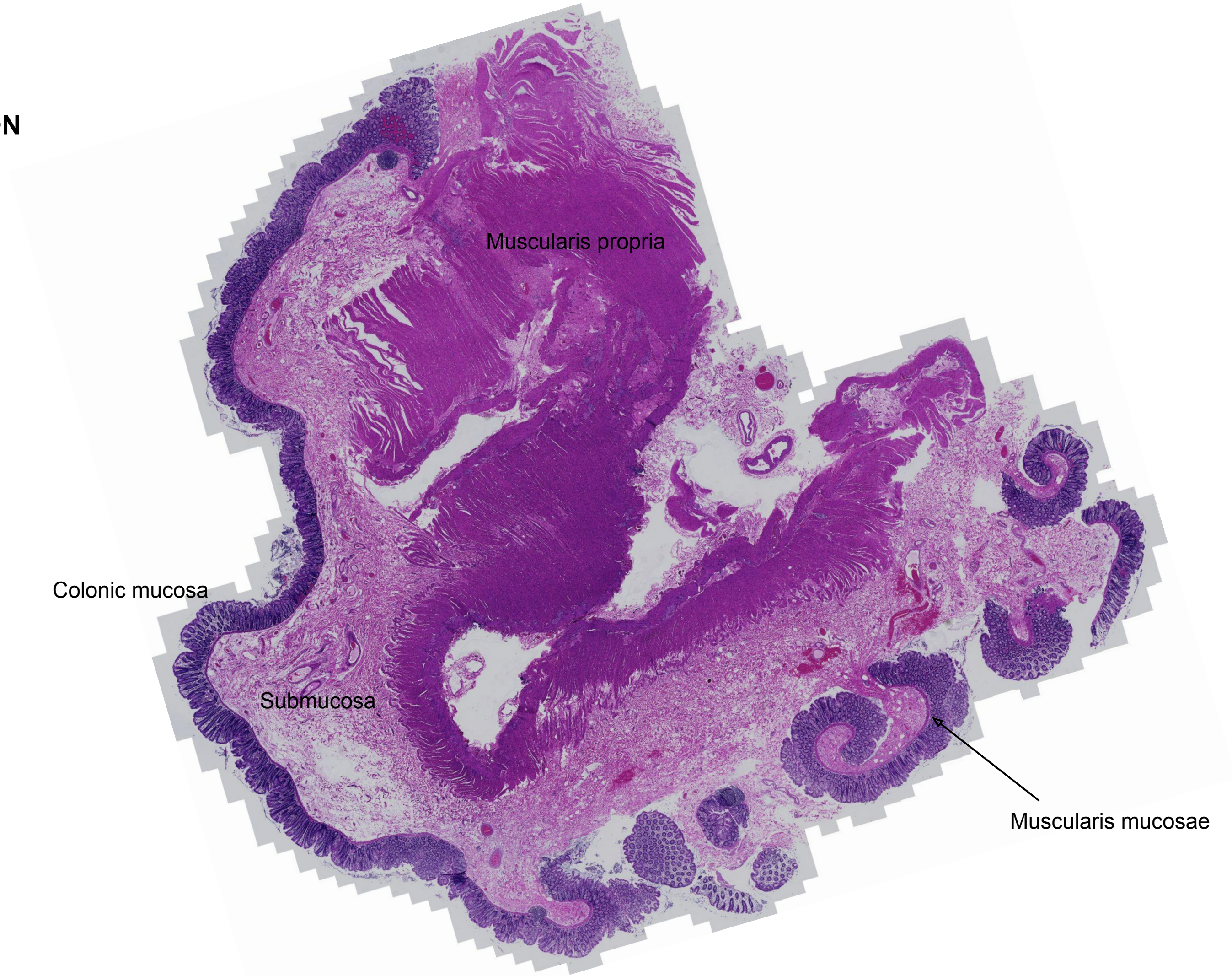

TIP 1071

HEALTHY COLON

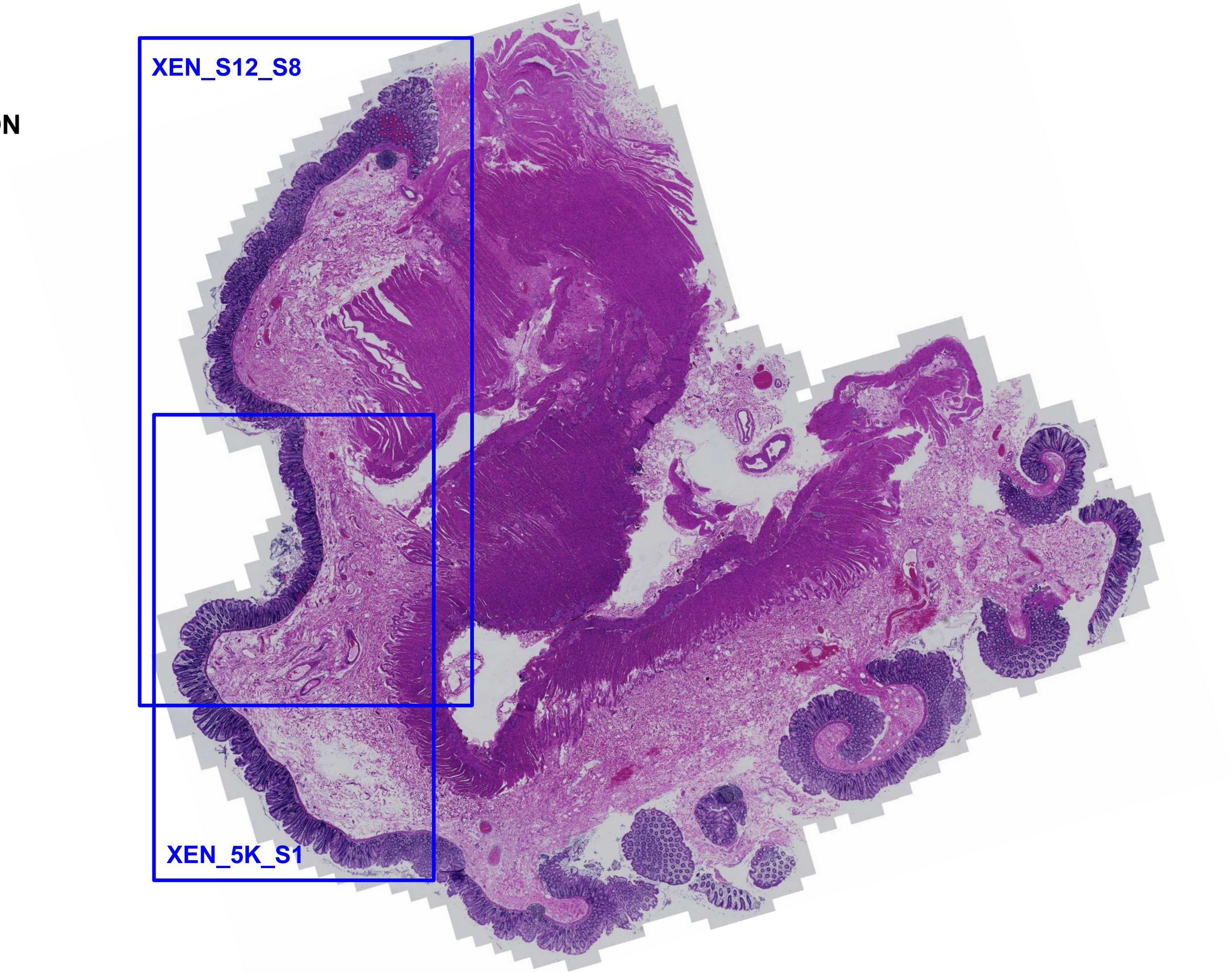

GI 9612

HEALTHY COLON

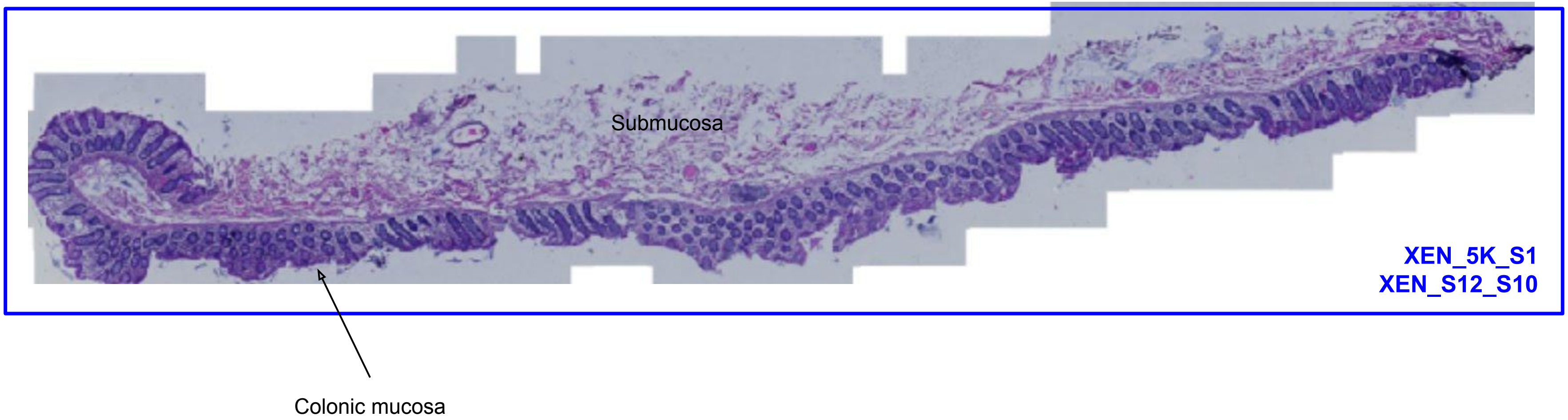

GI 11277

HEALTHY COLON

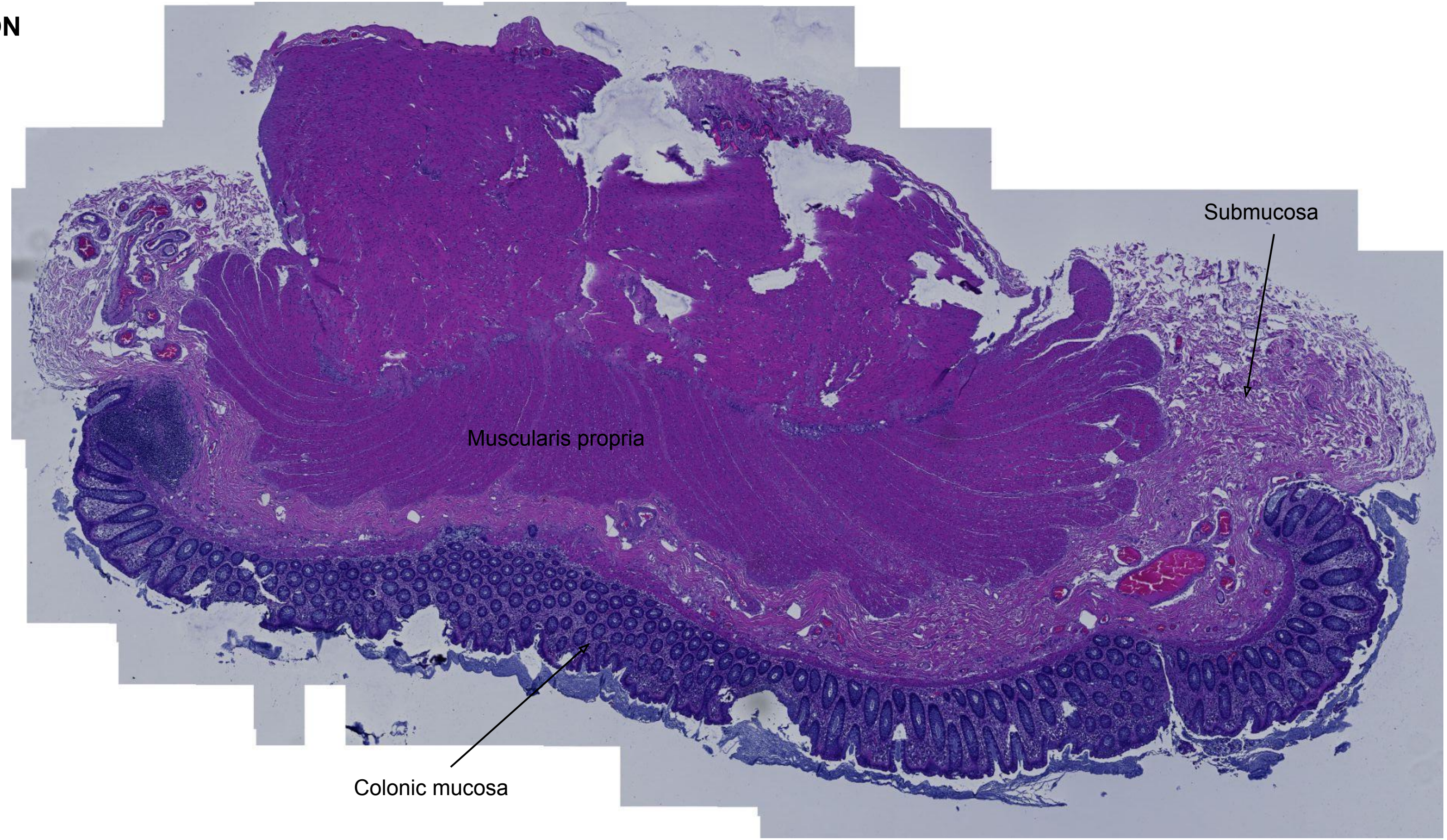

GI 11277

HEALTHY COLON

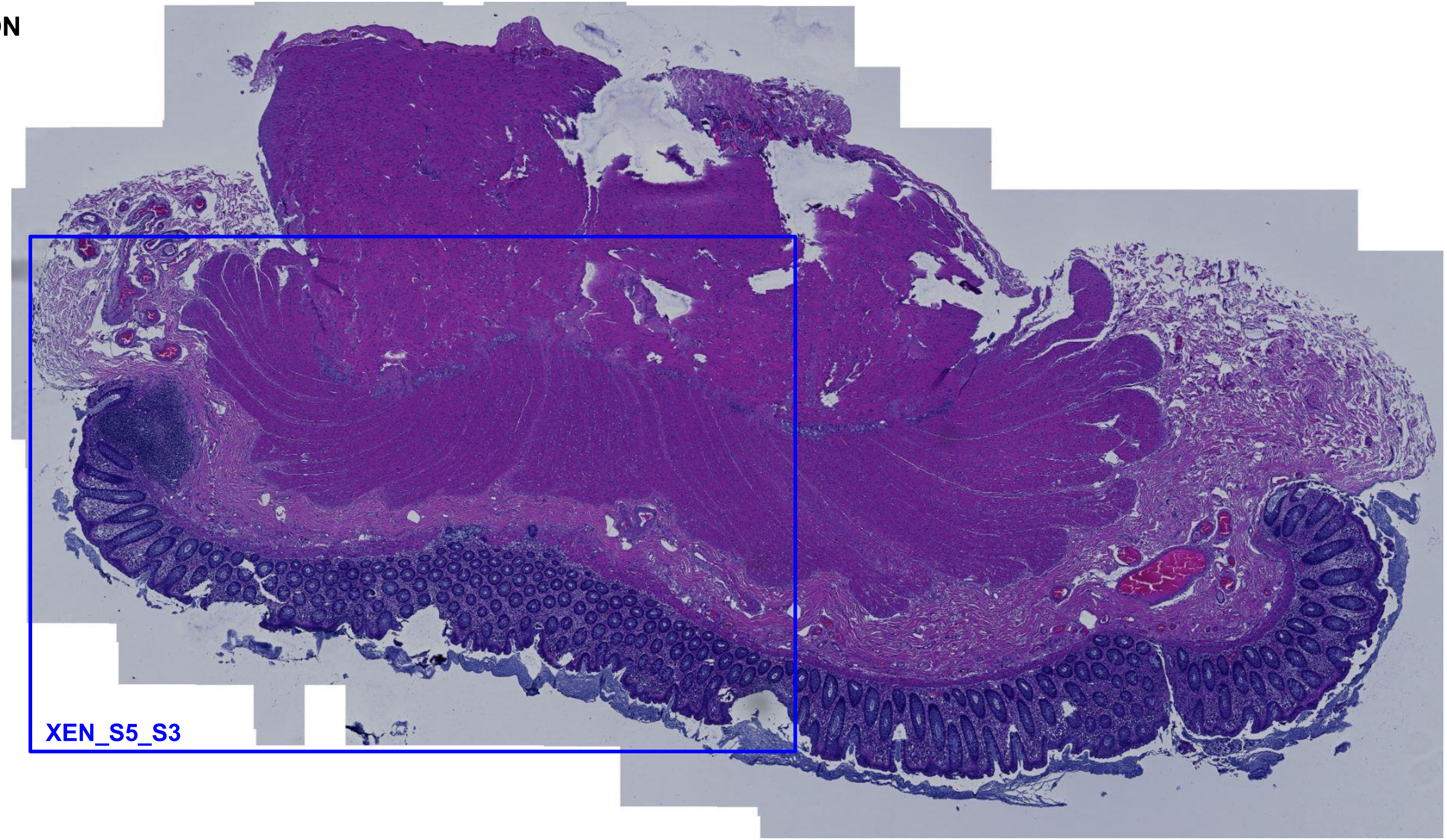

Supplement: Supplementary file 3 — Annotated PDF with H&E-stained tissue images for all study samples, with expert histopathology annotations indicating key morphological landmarks, ROIs, fistula tracts and other disease-related features. [file 41586_2025_9744_MOESM3_ESM.pdf]
